# Supplementary material for: Phytochemical and pharmacoinformatics analysis of a traditional antipsoriatic oil formulation for its potential against proinflammatory cytokines TNF-α and IL-17A
Source: PLoS One. 2025 Sep 2;20(9):e0330939. doi: 10.1371/journal.pone.0330939 (PMC12404448; doi:10.1371/journal.pone.0330939)

Analytical Research & Metallurgical Laboratories Pvt. Ltd.

Sample Information

Analyzed by	: Admin
Analyzed	: 11/10/2021 4:59:44 PM
Sample Type	: Unknown
Level #	1
Sample Name	: OIl sample VT
Sample ID	2042
IS Amount	: [1]=1
Vial #	6
Injection Volume	1
Data File	: D:\GCMS-QP2010+\Data\2021\Nov\10.11.2021\2042.qgd
Org Data File	: D:\GCMS-QP2010+\Data\2021\Nov\10.11.2021\2042.qgd
Method File	: D:\GCMS-QP2010+\Ullas\IADFAC SCAN.qgm
Org Method File	: D:\GCMS-QP2010+\Ullas\IADFAC SCAN.qgm

Chromatogram OIl sample VT D:\GCMS-QP2010+\Data\2021\Nov\10.11.2021\2042.qgd

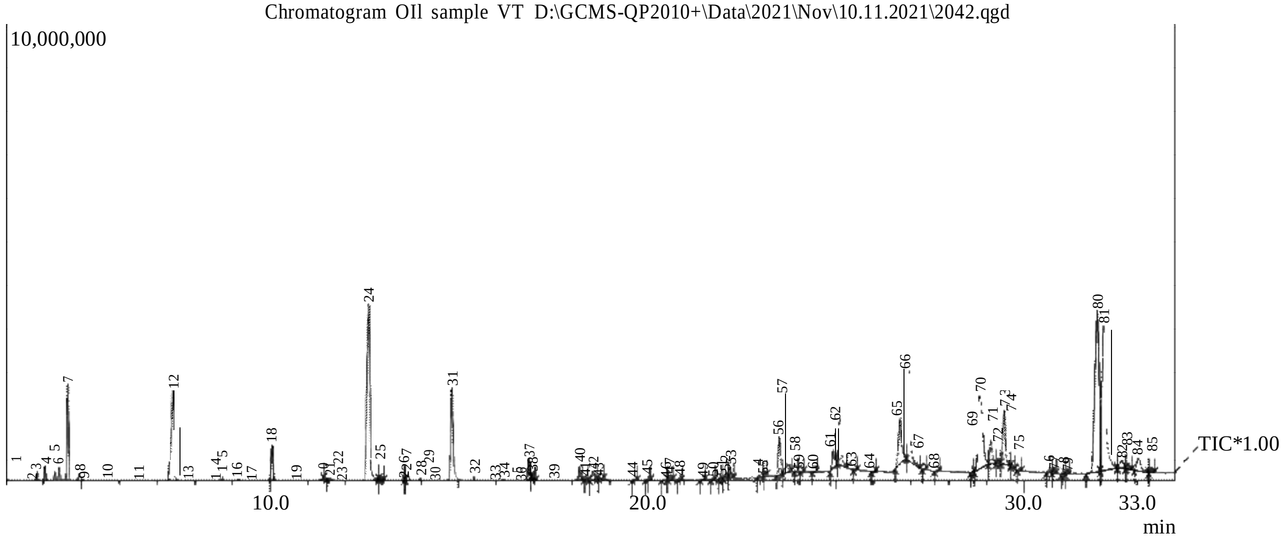

Peak Report TIC

Peak#	R.Time	I.Time	F.Time	Area	Area%	Name
1	3.151	3.033	3.217	266691	0.15	Hexanal
2	3.649	3.542	3.708	161412	0.09	2-Heptanone
3	3.813	3.708	3.908	926677	0.51	2-Pentanone, 5-methoxy-
4	4.035	3.967	4.125	1199828	0.66	3-Hydroxy-3-methylvaleric acid
5	4.273	4.158	4.333	888516	0.49	Hydroperoxide, 1-ethylbutyl
6	4.390	4.333	4.450	1062286	0.59	Hydroperoxide, 1-methylpentyl
7	4.611	4.533	4.767	6829714	3.77	3-Hexen-2-one
8	4.946	4.858	5.033	1012640	0.56	Cyclopropane, 1,1,2,3-tetramethyl-
9	5.083	5.033	5.150	119792	0.07	9-Oxabicyclo[6.1.0]nonane
10	5.657	5.508	5.733	193683	0.11	Nitrous acid, cyclohexyl ester
11	6.487	6.442	6.558	52015	0.03	Nonanal
12	7.411	7.242	7.667	11331810	6.26	Octanoic Acid
13	7.808	7.725	7.892	126447	0.07	Azulene
14	8.535	8.417	8.625	52614	0.03	2,4-Pentadien-1-ol, 3-pentyl-, (2Z)-
15	8.730	8.625	8.817	492536	0.27	2-Tridecenal, (E)-
16	9.162	9.100	9.292	329103	0.18	.delta. Nonalactone
17	9.501	9.433	9.567	52411	0.03	2,4-Decadienal, (E,E)-
18	10.054	9.950	10.350	3587699	1.98	n-Decanoic acid
19	10.688	10.550	10.767	183112	0.10	Z-2-Dodecenol
20	11.382	11.292	11.417	191643	0.11	Cycloheptasiloxane, tetradecamethyl-
21	11.561	11.500	11.700	469754	0.26	3-Isopropoxy-1,1,1,7,7,7-hexamethyl-3,5,5-tris(trimethylsiloxy)tetrasiloxane
22	11.772	11.725	11.833	54252	0.03	Dodecane, 2,6,11-trimethyl-
23	11.910	11.842	12.017	164029	0.09	2H-Pyran-2-one, tetrahydro-6-pentyl-
24	12.594	12.450	12.875	22121219	12.22	Dodecanoic acid
25	12.922	12.875	13.008	76335	0.04	Eicosanoic acid, ethyl ester
26	13.543	13.458	13.567	132557	0.07	Benzoic acid, 2,4-bis[(trimethylsilyl)oxy]-, trimethylsilyl ester

Peak#	R.Time	I.Time	F.Time	Area	Area%	Name
27	13.653	13.567	13.733	634564	0.35	Cyclooctasiloxane, hexadecamethyl-
28	13.985	13.917	14.083	444805	0.25	Butyl caprylate
29	14.193	14.083	14.292	158103	0.09	1,5-Anhydro-d-talitol
30	14.398	14.350	14.467	87087	0.05	.delta. Nonalactone
31	14.814	14.642	15.308	10448776	5.77	Tetradecanoic acid
32	15.406	15.317	15.592	656598	0.36	Octasiloxane, 1,1,3,3,5,5,7,7,9,9,11,11,13,13,15,15-hexadecamethyl-
33	15.948	15.875	16.083	153278	0.08	Silane, (butoxymethyl)trimethyl-
34	16.189	16.083	16.433	461015	0.25	Hexadecanoic acid, 1-(hydroxymethyl)-1,2-ethanediyl ester
35	16.504	16.433	16.567	83791	0.05	Octadecanoic acid, 10-methyl-, methyl ester
36	16.636	16.567	16.733	76239	0.04	2H-Pyran-2-one, tetrahydro-6-tridecyl-
37	16.858	16.792	16.925	1602967	0.89	Pentadecanoic acid
38	16.963	16.925	17.042	283796	0.16	Octasiloxane, 1,1,3,3,5,5,7,7,9,9,11,11,13,13,15,15-hexadecamethyl-
39	17.486	17.408	17.650	159180	0.09	Dodecanoic acid, 2,3-bis(acetyloxy)propyl ester
40	18.192	18.108	18.275	1239813	0.68	Dodecanoic acid, 2,3-dihydroxypropyl ester
41	18.376	18.333	18.442	155726	0.09	Octasiloxane, 1,1,3,3,5,5,7,7,9,9,11,11,13,13,15,15-hexadecamethyl-
42	18.560	18.475	18.683	989280	0.55	Oleic Acid
43	18.743	18.692	18.850	173312	0.10	Octadecanoic acid
44	19.655	19.583	19.717	174820	0.10	Octasiloxane, 1,1,3,3,5,5,7,7,9,9,11,11,13,13,15,15-hexadecamethyl-
45	20.025	19.917	20.100	339743	0.19	Hexadecanoic acid, 2-hydroxy-1-(hydroxymethyl)ethyl ester
46	20.468	20.358	20.517	72863	0.04	Vinyl caprylate
47	20.594	20.517	20.675	263305	0.15	Glycerol tricaprylate
48	20.832	20.767	20.908	187589	0.10	Octasiloxane, 1,1,3,3,5,5,7,7,9,9,11,11,13,13,15,15-hexadecamethyl-
49	21.452	21.383	21.517	56121	0.03	Naphthalene, decahydro-1,4a-dimethyl-7-(1-methylethyl)-, [1S-(1.alpha.,4a.alpha.,7.alpha
50	21.715	21.650	21.775	87273	0.05	5-Octadecenal
51	21.936	21.883	21.983	130391	0.07	Octasiloxane, 1,1,3,3,5,5,7,7,9,9,11,11,13,13,15,15-hexadecamethyl-
52	22.094	21.992	22.125	157560	0.09	Hexanoic acid, 4-tridecyl ester
53	22.193	22.125	22.283	849587	0.47	Hexanoic acid, 3-tridecyl ester
54	22.967	22.900	23.050	220848	0.12	Octasiloxane, 1,1,3,3,5,5,7,7,9,9,11,11,13,13,15,15-hexadecamethyl-
55	23.116	23.058	23.150	60706	0.03	3,3,7,11-Tetramethyltricyclo[5.4.0.0(4,11)]undecan-1-ol
56	23.509	23.408	23.575	3172744	1.75	Dodecanoic acid, 1-(hydroxymethyl)-1,2-ethanediyl ester
57	23.640	23.575	23.817	6829556	3.77	Dodecanoic acid, 1-(hydroxymethyl)-1,2-ethanediyl ester
58	23.927	23.883	23.975	71703	0.04	Cyclononasiloxane, octadecamethyl-
59	24.069	24.025	24.142	61985	0.03	Heptasiloxane, 1,1,3,3,5,5,7,7,9,9,11,11,13,13-tetradecamethyl-
60	24.419	24.358	24.492	56358	0.03	Cyclotrisiloxane, hexamethyl-
61	24.935	24.842	25.033	2049287	1.13	Octadecanoic acid, 2,3-bis[(1-oxotetradecyl)oxy]propyl ester
62	25.107	25.033	25.292	4399543	2.43	Octadecanoic acid, 2,3-bis[(1-oxotetradecyl)oxy]propyl ester
63	25.499	25.458	25.558	57835	0.03	3-Ethoxy-1,1,1,5,5,5-hexamethyl-3-(trimethylsiloxy)trisiloxane
64	26.006	25.942	26.075	86910	0.05	Cyclotrisiloxane, hexamethyl-
65	26.709	26.583	26.858	5227678	2.89	Dodecanoic acid, 1-(hydroxymethyl)-1,2-ethanediyl ester
66	26.950	26.858	27.225	9374963	5.18	Dodecanoic acid, 1-(hydroxymethyl)-1,2-ethanediyl ester
67	27.335	27.275	27.392	70822	0.04	Octasiloxane, 1,1,3,3,5,5,7,7,9,9,11,11,13,13,15,15-hexadecamethyl-
68	27.690	27.592	27.733	51976	0.03	Octasiloxane, 1,1,3,3,5,5,7,7,9,9,11,11,13,13,15,15-hexadecamethyl-
69	28.629	28.567	28.675	97706	0.05	Silane, 1,4-phenylenebis[trimethyl-
70	28.829	28.683	29.042	12937492	7.15	Glycerol tricaprylate
71	29.122	29.042	29.250	2430778	1.34	Tetradecanoic acid, 2-hydroxy-1,3-propanediyl ester
72	29.307	29.250	29.358	110098	0.06	3-t-Butyl-oct-6-en-1-ol
73	29.465	29.358	29.633	5928664	3.27	Tetradecanoic acid, 2-hydroxy-1,3-propanediyl ester
74	29.671	29.642	29.750	78878	0.04	1-Monolinoleoylglycerol trimethylsilyl ether
75	29.842	29.800	29.917	71338	0.04	Cyclotrisiloxane, hexamethyl-
76	30.642	30.567	30.725	208112	0.11	
77	30.782	30.725	30.842	116933	0.06	Cyclotrisiloxane, hexamethyl-
78	31.016	30.967	31.058	100426	0.06	Silicic acid, diethyl bis(trimethylsilyl) ester
79	31.130	31.058	31.183	81539	0.05	Hexasiloxane, 1,1,3,3,5,5,7,7,9,9,11,11-dodecamethyl-
80	31.934	31.633	32.017	26290314	14.52	Decanoic acid, 1,2,3-propanetriyl ester
81	32.101	32.017	32.467	25162448	13.90	Dodecanoic acid, 1,2,3-propanetriyl ester
82	32.579	32.467	32.700	1146360	0.63	Lupeol
83	32.736	32.700	32.842	57773	0.03	3-Isopropoxy-1,1,1,5,5,5-hexamethyl-3-(trimethylsiloxy)trisiloxane
84	33.036	32.917	33.300	2133579	1.18	Hexadecanoic acid, 1-(hydroxymethyl)-1,2-ethanediyl ester
85	33.376	33.300	33.450	96591	0.05	Tetrasiloxane, decamethyl-
				181048300	100.00	

Library

<< Target >>
Line#:1 R.Time:3.150(Scan#:19) MassPeaks:106
RawMode:Averaged 3.142-3.158(18-20) BasePeak:44.00(8417)
BG Mode:Calc. from Peak Group 1 - Event 1

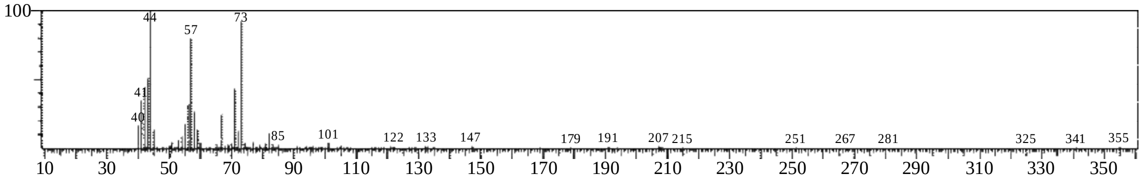

Hit#:1 Entry:1786 Library:NIST05s.LIB
SI:80 Formula:C6H12O CAS:66-25-1 MolWeight:100 RetIndex:806
CompName:Hexanal $$ n-Caproaldehyde $$ n-Hexanal $$ Caproaldehyde $$ Caproic aldehyde $$ Capronaldehyde $$ Hexaldehyde $$ n-C5H11CHO $$ 1-Hex

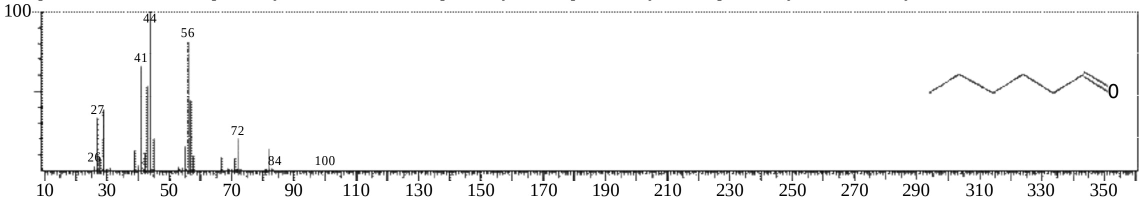

Hit#:2 Entry:1803 Library:NIST05s.LIB
SI:80 Formula:C6H12O CAS:24070-77-7 MolWeight:100 RetIndex:849
CompName:Cyclopentanol, 2-methyl- $$ 2-Methylcyclopentanol $$ 2-Methylcyclopentyl alcohol $$

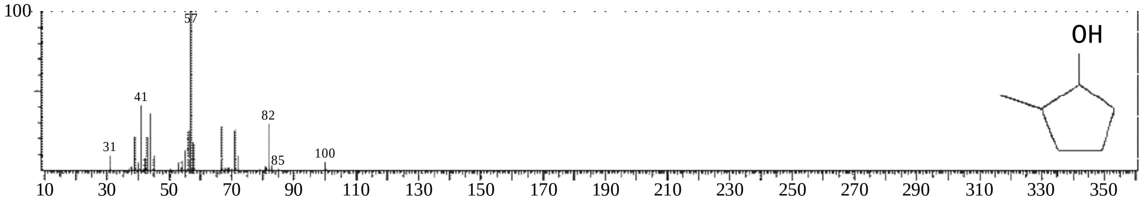

Hit#:3 Entry:1968 Library:NIST05.LIB
SI:79 Formula:C6H12O CAS:66-25-1 MolWeight:100 RetIndex:806
CompName:Hexanal $$ n-Caproaldehyde $$ n-Hexanal $$ Caproaldehyde $$ Caproic aldehyde $$ Capronaldehyde $$ Hexaldehyde $$ n-C5H11CHO $$ 1-Hex

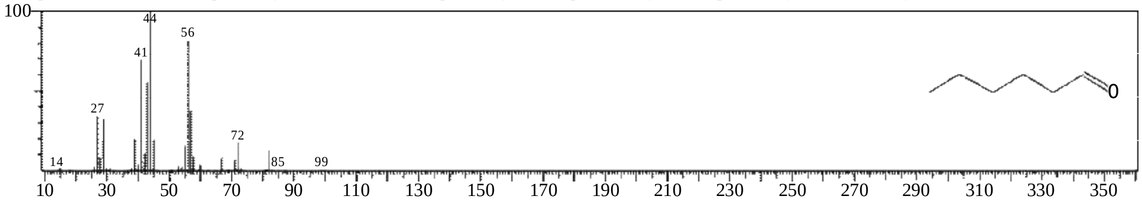

Hit#:4 Entry:2003 Library:NIST05.LIB
SI:79 Formula:C6H12O CAS:25144-05-2 MolWeight:100 RetIndex:849
CompName:Cyclopentanol, 2-methyl-, cis- $$ cis-2-Methylcyclopentanol $$ 2-Methylcyclopentanol # $$

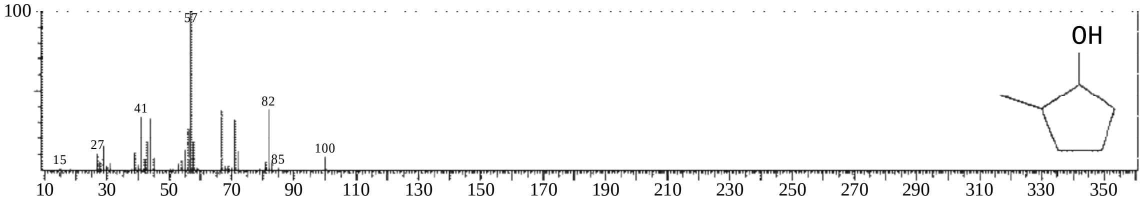

Hit#:5 Entry:74195 Library:NIST05.LIB
SI:79 Formula:C15H29NO2 CAS:2016-05-9 MolWeight:255 RetIndex:1776
CompName:Methanamine, 1,1-bis(cyclohexyloxy)-N,N-dimethyl- $$ N,N-Dimethylformamide dicyclohexyl acetal $$ N-[Bis(cyclohexyloxy)methyl]-N,N-dimet

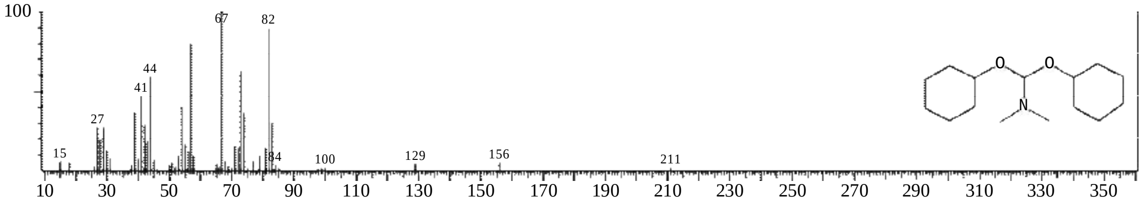

<< Target >>
Line#:2 R.Time:3.650(Scan#:79) MassPeaks:84
RawMode:Averaged 3.642-3.658(78-80) BasePeak:43.05(29238)
BG Mode:Calc. from Peak Group 1 - Event 1

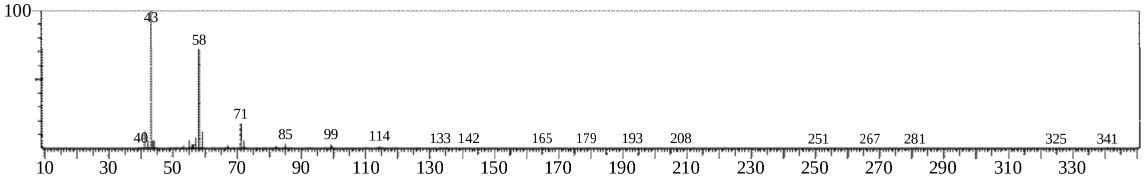

Hit#:1 Entry:3199 Library:NIST05s.LIB
SI:94 Formula:C7H14O CAS:110-43-0 MolWeight:114 RetIndex:853
CompName:2-Heptanone $$ n-Amyl methyl ketone $$ n-Pentyl methyl ketone $$ Amyl methyl ketone $$ Butylacetone $$ Heptan-2-one $$ Methyl amyl ketone

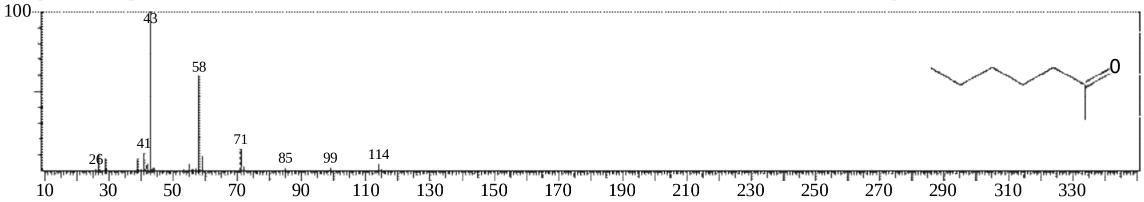

Hit#:2 Entry:3197 Library:NIST05s.LIB
SI:93 Formula:C7H14O CAS:110-43-0 MolWeight:114 RetIndex:853
CompName:2-Heptanone $$ n-Amyl methyl ketone $$ n-Pentyl methyl ketone $$ Amyl methyl ketone $$ Butylacetone $$ Heptan-2-one $$ Methyl amyl ketone

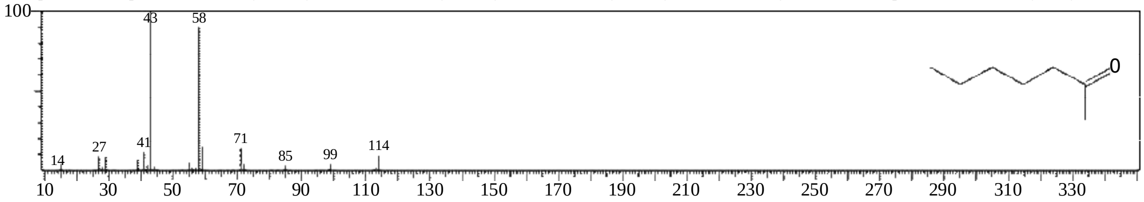

Hit#:3 Entry:4923 Library:NIST05s.LIB
SI:93 Formula:C8H16O CAS:111-13-7 MolWeight:128 RetIndex:952
CompName:2-Octanone $$ n-Hexyl methyl ketone $$ Hexyl methyl ketone $$ Methyl hexyl ketone $$ Methyl n-hexyl ketone $$ Octan-2-one $$ n-C6H13COC

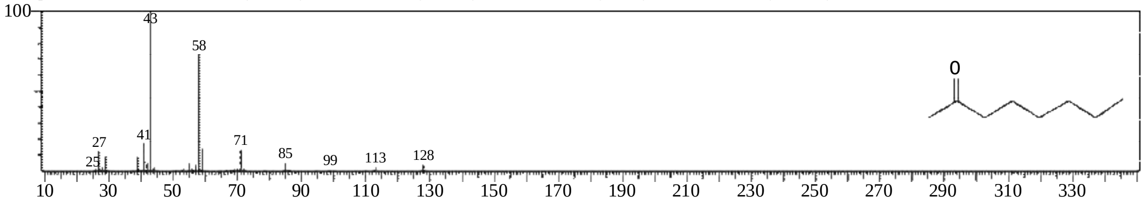

Hit#:4 Entry:4062 Library:NIST05.LIB
SI:93 Formula:C7H14O CAS:110-43-0 MolWeight:114 RetIndex:853
CompName:2-Heptanone $$ n-Amyl methyl ketone $$ n-Pentyl methyl ketone $$ Amyl methyl ketone $$ Butylacetone $$ Heptan-2-one $$ Methyl amyl ketone

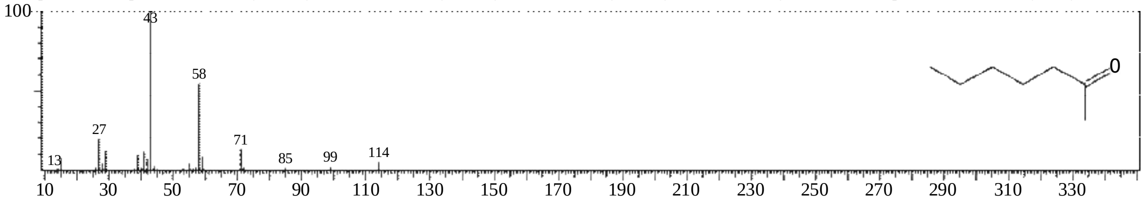

Hit#:5 Entry:4063 Library:NIST05.LIB
SI:92 Formula:C7H14O CAS:105-42-0 MolWeight:114 RetIndex:789
CompName:2-Hexanone, 4-methyl- $$ Methyl 2-methylbutyl ketone $$ 4-Methyl-2-hexanone $$

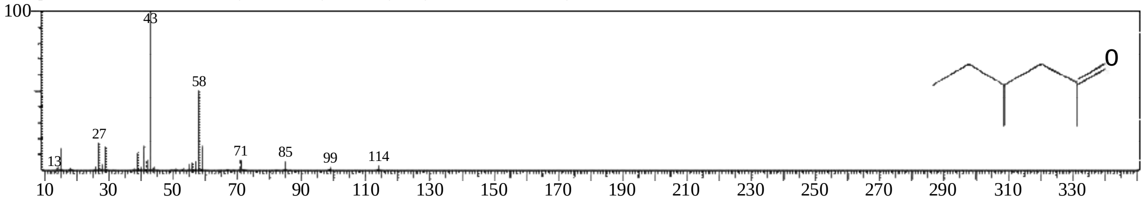

<< Target >>
Line#:3 R.Time:3.817(Scan#:99) MassPeaks:122
RawMode:Averaged 3.808-3.825(98-100) BasePeak:43.05(120627)
BG Mode:Calc. from Peak Group 1 - Event 1

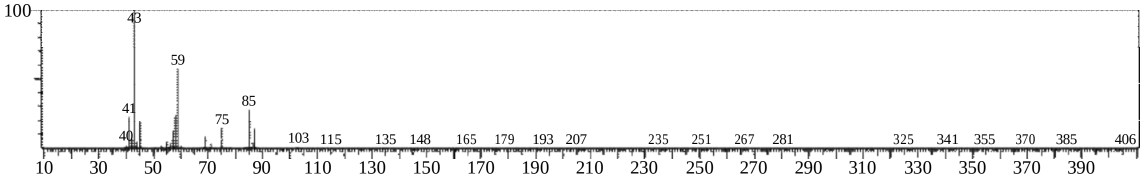

Hit#:1 Entry:4464 Library:NIST05.LIB
SI:85 Formula:C6H12O2 CAS:17429-04-8 MolWeight:116 RetIndex:830
CompName:2-Pentanone, 5-methoxy- $$ 5-Methoxy-2-pentanone # $$

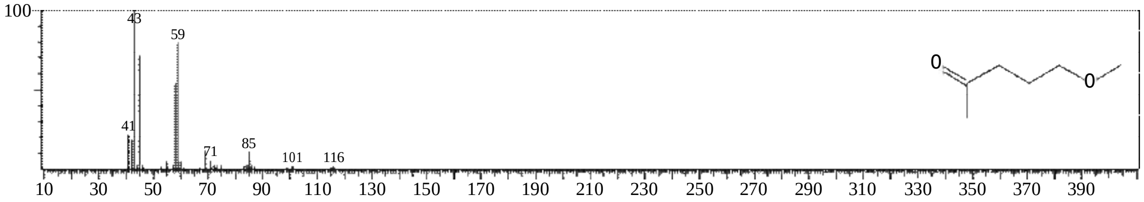

Hit#:2 Entry:3429 Library:NIST05s.LIB
SI:85 Formula:C6H12O2 CAS:3126-95-2 MolWeight:116 RetIndex:784
CompName:Oxirane, (propoxymethyl)- $$ Propane, 1,2-epoxy-3-propoxy- $$ Glycidyl propyl ether $$ Propyl glycidyl ether $$ (Propoxymethyl)oxirane $$ 1,2-

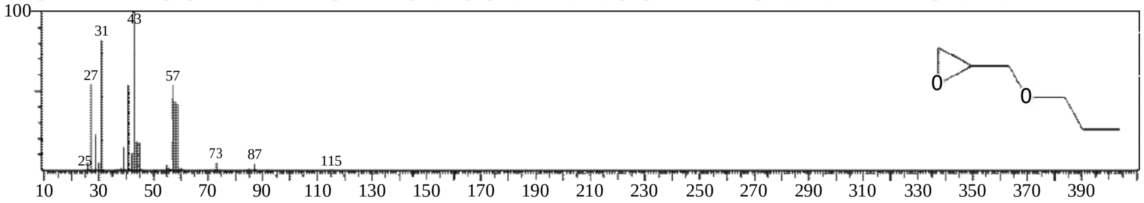

Hit#:3 Entry:18688 Library:NIST05.LIB
SI:85 Formula:C9H18O2 CAS:102840-52-8 MolWeight:158 RetIndex:1009
CompName:Pentan-2-ol, 4-allyloxy-2-methyl- $$ 4-(Allyloxy)-2-methyl-2-pentanol # $$

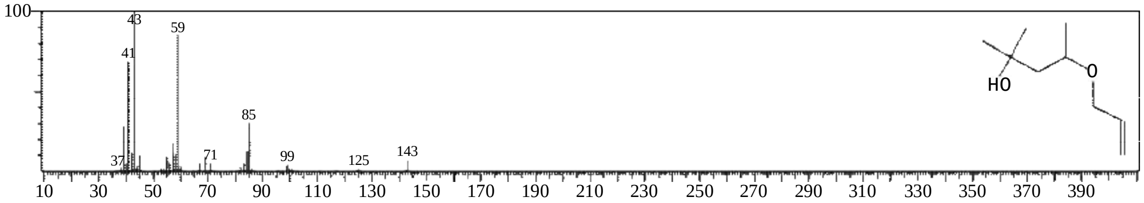

Hit#:4 Entry:4452 Library:NIST05.LIB
SI:84 Formula:C6H12O2 CAS:3126-95-2 MolWeight:116 RetIndex:784
CompName:Oxirane, (propoxymethyl)- $$ Propane, 1,2-epoxy-3-propoxy- $$ Glycidyl propyl ether $$ Propyl glycidyl ether $$ (Propoxymethyl)oxirane $$ 1,2-

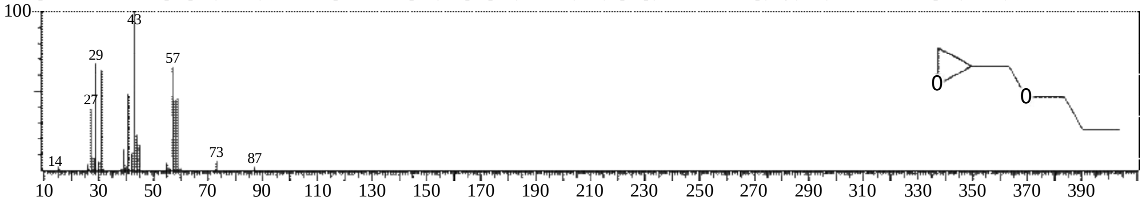

Hit#:5 Entry:4462 Library:NIST05.LIB
SI:84 Formula:C6H12O2 CAS:25910-96-7 MolWeight:116 RetIndex:815
CompName:3-Oxetanol, 2,2,3-trimethyl- $$ 2,2,3-Trimethyl-3-oxetanol # $$

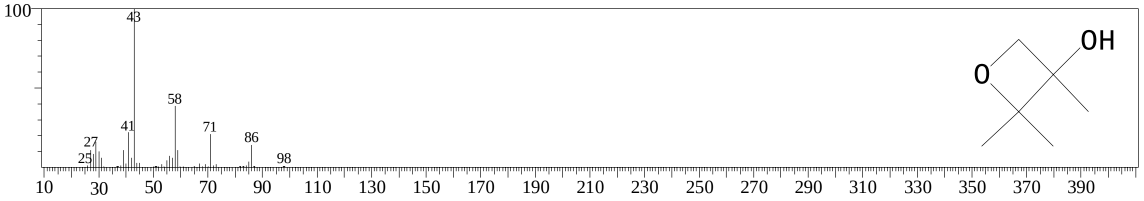

<< Target >>
Line#:4 R.Time:4.033(Scan#:125) MassPeaks:135
RawMode:Averaged 4.025-4.042(124-126) BasePeak:43.05(185482)
BG Mode:Calc. from Peak Group 1 - Event 1

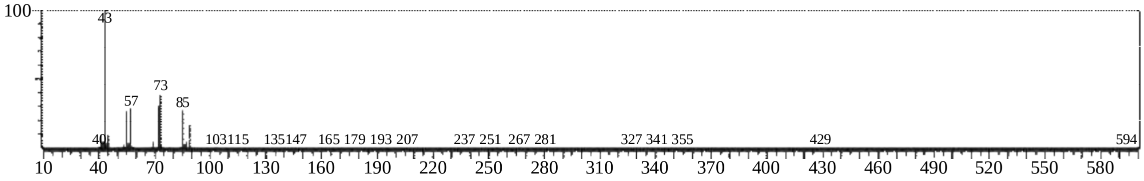

Hit#:1 Entry:8249 Library:NIST05.LIB
SI:84 Formula:C6H12O3 CAS:150-96-9 MolWeight:132 RetIndex:1066
CompName:3-Hydroxy-3-methylvaleric acid $$ 3-Hydroxy-3-methylpentanoic acid # $$

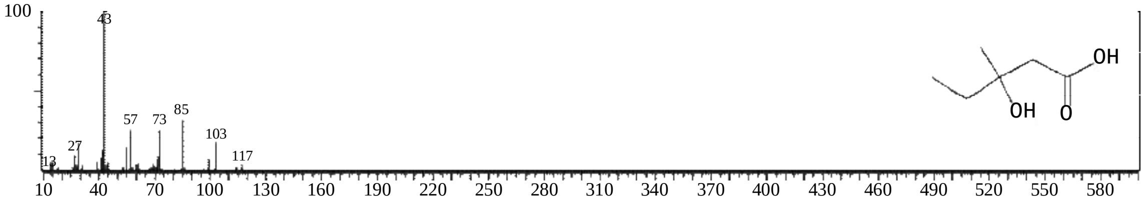

Hit#:2 Entry:7145 Library:NIST05.LIB
SI:82 Formula:C8H16O CAS:19550-10-8 MolWeight:128 RetIndex:824
CompName:2-Hexanone, 3,4-dimethyl- $$ 3,4-Dimethyl-2-hexanone $$

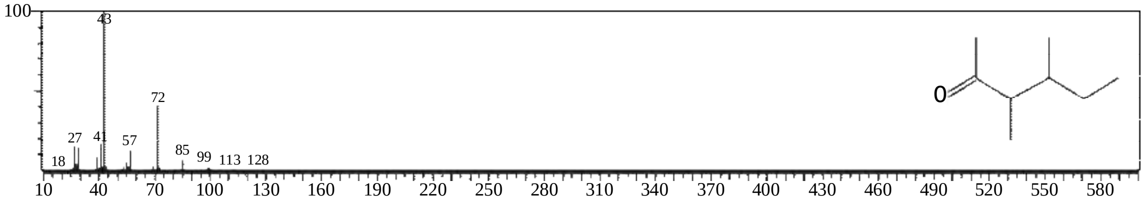

Hit#:3 Entry:12580 Library:NIST05.LIB
SI:82 Formula:C8H16O2 CAS:30536-44-8 MolWeight:144 RetIndex:1028
CompName:4-Butoxy-2-butanone

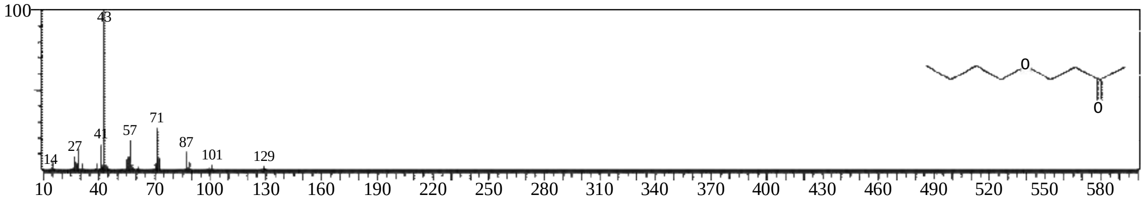

Hit#:4 Entry:7144 Library:NIST05.LIB
SI:82 Formula:C8H16O CAS:2371-19-9 MolWeight:128 RetIndex:888
CompName:2-Heptanone, 3-methyl- $$ 3-Methyl-2-heptanone $$ 3-Methylheptan-2-one $$

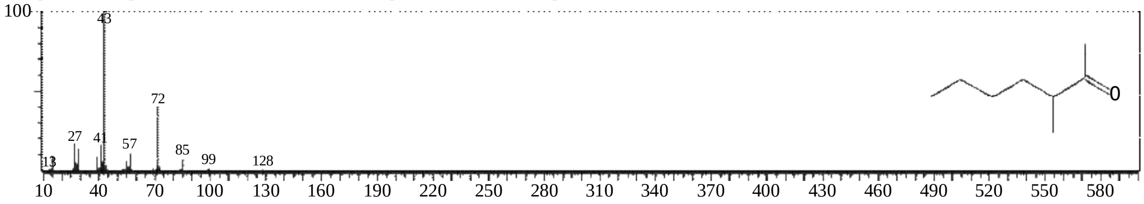

Hit#:5 Entry:4059 Library:NIST05.LIB
SI:82 Formula:C7H14O CAS:26254-92-2 MolWeight:114 RetIndex:777
CompName:Butanal, 2-ethyl-3-methyl- $$ (CH3)2CHCH(C2H5)CHO $$ 2-Ethylisovaleraldehyde $$ 2-Ethyl-3-methylbutanal # $$

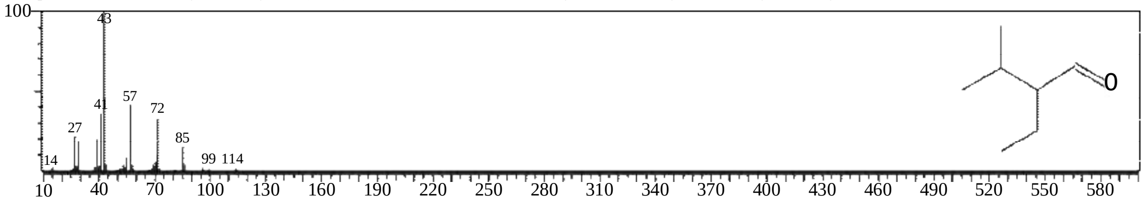

<< Target >>
Line#:5 R.Time:4.275(Scan#:154) MassPeaks:122
RawMode:Averaged 4.267-4.283(153-155) BasePeak:43.05(91747)
BG Mode:Calc. from Peak Group 1 - Event 1

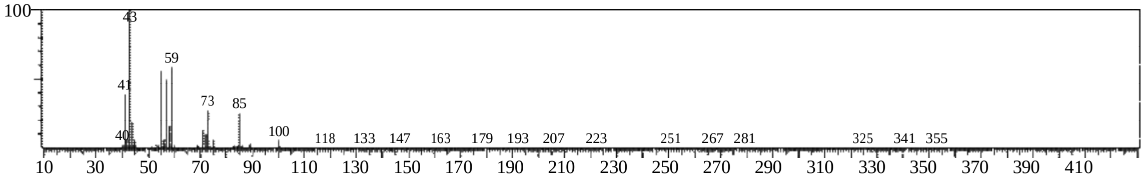

Hit#:1 Entry:4857 Library:NIST05.LIB
SI:92 Formula:C6H14O2 CAS:24254-56-6 MolWeight:118 RetIndex:914
CompName:Hydroperoxide, 1-ethylbutyl $$ 3-Hexyl hydroperoxide $$ 1-Ethylbutyl hydroperoxide $$ 3-Hydroperoxyhexane $$

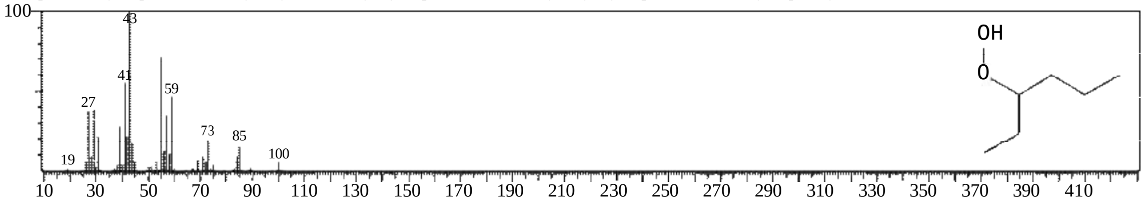

Hit#:2 Entry:4465 Library:NIST05.LIB
SI:86 Formula:C6H12O2 CAS:0-00-0 MolWeight:116 RetIndex:861
CompName:(2,3,3-Trimethyloxiranyl)methanol

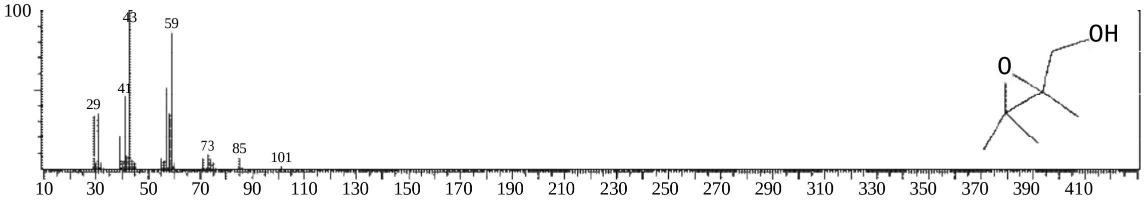

Hit#:3 Entry:4452 Library:NIST05.LIB
SI:84 Formula:C6H12O2 CAS:3126-95-2 MolWeight:116 RetIndex:784
CompName:Oxirane, (propoxymethyl)- $$ Propane, 1,2-epoxy-3-propoxy- $$ Glycidyl propyl ether $$ Propyl glycidyl ether $$ (Propoxymethyl)oxirane $$ 1,2-

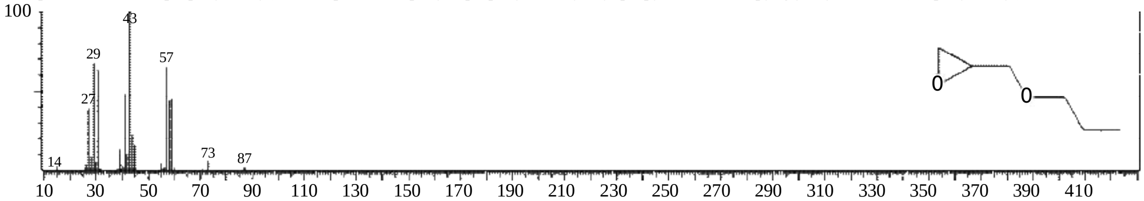

Hit#:4 Entry:3429 Library:NIST05s.LIB
SI:84 Formula:C6H12O2 CAS:3126-95-2 MolWeight:116 RetIndex:784
CompName:Oxirane, (propoxymethyl)- $$ Propane, 1,2-epoxy-3-propoxy- $$ Glycidyl propyl ether $$ Propyl glycidyl ether $$ (Propoxymethyl)oxirane $$ 1,2-

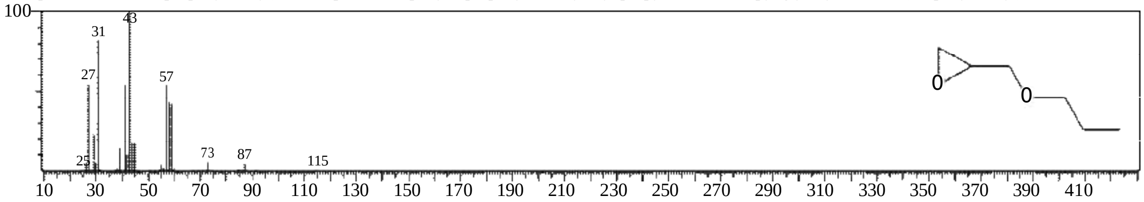

Hit#:5 Entry:4463 Library:NIST05.LIB
SI:83 Formula:C6H12O2 CAS:4016-14-2 MolWeight:116 RetIndex:720
CompName:Oxirane, [(1-methylethoxy)methyl]- $$ Propane, 1,2-epoxy-3-isopropoxy- $$ (Isopropoxymethyl)oxirane $$ Glycidyl isopropyl ether $$ Isopropyl g

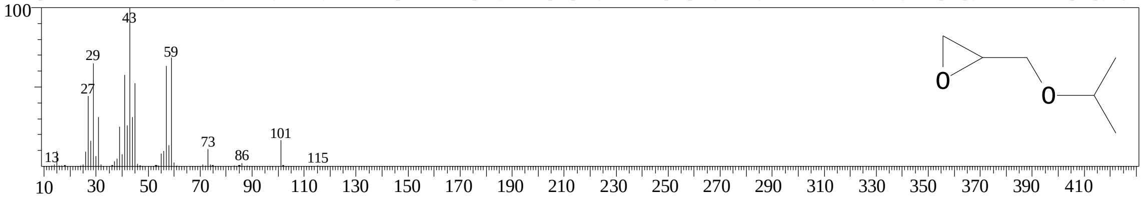

<< Target >>
Line#:6 R.Time:4.392(Scan#:168) MassPeaks:122
RawMode:Averaged 4.383-4.400(167-169) BasePeak:45.05(131019)
BG Mode:Calc. from Peak Group 1 - Event 1

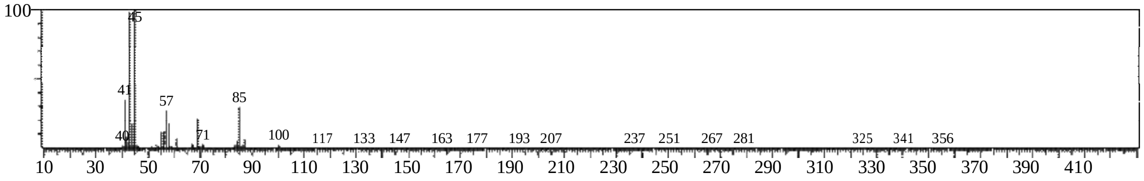

Hit#:1 Entry:4855 Library:NIST05.LIB
SI:94 Formula:C6H14O2 CAS:24254-55-5 MolWeight:118 RetIndex:914
CompName:Hydroperoxide, 1-methylpentyl $$ 2-Hexyl hydroperoxide $$ 1-Methylpentyl hydroperoxide $$ n-C4H9CH(CH3)OOH $$ 2-Hydroperoxyhexane $

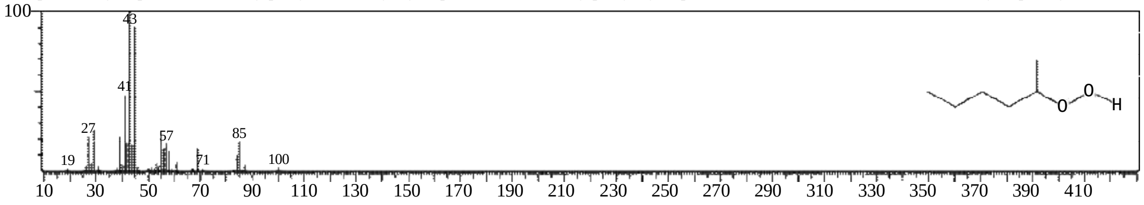

Hit#:2 Entry:7851 Library:NIST05.LIB
SI:90 Formula:C8H18O CAS:19550-05-1 MolWeight:130 RetIndex:851
CompName:2-Hexanol, 3,4-dimethyl- $$ 3,4-Dimethyl-2-hexanol $$

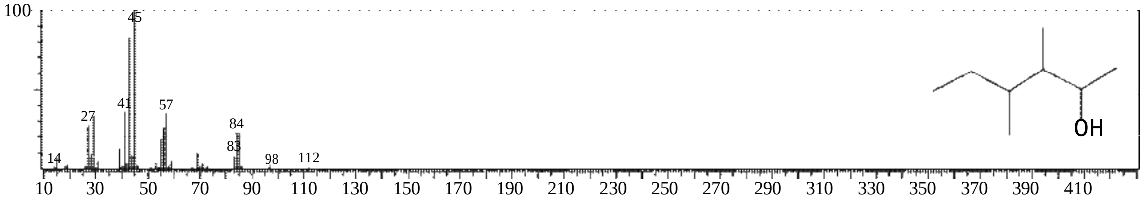

Hit#:3 Entry:26694 Library:NIST05.LIB
SI:88 Formula:C9H18O3 CAS:145747-16-6 MolWeight:174 RetIndex:1159
CompName:Methoxyacetic acid, hexyl ester $$ Hexyl methoxyacetate # $$

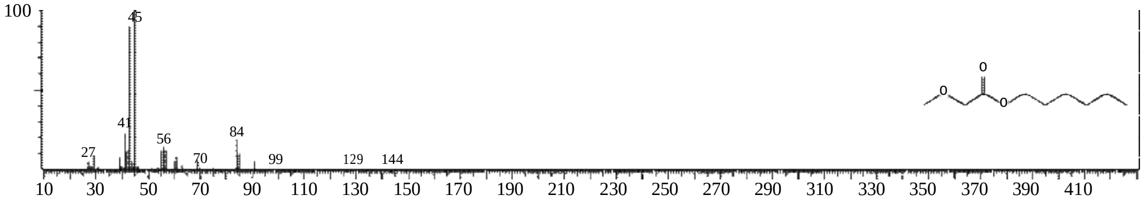

Hit#:4 Entry:8303 Library:NIST05.LIB
SI:88 Formula:C7H16O2 CAS:762-46-9 MolWeight:132 RetIndex:1013
CompName:Hydroperoxide, 1-methylhexyl $$ n-C5H11CH(CH3)OOH $$ 1-Methylhexyl hydroperoxide # $$

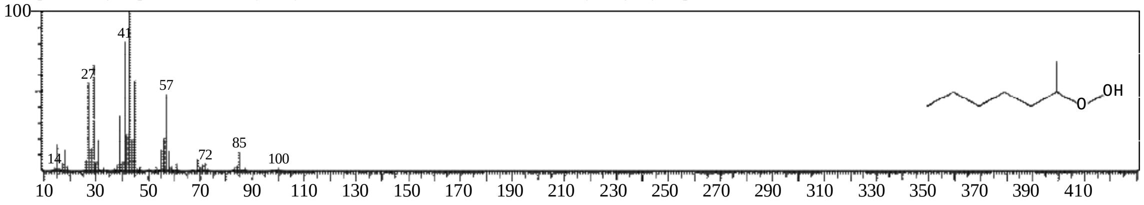

Hit#:5 Entry:5333 Library:NIST05s.LIB
SI:86 Formula:C8H18O CAS:19550-05-1 MolWeight:130 RetIndex:851
CompName:2-Hexanol, 3,4-dimethyl- $$ 3,4-Dimethyl-2-hexanol $$

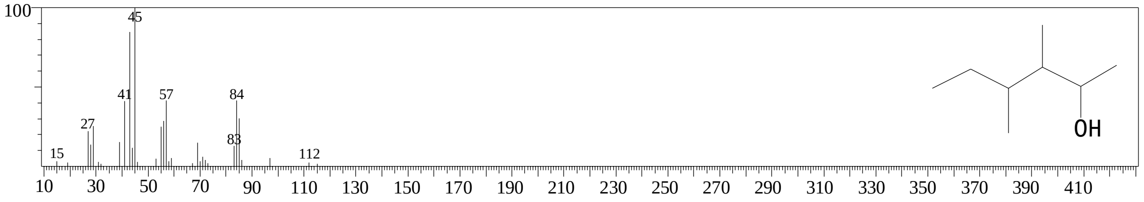

<< Target >>
Line#:7 R.Time:4.608(Scan#:194) MassPeaks:212
RawMode:Averaged 4.600-4.617(193-195) BasePeak:83.10(842633)
BG Mode:Calc. from Peak Group 1 - Event 1

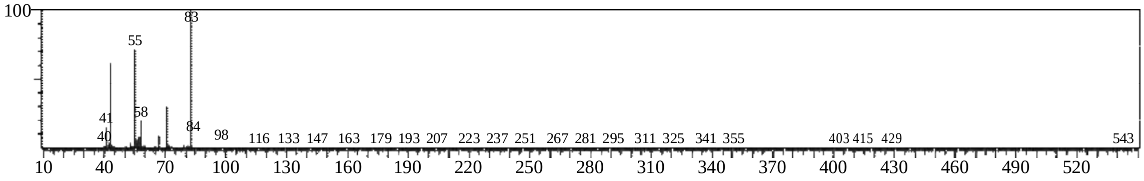

Hit#:1 Entry:1553 Library:NIST05s.LIB
SI:87 Formula:C6H10O CAS:763-93-9 MolWeight:98 RetIndex:762
CompName:3-Hexen-2-one $$ 1-Butenyl methyl ketone $$ Methyl 1-butenyl ketone $$ (3E)-3-Hexen-2-one # $$

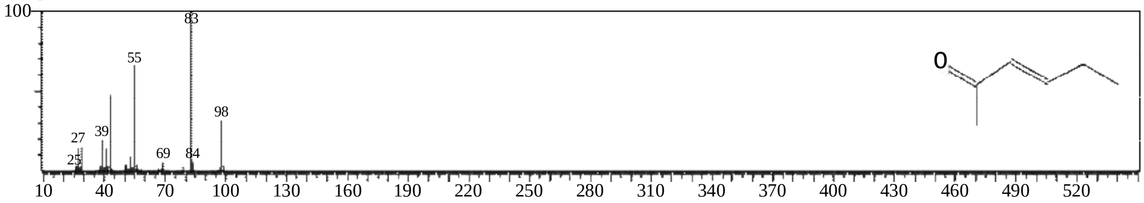

Hit#:2 Entry:1672 Library:NIST05.LIB
SI:87 Formula:C6H10O CAS:763-93-9 MolWeight:98 RetIndex:762
CompName:3-Hexen-2-one $$ 1-Butenyl methyl ketone $$ Methyl 1-butenyl ketone $$ (3E)-3-Hexen-2-one # $$

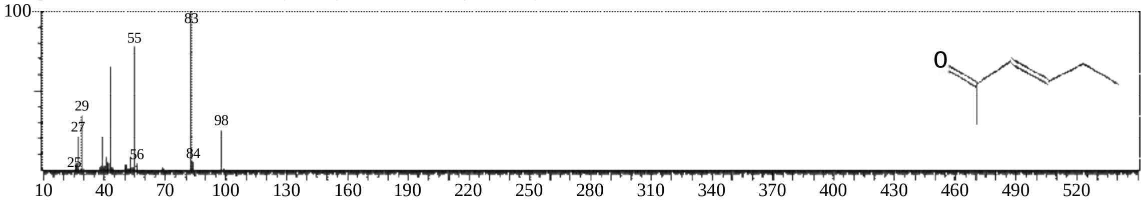

Hit#:3 Entry:1527 Library:NIST05s.LIB
SI:85 Formula:C6H10O CAS:141-79-7 MolWeight:98 RetIndex:739
CompName:3-Penten-2-one, 4-methyl- $$ Acetone, isopropylidene- $$ Isobutenyl methyl ketone $$ Isopropylideneacetone $$ Mesityl oxide $$ Methyl isobuten

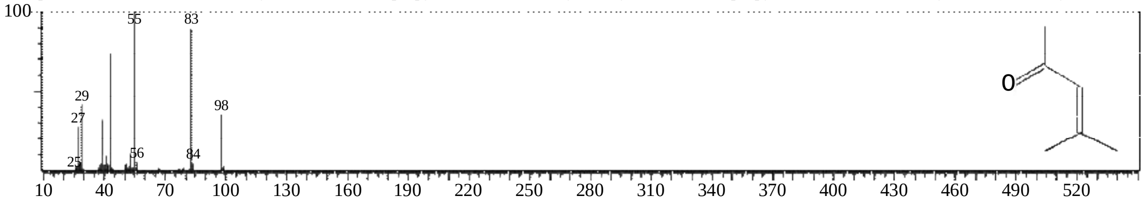

Hit#:4 Entry:17002 Library:NIST05.LIB
SI:85 Formula:C10H18O CAS:1462-27-7 MolWeight:154 RetIndex:1215
CompName:1-Butanone, 1-cyclohexyl- $$ 1-Cyclohexanol-1-butanone $$ 1-Cyclohexyl-1-butanone $$

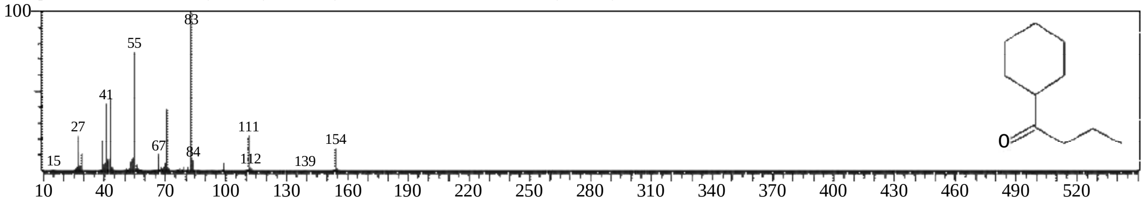

Hit#:5 Entry:1621 Library:NIST05s.LIB
SI:85 Formula:C7H14 CAS:625-65-0 MolWeight:98 RetIndex:638
CompName:2-Pentene, 2,4-dimethyl- $$ 2,4-Dimethyl-2-pentene $$ (CH3)2CHCH=C(CH3)2 $$

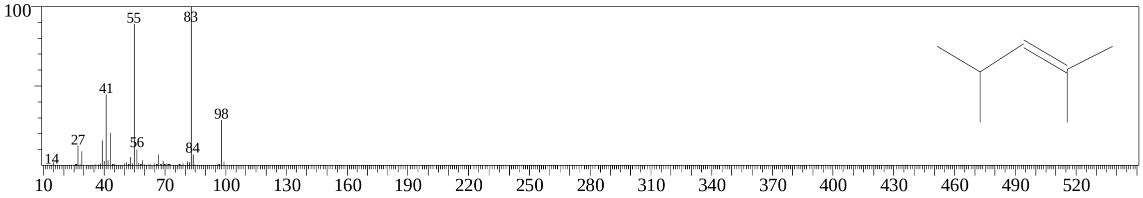

<< Target >>
Line#:8 R.Time:4.950(Scan#:235) MassPeaks:116
RawMode:Averaged 4.942-4.958(234-236) BasePeak:55.05(41033)
BG Mode:Calc. from Peak Group 1 - Event 1

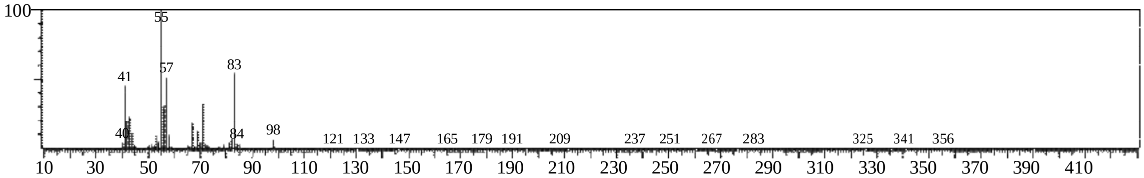

Hit#:1 Entry:1574 Library:NIST05s.LIB
SI:87 Formula:C7H14 CAS:74752-93-5 MolWeight:98 RetIndex:617
CompName:Cyclopropane, 1,1,2,3-tetramethyl- $$ 1,1,2,3-Tetramethylcyclopropane # $$

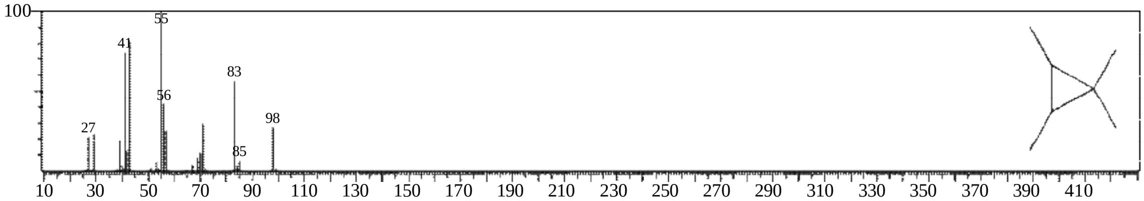

Hit#:2 Entry:3463 Library:NIST05.LIB
SI:86 Formula:C7H12O CAS:5204-80-8 MolWeight:112 RetIndex:831
CompName:4-Pentenal, 2-ethyl- $$ 2-Ethyl-4-pentenal # $$

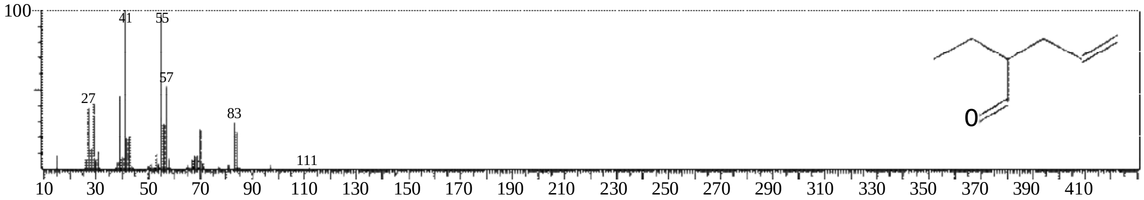

Hit#:3 Entry:718 Library:NIST05s.LIB
SI:86 Formula:C5H8O CAS:922-65-6 MolWeight:84 RetIndex:661
CompName:1,4-Pentadien-3-ol

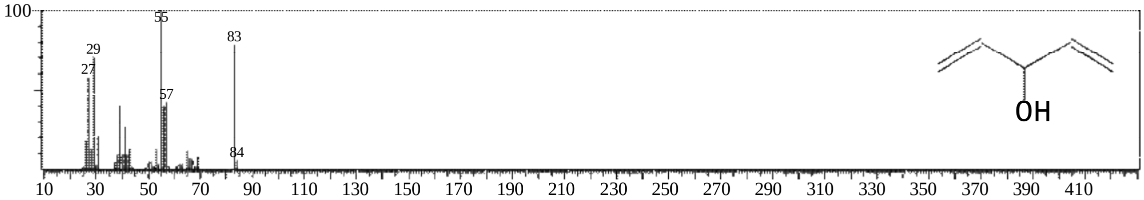

Hit#:4 Entry:2219 Library:NIST05.LIB
SI:85 Formula:C5H10O2 CAS:5057-99-8 MolWeight:102 RetIndex:976
CompName:1,2-Cyclopentanediol, trans- $$ trans-1,2-Cyclopentanediol $$ 1,2-Cyclopentanediol # $$

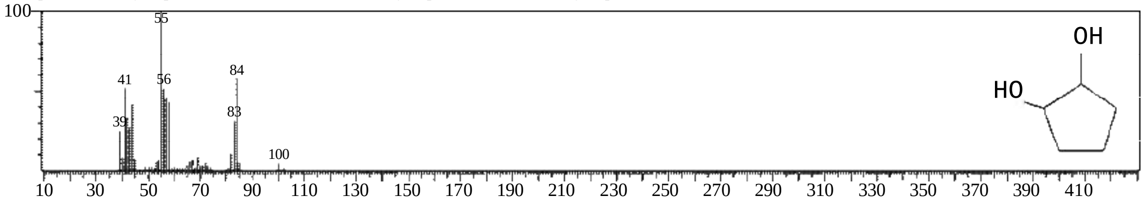

Hit#:5 Entry:4599 Library:NIST05s.LIB
SI:85 Formula:C8H14O CAS:286-62-4 MolWeight:126 RetIndex:970
CompName:9-Oxabicyclo[6.1.0]nonane $$ Cyclooctane, 1,2-epoxy- $$ Cyclooctene, oxide $$ 1,2-Epoxycyclooctane $$ Epoxycyclooctane $$

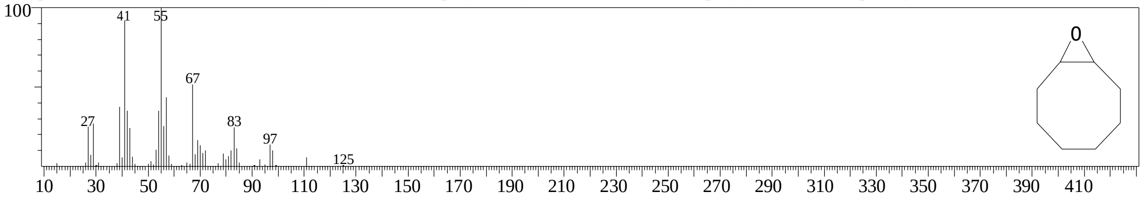

<< Target >>
Line#:9 R.Time:5.083(Scan#:251) MassPeaks:100
RawMode:Averaged 5.075-5.092(250-252) BasePeak:55.05(13968)
BG Mode:Calc. from Peak Group 1 - Event 1

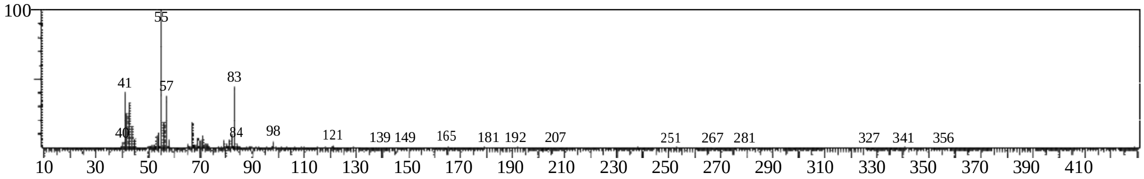

Hit#:1 Entry:4599 Library:NIST05s.LIB
SI:87 Formula:C8H14O CAS:286-62-4 MolWeight:126 RetIndex:970
CompName:9-Oxabicyclo[6.1.0]nonane $$ Cyclooctane, 1,2-epoxy- $$ Cyclooctene, oxide $$ 1,2-Epoxycyclooctane $$ Epoxycyclooctane $$

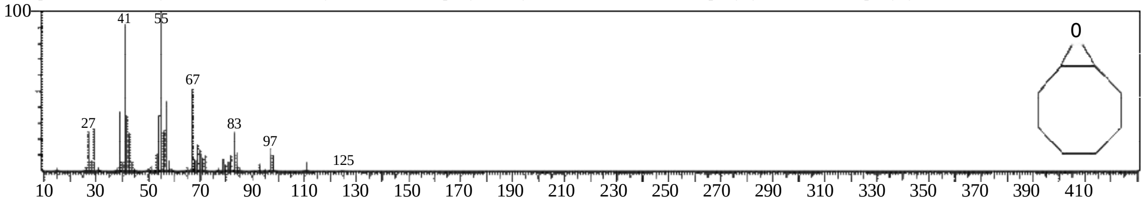

Hit#:2 Entry:6400 Library:NIST05.LIB
SI:86 Formula:C8H14O CAS:4925-71-7 MolWeight:126 RetIndex:970
CompName:9-Oxabicyclo[6.1.0]nonane, cis- $$ 9-Oxabicyclo[6.1.0]nonane # $$

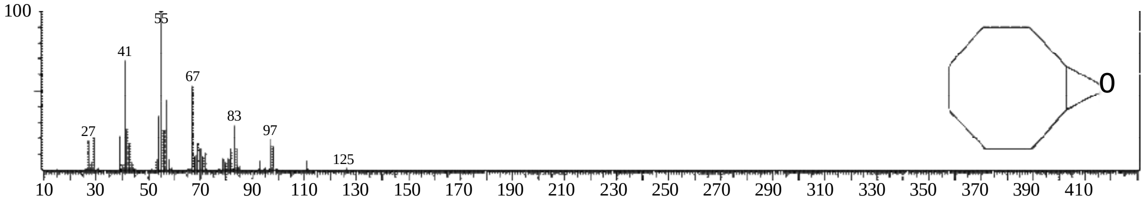

Hit#:3 Entry:68494 Library:NIST05.LIB
SI:85 Formula:C12H22O3S CAS:6214-17-1 MolWeight:246 RetIndex:1964
CompName:Sulfurous acid, dicyclohexyl ester $$ Dicyclohexyl sulfite # $$

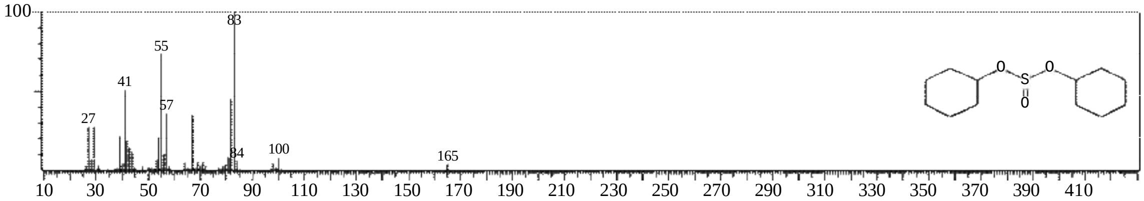

Hit#:4 Entry:1517 Library:NIST05s.LIB
SI:85 Formula:C6H10O CAS:105-31-7 MolWeight:98 RetIndex:778
CompName:1-Hexyn-3-ol $$ Propylethinylcarbinol $$ 1-Hexyne-3-ol $$ 1-Propylpropargyl alcohol $$ 3-Hydroxy-1-hexyne $$ Hexynol $$

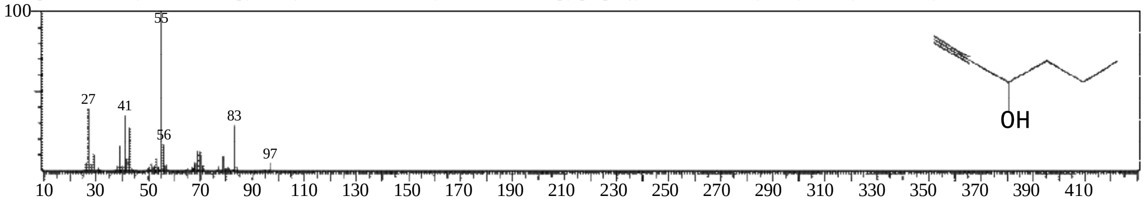

Hit#:5 Entry:12920 Library:NIST05.LIB
SI:85 Formula:C7H15NO2 CAS:693-39-0 MolWeight:145 RetIndex:1099
CompName:Heptane, 1-nitro- $$ 1-Nitroheptane $$

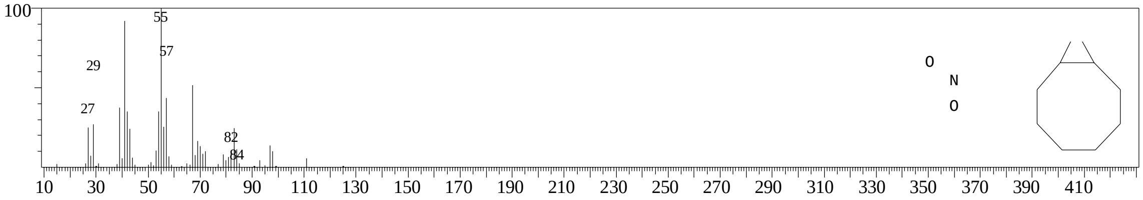

<< Target >>
Line#:10 R.Time:5.658(Scan#:320) MassPeaks:96
RawMode:Averaged 5.650-5.667(319-321) BasePeak:55.10(15920)
BG Mode:Calc. from Peak Group 1 - Event 1

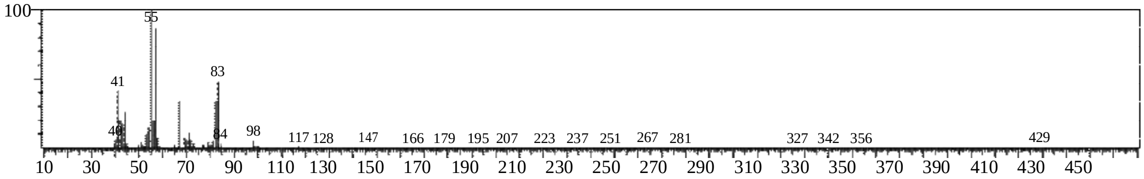

Hit#:1 Entry:7386 Library:NIST05.LIB
SI:87 Formula:C6H11NO2 CAS:5156-40-1 MolWeight:129 RetIndex:871
CompName:Nitrous acid, cyclohexyl ester $$ Cyclohexyl nitrite $$ Cyclohexyl ester of nitrous acid $$

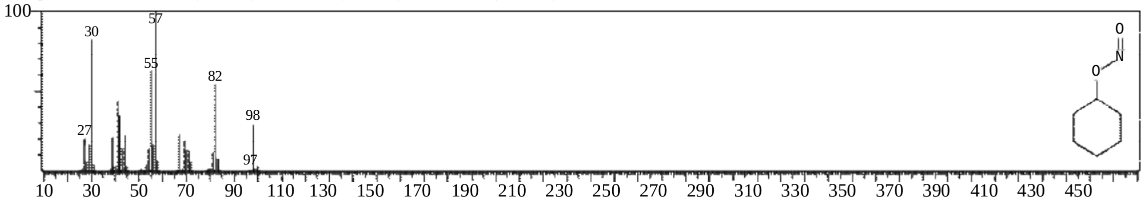

Hit#:2 Entry:68494 Library:NIST05.LIB
SI:86 Formula:C12H22O3S CAS:6214-17-1 MolWeight:246 RetIndex:1964
CompName:Sulfurous acid, dicyclohexyl ester $$ Dicyclohexyl sulfite # $$

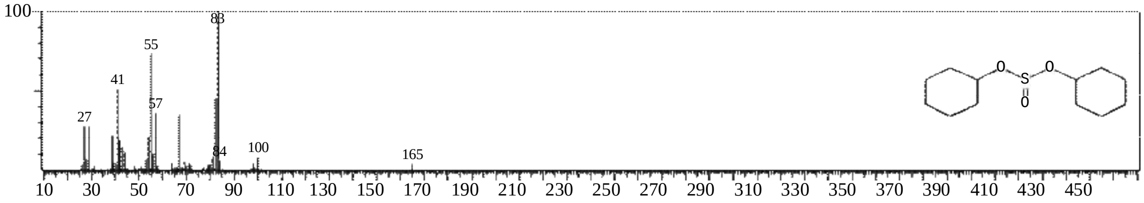

Hit#:3 Entry:1994 Library:NIST05.LIB
SI:86 Formula:C6H12O CAS:928-94-9 MolWeight:100 RetIndex:868
CompName:2-Hexen-1-ol, (Z)- $$ cis-2-Hexen-1-ol $$ (2Z)-2-Hexen-1-ol # $$

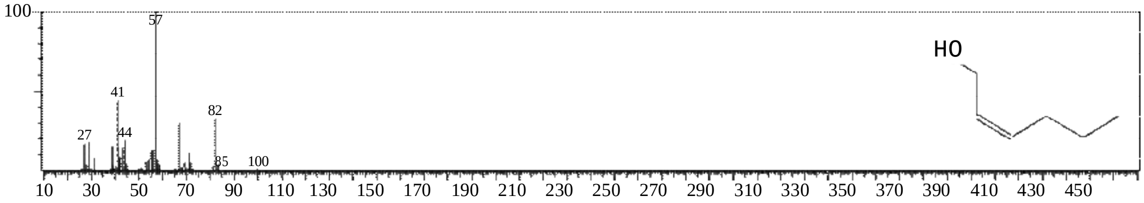

Hit#:4 Entry:1806 Library:NIST05s.LIB
SI:85 Formula:C6H12O CAS:928-94-9 MolWeight:100 RetIndex:868
CompName:2-Hexen-1-ol, (Z)- $$ cis-2-Hexen-1-ol $$ (2Z)-2-Hexen-1-ol # $$

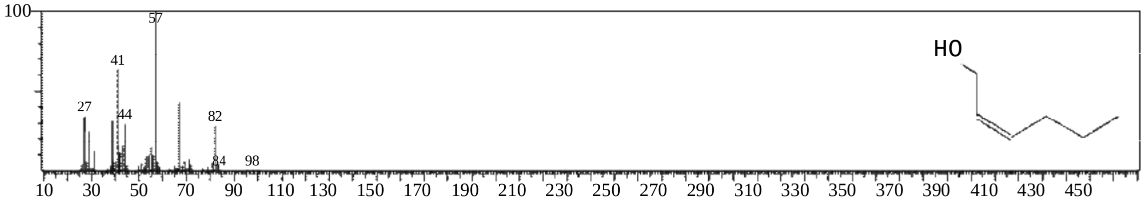

Hit#:5 Entry:1807 Library:NIST05s.LIB
SI:85 Formula:C6H12O CAS:928-94-9 MolWeight:100 RetIndex:868
CompName:2-Hexen-1-ol, (Z)- $$ cis-2-Hexen-1-ol $$ (2Z)-2-Hexen-1-ol # $$

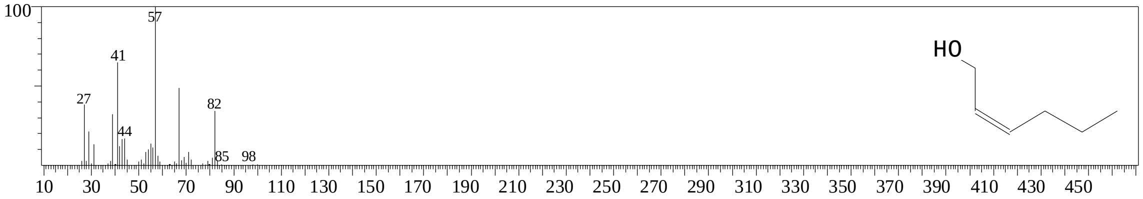

<< Target >>
Line#:11 R.Time:6.483(Scan#:419) MassPeaks:68
RawMode:Averaged 6.475-6.492(418-420) BasePeak:57.10(3478)
BG Mode:Calc. from Peak Group 1 - Event 1

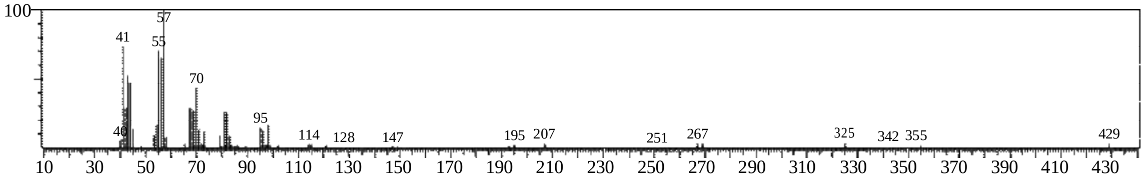

Hit#:1 Entry:7220 Library:NIST05s.LIB
SI:91 Formula:C9H18O CAS:124-19-6 MolWeight:142 RetIndex:1104
CompName:Nonanal $$ n-Nonaldehyde $$ n-Nonanal $$ n-Nonylaldehyde $$ Nonaldehyde $$ Nonanaldehyde $$ Nonanoic aldehyde $$ Nonylaldehyde $$ No

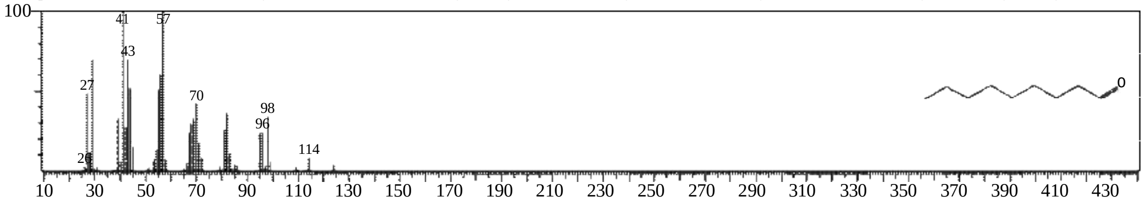

Hit#:2 Entry:11935 Library:NIST05.LIB
SI:91 Formula:C9H18O CAS:124-19-6 MolWeight:142 RetIndex:1104
CompName:Nonanal $$ n-Nonaldehyde $$ n-Nonanal $$ n-Nonylaldehyde $$ Nonaldehyde $$ Nonanaldehyde $$ Nonanoic aldehyde $$ Nonylaldehyde $$ No

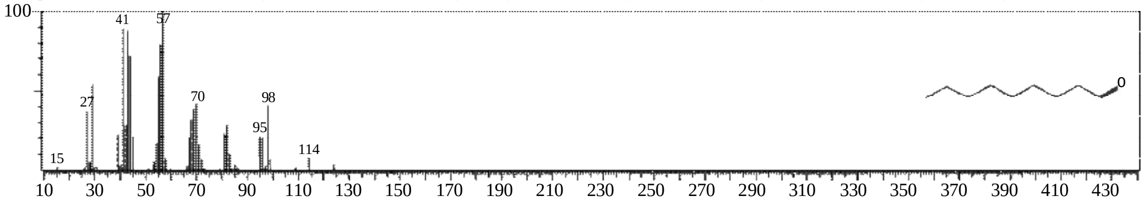

Hit#:3 Entry:7237 Library:NIST05s.LIB
SI:91 Formula:C9H18O CAS:124-19-6 MolWeight:142 RetIndex:1104
CompName:Nonanal $$ n-Nonaldehyde $$ n-Nonanal $$ n-Nonylaldehyde $$ Nonaldehyde $$ Nonanaldehyde $$ Nonanoic aldehyde $$ Nonylaldehyde $$ No

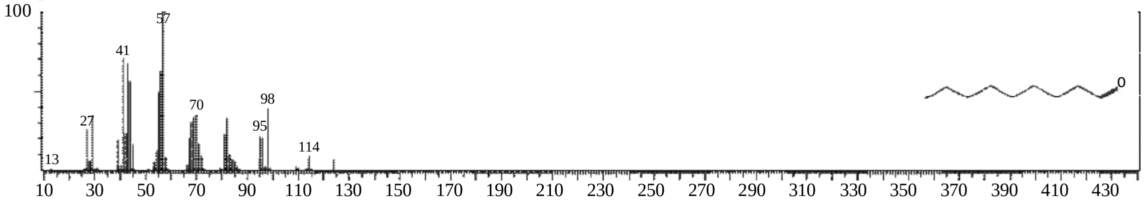

Hit#:4 Entry:7238 Library:NIST05s.LIB
SI:87 Formula:C9H18O CAS:31502-14-4 MolWeight:142 RetIndex:1167
CompName:2-Nonen-1-ol, (E)- $$ (E)-2-Nonen-1-ol $$ trans-2-Nonen-1-Ol $$ trans-2-Nonenol $$ (2E)-2-Nonen-1-ol # $$

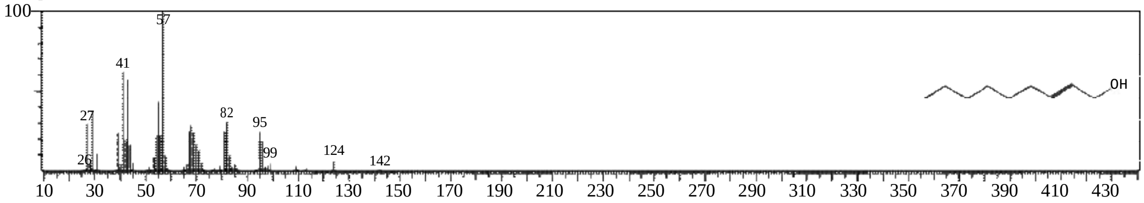

Hit#:5 Entry:7239 Library:NIST05s.LIB
SI:87 Formula:C9H18O CAS:31502-14-4 MolWeight:142 RetIndex:1167
CompName:2-Nonen-1-ol, (E)- $$ (E)-2-Nonen-1-ol $$ trans-2-Nonen-1-Ol $$ trans-2-Nonenol $$ (2E)-2-Nonen-1-ol # $$

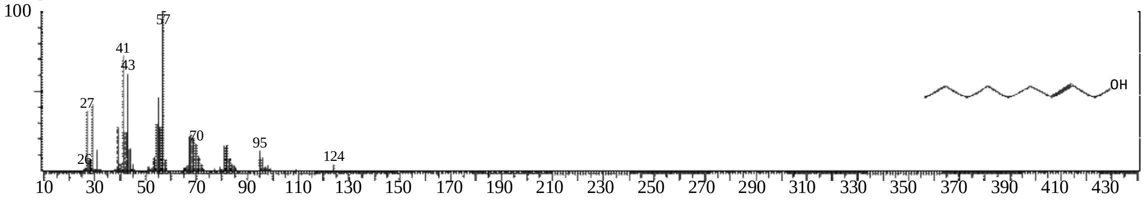

<< Target >>
Line#:12 R.Time:7.408(Scan#:530) MassPeaks:192
RawMode:Averaged 7.400-7.417(529-531) BasePeak:60.05(396193)
BG Mode:Calc. from Peak Group 1 - Event 1

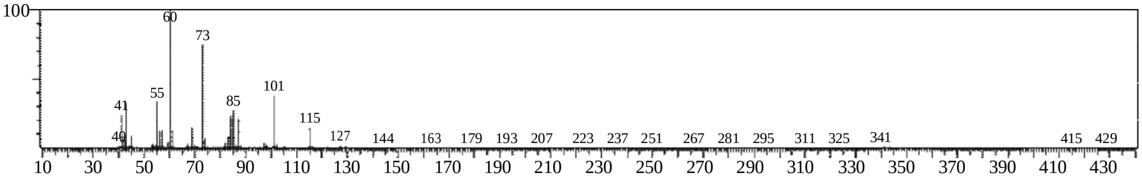

Hit#:1 Entry:7496 Library:NIST05s.LIB
SI:94 Formula:C8H16O2 CAS:124-07-2 MolWeight:144 RetIndex:1173
CompName:Octanoic Acid $$ n-Caprylic acid $$ n-Octanoic acid $$ n-Octoic acid $$ n-Octylic acid $$ Neo-Fat 8 $$ Caprylic acid $$ Enantic acid $$ Octylic a

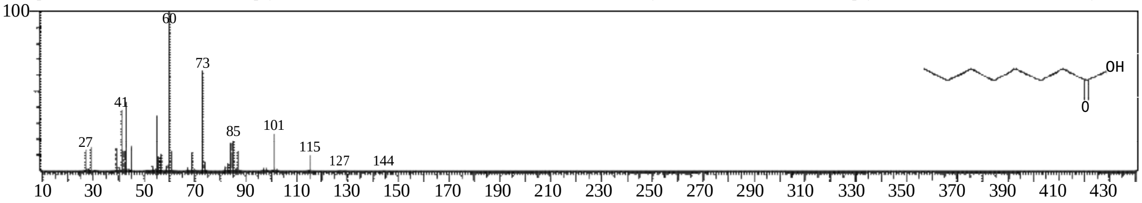

Hit#:2 Entry:12611 Library:NIST05.LIB
SI:93 Formula:C8H16O2 CAS:124-07-2 MolWeight:144 RetIndex:1173
CompName:Octanoic Acid $$ n-Caprylic acid $$ n-Octanoic acid $$ n-Octoic acid $$ n-Octylic acid $$ Neo-Fat 8 $$ Caprylic acid $$ Enantic acid $$ Octylic a

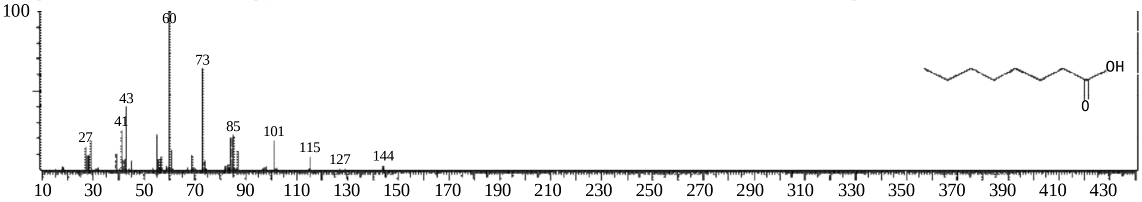

Hit#:3 Entry:7495 Library:NIST05s.LIB
SI:90 Formula:C8H16O2 CAS:124-07-2 MolWeight:144 RetIndex:1173
CompName:Octanoic Acid $$ n-Caprylic acid $$ n-Octanoic acid $$ n-Octoic acid $$ n-Octylic acid $$ Neo-Fat 8 $$ Caprylic acid $$ Enantic acid $$ Octylic a

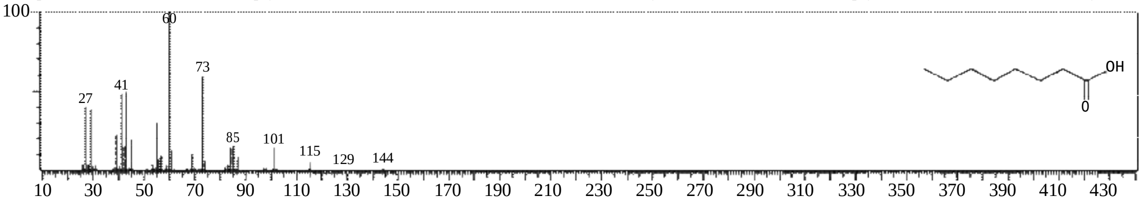

Hit#:4 Entry:18733 Library:NIST05.LIB
SI:87 Formula:C9H18O2 CAS:112-05-0 MolWeight:158 RetIndex:1272
CompName:Nonanoic acid $$ n-Nonanoic acid $$ n-Nonoic acid $$ n-Nonylic acid $$ Nonoic acid $$ Nonylic acid $$ Pelargic acid $$ Pelargonic acid $$ 1-Oc

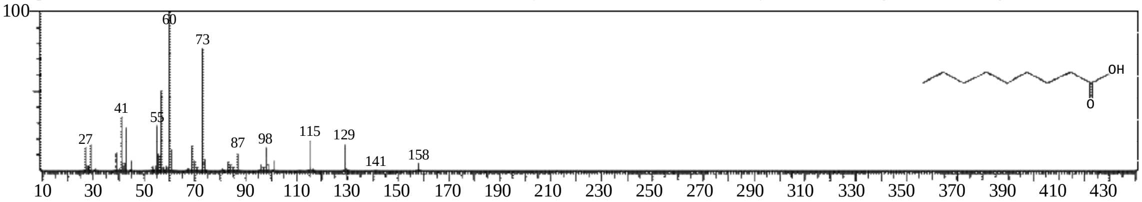

Hit#:5 Entry:5293 Library:NIST05s.LIB
SI:86 Formula:C7H14O2 CAS:111-14-8 MolWeight:130 RetIndex:1073
CompName:Heptanoic acid $$ Enanthic acid $$ n-Heptanoic acid $$ n-Heptoic acid $$ n-Heptylic acid $$ Enanthylic acid $$ Heptoic acid $$ Heptylic acid $$ O

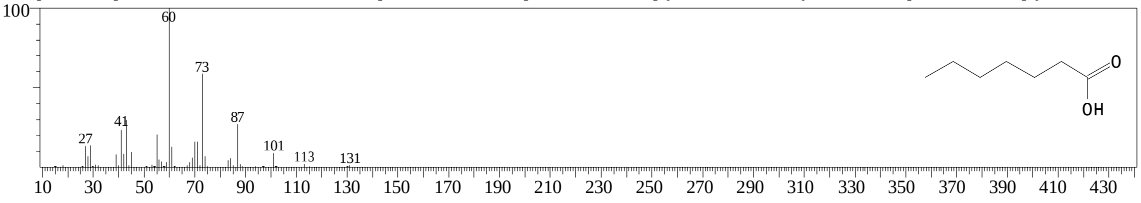

<< Target >>
Line#:13 R.Time:7.808(Scan#:578) MassPeaks:130
RawMode:Averaged 7.800-7.817(577-579) BasePeak:128.10(9805)
BG Mode:Calc. from Peak Group 1 - Event 1

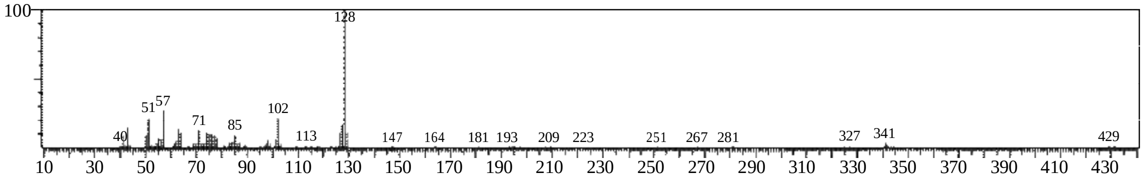

Hit#:1 Entry:5053 Library:NIST05s.LIB
SI:79 Formula:C10H8 CAS:275-51-4 MolWeight:128 RetIndex:1069
CompName:Azulene $$ Bicyclo[5.3.0]decapentaene $$ Cyclopentacycloheptene $$ Azunamic $$ Bicyclo(5.3.0)-1,3,5,7,9-decapentaene $$ Bicyclo(0.3.5)deca-1

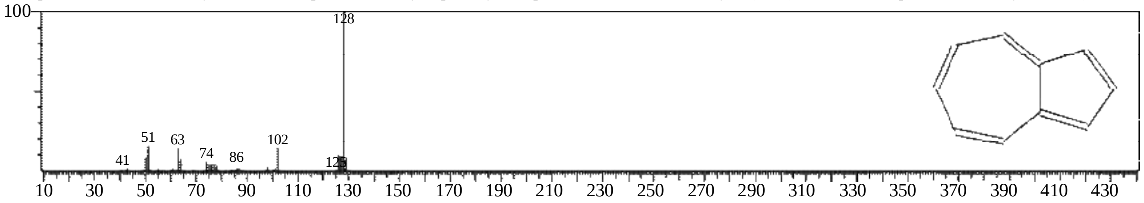

Hit#:2 Entry:5054 Library:NIST05s.LIB
SI:78 Formula:C10H8 CAS:275-51-4 MolWeight:128 RetIndex:1069
CompName:Azulene $$ Bicyclo[5.3.0]decapentaene $$ Cyclopentacycloheptene $$ Azunamic $$ Bicyclo(5.3.0)-1,3,5,7,9-decapentaene $$ Bicyclo(0.3.5)deca-1

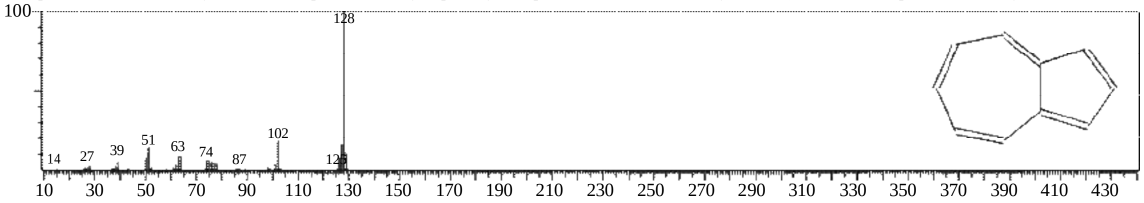

Hit#:3 Entry:5052 Library:NIST05s.LIB
SI:77 Formula:C10H8 CAS:275-51-4 MolWeight:128 RetIndex:1069
CompName:Azulene $$ Bicyclo[5.3.0]decapentaene $$ Cyclopentacycloheptene $$ Azunamic $$ Bicyclo(5.3.0)-1,3,5,7,9-decapentaene $$ Bicyclo(0.3.5)deca-1

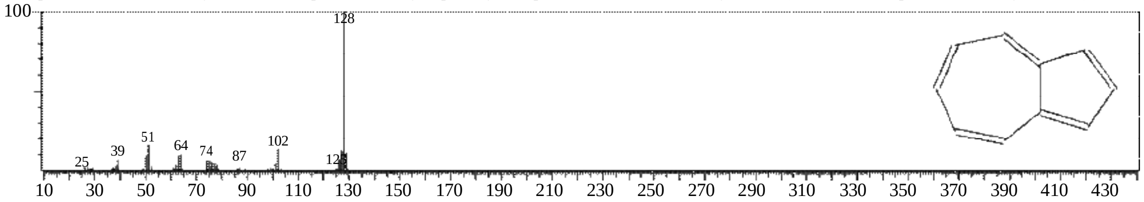

Hit#:4 Entry:7298 Library:NIST05.LIB
SI:76 Formula:C10H8 CAS:88090-34-0 MolWeight:128 RetIndex:957
CompName:[4.2.2]Propella-2,4,7,9-tetraene

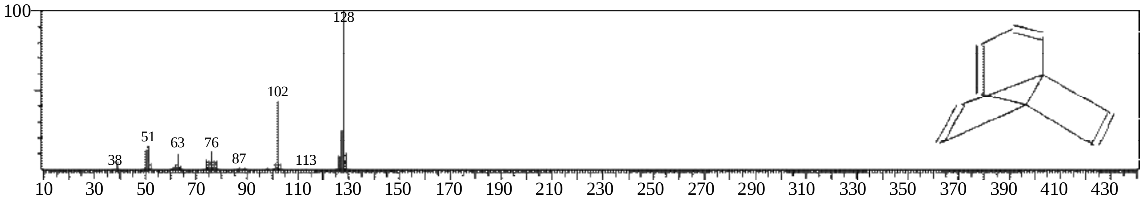

Hit#:5 Entry:7296 Library:NIST05.LIB
SI:76 Formula:C10H8 CAS:0-00-0 MolWeight:128 RetIndex:1098
CompName:4-Phenylbut-3-ene-1-yne

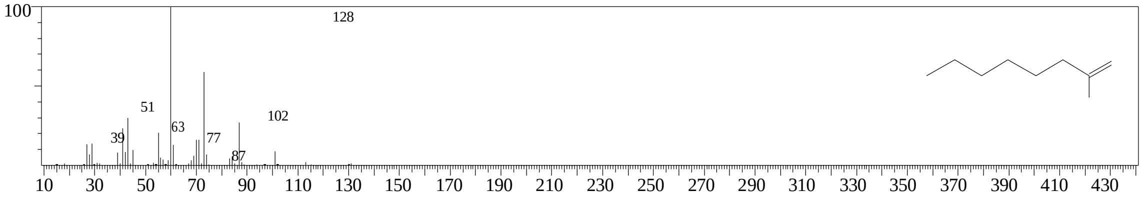

<< Target >>
Line#:14 R.Time:8.533(Scan#:665) MassPeaks:97
RawMode:Averaged 8.525-8.542(664-666) BasePeak:83.10(2643)
BG Mode:Calc. from Peak Group 1 - Event 1

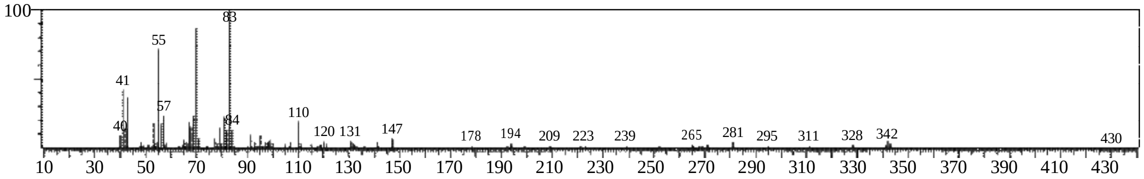

Hit#:1 Entry:16999 Library:NIST05.LIB
SI:82 Formula:C10H18O CAS:0-00-0 MolWeight:154 RetIndex:1233
CompName:2,4-Pentadien-1-ol, 3-pentyl-, (2Z)-

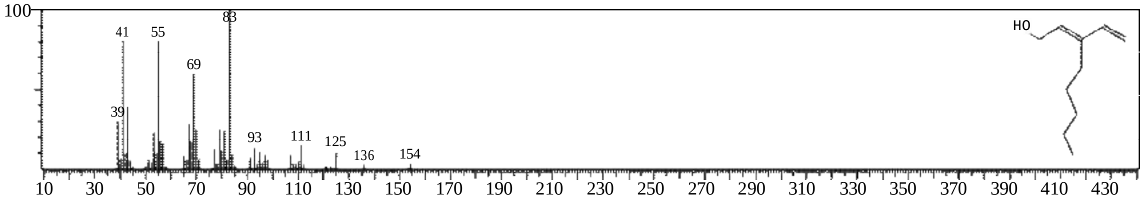

Hit#:2 Entry:31237 Library:NIST05.LIB
SI:81 Formula:C12H22O CAS:20407-84-5 MolWeight:182 RetIndex:1410
CompName:2-Dodecenal, (E)- $$ (E)-2-Dodecen-1-al $$ (2E)-2-Dodecenal # $$

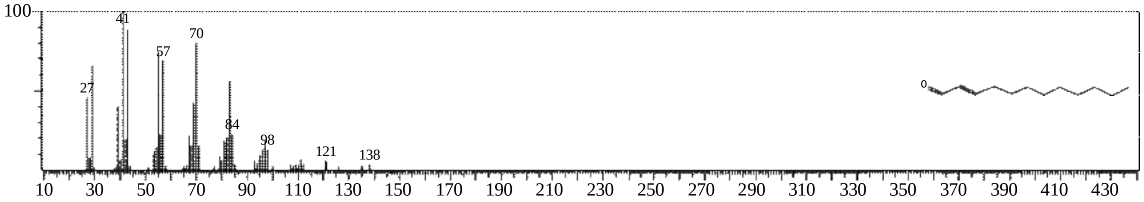

Hit#:3 Entry:4596 Library:NIST05s.LIB
SI:81 Formula:C8H14O CAS:2548-87-0 MolWeight:126 RetIndex:1013
CompName:2-Octenal, (E)- $$ (E)-2-Octen-1-al $$ (E)-2-Octenal $$ trans-2-Octenal $$ trans-2-Octen-1-al $$ (2E)-2-Octenal # $$

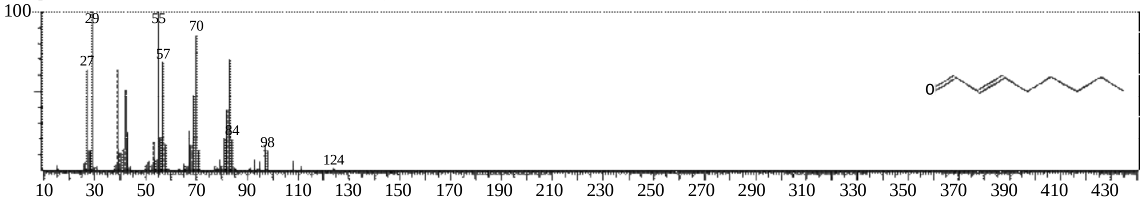

Hit#:4 Entry:9350 Library:NIST05s.LIB
SI:80 Formula:C10H18O CAS:27779-29-9 MolWeight:154 RetIndex:1125
CompName:Bicyclo[3.1.1]heptan-3-ol, 2,6,6-trimethyl-, (1.alpha.,2.beta.,3.alpha.,5.alpha.)- $$ 3-Pinanol, stereoisomer $$ Isopinocampheol $$ (1S,2S,3S,5R)-(

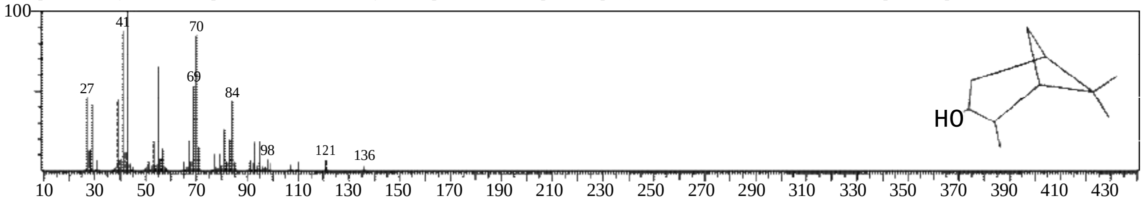

Hit#:5 Entry:16843 Library:NIST05.LIB
SI:80 Formula:C10H18O CAS:2497-25-8 MolWeight:154 RetIndex:1212
CompName:2-Decenal, (Z)- $$ (Z)-2-Decenal $$ (2Z)-2-Decenal # $$

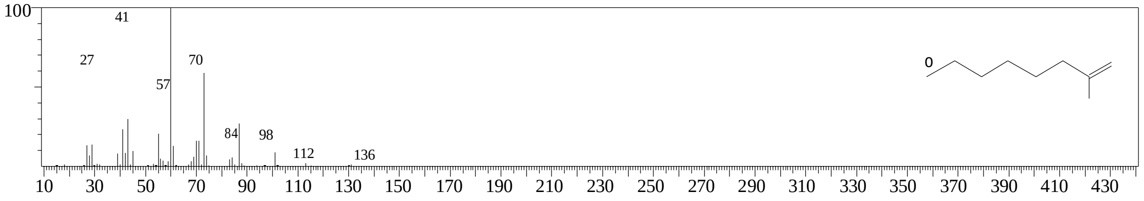

Analytical Research & Metallurgical Laboratories Pvt. Ltd.

<< Target >>
Line#:15 R.Time:8.733(Scan#:689) MassPeaks:143
RawMode:Averaged 8.725-8.742(688-690) BasePeak:55.10(20792)
BG Mode:Calc. from Peak Group 1 - Event 1

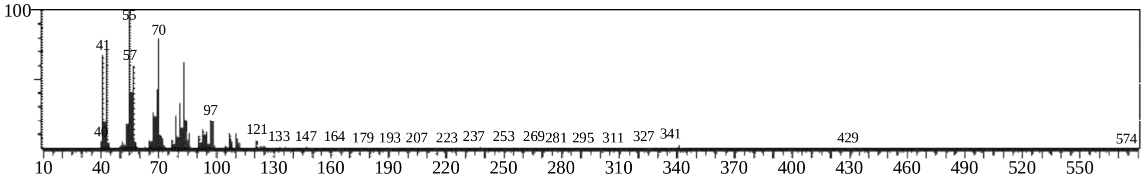

Hit#:1 Entry:39015 Library:NIST05.LIB
SI:90 Formula:C13H24O CAS:7069-41-2 MolWeight:196 RetIndex:1510
CompName:2-Tridecenal, (E)- $$ (2E)-2-Tridecenal # $$

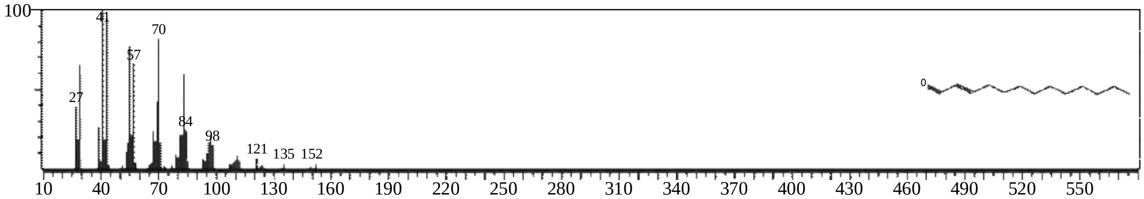

Hit#:2 Entry:10976 Library:NIST05.LIB
SI:90 Formula:C9H16O CAS:18829-56-6 MolWeight:140 RetIndex:1112
CompName:2-Nonenal, (E)- $$ (E)-2-Nonenal $$ trans-2-Nonen-1-al $$ trans-2-Nonenal $$ (2E)-2-Nonenal # $$

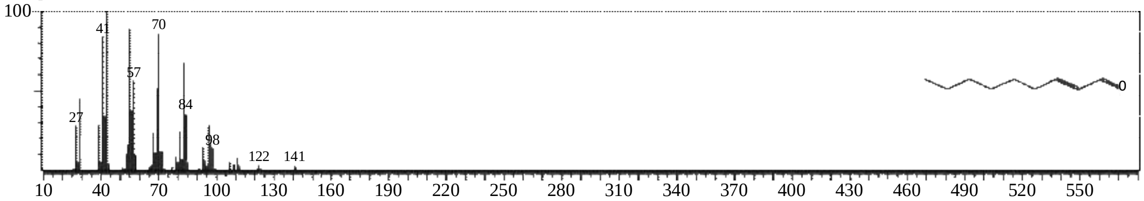

Hit#:3 Entry:31237 Library:NIST05.LIB
SI:90 Formula:C12H22O CAS:20407-84-5 MolWeight:182 RetIndex:1410
CompName:2-Dodecenal, (E)- $$ (E)-2-Dodecen-1-al $$ (2E)-2-Dodecenal # $$

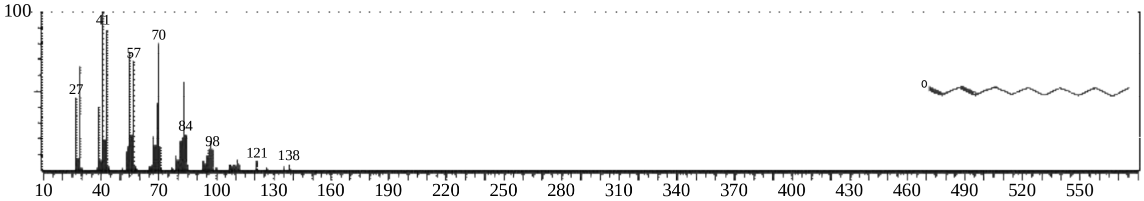

Hit#:4 Entry:16866 Library:NIST05.LIB
SI:90 Formula:C10H18O CAS:3913-81-3 MolWeight:154 RetIndex:1212
CompName:2-Decenal, (E)- $$ trans-2-Decenal $$ (E)-2-Decenol $$ (2E)-2-Decenal # $$

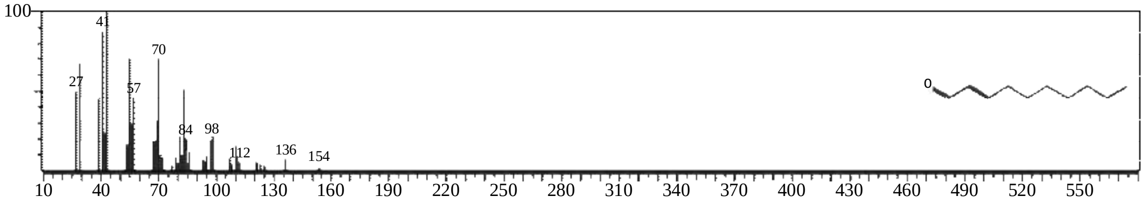

Hit#:5 Entry:23754 Library:NIST05.LIB
SI:89 Formula:C11H20O CAS:2463-77-6 MolWeight:168 RetIndex:1311
CompName:2-Undecenal $$ Undec-2-enal $$ 2-Undecen-1-al $$ trans-2-Undecen-1-al $$ (2E)-2-Undecenal # $$

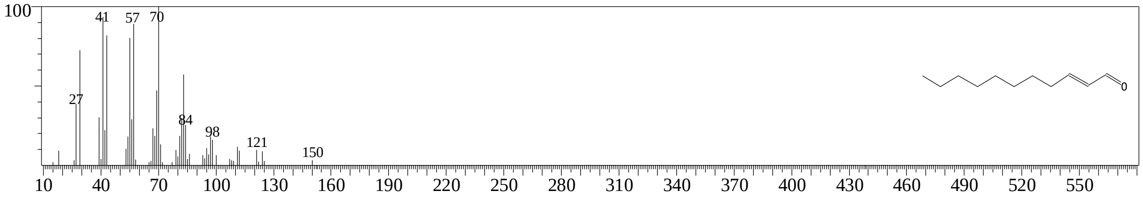

<< Target >>
Line#:16 R.Time:9.158(Scan#:740) MassPeaks:141
RawMode:Averaged 9.150-9.167(739-741) BasePeak:99.05(19419)
BG Mode:Calc. from Peak Group 1 - Event 1

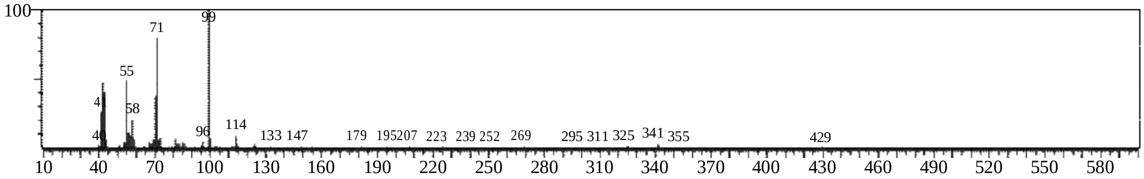

Hit#:1 Entry:9696 Library:NIST05s.LIB
SI:91 Formula:C9H16O2 CAS:3301-94-8 MolWeight:156 RetIndex:1304
CompName:.delta. Nonalactone $$ 2H-Pyran-2-one, 6-butyltetrahydro- $$ 2H-Pyran-2-one, tetrahydro-6-butyl $$ .beta.-Nonalactone $$ 6-Butyltetrahydro-2H-p

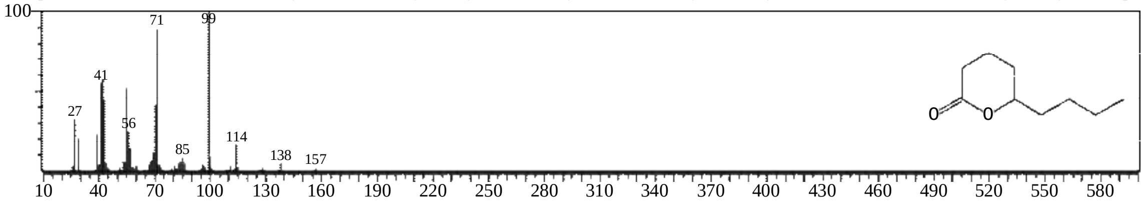

Hit#:2 Entry:7205 Library:NIST05s.LIB
SI:91 Formula:C8H14O2 CAS:698-76-0 MolWeight:142 RetIndex:1205
CompName:2H-Pyran-2-one, tetrahydro-6-propyl- $$ .delta.-Octalactone $$ .delta.-Propylvalerolactone $$ Octanoic acid, 5-hydroxy-, .delta.-lactone $$ 5-Hydro

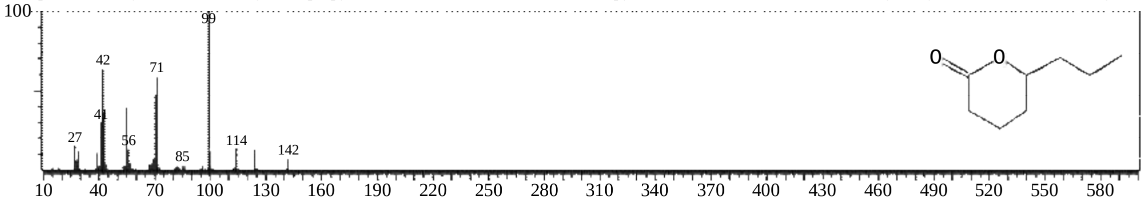

Hit#:3 Entry:7206 Library:NIST05s.LIB
SI:90 Formula:C8H14O2 CAS:698-76-0 MolWeight:142 RetIndex:1205
CompName:2H-Pyran-2-one, tetrahydro-6-propyl- $$ .delta.-Octalactone $$ .delta.-Propylvalerolactone $$ Octanoic acid, 5-hydroxy-, .delta.-lactone $$ 5-Hydro

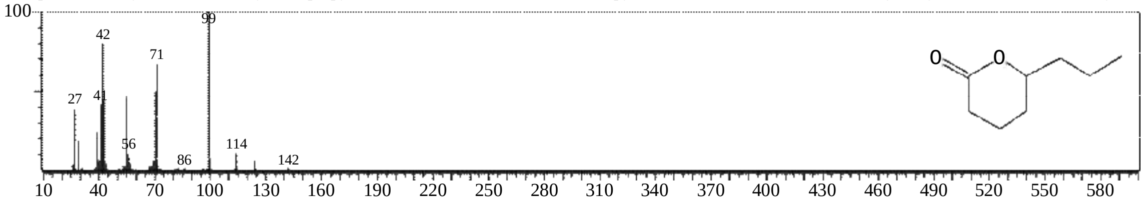

Hit#:4 Entry:11958 Library:NIST05s.LIB
SI:89 Formula:C10H18O2 CAS:705-86-2 MolWeight:170 RetIndex:1404
CompName:2H-Pyran-2-one, tetrahydro-6-pentyl- $$ .delta.-Amylvalerolactone $$ .delta.-Decalactone $$ Decanoic acid, 5-hydroxy-, .delta.-lactone $$ 5-Decan

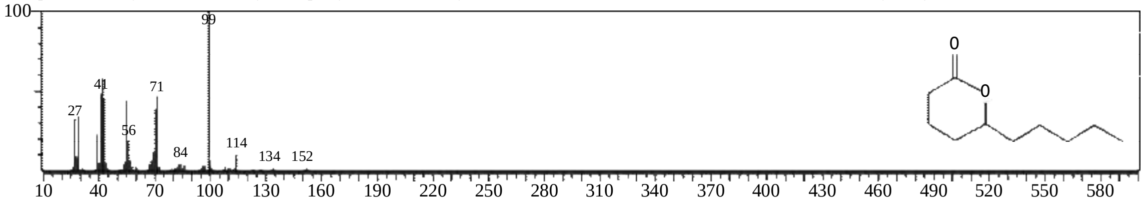

Hit#:5 Entry:17870 Library:NIST05.LIB
SI:88 Formula:C9H16O2 CAS:3301-94-8 MolWeight:156 RetIndex:1304
CompName:.delta. Nonalactone $$ 2H-Pyran-2-one, 6-butyltetrahydro- $$ 2H-Pyran-2-one, tetrahydro-6-butyl $$ .beta.-Nonalactone $$ 6-Butyltetrahydro-2H-p

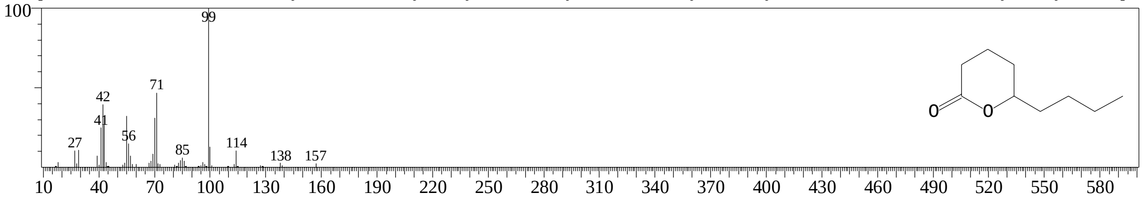

<< Target >>
Line#:17 R.Time:9.500(Scan#:781) MassPeaks:67
RawMode:Averaged 9.492-9.508(780-782) BasePeak:81.05(7030)
BG Mode:Calc. from Peak Group 1 - Event 1

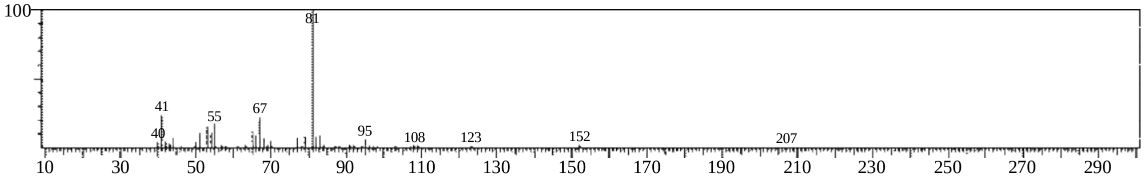

Hit#:1 Entry:15851 Library:NIST05.LIB
SI:91 Formula:C10H16O CAS:25152-84-5 MolWeight:152 RetIndex:1220
CompName:2,4-Decadienal, (E,E)- $$ (E,E)-2,4-Decadienal $$ trans-2, trans-4-Decadienal $$ trans,trans-2,4-Decadien-1-al $$ trans,trans-2,4-Decadienal $$ 2-t

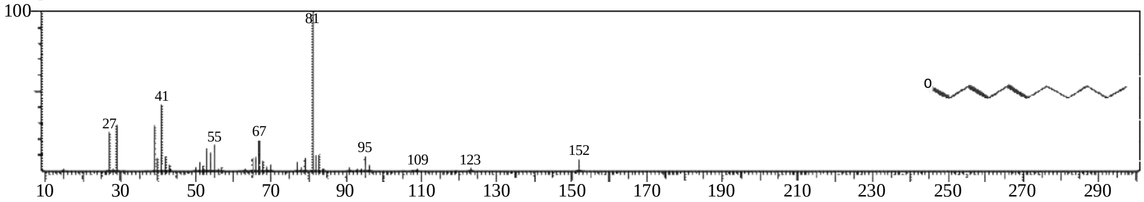

Hit#:2 Entry:8957 Library:NIST05s.LIB
SI:89 Formula:C10H16O CAS:25152-84-5 MolWeight:152 RetIndex:1220
CompName:2,4-Decadienal, (E,E)- $$ (E,E)-2,4-Decadienal $$ trans-2, trans-4-Decadienal $$ trans,trans-2,4-Decadien-1-al $$ trans,trans-2,4-Decadienal $$ 2-t

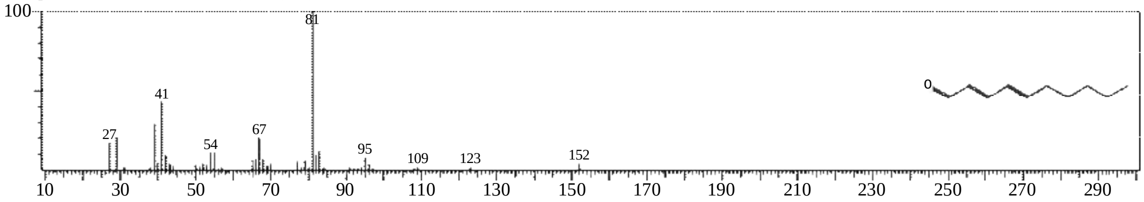

Hit#:3 Entry:15852 Library:NIST05.LIB
SI:89 Formula:C10H16O CAS:2363-88-4 MolWeight:152 RetIndex:1220
CompName:2,4-Decadienal $$ (2E,4E)-2,4-Decadienal # $$

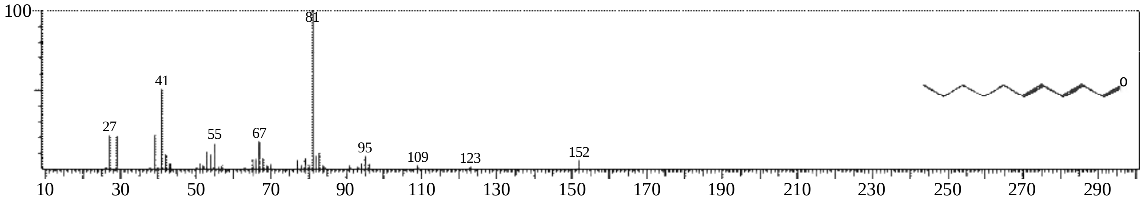

Hit#:4 Entry:10178 Library:NIST05.LIB
SI:87 Formula:C9H14O CAS:5910-87-2 MolWeight:138 RetIndex:1120
CompName:2,4-Nonadienal, (E,E)- $$ 2,4-trans,trans-Nonadienal $$ 2,4-Nonadien-1-al $$ (2E,4E)-2,4-Nonadienal # $$

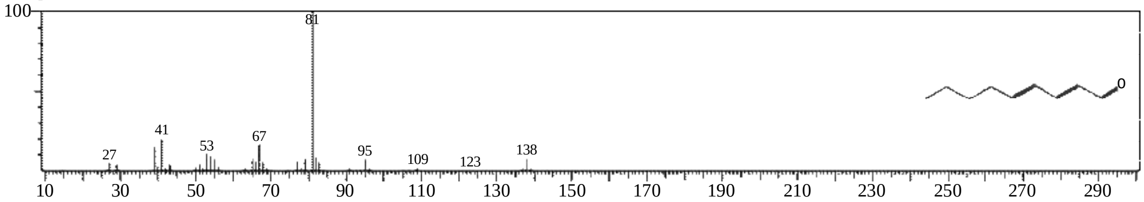

Hit#:5 Entry:10177 Library:NIST05.LIB
SI:87 Formula:C9H14O CAS:6750-03-4 MolWeight:138 RetIndex:1120
CompName:2,4-Nonadienal $$ n-Nona-2,4-dienal $$ (2E,4E)-2,4-Nonadienal # $$

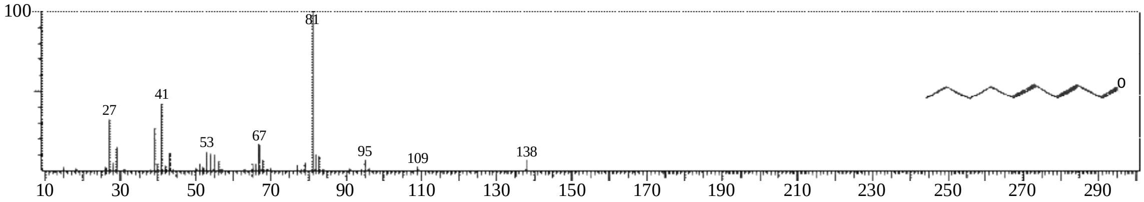

<< Target >>
Line#:18 R.Time:10.050(Scan#:847) MassPeaks:161
RawMode:Averaged 10.042-10.058(846-848) BasePeak:60.05(144785)
BG Mode:Calc. from Peak Group 1 - Event 1

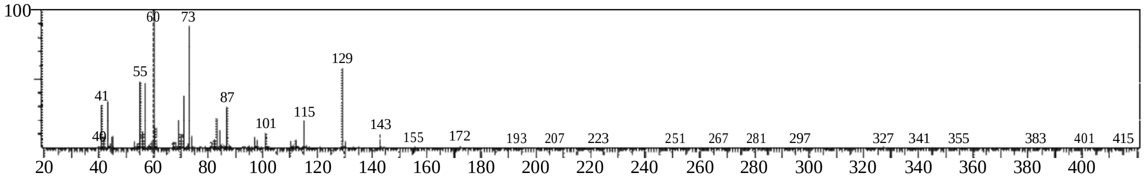

Hit#:1 Entry:12250 Library:NIST05s.LIB
SI:95 Formula:C10H20O2 CAS:334-48-5 MolWeight:172 RetIndex:1372
CompName:n-Decanoic acid $$ Decanoic acid $$ n-Capric acid $$ n-Decoic acid $$ n-Decylic acid $$ Capric acid $$ Caprinic acid $$ Caprynic acid $$ Decoic

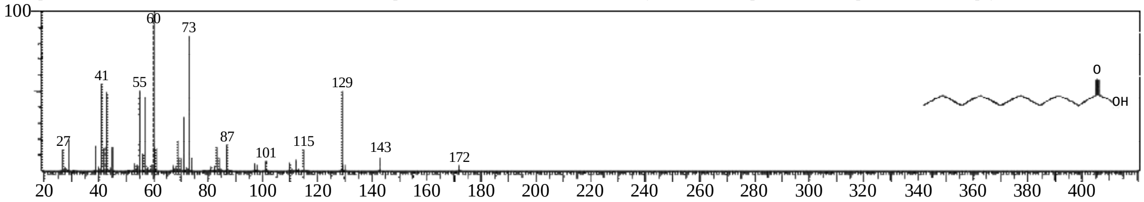

Hit#:2 Entry:12247 Library:NIST05s.LIB
SI:93 Formula:C10H20O2 CAS:334-48-5 MolWeight:172 RetIndex:1372
CompName:n-Decanoic acid $$ Decanoic acid $$ n-Capric acid $$ n-Decoic acid $$ n-Decylic acid $$ Capric acid $$ Caprinic acid $$ Caprynic acid $$ Decoic

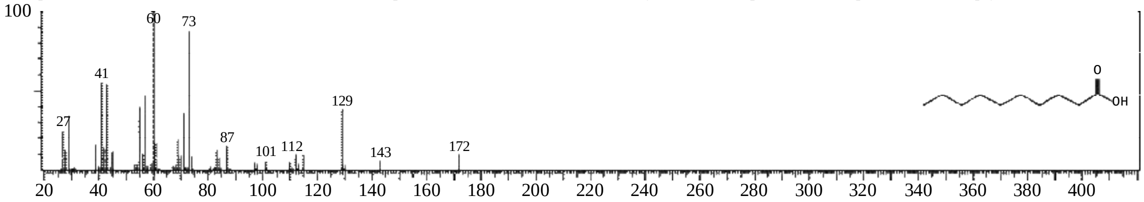

Hit#:3 Entry:25872 Library:NIST05.LIB
SI:93 Formula:C10H20O2 CAS:334-48-5 MolWeight:172 RetIndex:1372
CompName:n-Decanoic acid $$ Decanoic acid $$ n-Capric acid $$ n-Decoic acid $$ n-Decylic acid $$ Capric acid $$ Caprinic acid $$ Caprynic acid $$ Decoic

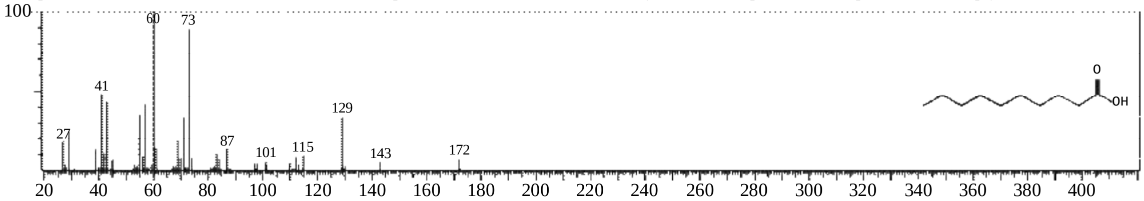

Hit#:4 Entry:12249 Library:NIST05s.LIB
SI:92 Formula:C10H20O2 CAS:334-48-5 MolWeight:172 RetIndex:1372
CompName:n-Decanoic acid $$ Decanoic acid $$ n-Capric acid $$ n-Decoic acid $$ n-Decylic acid $$ Capric acid $$ Caprinic acid $$ Caprynic acid $$ Decoic

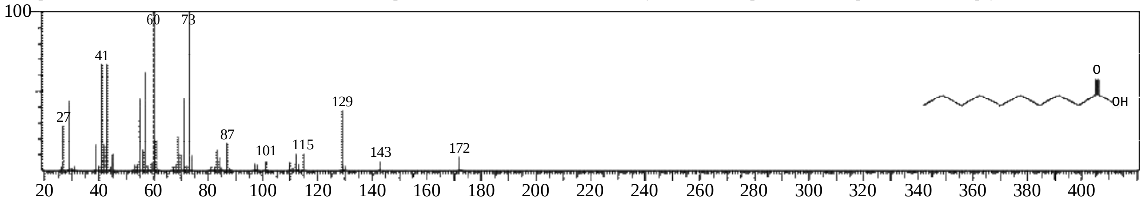

Hit#:5 Entry:12248 Library:NIST05s.LIB
SI:90 Formula:C10H20O2 CAS:334-48-5 MolWeight:172 RetIndex:1372
CompName:n-Decanoic acid $$ Decanoic acid $$ n-Capric acid $$ n-Decoic acid $$ n-Decylic acid $$ Capric acid $$ Caprinic acid $$ Caprynic acid $$ Decoic

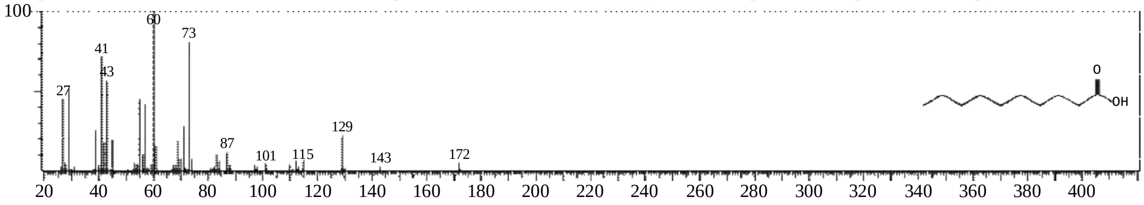

<< Target >>
Line#:19 R.Time:10.692(Scan#:924) MassPeaks:129
RawMode:Averaged 10.683-10.700(923-925) BasePeak:57.10(2344)
BG Mode:Calc. from Peak Group 1 - Event 1

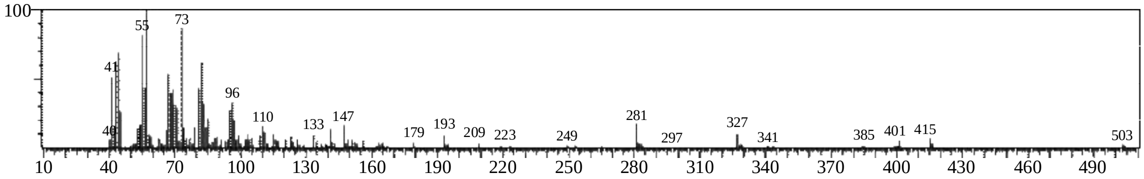

Hit#:1 Entry:14095 Library:NIST05s.LIB
SI:82 Formula:C12H24O CAS:69064-36-4 MolWeight:184 RetIndex:1465
CompName:Z-2-Dodecenol $$ Z-2-Dodecen-1-ol $$ (2Z)-2-Dodecen-1-ol # $$

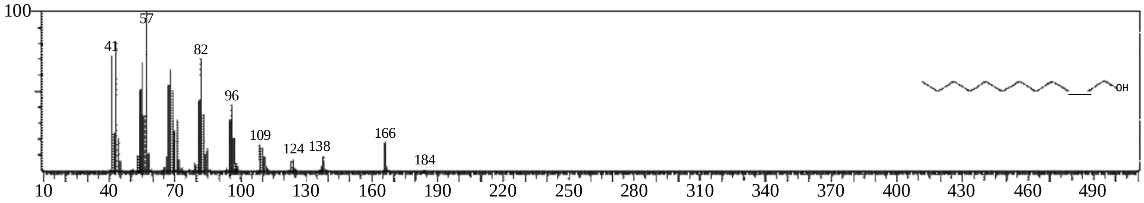

Hit#:2 Entry:14087 Library:NIST05s.LIB
SI:82 Formula:C12H24O CAS:112-54-9 MolWeight:184 RetIndex:1402
CompName:Dodecanal $$ Lauraldehyde $$ n-Dodecanal $$ n-Dodecyl aldehyde $$ n-Dodecylic aldehyde $$ n-Lauraldehyde $$ Aldehyde C-12 $$ Dodecanald

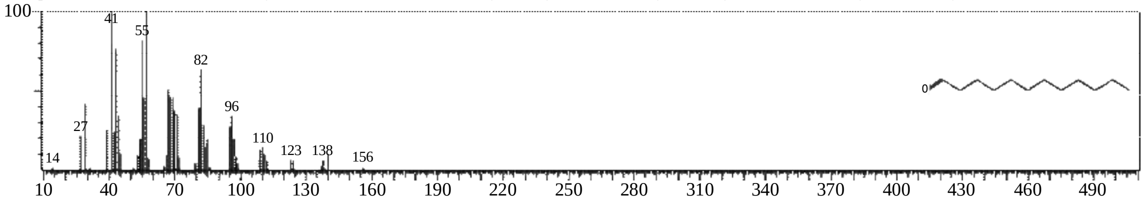

Hit#:3 Entry:11968 Library:NIST05s.LIB
SI:82 Formula:C11H22O CAS:112-44-7 MolWeight:170 RetIndex:1303
CompName:Undecanal $$ n-Undecanal $$ n-Undecyl aldehyde $$ Hendecanal $$ Hendecanaldehyde $$ Undecyl aldehyde $$ Undecylic aldehyde $$ 1-Undecan

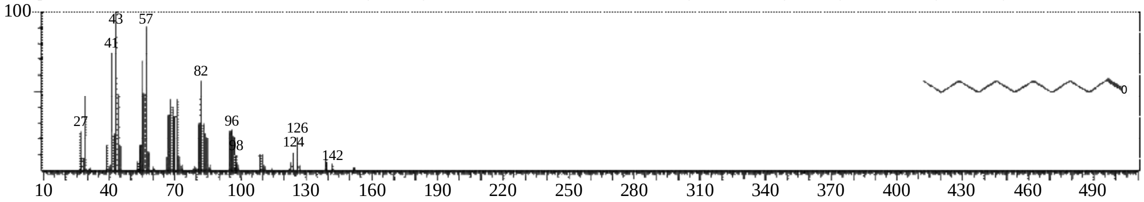

Hit#:4 Entry:48542 Library:NIST05.LIB
SI:81 Formula:C14H28O CAS:0-00-0 MolWeight:212 RetIndex:1664
CompName:E-2-Tetradecen-1-ol

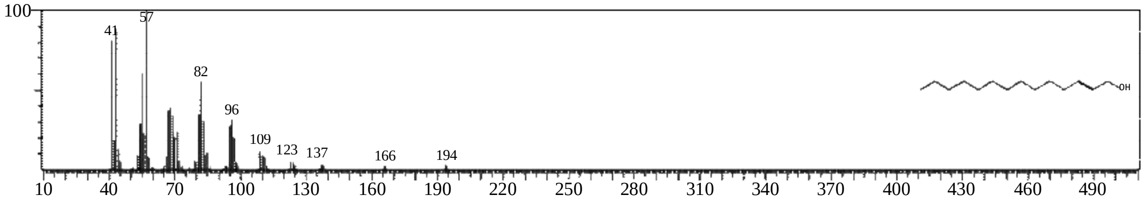

Hit#:5 Entry:17683 Library:NIST05s.LIB
SI:81 Formula:C14H28O CAS:124-25-4 MolWeight:212 RetIndex:1601
CompName:Tetradecanal $$ Myristaldehyde $$ Myristylaldehyde $$ Tetradecylaldehyde $$ n-Tetradecanal $$ Aldehyde C-14 $$ Aldehyde C-14, myristic $$ C-

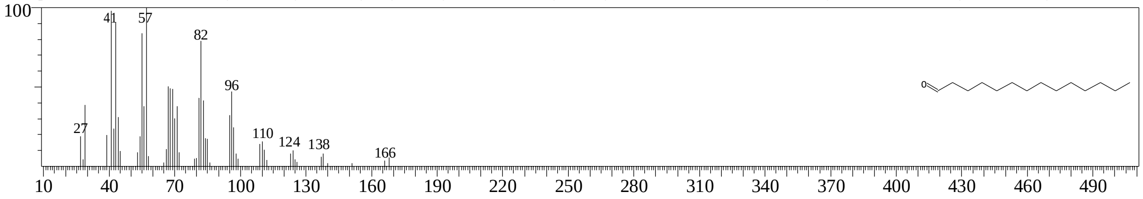

<< Target >>
Line#:20 R.Time:11.383(Scan#:1007) MassPeaks:185
RawMode:Averaged 11.375-11.392(1006-1008) BasePeak:73.05(4790)
BG Mode:Calc. from Peak Group 1 - Event 1

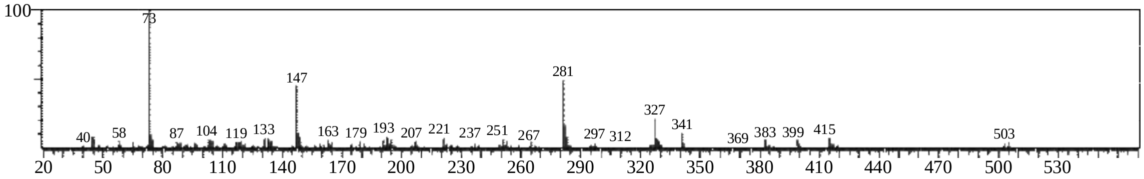

Hit#:1 Entry:158197 Library:NIST05.LIB
SI:74 Formula:C14H42O7Si7 CAS:107-50-6 MolWeight:518 RetIndex:1447
CompName:Cycloheptasiloxane, tetradecamethyl- $$ 2,2,4,4,6,6,8,8,10,10,12,12,14,14-Tetradecamethylcycloheptasiloxane # $$

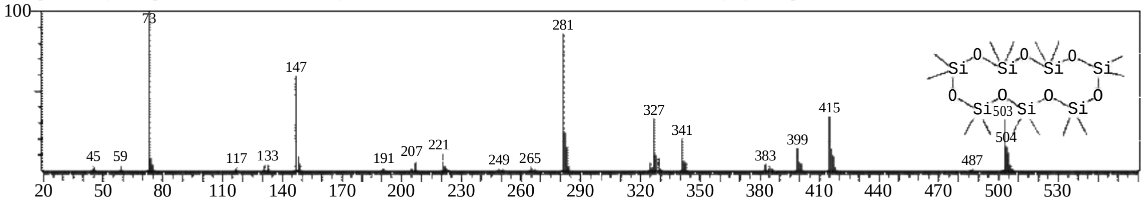

Hit#:2 Entry:150228 Library:NIST05.LIB
SI:70 Formula:C12H38O5Si6 CAS:995-82-4 MolWeight:430 RetIndex:1341
CompName:Hexasiloxane, 1,1,3,3,5,5,7,7,9,9,11,11-dodecamethyl- $$ 1,1,3,3,5,5,7,7,9,9,11,11-Dodecamethylhexasiloxane # $$

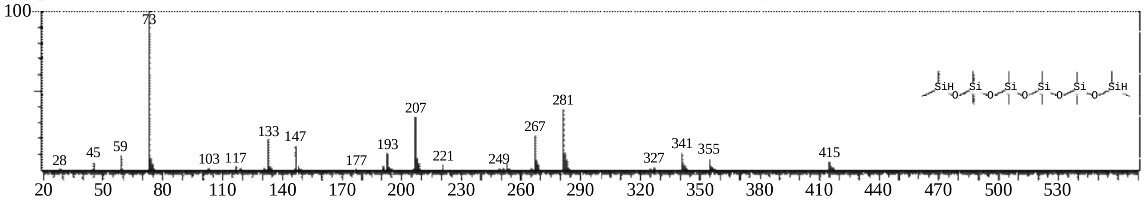

Hit#:3 Entry:160345 Library:NIST05.LIB
SI:69 Formula:C18H52O7Si7 CAS:71579-69-6 MolWeight:576 RetIndex:1648
CompName:3-Isopropoxy-1,1,1,7,7,7-hexamethyl-3,5,5-tris(trimethylsiloxy)tetrasiloxane $$ 1-Isopropoxy-3,3,3-trimethyl-1-[(trimethylsilyl)oxy]disiloxanyl tris

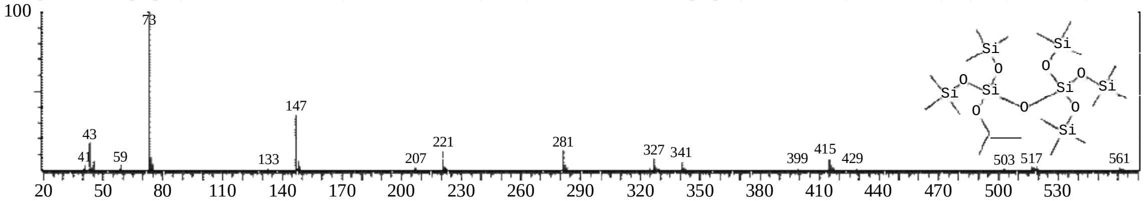

Hit#:4 Entry:139992 Library:NIST05.LIB
SI:69 Formula:C12H36O4Si5 CAS:3555-47-3 MolWeight:384 RetIndex:1068
CompName:Trisiloxane, 1,1,1,5,5,5-hexamethyl-3,3-bis[(trimethylsilyl)oxy]- $$ Tetrakis(trimethylsiloxy)silane $$ Trisiloxane, 1,1,1,5,5,5-hexamethyl-3,3-bis(t

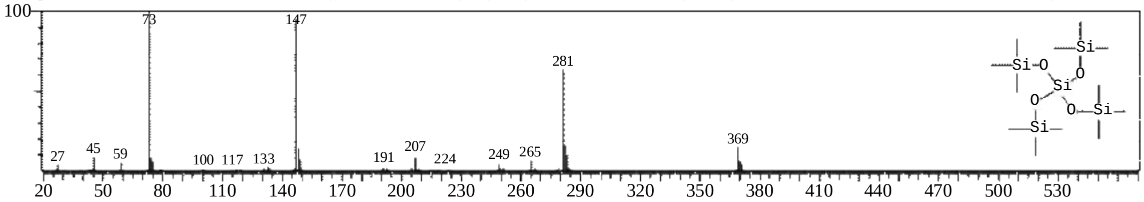

Hit#:5 Entry:139993 Library:NIST05.LIB
SI:68 Formula:C12H36O4Si5 CAS:38146-99-5 MolWeight:384 RetIndex:1068
CompName:1,1,1,3,5,5,7,7,7-Nonamethyl-3-(trimethylsiloxy)tetrasiloxane $$ 1,1,1,3,3,5,7,7,7-Nonamethyl-5-[(trimethylsilyl)oxy]tetrasiloxane # $$

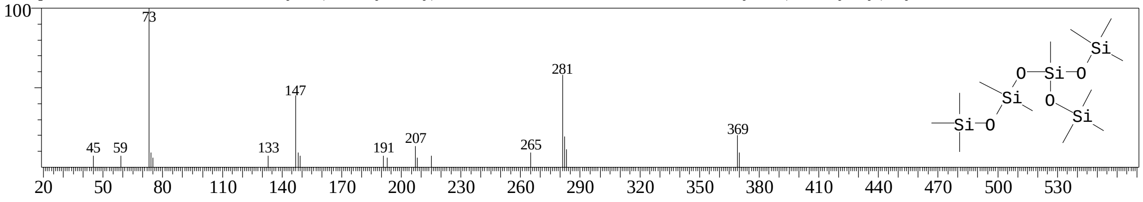

<< Target >>
Line#:21 R.Time:11.558(Scan#:1028) MassPeaks:207
RawMode:Averaged 11.550-11.567(1027-1029) BasePeak:73.10(7015)
BG Mode:Calc. from Peak Group 1 - Event 1

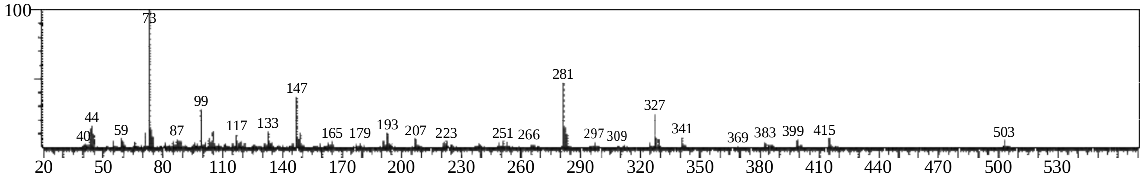

Hit#:1 Entry:160345 Library:NIST05.LIB
SI:67 Formula:C18H52O7Si7 CAS:71579-69-6 MolWeight:576 RetIndex:1648
CompName:3-Isopropoxy-1,1,1,7,7,7-hexamethyl-3,5,5-tris(trimethylsiloxy)tetrasiloxane $$ 1-Isopropoxy-3,3,3-trimethyl-1-[(trimethylsilyl)oxy]disiloxanyl tris

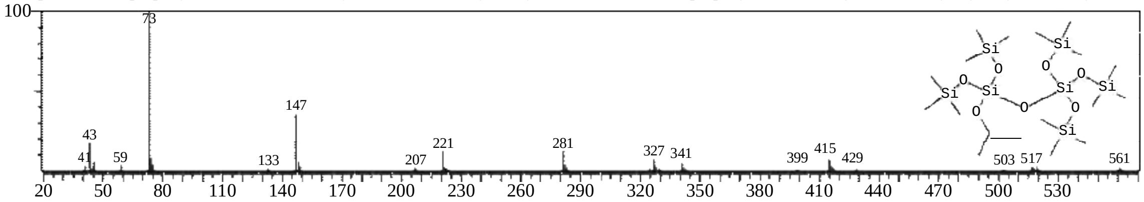

Hit#:2 Entry:158197 Library:NIST05.LIB
SI:67 Formula:C14H42O7Si7 CAS:107-50-6 MolWeight:518 RetIndex:1447
CompName:Cycloheptasiloxane, tetradecamethyl- $$ 2,2,4,4,6,6,8,8,10,10,12,12,14,14-Tetradecamethylcycloheptasiloxane # $$

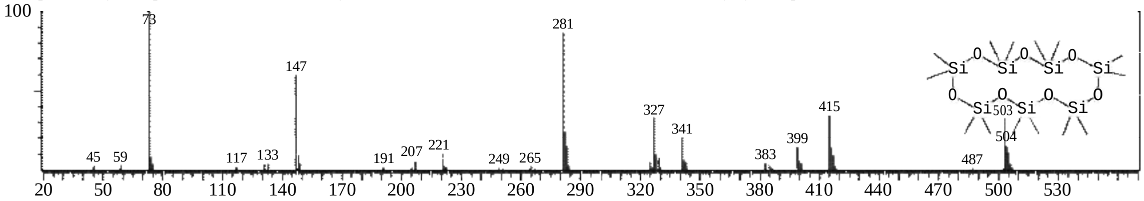

Hit#:3 Entry:139992 Library:NIST05.LIB
SI:66 Formula:C12H36O4Si5 CAS:3555-47-3 MolWeight:384 RetIndex:1068
CompName:Trisiloxane, 1,1,1,5,5,5-hexamethyl-3,3-bis[(trimethylsilyl)oxy]- $$ Tetrakis(trimethylsiloxy)silane $$ Trisiloxane, 1,1,1,5,5,5-hexamethyl-3,3-bis(t

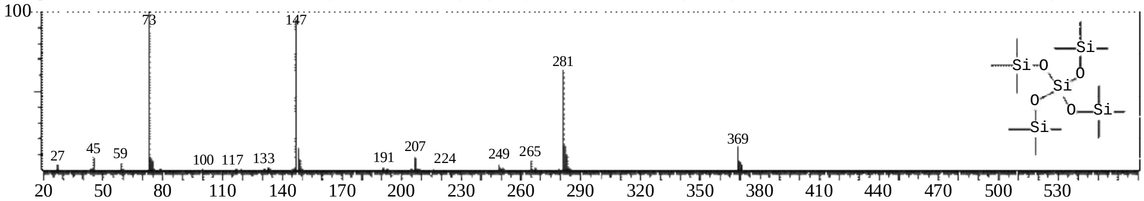

Hit#:4 Entry:150228 Library:NIST05.LIB
SI:66 Formula:C12H38O5Si6 CAS:995-82-4 MolWeight:430 RetIndex:1341
CompName:Hexasiloxane, 1,1,3,3,5,5,7,7,9,9,11,11-dodecamethyl- $$ 1,1,3,3,5,5,7,7,9,9,11,11-Dodecamethylhexasiloxane # $$

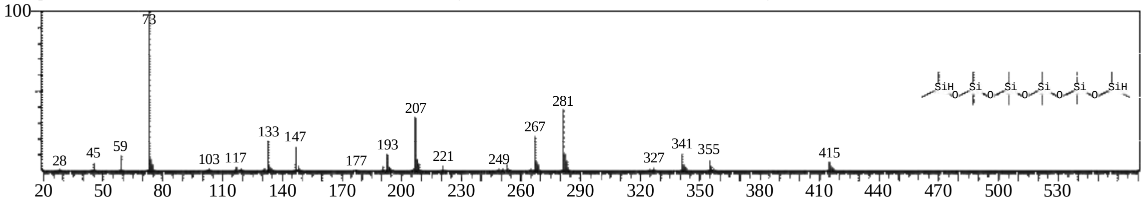

Hit#:5 Entry:160407 Library:NIST05.LIB
SI:64 Formula:C16H50O7Si8 CAS:19095-24-0 MolWeight:578 RetIndex:1710
CompName:Octasiloxane, 1,1,3,3,5,5,7,7,9,9,11,11,13,13,15,15-hexadecamethyl- $$ 1,1,3,3,5,5,7,7,9,9,11,11,13,13,15,15-Hexadecamethyloctasiloxane # $$

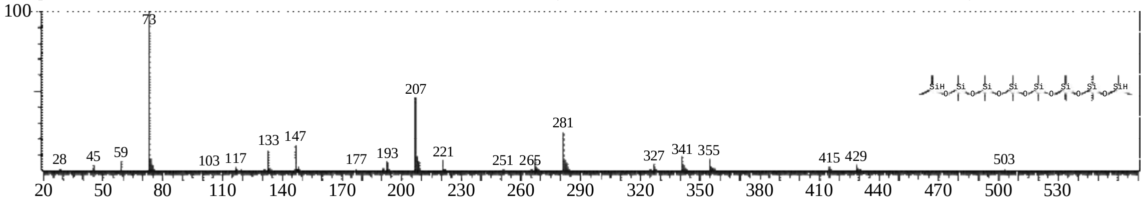

<< Target >>
Line#:22 R.Time:11.775(Scan#:1054) MassPeaks:63
RawMode:Averaged 11.767-11.783(1053-1055) BasePeak:57.10(6525)
BG Mode:Calc. from Peak Group 1 - Event 1

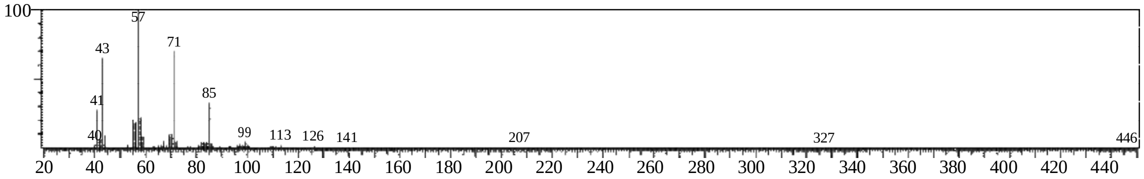

Hit#:1 Entry:48603 Library:NIST05.LIB
SI:90 Formula:C15H32 CAS:31295-56-4 MolWeight:212 RetIndex:1320
CompName:Dodecane, 2,6,11-trimethyl- $$ 2,6,11-Trimethyldodecane $$

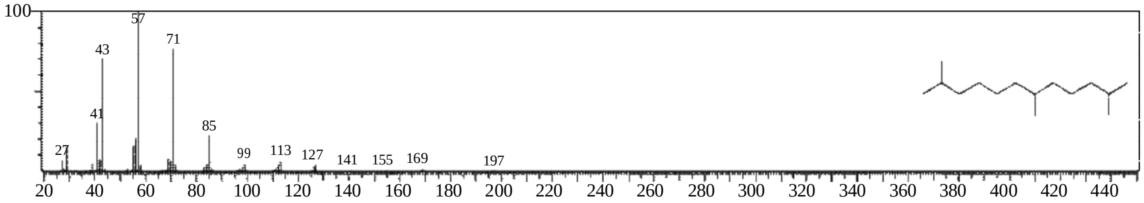

Hit#:2 Entry:48605 Library:NIST05.LIB
SI:89 Formula:C15H32 CAS:74645-98-0 MolWeight:212 RetIndex:1320
CompName:Dodecane, 2,7,10-trimethyl- $$ 2,7,10-Trimethyldodecane # $$

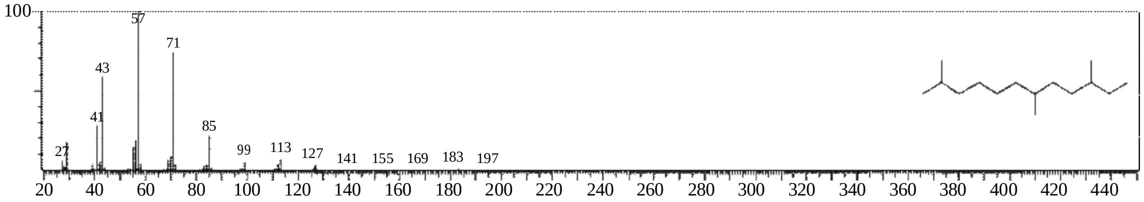

Hit#:3 Entry:32413 Library:NIST05.LIB
SI:89 Formula:C13H28 CAS:31081-17-1 MolWeight:184 RetIndex:1185
CompName:Nonane, 2-methyl-5-propyl- $$ 2-Methyl-5-propylnonane # $$

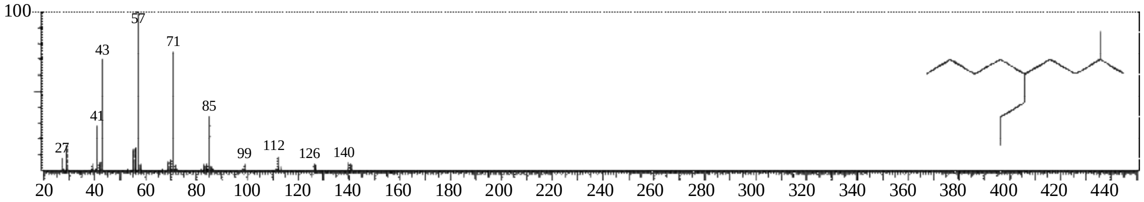

Hit#:4 Entry:57192 Library:NIST05.LIB
SI:89 Formula:C16H34 CAS:6165-40-8 MolWeight:226 RetIndex:1548
CompName:Pentadecane, 7-methyl- $$ 7-Methylpentadecane # $$

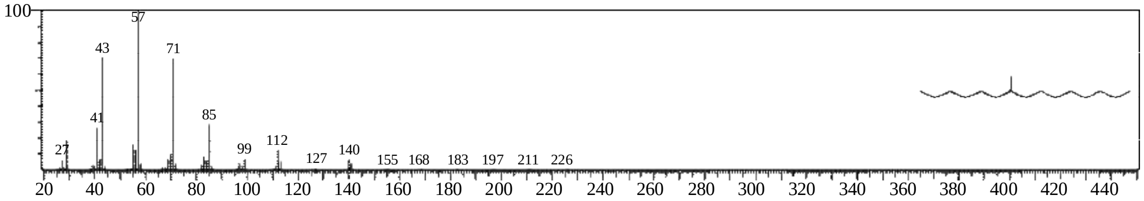

Hit#:5 Entry:12008 Library:NIST05s.LIB
SI:89 Formula:C12H26 CAS:112-40-3 MolWeight:170 RetIndex:1214
CompName:Dodecane $$ n-Dodecane $$ Adakane 12 $$ Ba 51-090453 $$ CH3(CH2)10CH3 $$ Bihexyl $$ Dihexyl $$ n-Dodecane min $$ Duodecane $$

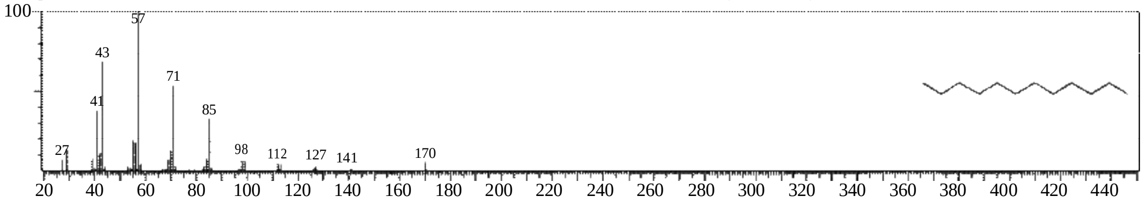

<< Target >>
Line#:23 R.Time:11.908(Scan#:1070) MassPeaks:88
RawMode:Averaged 11.900-11.917(1069-1071) BasePeak:99.10(13908)
BG Mode:Calc. from Peak Group 1 - Event 1

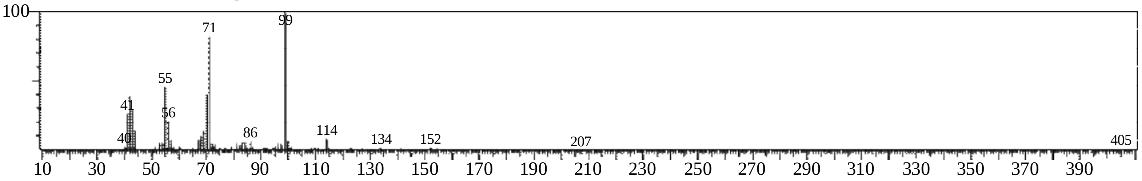

Hit#:1 Entry:11958 Library:NIST05s.LIB
SI:91 Formula:C10H18O2 CAS:705-86-2 MolWeight:170 RetIndex:1404
CompName:2H-Pyran-2-one, tetrahydro-6-pentyl- $$ .delta.-Amylvalerolactone $$ .delta.-Decalactone $$ Decanoic acid, 5-hydroxy-, .delta.-lactone $$ 5-Decan

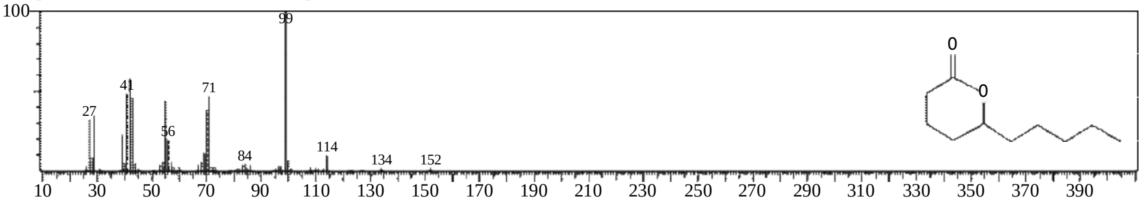

Hit#:2 Entry:9696 Library:NIST05s.LIB
SI:91 Formula:C9H16O2 CAS:3301-94-8 MolWeight:156 RetIndex:1304
CompName:.delta. Nonalactone $$ 2H-Pyran-2-one, 6-butyltetrahydro- $$ 2H-Pyran-2-one, tetrahydro-6-butyl $$ .beta.-Nonalactone $$ 6-Butyltetrahydro-2H-p

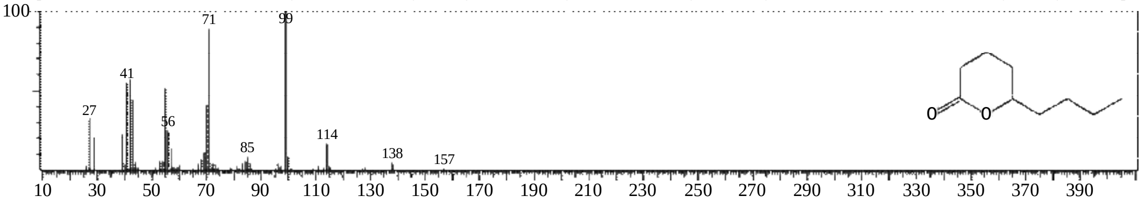

Hit#:3 Entry:11959 Library:NIST05s.LIB
SI:91 Formula:C10H18O2 CAS:705-86-2 MolWeight:170 RetIndex:1404
CompName:2H-Pyran-2-one, tetrahydro-6-pentyl- $$ .delta.-Amylvalerolactone $$ .delta.-Decalactone $$ Decanoic acid, 5-hydroxy-, .delta.-lactone $$ 5-Decan

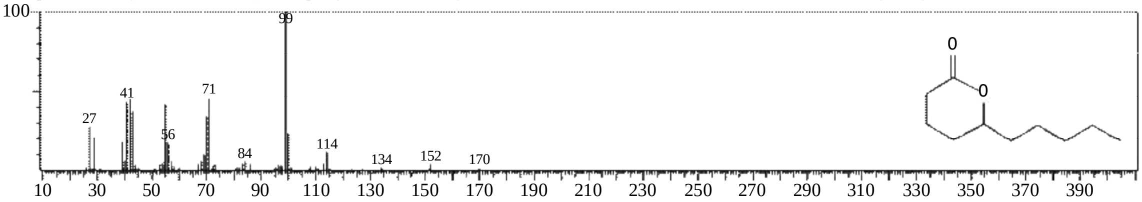

Hit#:4 Entry:24845 Library:NIST05.LIB
SI:90 Formula:C10H18O2 CAS:705-86-2 MolWeight:170 RetIndex:1404
CompName:2H-Pyran-2-one, tetrahydro-6-pentyl- $$ .delta.-Amylvalerolactone $$ .delta.-Decalactone $$ Decanoic acid, 5-hydroxy-, .delta.-lactone $$ 5-Decan

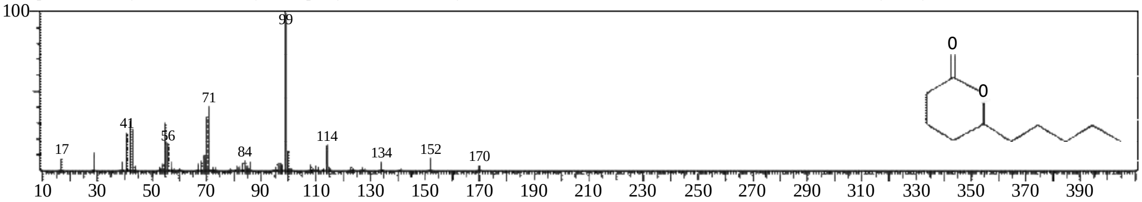

Hit#:5 Entry:17870 Library:NIST05.LIB
SI:89 Formula:C9H16O2 CAS:3301-94-8 MolWeight:156 RetIndex:1304
CompName:.delta. Nonalactone $$ 2H-Pyran-2-one, 6-butyltetrahydro- $$ 2H-Pyran-2-one, tetrahydro-6-butyl $$ .beta.-Nonalactone $$ 6-Butyltetrahydro-2H-p

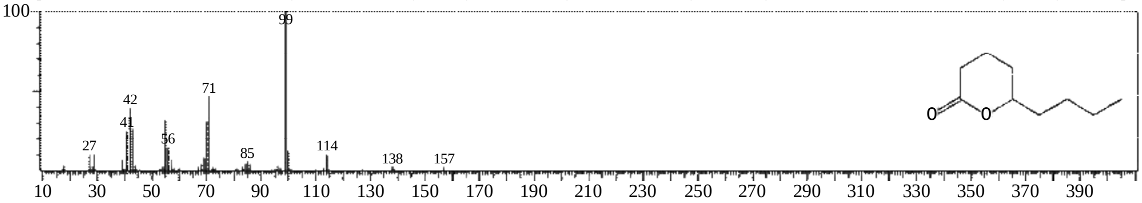

<< Target >>
Line#:24 R.Time:12.592(Scan#:1152) MassPeaks:174
RawMode:Averaged 12.583-12.600(1151-1153) BasePeak:73.05(425051)
BG Mode:Calc. from Peak Group 1 - Event 1

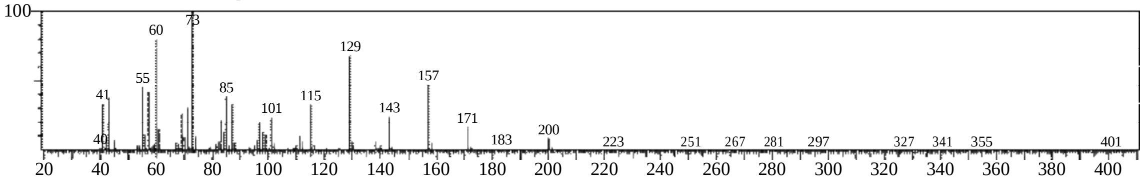

Hit#:1 Entry:16246 Library:NIST05s.LIB
SI:95 Formula:C12H24O2 CAS:143-07-7 MolWeight:200 RetIndex:1570
CompName:Dodecanoic acid $$ n-Dodecanoic acid $$ Neo-fat 12 $$ Aliphat no. 4 $$ Abl $$ Dodecylic acid $$ Lauric acid $$ Laurostearic acid $$ Neo-fat 12-

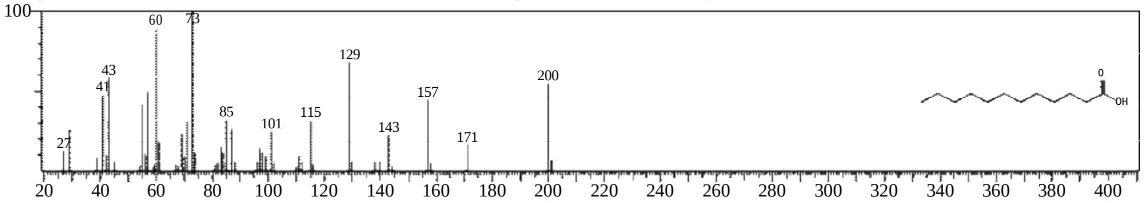

Hit#:2 Entry:16239 Library:NIST05s.LIB
SI:91 Formula:C12H24O2 CAS:143-07-7 MolWeight:200 RetIndex:1570
CompName:Dodecanoic acid $$ n-Dodecanoic acid $$ Neo-fat 12 $$ Aliphat no. 4 $$ Abl $$ Dodecylic acid $$ Lauric acid $$ Laurostearic acid $$ Neo-fat 12-

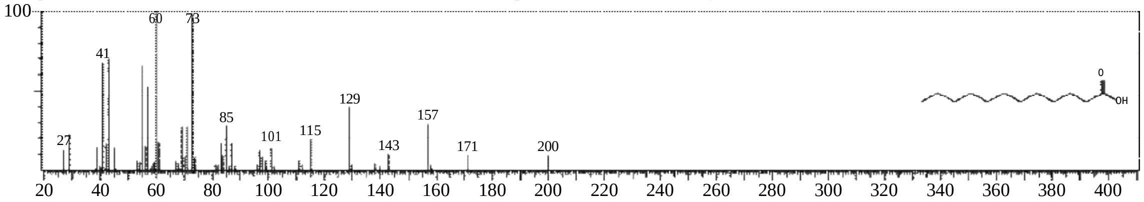

Hit#:3 Entry:41176 Library:NIST05.LIB
SI:90 Formula:C12H24O2 CAS:143-07-7 MolWeight:200 RetIndex:1570
CompName:Dodecanoic acid $$ n-Dodecanoic acid $$ Neo-fat 12 $$ Aliphat no. 4 $$ Abl $$ Dodecylic acid $$ Lauric acid $$ Laurostearic acid $$ Neo-fat 12-

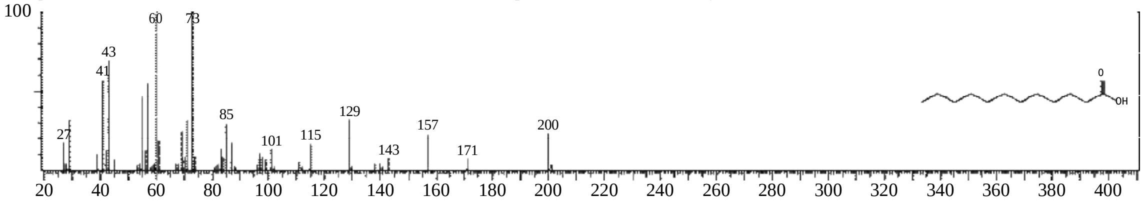

Hit#:4 Entry:16245 Library:NIST05s.LIB
SI:89 Formula:C12H24O2 CAS:143-07-7 MolWeight:200 RetIndex:1570
CompName:Dodecanoic acid $$ n-Dodecanoic acid $$ Neo-fat 12 $$ Aliphat no. 4 $$ Abl $$ Dodecylic acid $$ Lauric acid $$ Laurostearic acid $$ Neo-fat 12-

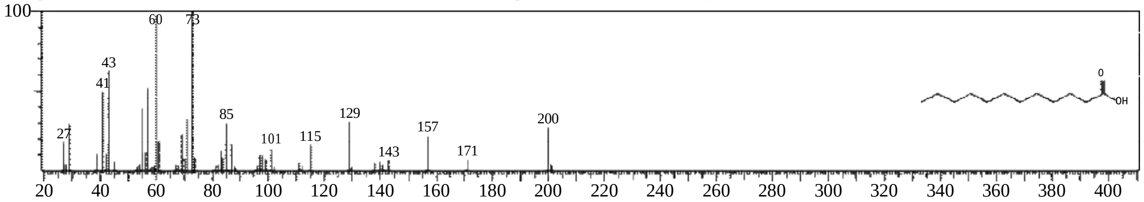

Hit#:5 Entry:17905 Library:NIST05s.LIB
SI:88 Formula:C13H26O2 CAS:638-53-9 MolWeight:214 RetIndex:1670
CompName:Tridecanoic acid $$ n-Tridecanoic acid $$ n-Tridecoic acid $$ Tridecylic acid $$

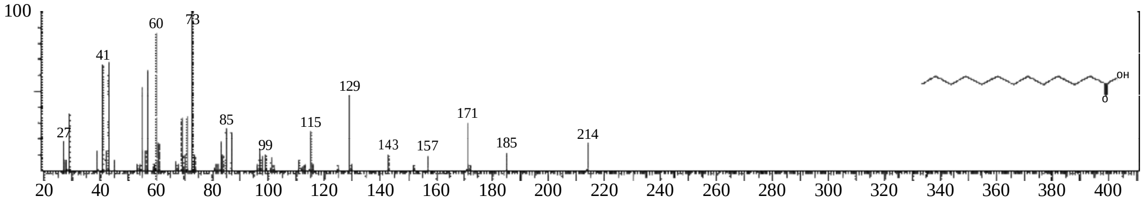

<< Target >>
Line#:25 R.Time:12.925(Scan#:1192) MassPeaks:95
RawMode:Averaged 12.917-12.933(1191-1193) BasePeak:88.10(7687)
BG Mode:Calc. from Peak Group 1 - Event 1

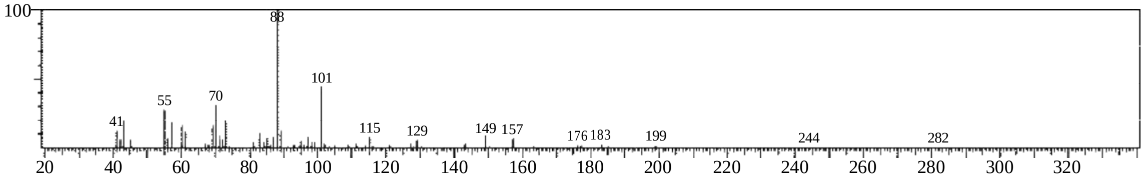

Hit#:1 Entry:122711 Library:NIST05.LIB
SI:87 Formula:C22H44O2 CAS:18281-05-5 MolWeight:340 RetIndex:2375
CompName:Eicosanoic acid, ethyl ester $$ Ethyl icosanoate # $$

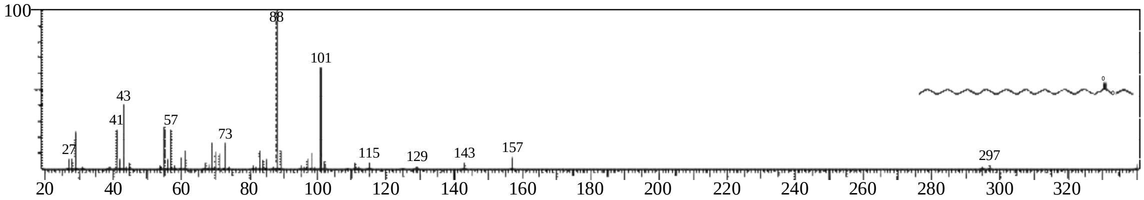

Hit#:2 Entry:21343 Library:NIST05s.LIB
SI:86 Formula:C16H32O2 CAS:124-06-1 MolWeight:256 RetIndex:1779
CompName:Tetradecanoic acid, ethyl ester $$ Myristic acid, ethyl ester $$ Ethyl myristate $$ Ethyl tetradecanoate $$ Ethyl ester of tetradecanoic acid $$ Ethyl

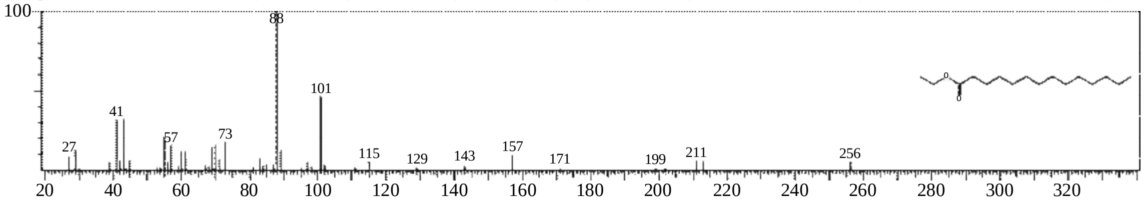

Hit#:3 Entry:83499 Library:NIST05.LIB
SI:86 Formula:C17H34O2 CAS:41114-00-5 MolWeight:270 RetIndex:1878
CompName:Pentadecanoic acid, ethyl ester $$ Ethyl pentadecanoate $$ n-Pentadecanoic acid ethyl ester $$

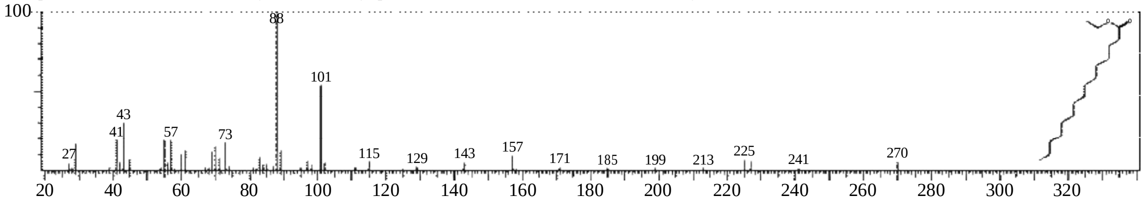

Hit#:4 Entry:23661 Library:NIST05s.LIB
SI:85 Formula:C19H38O2 CAS:14010-23-2 MolWeight:298 RetIndex:2077
CompName:Heptadecanoic acid, ethyl ester $$ Ethyl heptadecanoate $$ Ethyl n-heptadecanoate $$

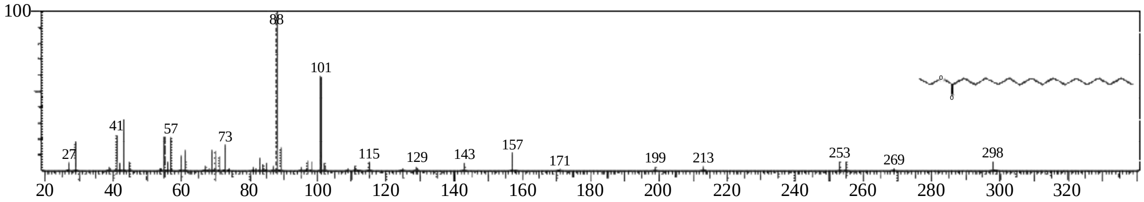

Hit#:5 Entry:17913 Library:NIST05s.LIB
SI:85 Formula:C13H26O2 CAS:627-90-7 MolWeight:214 RetIndex:1481
CompName:Undecanoic acid, ethyl ester $$ Ethyl undecanoate $$ Ethyl undecylate $$ n-Undecanoic acid ethyl ester $$ Ethyl n-undecanoate $$

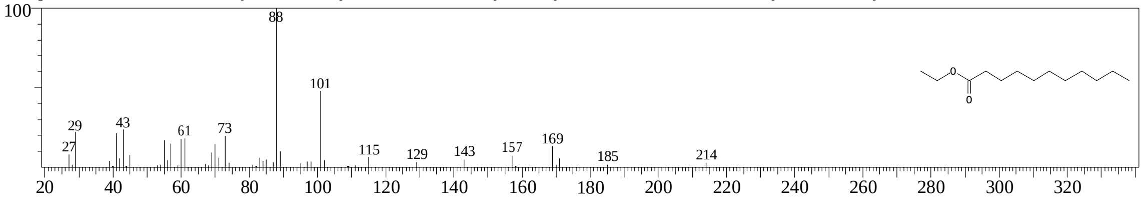

<< Target >>
Line#:26 R.Time:13.542(Scan#:1266) MassPeaks:174
RawMode:Averaged 13.533-13.550(1265-1267) BasePeak:73.05(4716)
BG Mode:Calc. from Peak Group 1 - Event 1

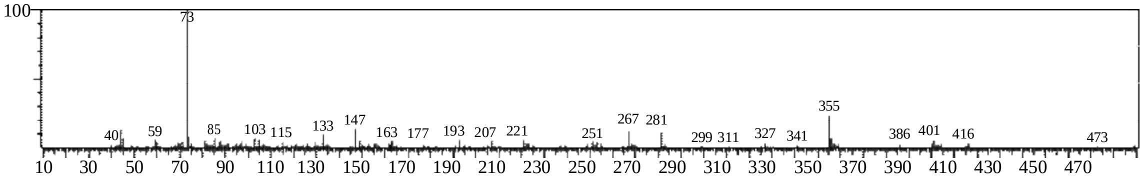

Hit#:1 Entry:25961 Library:NIST05s.LIB
SI:69 Formula:C16H30O4Si3 CAS:10586-16-0 MolWeight:370 RetIndex:1765
CompName:Benzoic acid, 2,4-bis[(trimethylsilyl)oxy]-, trimethylsilyl ester $$ .beta.-Resorcylic acid (tms) $$ 2,4-Bis(trimethylsiloxy)trimethylsilylbenzoate $$

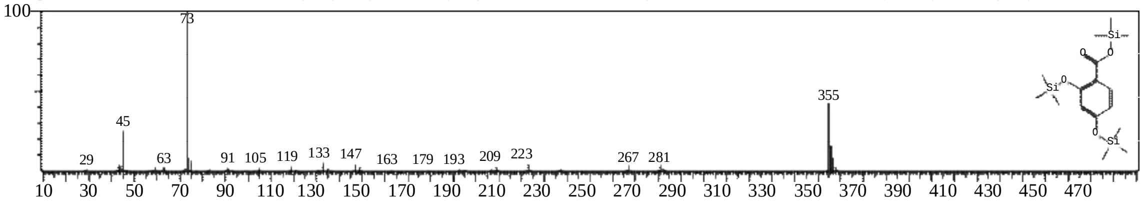

Hit#:2 Entry:25962 Library:NIST05s.LIB
SI:68 Formula:C16H30O4Si3 CAS:3618-20-0 MolWeight:370 RetIndex:1765
CompName:Benzoic acid, 2,5-bis(trimethylsiloxy)-, trimethylsilyl ester $$ Gentisic acid (tms) $$ Trimethylsilyl 2,5-bis[(trimethylsilyl)oxy]benzoate # $$

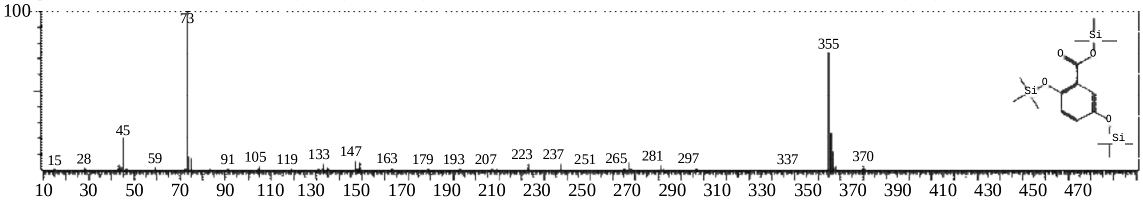

Hit#:3 Entry:135187 Library:NIST05.LIB
SI:67 Formula:C16H30O4Si3 CAS:3782-85-2 MolWeight:370 RetIndex:1765
CompName:Benzoic acid, 2,6-bis[(trimethylsilyl)oxy]-, trimethylsilyl ester $$ Trimethylsilyl 2,6-bis[(trimethylsilyl)oxy]benzoate # $$

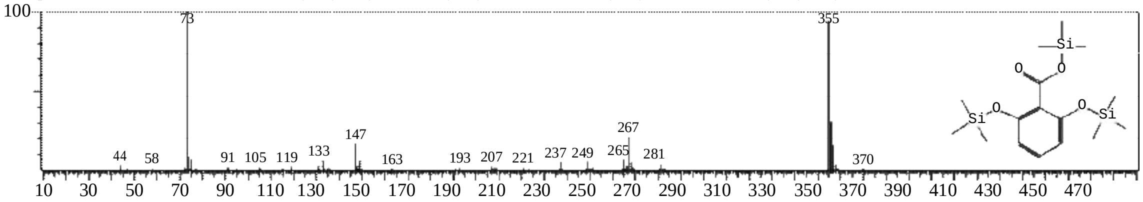

Hit#:4 Entry:157431 Library:NIST05.LIB
SI:67 Formula:C14H44O6Si7 CAS:19095-23-9 MolWeight:504 RetIndex:1526
CompName:Heptasiloxane, 1,1,3,3,5,5,7,7,9,9,11,11,13,13-tetradecamethyl- $$ 1,1,3,3,5,5,7,7,9,9,11,11,13,13-Tetradecamethylheptasiloxane # $$

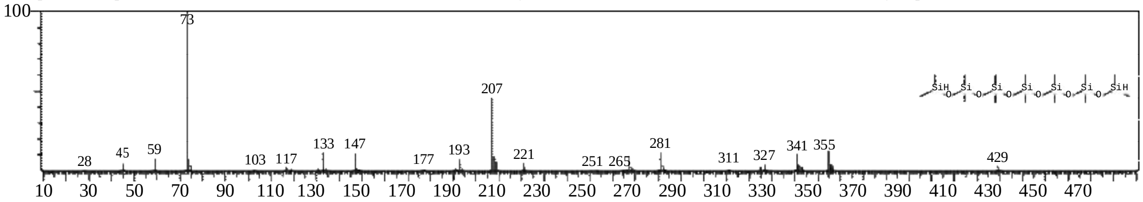

Hit#:5 Entry:135189 Library:NIST05.LIB
SI:66 Formula:C16H30O4Si3 CAS:10586-16-0 MolWeight:370 RetIndex:1765
CompName:Benzoic acid, 2,4-bis[(trimethylsilyl)oxy]-, trimethylsilyl ester $$ .beta.-Resorcylic acid (tms) $$ 2,4-Bis(trimethylsiloxy)trimethylsilylbenzoate $$

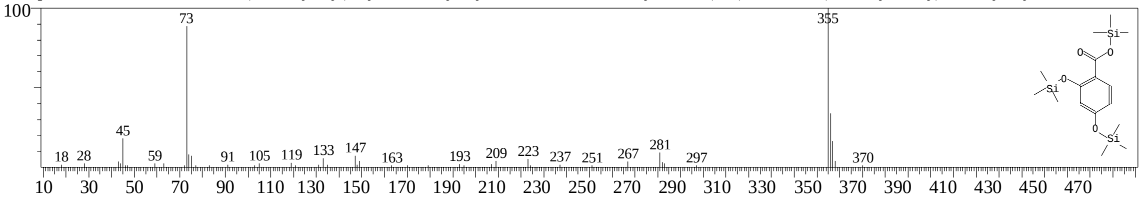

Analytical Research & Metallurgical Laboratories Pvt. Ltd.

<< Target >>
Line#:27 R.Time:13.650(Scan#:1279) MassPeaks:197
RawMode:Averaged 13.642-13.658(1278-1280) BasePeak:73.05(32870)
BG Mode:Calc. from Peak Group 1 - Event 1

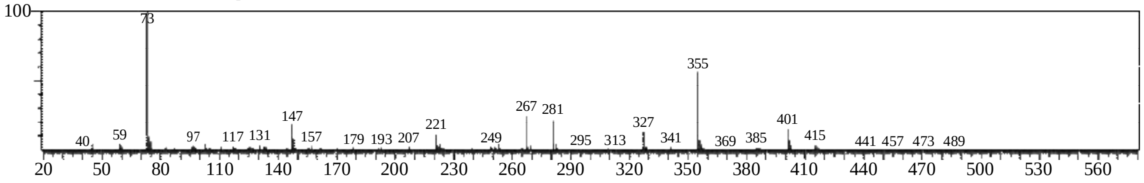

Hit#:1 Entry:27473 Library:NIST05s.LIB
SI:74 Formula:C16H48O8Si8 CAS:556-68-3 MolWeight:592 RetIndex:1654
CompName:Cyclooctasiloxane, hexadecamethyl- $$ Hexadecamethyl-cyclooctasioxane $$ Hexadecamethylcyclooctasiloxane $$ 2,2,4,4,6,6,8,8,10,10,12,12,14,

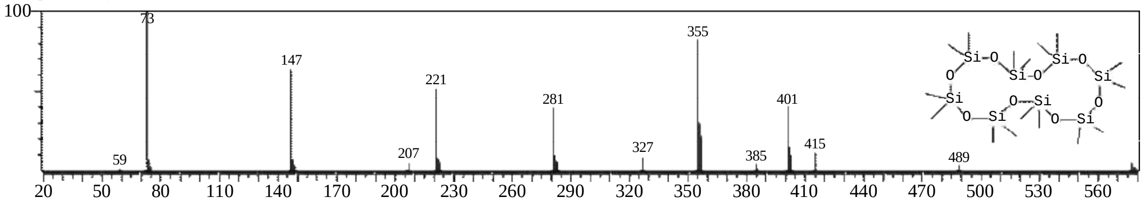

Hit#:2 Entry:160758 Library:NIST05.LIB
SI:74 Formula:C16H48O8Si8 CAS:556-68-3 MolWeight:592 RetIndex:1654
CompName:Cyclooctasiloxane, hexadecamethyl- $$ Hexadecamethyl-cyclooctasioxane $$ Hexadecamethylcyclooctasiloxane $$ 2,2,4,4,6,6,8,8,10,10,12,12,14,

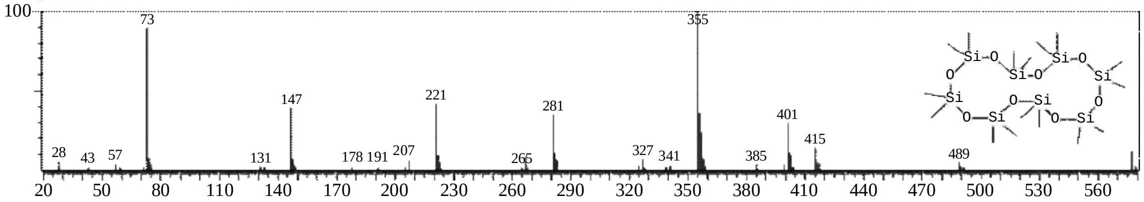

Hit#:3 Entry:150228 Library:NIST05.LIB
SI:74 Formula:C12H38O5Si6 CAS:995-82-4 MolWeight:430 RetIndex:1341
CompName:Hexasiloxane, 1,1,3,3,5,5,7,7,9,9,11,11-dodecamethyl- $$ 1,1,3,3,5,5,7,7,9,9,11,11-Dodecamethylhexasiloxane # $$

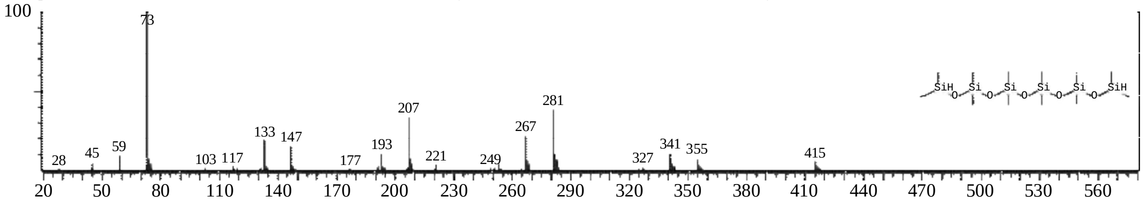

Hit#:4 Entry:160407 Library:NIST05.LIB
SI:72 Formula:C16H50O7Si8 CAS:19095-24-0 MolWeight:578 RetIndex:1710
CompName:Octasiloxane, 1,1,3,3,5,5,7,7,9,9,11,11,13,13,15,15-hexadecamethyl- $$ 1,1,3,3,5,5,7,7,9,9,11,11,13,13,15,15-Hexadecamethyloctasiloxane # $$

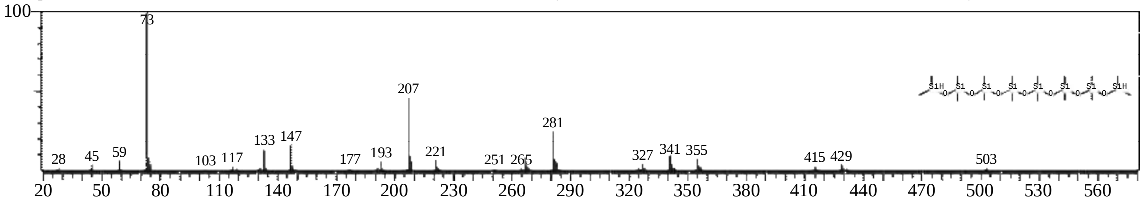

Hit#:5 Entry:135187 Library:NIST05.LIB
SI:72 Formula:C16H30O4Si3 CAS:3782-85-2 MolWeight:370 RetIndex:1765
CompName:Benzoic acid, 2,6-bis[(trimethylsilyl)oxy]-, trimethylsilyl ester $$ Trimethylsilyl 2,6-bis[(trimethylsilyl)oxy]benzoate # $$

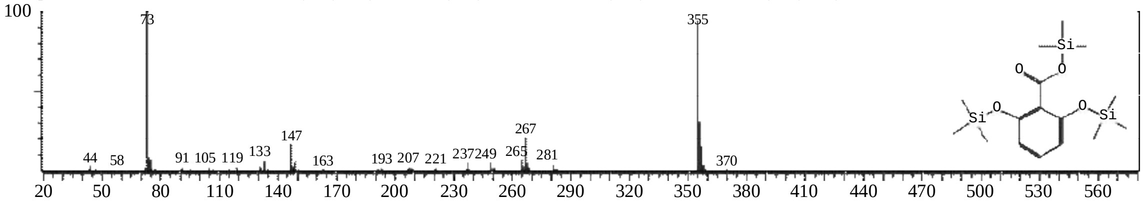

<< Target >>
Line#:28 R.Time:13.983(Scan#:1319) MassPeaks:113
RawMode:Averaged 13.975-13.992(1318-1320) BasePeak:57.10(32838)
BG Mode:Calc. from Peak Group 1 - Event 1

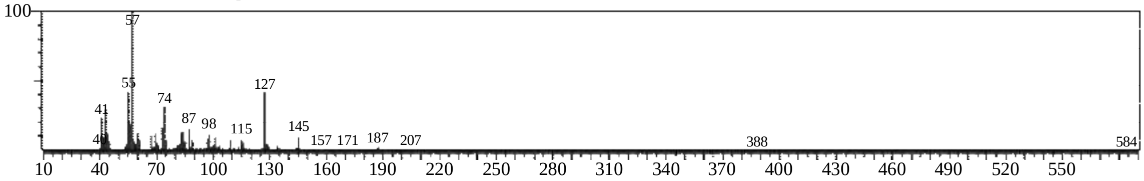

Hit#:1 Entry:41154 Library:NIST05.LIB
SI:82 Formula:C12H24O2 CAS:589-75-3 MolWeight:200 RetIndex:1381
CompName:Butyl caprylate $$ Caprylic acid n-butyl ester $$ n-Caprylic acid n-butyl ester $$ Octanoic acid, butyl ester $$ n-Butylcaprylate $$ Butyl octanoate

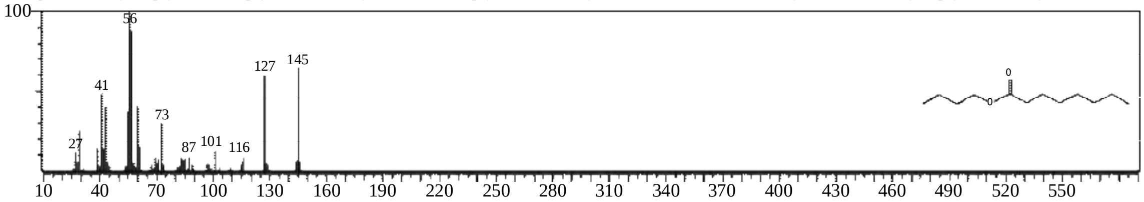

Hit#:2 Entry:41160 Library:NIST05.LIB
SI:82 Formula:C12H24O2 CAS:5461-06-3 MolWeight:200 RetIndex:1317
CompName:n-Caprylic acid isobutyl ester $$ Octanoic acid, 2-methylpropyl ester $$ iso-Butyl caprylate $$ Octanoic acid, isobutyl ester $$ iso-Butyl n-octanoa

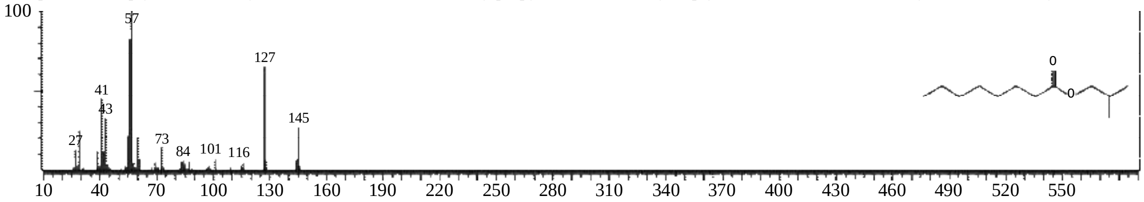

Hit#:3 Entry:41163 Library:NIST05.LIB
SI:81 Formula:C12H24O2 CAS:5458-61-7 MolWeight:200 RetIndex:1317
CompName:Octanoic acid, 2-butyl ester $$ Sec-butyl octanoate # $$

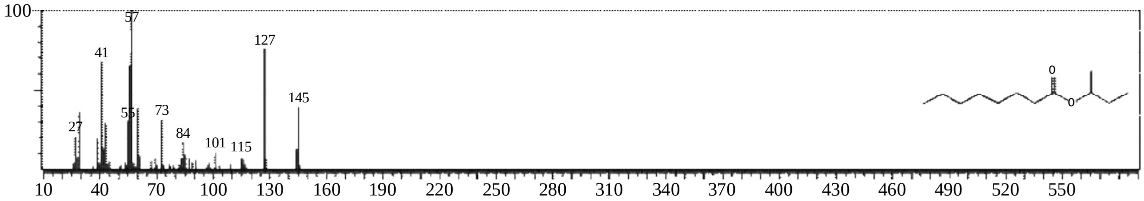

Hit#:4 Entry:66531 Library:NIST05.LIB
SI:81 Formula:C15H30O2 CAS:4265-97-8 MolWeight:242 RetIndex:1680
CompName:Heptyl caprylate $$ Octanoic acid, heptyl ester $$ Heptyl octanoate $$ n-Heptyl n-octanoate $$

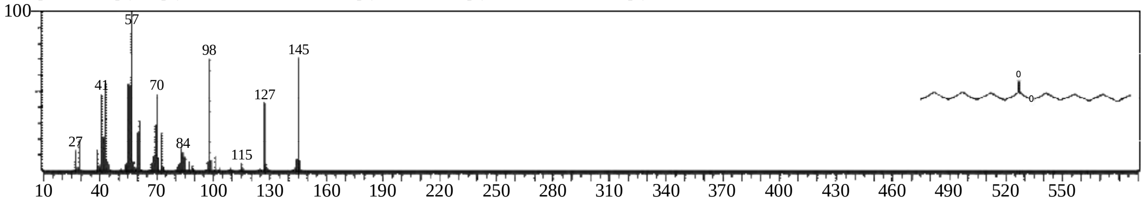

Hit#:5 Entry:83373 Library:NIST05.LIB
SI:81 Formula:C16H30O3 CAS:623-66-5 MolWeight:270 RetIndex:1915
CompName:Caprylic anhydride $$ Octanoic acid, anhydride $$ Octanoic anhydride $$

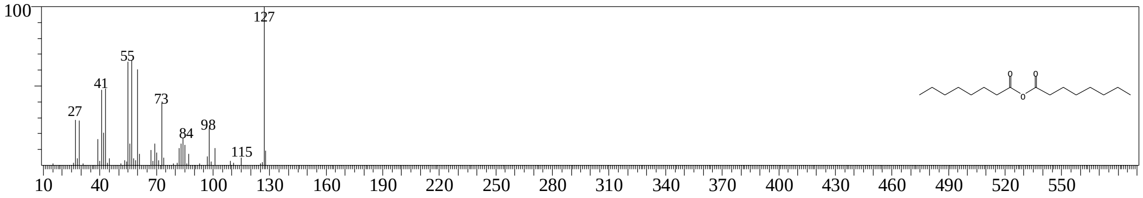

<< Target >>
Line#:29 R.Time:14.192(Scan#:1344) MassPeaks:114
RawMode:Averaged 14.183-14.200(1343-1345) BasePeak:73.05(4122)
BG Mode:Calc. from Peak Group 1 - Event 1

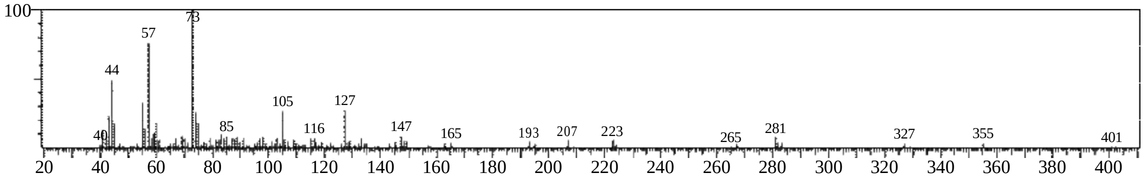

Hit#:1 Entry:21058 Library:NIST05.LIB
SI:75 Formula:C6H12O5 CAS:0-00-0 MolWeight:164 RetIndex:1577
CompName:1,5-Anhydro-d-talitol

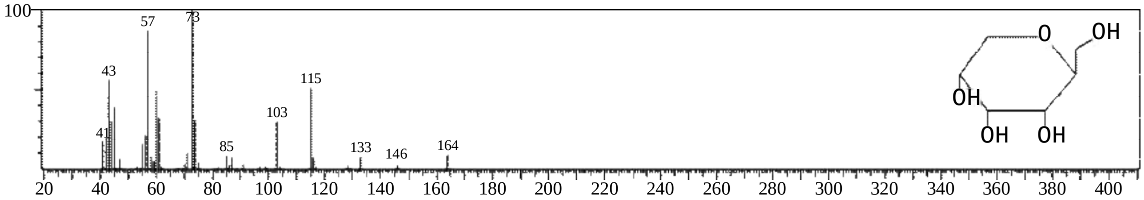

Hit#:2 Entry:88878 Library:NIST05.LIB
SI:74 Formula:C13H28O6 CAS:0-00-0 MolWeight:280 RetIndex:2281
CompName:d-Glucitol, 1-O-heptyl-

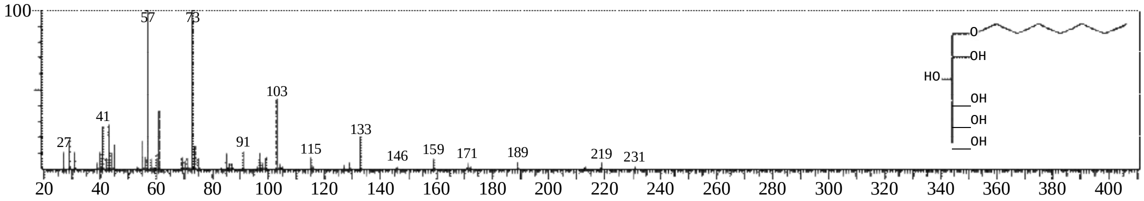

Hit#:3 Entry:21052 Library:NIST05.LIB
SI:74 Formula:C6H12O5 CAS:0-00-0 MolWeight:164 RetIndex:1577
CompName:1,5-Anhydro-d-mannitol

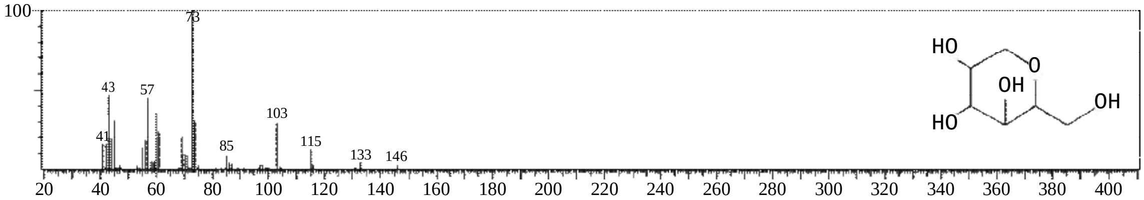

Hit#:4 Entry:52499 Library:NIST05.LIB
SI:74 Formula:C9H16O6 CAS:0-00-0 MolWeight:220 RetIndex:1643
CompName:Methyl 4,6-ethylidene-.alpha.-d-galactopyranoside

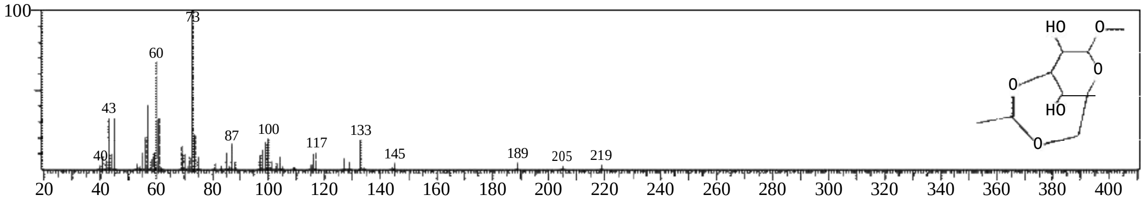

Hit#:5 Entry:88881 Library:NIST05.LIB
SI:73 Formula:C13H28O6 CAS:126403-07-4 MolWeight:280 RetIndex:2281
CompName:d-Mannitol, 1-O-heptyl- $$ 1-O-Heptyl-d-mannitol # $$

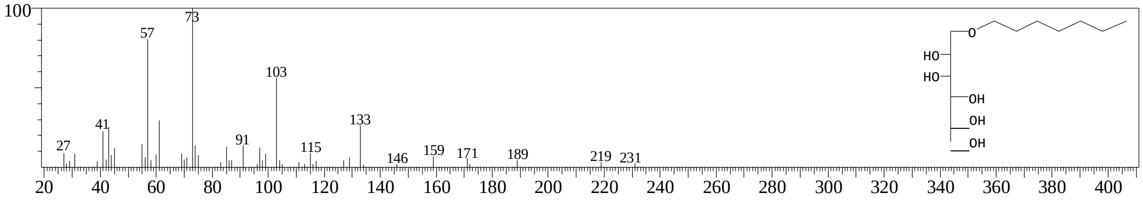

<< Target >>
Line#:30 R.Time:14.400(Scan#:1369) MassPeaks:95
RawMode:Averaged 14.392-14.408(1368-1370) BasePeak:99.10(6429)
BG Mode:Calc. from Peak Group 1 - Event 1

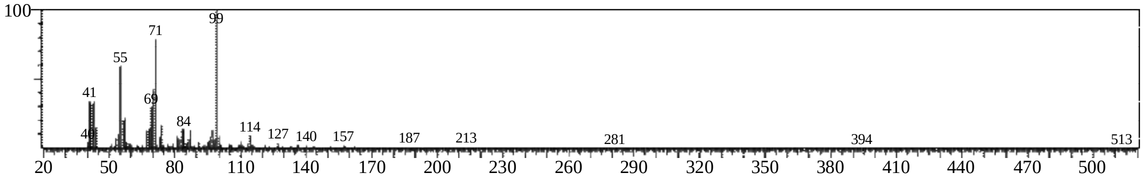

Hit#:1 Entry:9696 Library:NIST05s.LIB
SI:87 Formula:C9H16O2 CAS:3301-94-8 MolWeight:156 RetIndex:1304
CompName:.delta. Nonalactone $$ 2H-Pyran-2-one, 6-butyltetrahydro- $$ 2H-Pyran-2-one, tetrahydro-6-butyl $$ .beta.-Nonalactone $$ 6-Butyltetrahydro-2H-p

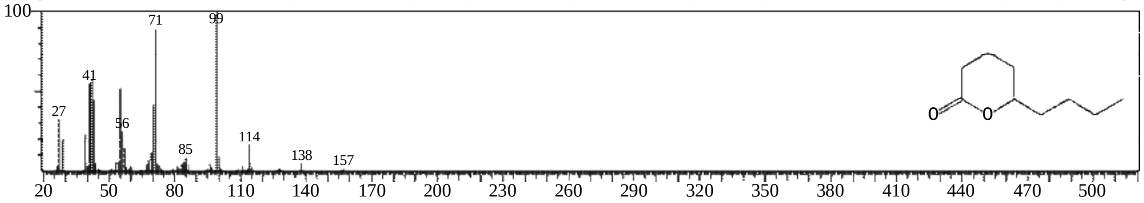

Hit#:2 Entry:15996 Library:NIST05s.LIB
SI:86 Formula:C12H22O2 CAS:713-95-1 MolWeight:198 RetIndex:1602
CompName:2H-Pyran-2-one, 6-heptyltetrahydro- $$ .delta.-Dodecalactone $$ .delta.-Heptyl-.delta.-valerolactone $$ Dodecanoic acid, 5-hydroxy-, .delta.-lacton

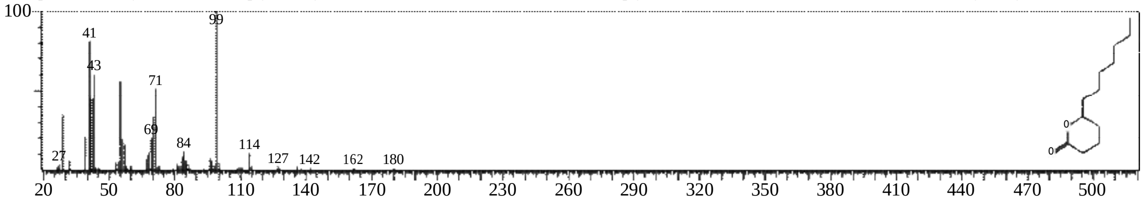

Hit#:3 Entry:11958 Library:NIST05s.LIB
SI:84 Formula:C10H18O2 CAS:705-86-2 MolWeight:170 RetIndex:1404
CompName:2H-Pyran-2-one, tetrahydro-6-pentyl- $$ .delta.-Amylvalerolactone $$ .delta.-Decalactone $$ Decanoic acid, 5-hydroxy-, .delta.-lactone $$ 5-Decan

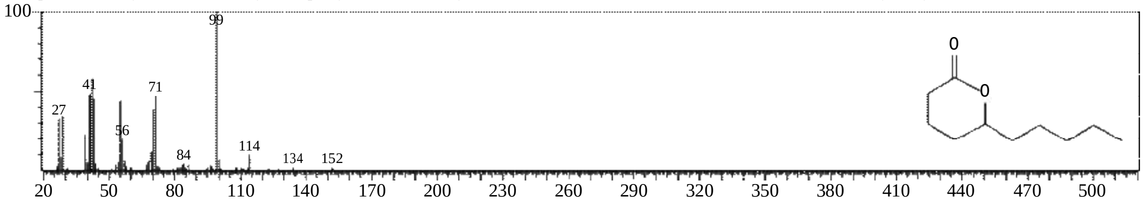

Hit#:4 Entry:14048 Library:NIST05s.LIB
SI:84 Formula:C11H20O2 CAS:710-04-3 MolWeight:184 RetIndex:1503
CompName:2H-Pyran-2-one, 6-hexyltetrahydro- $$ .delta.-Hexylvalerolactone $$ .delta.-Hexyl-.delta.-valerolactone $$ .delta.-Undecalactone $$ 5-Hydroxyunde

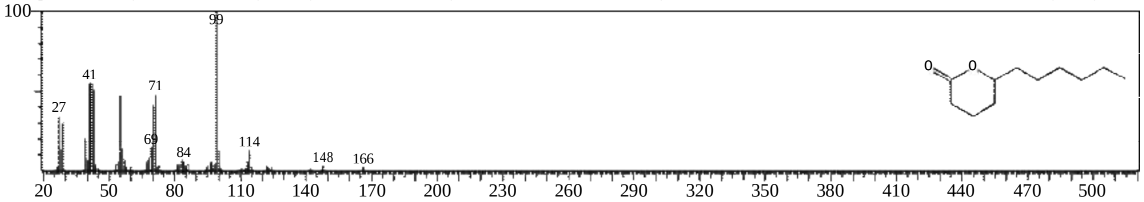

Hit#:5 Entry:15999 Library:NIST05s.LIB
SI:84 Formula:C12H22O2 CAS:713-95-1 MolWeight:198 RetIndex:1602
CompName:2H-Pyran-2-one, 6-heptyltetrahydro- $$ .delta.-Dodecalactone $$ .delta.-Heptyl-.delta.-valerolactone $$ Dodecanoic acid, 5-hydroxy-, .delta.-lacton

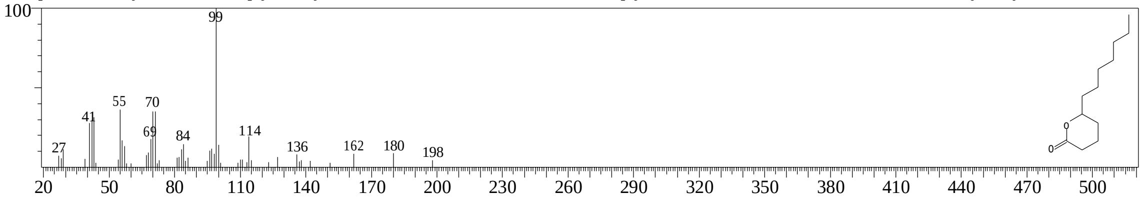

<< Target >>
Line#:31 R.Time:14.817(Scan#:1419) MassPeaks:185
RawMode:Averaged 14.808-14.825(1418-1420) BasePeak:73.05(232988)
BG Mode:Calc. from Peak Group 1 - Event 1

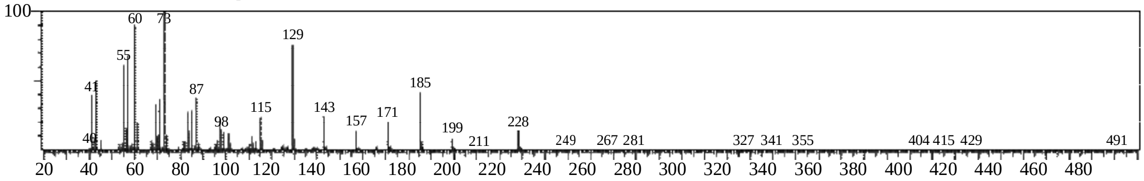

Hit#:1 Entry:19252 Library:NIST05s.LIB
SI:93 Formula:C14H28O2 CAS:544-63-8 MolWeight:228 RetIndex:1769
CompName:Tetradecanoic acid $$ Myristic acid $$ n-Tetradecanoic acid $$ n-Tetradecoic acid $$ Neo-Fat 14 $$ Univol U 316S $$ 1-Tridecanecarboxylic acid

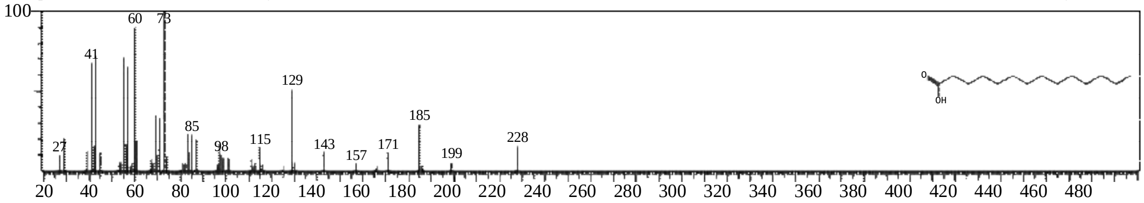

Hit#:2 Entry:20371 Library:NIST05s.LIB
SI:91 Formula:C15H30O2 CAS:1002-84-2 MolWeight:242 RetIndex:1869
CompName:Pentadecanoic acid $$ Pentadecylic acid $$ n-Pentadecanoic acid $$ n-Pentadecylic acid $$

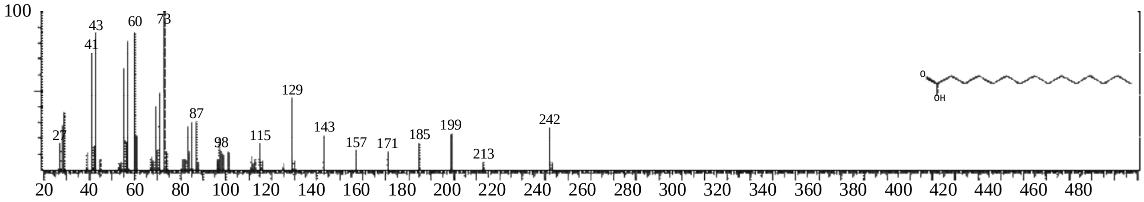

Hit#:3 Entry:19251 Library:NIST05s.LIB
SI:91 Formula:C14H28O2 CAS:544-63-8 MolWeight:228 RetIndex:1769
CompName:Tetradecanoic acid $$ Myristic acid $$ n-Tetradecanoic acid $$ n-Tetradecoic acid $$ Neo-Fat 14 $$ Univol U 316S $$ 1-Tridecanecarboxylic acid

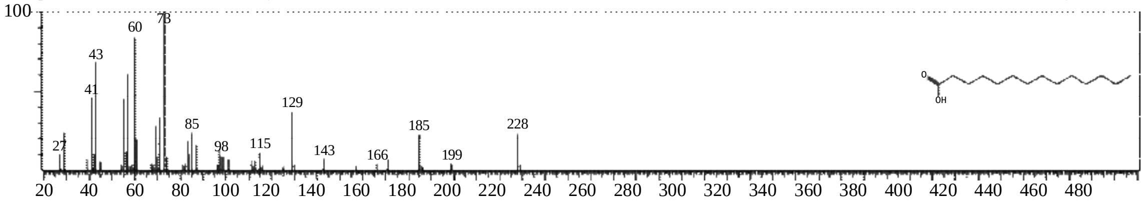

Hit#:4 Entry:17905 Library:NIST05s.LIB
SI:91 Formula:C13H26O2 CAS:638-53-9 MolWeight:214 RetIndex:1670
CompName:Tridecanoic acid $$ n-Tridecanoic acid $$ n-Tridecoic acid $$ Tridecylic acid $$

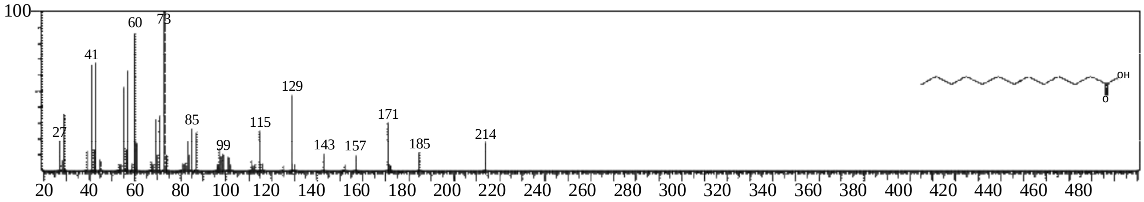

Hit#:5 Entry:58280 Library:NIST05.LIB
SI:90 Formula:C14H28O2 CAS:544-63-8 MolWeight:228 RetIndex:1769
CompName:Tetradecanoic acid $$ Myristic acid $$ n-Tetradecanoic acid $$ n-Tetradecoic acid $$ Neo-Fat 14 $$ Univol U 316S $$ 1-Tridecanecarboxylic acid

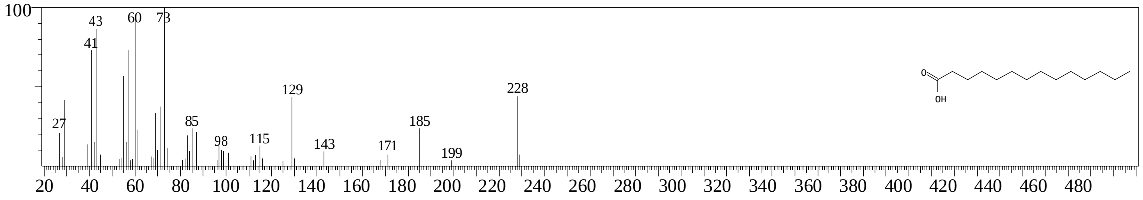

<< Target >>
Line#:32 R.Time:15.408(Scan#:1490) MassPeaks:241
RawMode:Averaged 15.400-15.417(1489-1491) BasePeak:73.10(43773)
BG Mode:Calc. from Peak Group 1 - Event 1

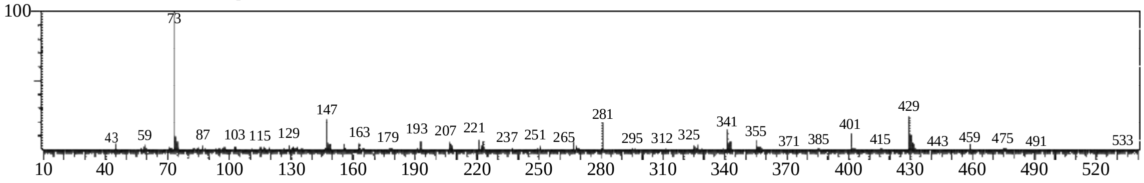

Hit#:1 Entry:160407 Library:NIST05.LIB
SI:77 Formula:C16H50O7Si8 CAS:19095-24-0 MolWeight:578 RetIndex:1710
CompName:Octasiloxane, 1,1,3,3,5,5,7,7,9,9,11,11,13,13,15,15-hexadecamethyl- $$ 1,1,3,3,5,5,7,7,9,9,11,11,13,13,15,15-Hexadecamethyloctasiloxane # $$

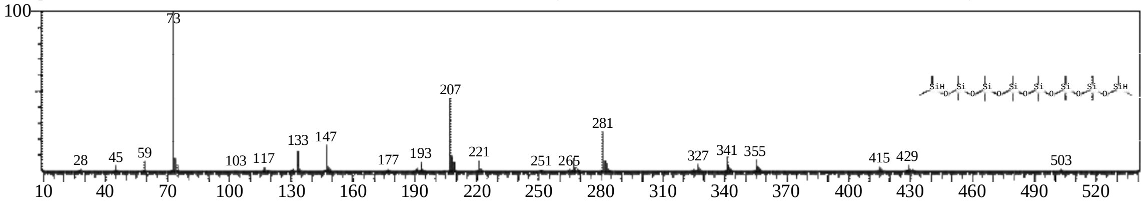

Hit#:2 Entry:152156 Library:NIST05.LIB
SI:77 Formula:C12H36O6Si6 CAS:540-97-6 MolWeight:444 RetIndex:1240
CompName:Cyclohexasiloxane, dodecamethyl- $$ Dodecamethylcyclohexasiloxane $$ 2,2,4,4,6,6,8,8,10,10,12,12-Dodecamethylcyclohexasiloxane # $$

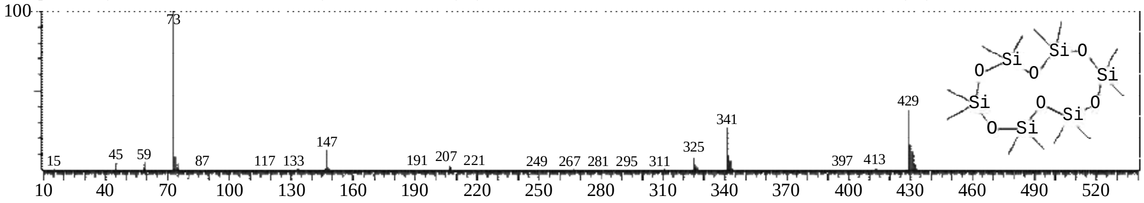

Hit#:3 Entry:27002 Library:NIST05s.LIB
SI:76 Formula:C12H36O6Si6 CAS:540-97-6 MolWeight:444 RetIndex:1240
CompName:Cyclohexasiloxane, dodecamethyl- $$ Dodecamethylcyclohexasiloxane $$ 2,2,4,4,6,6,8,8,10,10,12,12-Dodecamethylcyclohexasiloxane # $$

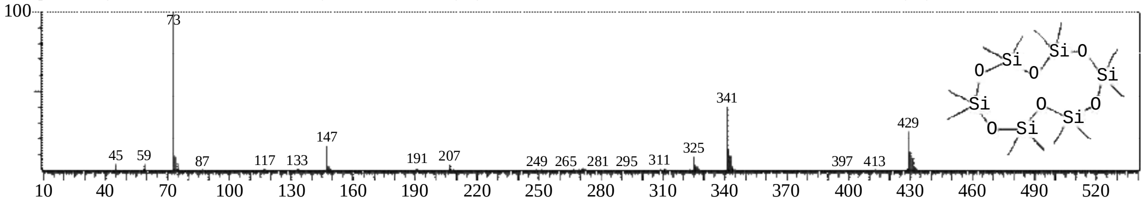

Hit#:4 Entry:157431 Library:NIST05.LIB
SI:76 Formula:C14H44O6Si7 CAS:19095-23-9 MolWeight:504 RetIndex:1526
CompName:Heptasiloxane, 1,1,3,3,5,5,7,7,9,9,11,11,13,13-tetradecamethyl- $$ 1,1,3,3,5,5,7,7,9,9,11,11,13,13-Tetradecamethylheptasiloxane # $$

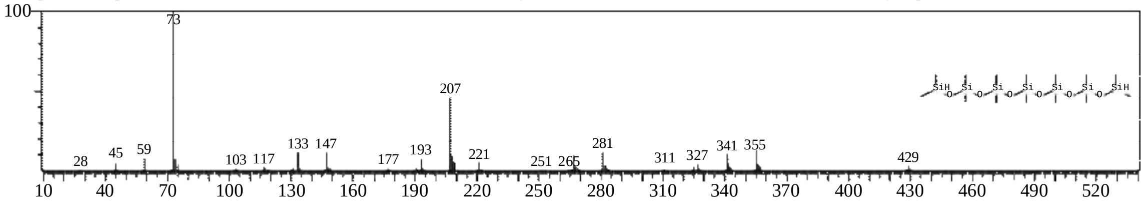

Hit#:5 Entry:156530 Library:NIST05.LIB
SI:73 Formula:C13H42O6Si7 CAS:50694-26-3 MolWeight:490 RetIndex:1570
CompName:1,1,1,3,5,7,9,11,11,11-Decamethyl-5-(trimethylsiloxy)hexasiloxane $$ 1,1,1,3,5,7,9,11,11,11-Decamethyl-5-[(trimethylsilyl)oxy]hexasiloxane # $$

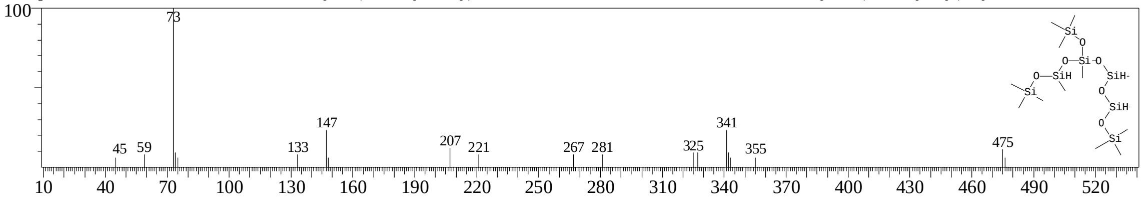

<< Target >>
Line#:33 R.Time:15.950(Scan#:1555) MassPeaks:139
RawMode:Averaged 15.942-15.958(1554-1556) BasePeak:73.10(6877)
BG Mode:Calc. from Peak Group 1 - Event 1

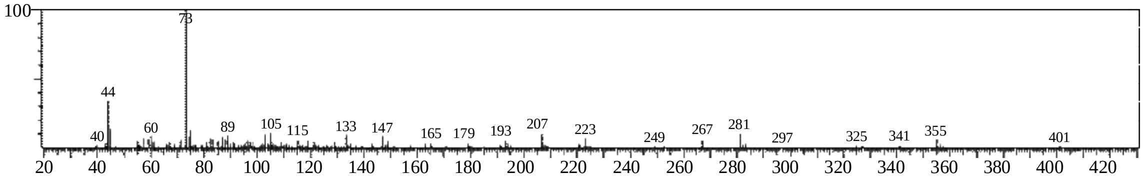

Hit#:1 Entry:19490 Library:NIST05.LIB
SI:66 Formula:C8H20OSi CAS:18246-52-1 MolWeight:160 RetIndex:802
CompName:Silane, (butoxymethyl)trimethyl- $$ (Butoxymethyl)(trimethyl)silane # $$

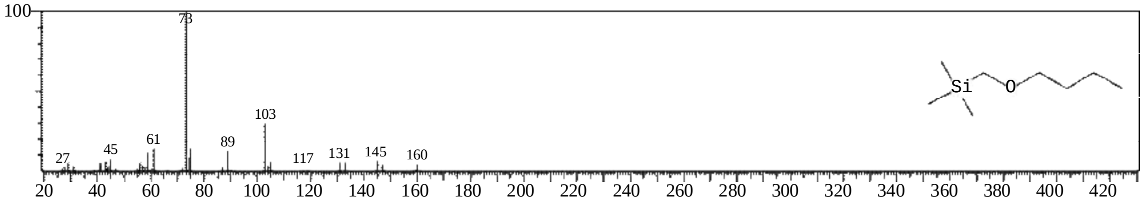

Hit#:2 Entry:13626 Library:NIST05s.LIB
SI:66 Formula:C6H14O6 CAS:608-66-2 MolWeight:182 RetIndex:1752
CompName:Galactitol $$ D-Dulcitol $$ Dulcite $$ Dulcitol $$ Dulcose $$ DAG $$ Euonymit $$ Melampyrin $$ Melampyrit $$ Melampyrite $$ Melampyrum $

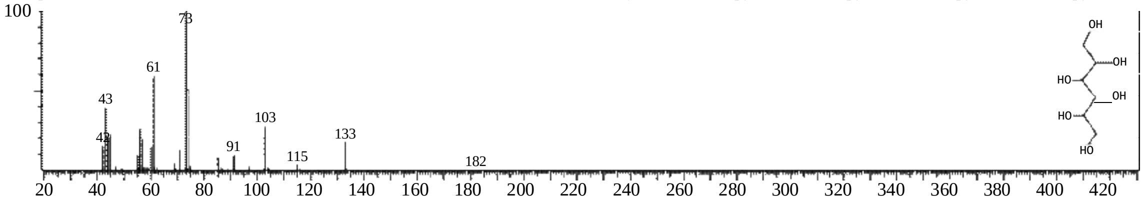

Hit#:3 Entry:19670 Library:NIST05s.LIB
SI:66 Formula:C9H22O3Si2 CAS:17877-42-8 MolWeight:234 RetIndex:1024
CompName:3,7-Dioxa-2,8-disilanonan-5-one, 2,2,8,8-tetramethyl- $$ 2-Propanone, bis(trimethylsilyloxy)- $$ Bis(trimethylsiloxy)acetone $$ 3,7-Dioxa-2,8-dis

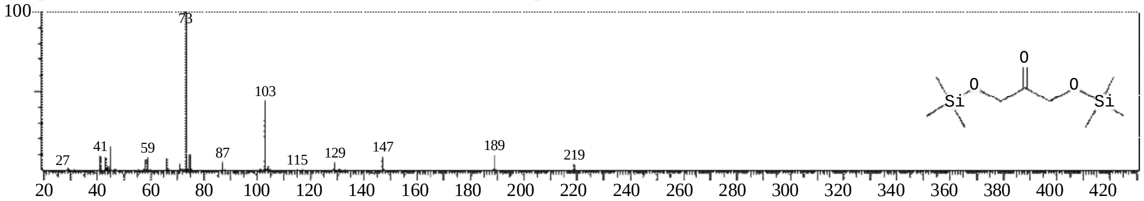

Hit#:4 Entry:28359 Library:NIST05.LIB
SI:66 Formula:C6H10O6 CAS:0-00-0 MolWeight:178 RetIndex:1813
CompName:d-Talonic acid lactone

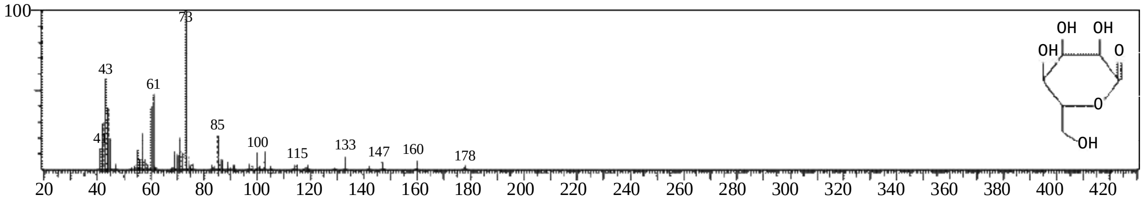

Hit#:5 Entry:107466 Library:NIST05.LIB
SI:66 Formula:C11H28O2S2Si2 CAS:0-00-0 MolWeight:312 RetIndex:1588
CompName:Bis(2-trimethylsiloxyethylthio)methane

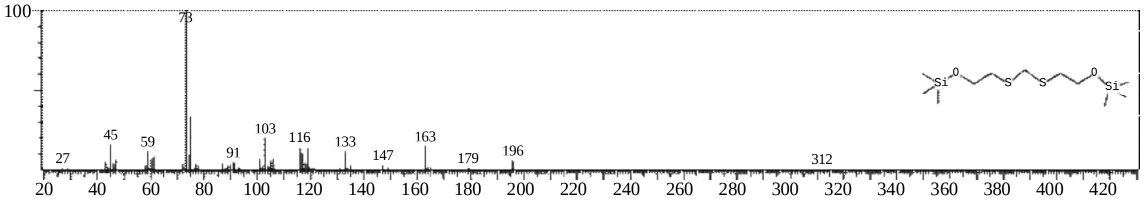

<< Target >>
Line#:34 R.Time:16.192(Scan#:1584) MassPeaks:116
RawMode:Averaged 16.183-16.200(1583-1585) BasePeak:57.10(10319)
BG Mode:Calc. from Peak Group 1 - Event 1

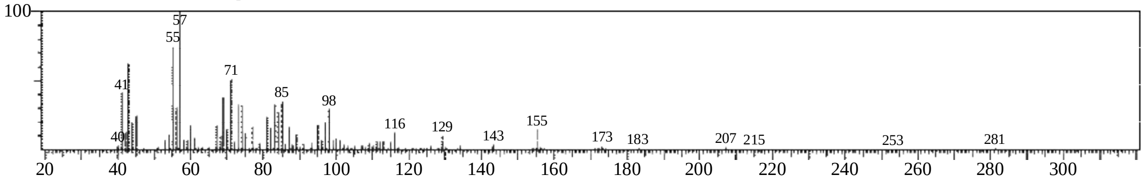

Hit#:1 Entry:160165 Library:NIST05.LIB
SI:83 Formula:C35H68O5 CAS:761-35-3 MolWeight:568 RetIndex:4013
CompName:Hexadecanoic acid, 1-(hydroxymethyl)-1,2-ethanediyl ester $$ Palmitin, 1,2-di- $$ Dipalmitin $$ Glycerol 1,2-dipalmitate $$ 1,2-Dipalmitin $$ 1,2

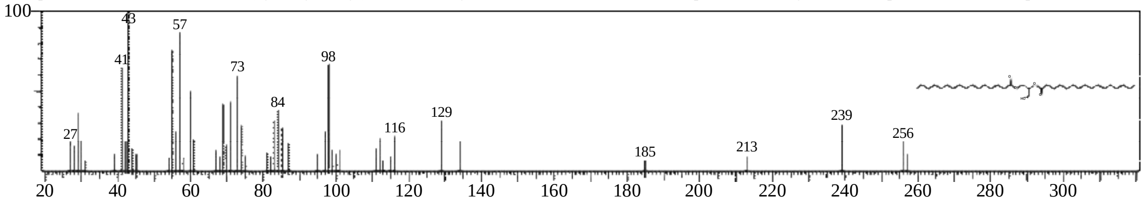

Hit#:2 Entry:140926 Library:NIST05.LIB
SI:83 Formula:C23H46O4 CAS:55334-78-6 MolWeight:386 RetIndex:2896
CompName:Eicosanoic acid, 2-hydroxy-1-(hydroxymethyl)ethyl ester $$ 2-Hydroxy-1-(hydroxymethyl)ethyl icosanoate # $$

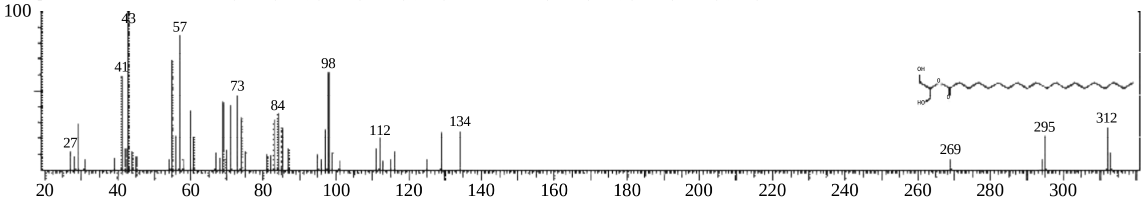

Hit#:3 Entry:100059 Library:NIST05.LIB
SI:83 Formula:C19H38O2 CAS:15965-99-8 MolWeight:298 RetIndex:2076
CompName:Oxirane, [(hexadecyloxy)methyl]- $$ Propane, 1,2-epoxy-3-(hexadecyloxy)- $$ (Cetyloxymethyl)oxirane $$ Cetyl glycidyl ether $$ Glycidol .alpha.

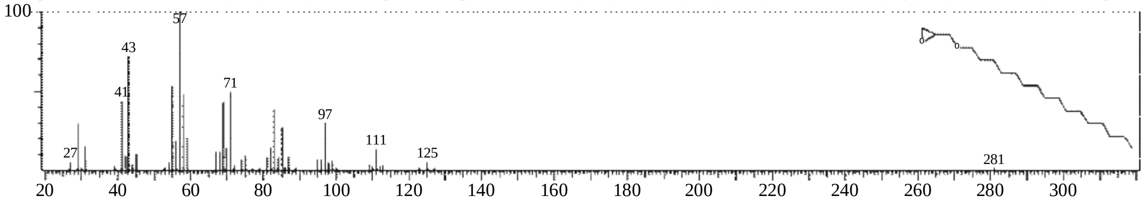

Hit#:4 Entry:110136 Library:NIST05.LIB
SI:83 Formula:C18H36O4 CAS:98863-01-5 MolWeight:316 RetIndex:2399
CompName:Pentadecanoic acid, 2-hydroxy-1-(hydroxymethyl)ethyl ester $$ 2-Hydroxy-1-(hydroxymethyl)ethyl pentadecanoate # $$

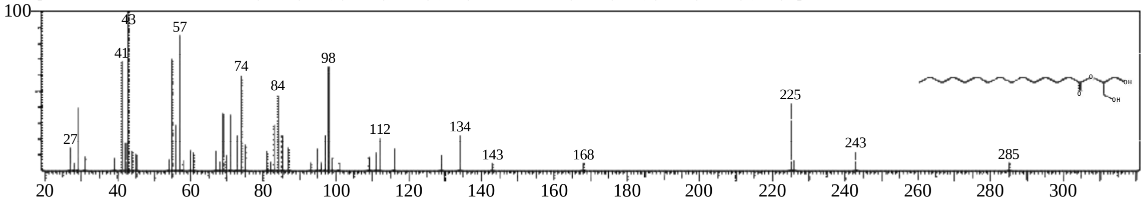

Hit#:5 Entry:27447 Library:NIST05s.LIB
SI:82 Formula:C35H68O5 CAS:761-35-3 MolWeight:568 RetIndex:4013
CompName:Hexadecanoic acid, 1-(hydroxymethyl)-1,2-ethanediyl ester $$ Palmitin, 1,2-di- $$ Dipalmitin $$ Glycerol 1,2-dipalmitate $$ 1,2-Dipalmitin $$ 1,2

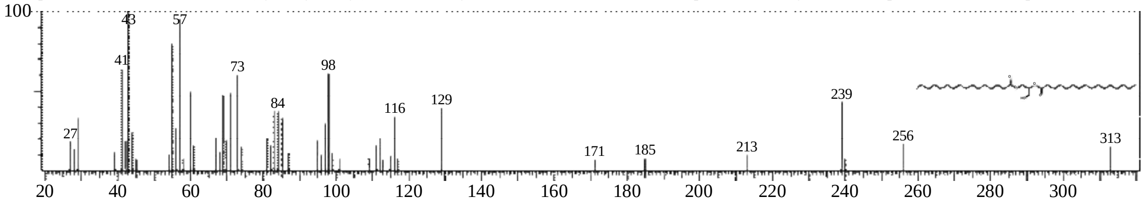

<< Target >>
Line#:35 R.Time:16.500(Scan#:1621) MassPeaks:94
RawMode:Averaged 16.492-16.508(1620-1622) BasePeak:74.10(5860)
BG Mode:Calc. from Peak Group 1 - Event 1

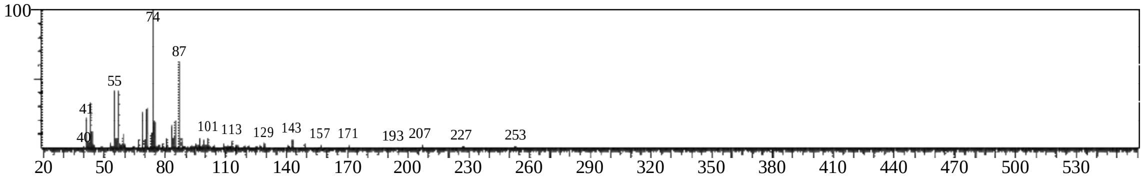

Hit#:1 Entry:24289 Library:NIST05s.LIB
SI:86 Formula:C20H40O2 CAS:2490-19-9 MolWeight:312 RetIndex:2112
CompName:Octadecanoic acid, 10-methyl-, methyl ester $$ Methyl 10-methyloctadecanoate # $$

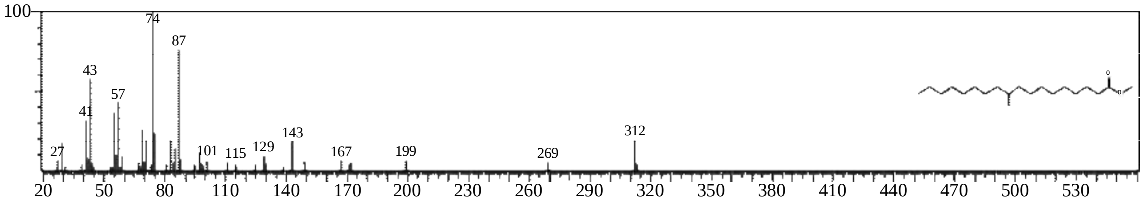

Hit#:2 Entry:26833 Library:NIST05s.LIB
SI:86 Formula:C28H56O2 CAS:55682-91-2 MolWeight:424 RetIndex:2972
CompName:Heptacosanoic acid, methyl ester $$ Methyl heptacosanoate # $$

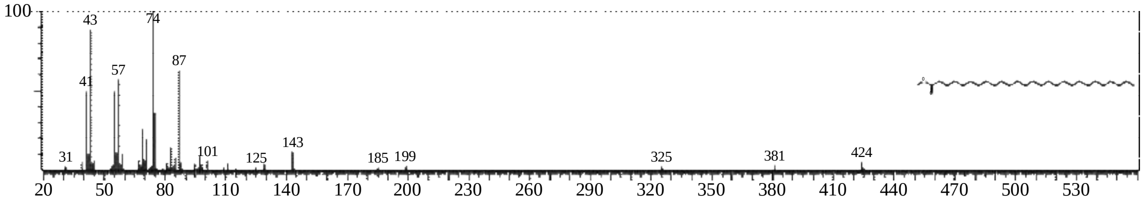

Hit#:3 Entry:27154 Library:NIST05s.LIB
SI:85 Formula:C31H62O2 CAS:629-83-4 MolWeight:466 RetIndex:3270
CompName:Triacontanoic acid, methyl ester $$ Methyl melissate $$ Methyl melissicate $$ Methyl triacontanoate $$

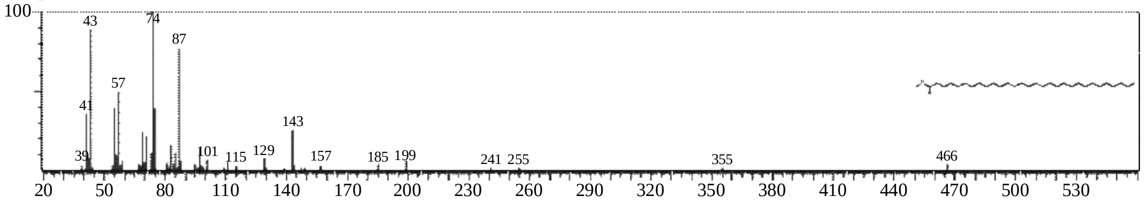

Hit#:4 Entry:26186 Library:NIST05s.LIB
SI:85 Formula:C25H50O2 CAS:2442-49-1 MolWeight:382 RetIndex:2674
CompName:Tetracosanoic acid, methyl ester $$ Methyl lignocerate $$ Methyl tetracosanoate $$ Lignoceric acid methyl ester $$

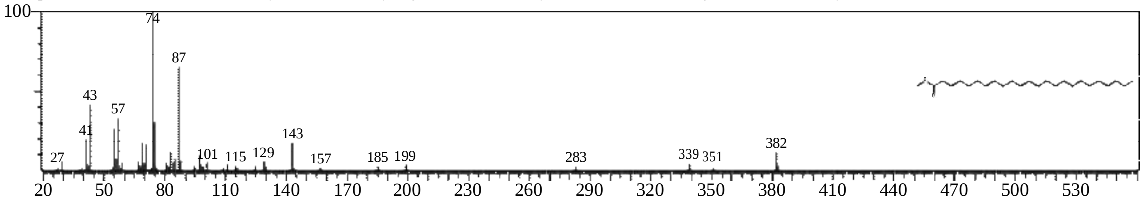

Hit#:5 Entry:49562 Library:NIST05.LIB
SI:85 Formula:C13H26O2 CAS:5129-56-6 MolWeight:214 RetIndex:1417
CompName:Undecanoic acid, 10-methyl-, methyl ester $$ Methyl 10-methylundecanoate # $$

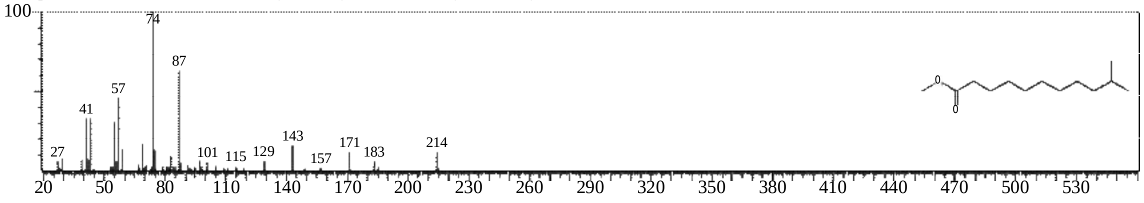

<< Target >>
Line#:36 R.Time:16.633(Scan#:1637) MassPeaks:92
RawMode:Averaged 16.625-16.642(1636-1638) BasePeak:99.10(3450)
BG Mode:Calc. from Peak Group 1 - Event 1

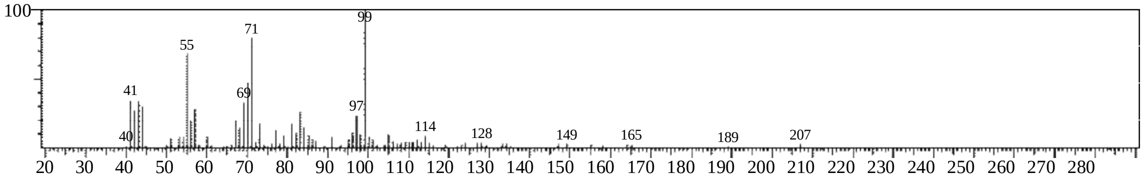

Hit#:1 Entry:90609 Library:NIST05.LIB
SI:83 Formula:C18H34O2 CAS:1227-51-6 MolWeight:282 RetIndex:2199
CompName:2H-Pyran-2-one, tetrahydro-6-tridecyl- $$ Octadecanoic acid, 5-hydroxy-, .delta.-lactone $$ 6-Tridecyltetrahydro-2H-pyran-2-one # $$

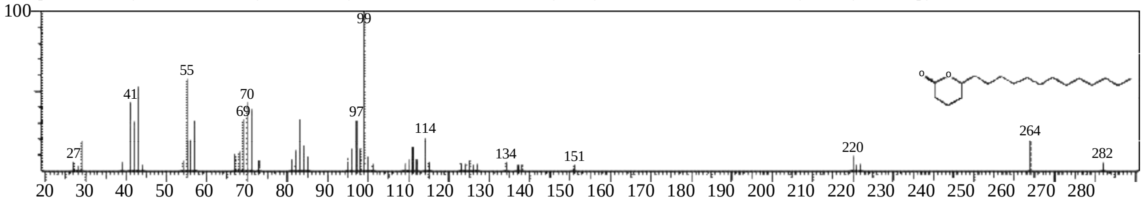

Hit#:2 Entry:14050 Library:NIST05s.LIB
SI:83 Formula:C11H20O2 CAS:710-04-3 MolWeight:184 RetIndex:1503
CompName:2H-Pyran-2-one, 6-hexyltetrahydro- $$ .delta.-Hexylvalerolactone $$ .delta.-Hexyl-.delta.-valerolactone $$ .delta.-Undecalactone $$ 5-Hydroxyunde

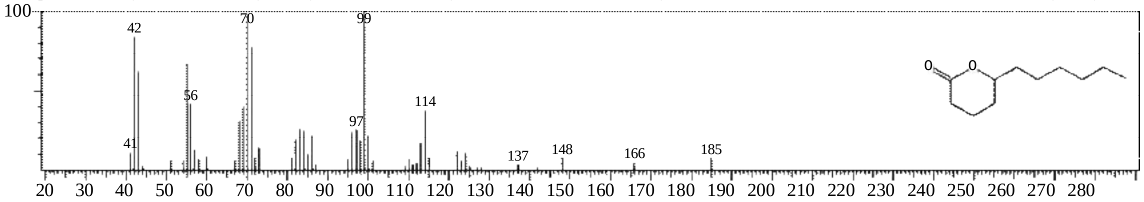

Hit#:3 Entry:9696 Library:NIST05s.LIB
SI:82 Formula:C9H16O2 CAS:3301-94-8 MolWeight:156 RetIndex:1304
CompName:.delta. Nonalactone $$ 2H-Pyran-2-one, 6-butyltetrahydro- $$ 2H-Pyran-2-one, tetrahydro-6-butyl $$ .beta.-Nonalactone $$ 6-Butyltetrahydro-2H-p

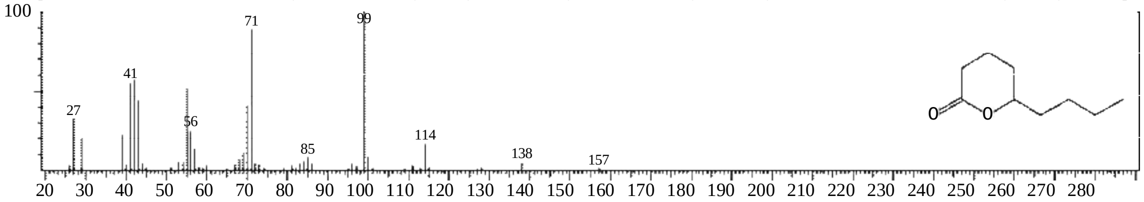

Hit#:4 Entry:15996 Library:NIST05s.LIB
SI:82 Formula:C12H22O2 CAS:713-95-1 MolWeight:198 RetIndex:1602
CompName:2H-Pyran-2-one, 6-heptyltetrahydro- $$ .delta.-Dodecalactone $$ .delta.-Heptyl-.delta.-valerolactone $$ Dodecanoic acid, 5-hydroxy-, .delta.-lacton

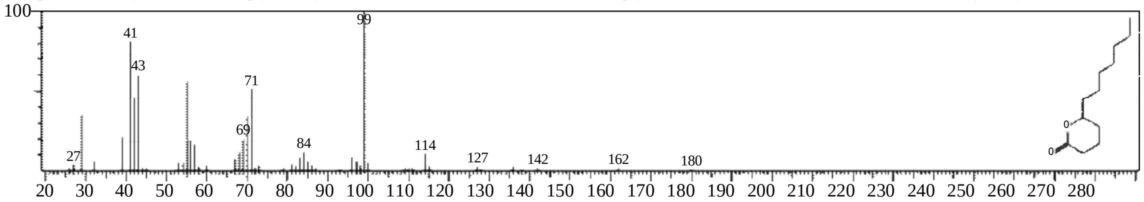

Hit#:5 Entry:9662 Library:NIST05s.LIB
SI:81 Formula:C9H16O2 CAS:3301-94-8 MolWeight:156 RetIndex:1304
CompName:.delta. Nonalactone $$ 2H-Pyran-2-one, 6-butyltetrahydro- $$ 2H-Pyran-2-one, tetrahydro-6-butyl $$ .beta.-Nonalactone $$ 6-Butyltetrahydro-2H-p

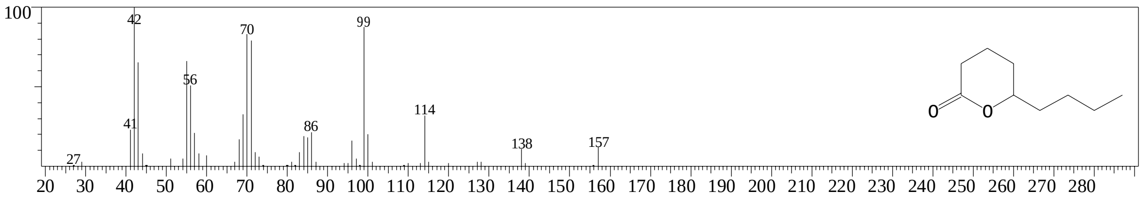

<< Target >>
Line#:37 R.Time:16.858(Scan#:1664) MassPeaks:180
RawMode:Averaged 16.850-16.867(1663-1665) BasePeak:73.10(60247)
BG Mode:Calc. from Peak Group 1 - Event 1

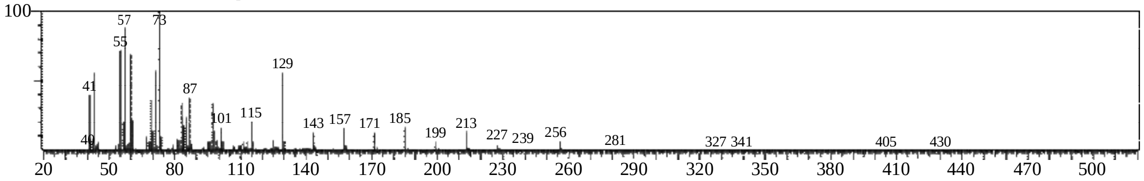

Hit#:1 Entry:20371 Library:NIST05s.LIB
SI:91 Formula:C15H30O2 CAS:1002-84-2 MolWeight:242 RetIndex:1869
CompName:Pentadecanoic acid $$ Pentadecylic acid $$ n-Pentadecanoic acid $$ n-Pentadecylic acid $$

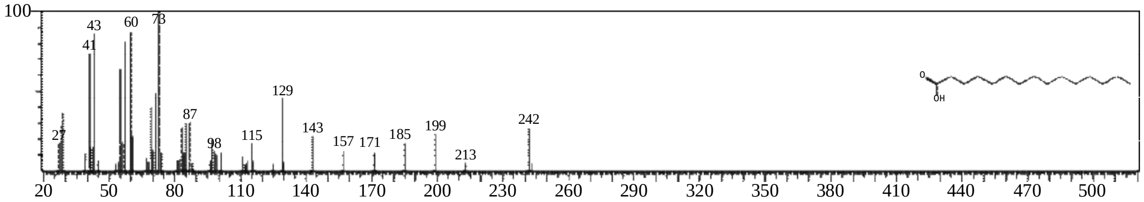

Hit#:2 Entry:161860 Library:NIST05.LIB
SI:90 Formula:C38H68O8 CAS:28474-90-0 MolWeight:652 RetIndex:4765
CompName:l-(+)-Ascorbic acid 2,6-dihexadecanoate

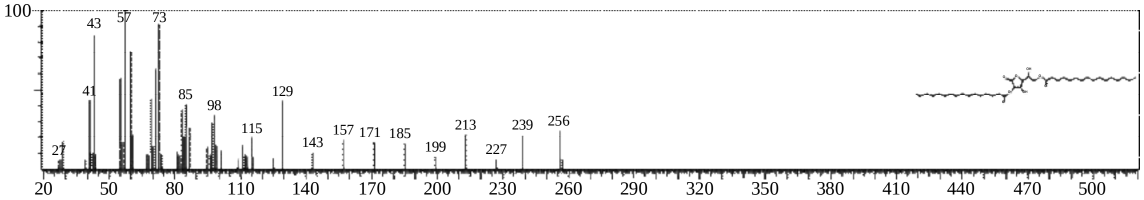

Hit#:3 Entry:19252 Library:NIST05s.LIB
SI:90 Formula:C14H28O2 CAS:544-63-8 MolWeight:228 RetIndex:1769
CompName:Tetradecanoic acid $$ Myristic acid $$ n-Tetradecanoic acid $$ n-Tetradecoic acid $$ Neo-Fat 14 $$ Univol U 316S $$ 1-Tridecanecarboxylic acid

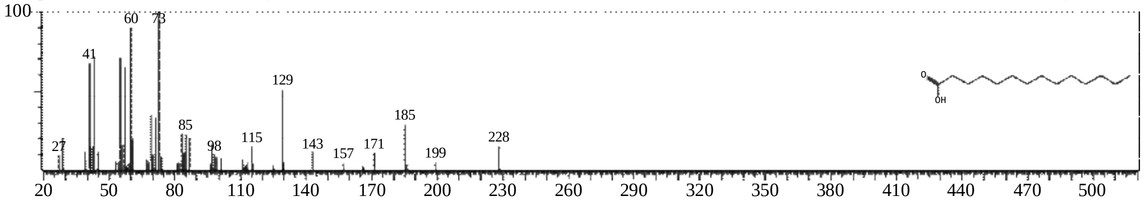

Hit#:4 Entry:22979 Library:NIST05s.LIB
SI:90 Formula:C18H36O2 CAS:57-11-4 MolWeight:284 RetIndex:2167
CompName:Octadecanoic acid $$ Stearic acid $$ n-Octadecanoic acid $$ Humko Industrene R $$ Hydrofol Acid 150 $$ Hystrene S-97 $$ Hystrene T-70 $$ Hys

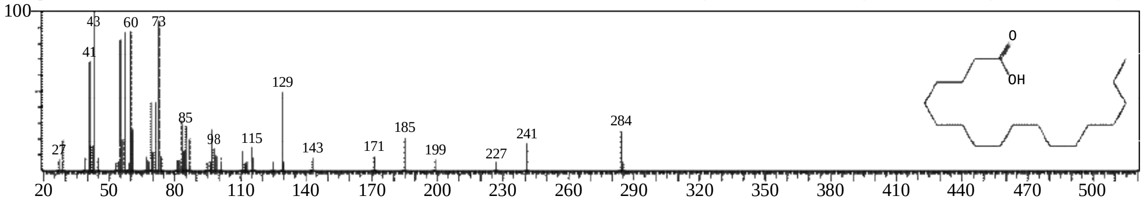

Hit#:5 Entry:21331 Library:NIST05s.LIB
SI:89 Formula:C16H32O2 CAS:57-10-3 MolWeight:256 RetIndex:1968
CompName:n-Hexadecanoic acid $$ Hexadecanoic acid $$ n-Hexadecoic acid $$ Palmitic acid $$ Pentadecanecarboxylic acid $$ 1-Pentadecanecarboxylic acid

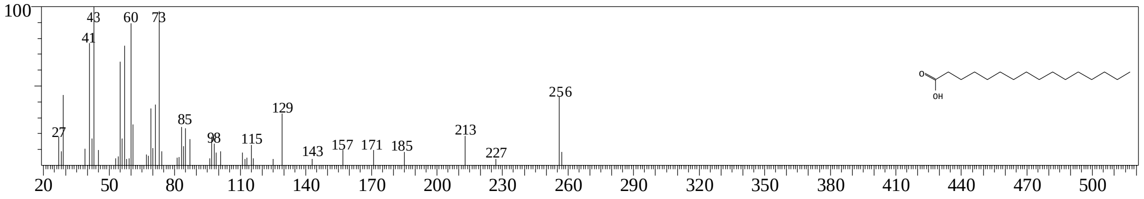

<< Target >>
Line#:38 R.Time:16.967(Scan#:1677) MassPeaks:212
RawMode:Averaged 16.958-16.975(1676-1678) BasePeak:73.10(35319)
BG Mode:Calc. from Peak Group 1 - Event 1

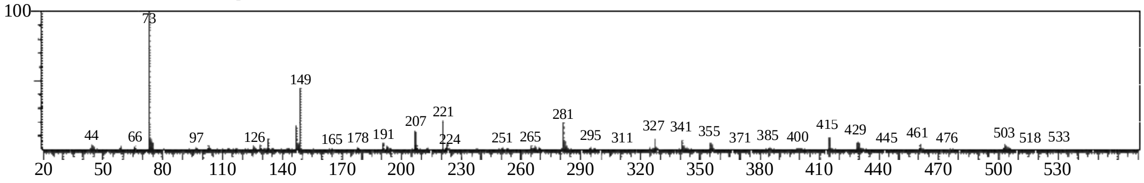

Hit#:1 Entry:160407 Library:NIST05.LIB
SI:81 Formula:C16H50O7Si8 CAS:19095-24-0 MolWeight:578 RetIndex:1710
CompName:Octasiloxane, 1,1,3,3,5,5,7,7,9,9,11,11,13,13,15,15-hexadecamethyl- $$ 1,1,3,3,5,5,7,7,9,9,11,11,13,13,15,15-Hexadecamethyloctasiloxane # $$

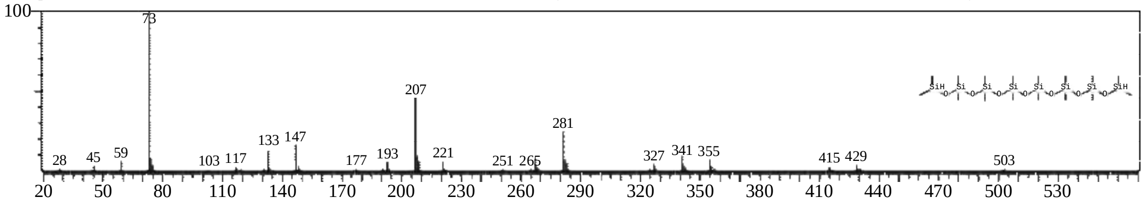

Hit#:2 Entry:157431 Library:NIST05.LIB
SI:77 Formula:C14H44O6Si7 CAS:19095-23-9 MolWeight:504 RetIndex:1526
CompName:Heptasiloxane, 1,1,3,3,5,5,7,7,9,9,11,11,13,13-tetradecamethyl- $$ 1,1,3,3,5,5,7,7,9,9,11,11,13,13-Tetradecamethylheptasiloxane # $$

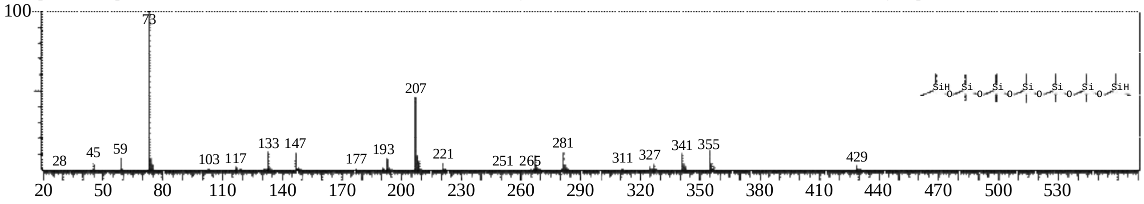

Hit#:3 Entry:160345 Library:NIST05.LIB
SI:76 Formula:C18H52O7Si7 CAS:71579-69-6 MolWeight:576 RetIndex:1648
CompName:3-Isopropoxy-1,1,1,7,7,7-hexamethyl-3,5,5-tris(trimethylsiloxy)tetrasiloxane $$ 1-Isopropoxy-3,3,3-trimethyl-1-[(trimethylsilyl)oxy]disiloxanyl tris

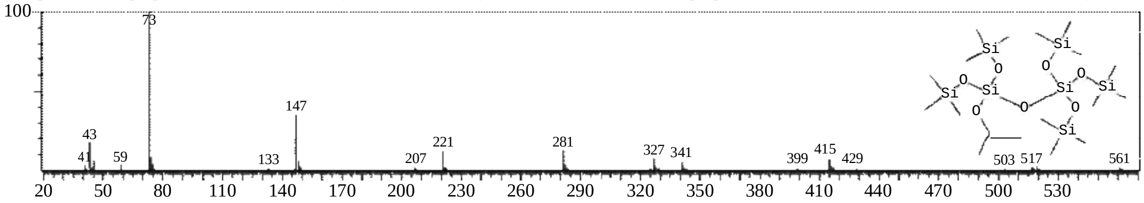

Hit#:4 Entry:150228 Library:NIST05.LIB
SI:76 Formula:C12H38O5Si6 CAS:995-82-4 MolWeight:430 RetIndex:1341
CompName:Hexasiloxane, 1,1,3,3,5,5,7,7,9,9,11,11-dodecamethyl- $$ 1,1,3,3,5,5,7,7,9,9,11,11-Dodecamethylhexasiloxane # $$

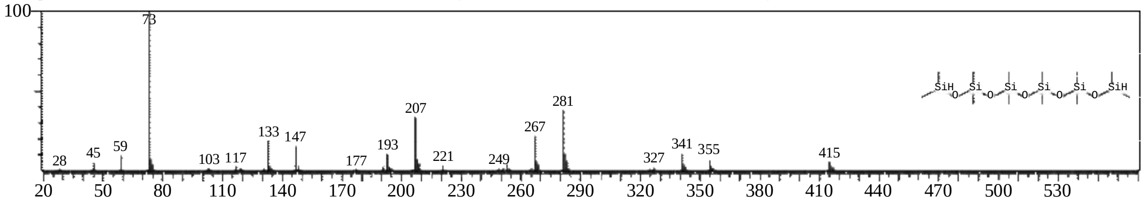

Hit#:5 Entry:158804 Library:NIST05.LIB
SI:72 Formula:C16H48O6Si7 CAS:541-01-5 MolWeight:532 RetIndex:1437
CompName:Heptasiloxane, hexadecamethyl- $$ Hexadecamethylheptasiloxane $$ 1,1,1,3,3,5,5,7,7,9,9,11,11,13,13,13-Hexadecamethylheptasiloxane # $$

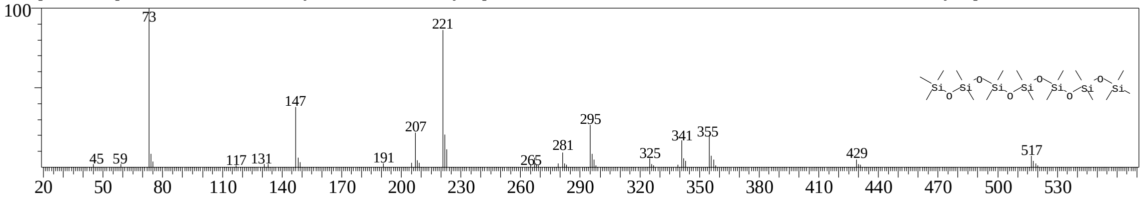

<< Target >>
Line#:39 R.Time:17.483(Scan#:1739) MassPeaks:153
RawMode:Averaged 17.475-17.492(1738-1740) BasePeak:73.05(6101)
BG Mode:Calc. from Peak Group 1 - Event 1

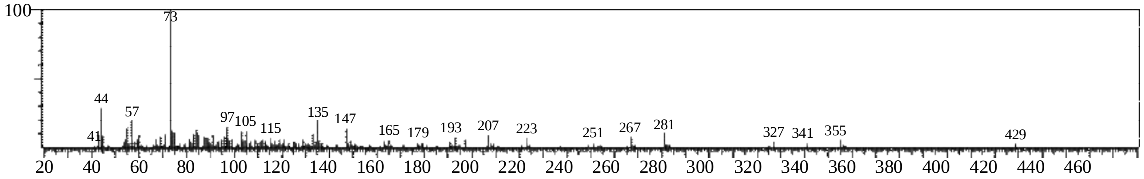

Hit#:1 Entry:130518 Library:NIST05.LIB
SI:68 Formula:C19H34O6 CAS:55191-44-1 MolWeight:358 RetIndex:2348
CompName:Dodecanoic acid, 2,3-bis(acetyloxy)propyl ester $$ 2,3-Bis(acetyloxy)propyl laurate # $$

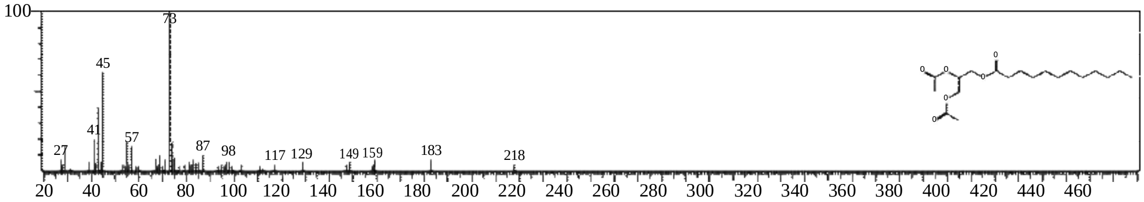

Hit#:2 Entry:143255 Library:NIST05.LIB
SI:67 Formula:C26H50O2 CAS:52355-42-7 MolWeight:394 RetIndex:2737
CompName:Cyclopropanetetradecanoic acid, 2-octyl-, methyl ester $$ Methyl 14-(2-octylcyclopropyl)tetradecanoate # $$

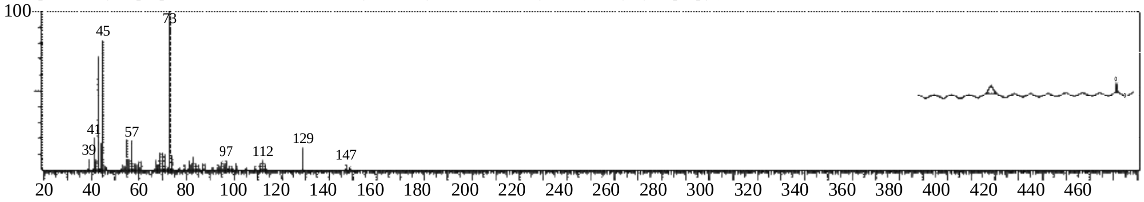

Hit#:3 Entry:130517 Library:NIST05.LIB
SI:65 Formula:C19H34O6 CAS:55191-43-0 MolWeight:358 RetIndex:2348
CompName:Dodecanoic acid, 2-(acetyloxy)-1-[(acetyloxy)methyl]ethyl ester $$ 2-(Acetyloxy)-1-[(acetyloxy)methyl]ethyl laurate # $$

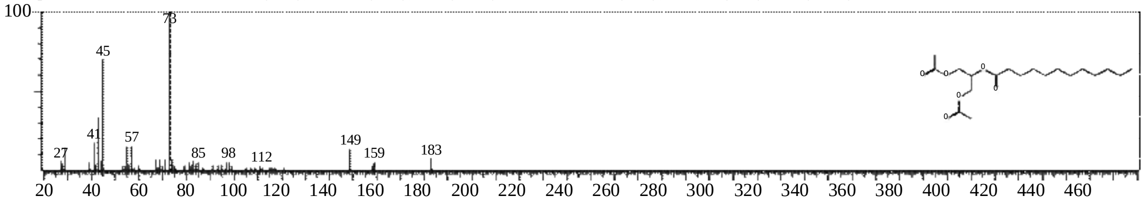

Hit#:4 Entry:37128 Library:NIST05.LIB
SI:65 Formula:C7H14O6 CAS:0-00-0 MolWeight:194 RetIndex:1647
CompName:3-O-Methyl-d-glucose

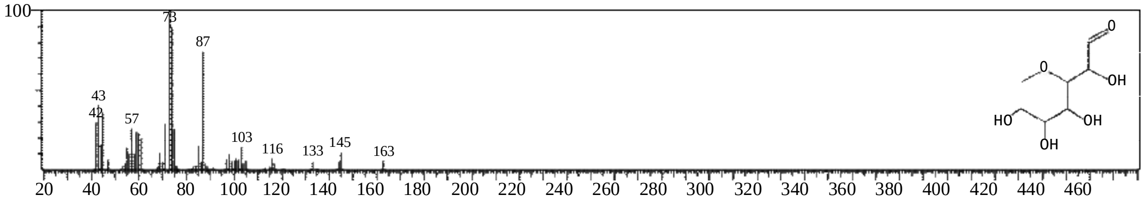

Hit#:5 Entry:129855 Library:NIST05.LIB
SI:65 Formula:C22H44O3 CAS:15337-64-1 MolWeight:356 RetIndex:2618
CompName:Octadecanoic acid, 4-hydroxybutyl ester $$ Stearic acid, 4-hydroxybutyl ester $$ 1,4-Butanediol monostearate $$ 4-Hydroxybutyl stearate # $$

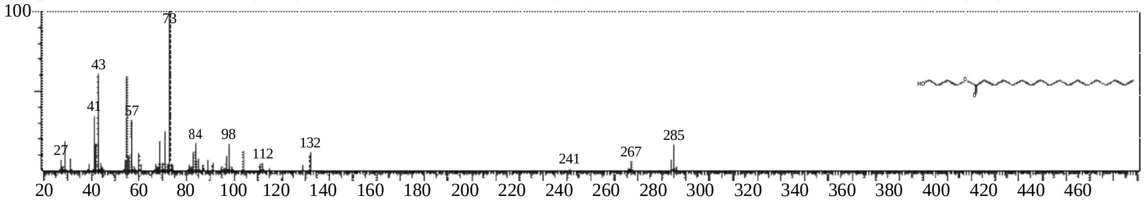

<< Target >>
Line#:40 R.Time:18.192(Scan#:1824) MassPeaks:196
RawMode:Averaged 18.183-18.200(1823-1825) BasePeak:74.05(34184)
BG Mode:Calc. from Peak Group 1 - Event 1

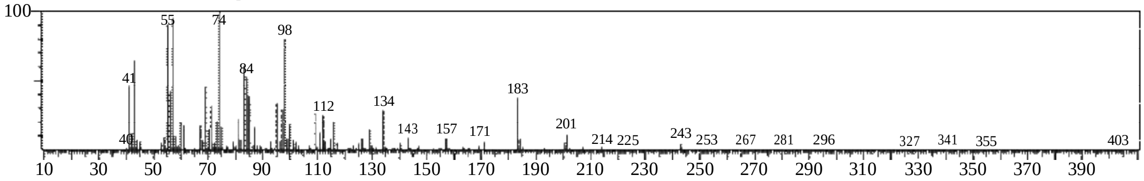

Hit#:1 Entry:22404 Library:NIST05s.LIB
SI:86 Formula:C15H30O4 CAS:142-18-7 MolWeight:274 RetIndex:2085
CompName:Dodecanoic acid, 2,3-dihydroxypropyl ester $$ Laurin, 1-mono- $$ .alpha.-Monolaurin $$ Glycerin 1-monolaurate $$ Glycerol .alpha.-monolaurate

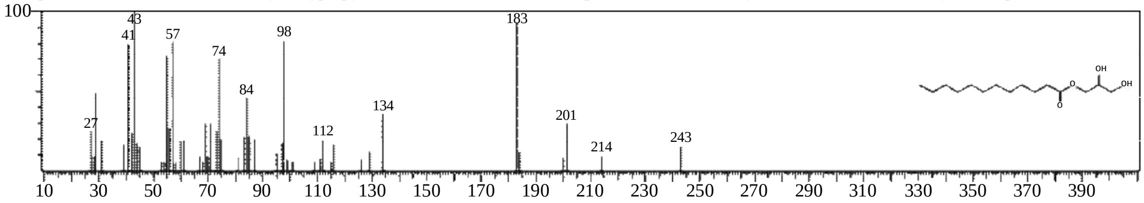

Hit#:2 Entry:85782 Library:NIST05.LIB
SI:84 Formula:C15H30O4 CAS:142-18-7 MolWeight:274 RetIndex:2085
CompName:Dodecanoic acid, 2,3-dihydroxypropyl ester $$ Laurin, 1-mono- $$ .alpha.-Monolaurin $$ Glycerin 1-monolaurate $$ Glycerol .alpha.-monolaurate

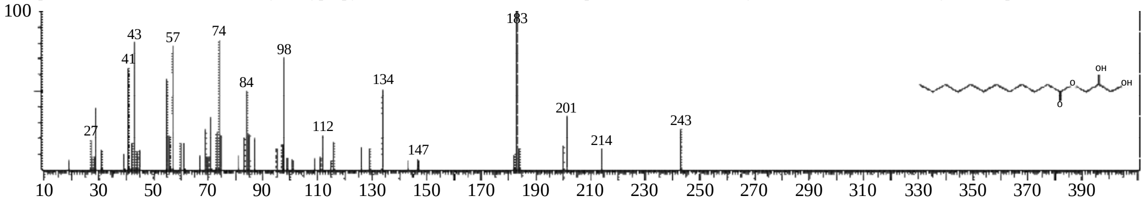

Hit#:3 Entry:110136 Library:NIST05.LIB
SI:84 Formula:C18H36O4 CAS:98863-01-5 MolWeight:316 RetIndex:2399
CompName:Pentadecanoic acid, 2-hydroxy-1-(hydroxymethyl)ethyl ester $$ 2-Hydroxy-1-(hydroxymethyl)ethyl pentadecanoate # $$

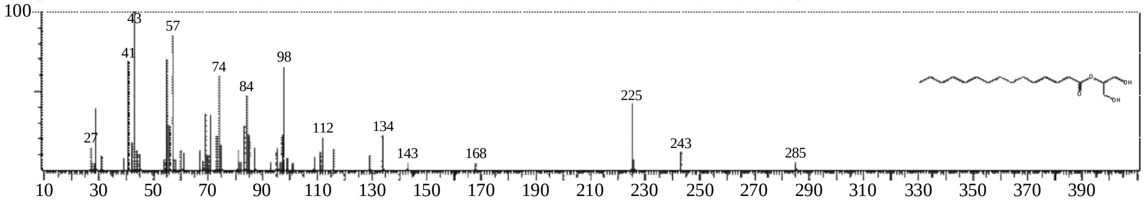

Hit#:4 Entry:117519 Library:NIST05.LIB
SI:84 Formula:C19H38O4 CAS:23470-00-0 MolWeight:330 RetIndex:2498
CompName:Hexadecanoic acid, 2-hydroxy-1-(hydroxymethyl)ethyl ester $$ Palmitin, 2-mono- $$ Palmitic acid .beta.-monoglyceride $$ 2-Hexadecanoyl glycero

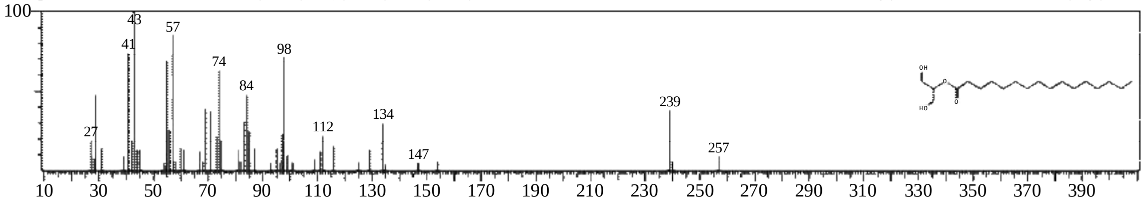

Hit#:5 Entry:85777 Library:NIST05.LIB
SI:83 Formula:C15H30O4 CAS:1678-45-1 MolWeight:274 RetIndex:2101
CompName:Dodecanoic acid, 2-hydroxy-1-(hydroxymethyl)ethyl ester $$ Laurin, 2-mono- $$ .beta.-Monolaurin $$ Glycerol 2-laurate $$ Lauric acid .beta.-mon

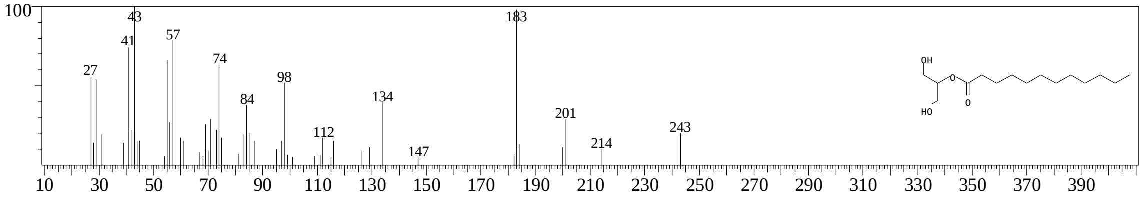

<< Target >>
Line#:41 R.Time:18.375(Scan#:1846) MassPeaks:184
RawMode:Averaged 18.367-18.383(1845-1847) BasePeak:73.05(36085)
BG Mode:Calc. from Peak Group 1 - Event 1

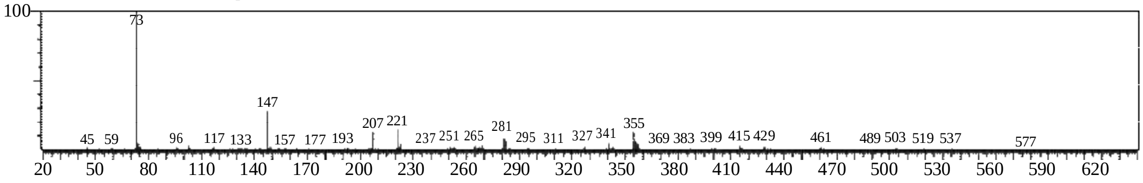

Hit#:1 Entry:160407 Library:NIST05.LIB
SI:81 Formula:C16H50O7Si8 CAS:19095-24-0 MolWeight:578 RetIndex:1710
CompName:Octasiloxane, 1,1,3,3,5,5,7,7,9,9,11,11,13,13,15,15-hexadecamethyl- $$ 1,1,3,3,5,5,7,7,9,9,11,11,13,13,15,15-Hexadecamethyloctasiloxane # $$

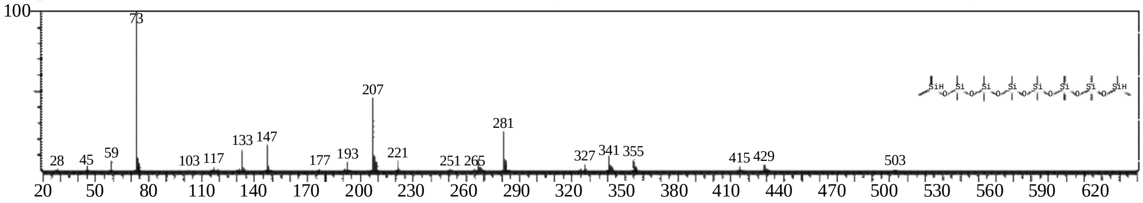

Hit#:2 Entry:157431 Library:NIST05.LIB
SI:79 Formula:C14H44O6Si7 CAS:19095-23-9 MolWeight:504 RetIndex:1526
CompName:Heptasiloxane, 1,1,3,3,5,5,7,7,9,9,11,11,13,13-tetradecamethyl- $$ 1,1,3,3,5,5,7,7,9,9,11,11,13,13-Tetradecamethylheptasiloxane # $$

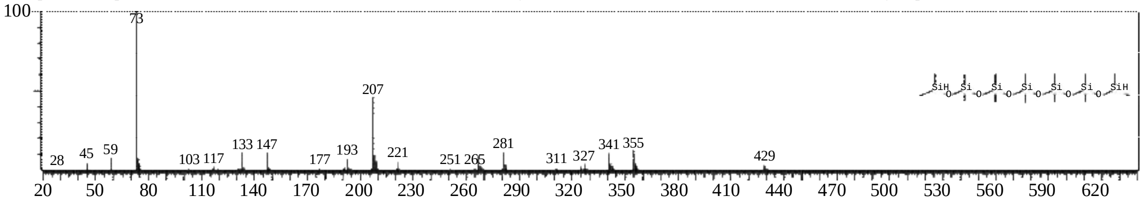

Hit#:3 Entry:161854 Library:NIST05.LIB
SI:79 Formula:C16H48O10Si9 CAS:145344-72-5 MolWeight:652 RetIndex:1716
CompName:2-(2',4',4',6',6',8',8'-Heptamethyltetrasiloxan-2'-yloxy)-2,4,4,6,6,8,8,10,10-nonamethylcyclopentasiloxane $$ 2-[(2,4,4,6,6,8,8-Heptamethyl-1,3,5,7,2

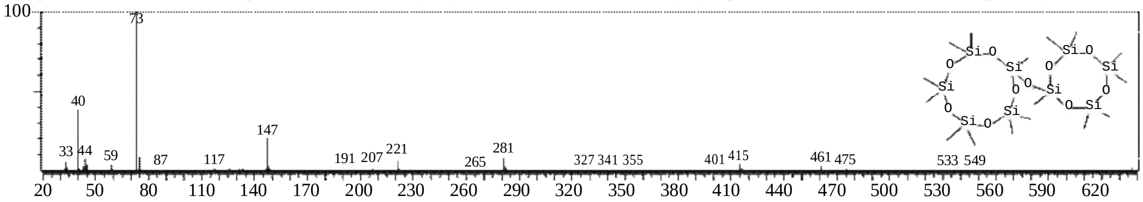

Hit#:4 Entry:160345 Library:NIST05.LIB
SI:78 Formula:C18H52O7Si7 CAS:71579-69-6 MolWeight:576 RetIndex:1648
CompName:3-Isopropoxy-1,1,1,7,7,7-hexamethyl-3,5,5-tris(trimethylsiloxy)tetrasiloxane $$ 1-Isopropoxy-3,3,3-trimethyl-1-[(trimethylsilyl)oxy]disiloxanyl tris

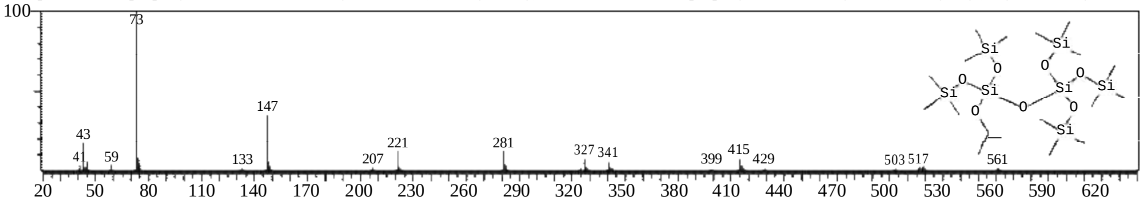

Hit#:5 Entry:150228 Library:NIST05.LIB
SI:78 Formula:C12H38O5Si6 CAS:995-82-4 MolWeight:430 RetIndex:1341
CompName:Hexasiloxane, 1,1,3,3,5,5,7,7,9,9,11,11-dodecamethyl- $$ 1,1,3,3,5,5,7,7,9,9,11,11-Dodecamethylhexasiloxane # $$

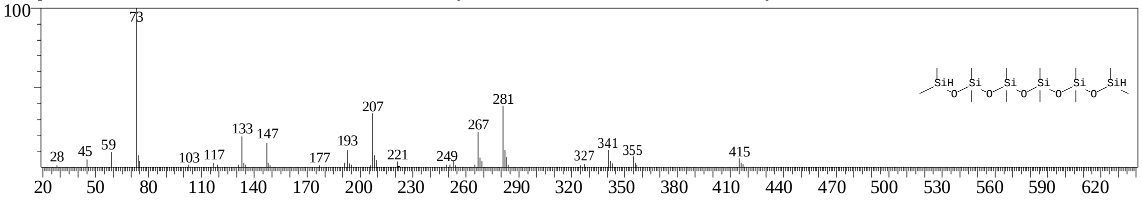

<< Target >>
Line#:42 R.Time:18.558(Scan#:1868) MassPeaks:171
RawMode:Averaged 18.550-18.567(1867-1869) BasePeak:55.05(21361)
BG Mode:Calc. from Peak Group 1 - Event 1

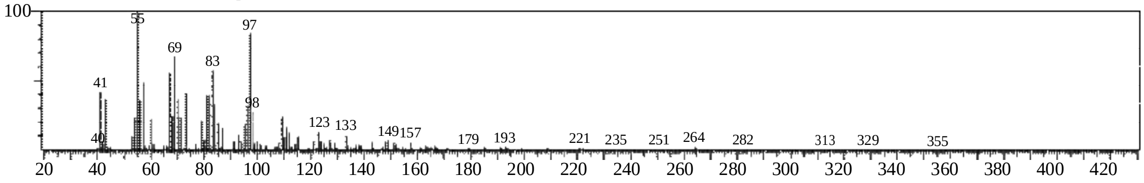

Hit#:1 Entry:90577 Library:NIST05.LIB
SI:87 Formula:C18H34O2 CAS:112-80-1 MolWeight:282 RetIndex:2175
CompName:Oleic Acid $$ 9-Octadecenoic acid (Z)- $$ .delta.(Sup9)-cis-Oleic acid $$ cis-.delta.(Sup9)-Octadecenoic acid $$ cis-Oleic Acid $$ cis-9-Octadecen

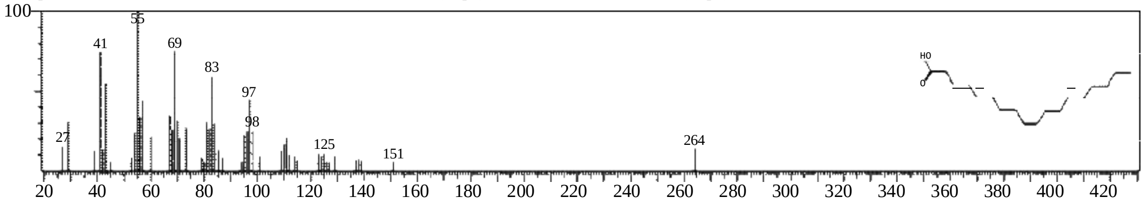

Hit#:2 Entry:65295 Library:NIST05.LIB
SI:86 Formula:C15H28O2 CAS:4727-18-8 MolWeight:240 RetIndex:2158
CompName:Cyclopentadecanone, 2-hydroxy- $$ 2-Hydroxycyclopentadecanone # $$

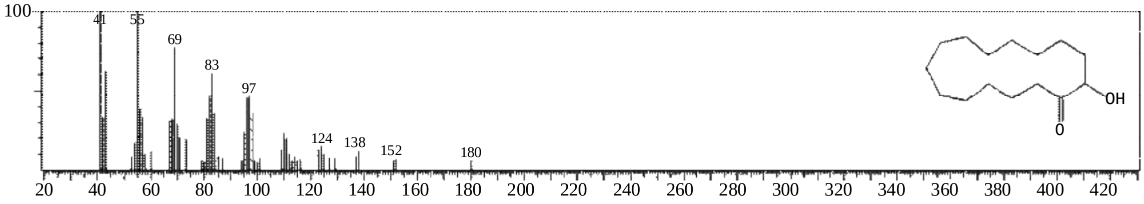

Hit#:3 Entry:90568 Library:NIST05.LIB
SI:84 Formula:C18H34O2 CAS:593-39-5 MolWeight:282 RetIndex:2175
CompName:6-Octadecenoic acid, (Z)- $$ (6Z)-6-Octadecenoic acid # $$

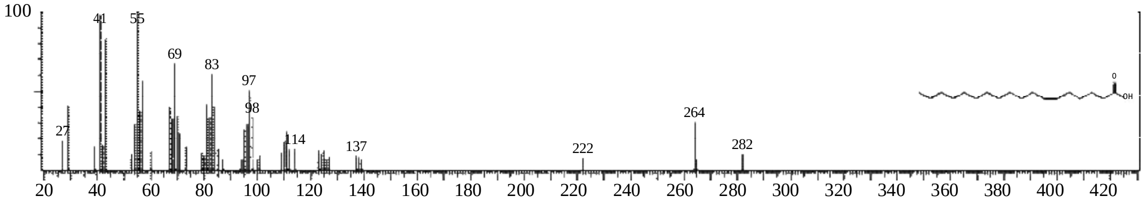

Hit#:4 Entry:80935 Library:NIST05.LIB
SI:84 Formula:C18H34O CAS:2423-10-1 MolWeight:266 RetIndex:2007
CompName:9-Octadecenal, (Z)- $$ Olealdehyde $$ cis-9-Octadecenal $$ Oleylaldehyde $$ Z-9-Octadecenal $$ (9Z)-9-Octadecenal # $$

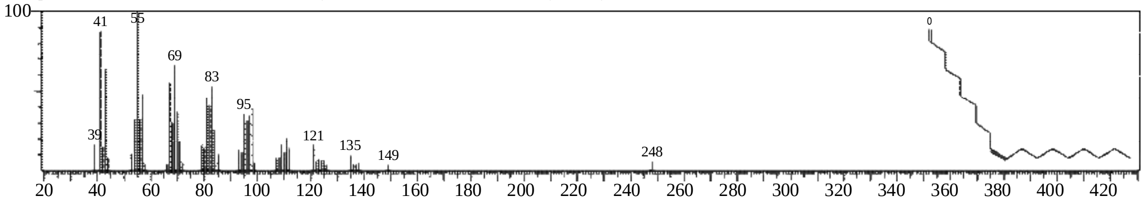

Hit#:5 Entry:163045 Library:NIST05.LIB
SI:84 Formula:C57H104O6 CAS:537-39-3 MolWeight:884 RetIndex:6149
CompName:9-Octadecenoic acid, 1,2,3-propanetriyl ester, (E,E,E)- $$ 2,3-Bis[(9E)-9-octadecenoyloxy]propyl (9E)-9-octadecenoate # $$

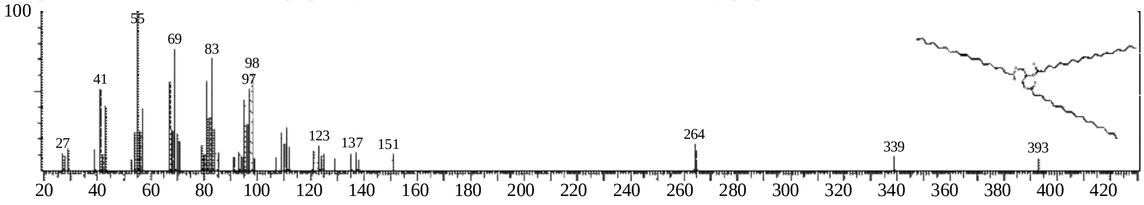

<< Target >>
Line#:43 R.Time:18.742(Scan#:1890) MassPeaks:161
RawMode:Averaged 18.733-18.750(1889-1891) BasePeak:73.05(5009)
BG Mode:Calc. from Peak Group 1 - Event 1

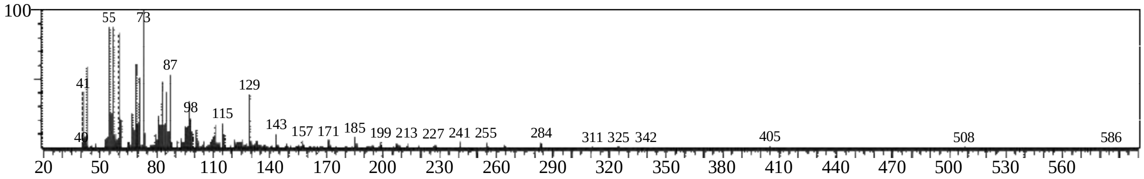

Hit#:1 Entry:22979 Library:NIST05s.LIB
SI:85 Formula:C18H36O2 CAS:57-11-4 MolWeight:284 RetIndex:2167
CompName:Octadecanoic acid $$ Stearic acid $$ n-Octadecanoic acid $$ Humko Industrene R $$ Hydrofol Acid 150 $$ Hystrene S-97 $$ Hystrene T-70 $$ Hys

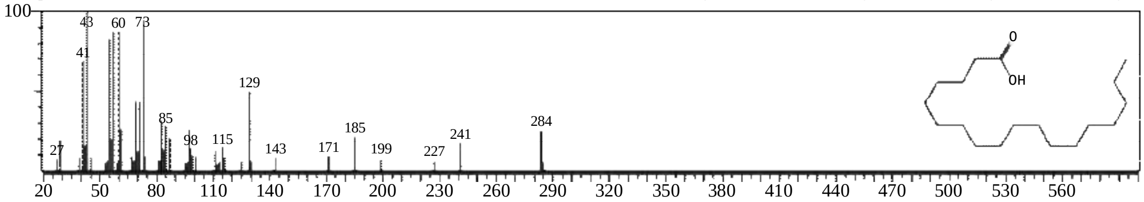

Hit#:2 Entry:161860 Library:NIST05.LIB
SI:85 Formula:C38H68O8 CAS:28474-90-0 MolWeight:652 RetIndex:4765
CompName:l-(+)-Ascorbic acid 2,6-dihexadecanoate

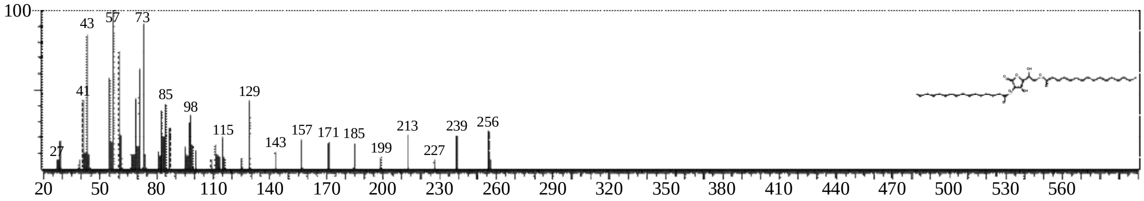

Hit#:3 Entry:20371 Library:NIST05s.LIB
SI:84 Formula:C15H30O2 CAS:1002-84-2 MolWeight:242 RetIndex:1869
CompName:Pentadecanoic acid $$ Pentadecylic acid $$ n-Pentadecanoic acid $$ n-Pentadecylic acid $$

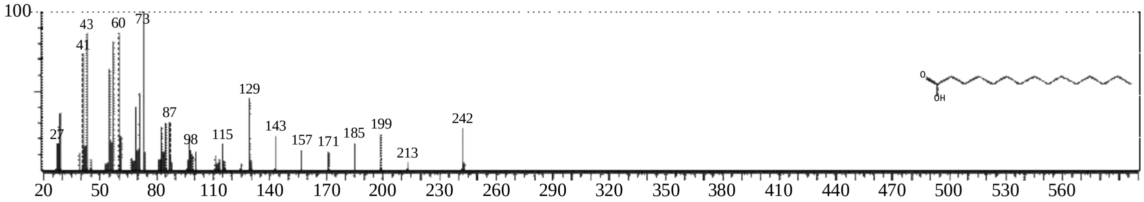

Hit#:4 Entry:19250 Library:NIST05s.LIB
SI:84 Formula:C14H28O2 CAS:544-63-8 MolWeight:228 RetIndex:1769
CompName:Tetradecanoic acid $$ Myristic acid $$ n-Tetradecanoic acid $$ n-Tetradecoic acid $$ Neo-Fat 14 $$ Univol U 316S $$ 1-Tridecanecarboxylic acid

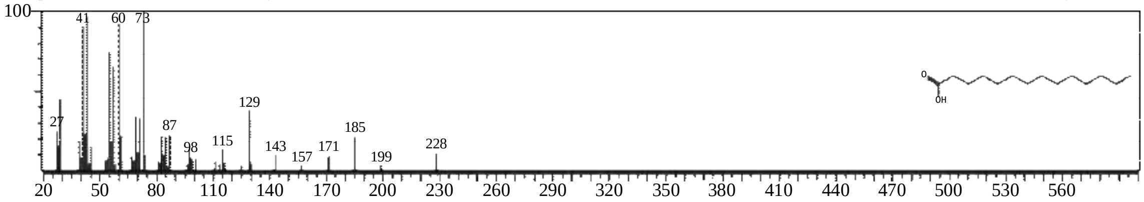

Hit#:5 Entry:19252 Library:NIST05s.LIB
SI:84 Formula:C14H28O2 CAS:544-63-8 MolWeight:228 RetIndex:1769
CompName:Tetradecanoic acid $$ Myristic acid $$ n-Tetradecanoic acid $$ n-Tetradecoic acid $$ Neo-Fat 14 $$ Univol U 316S $$ 1-Tridecanecarboxylic acid

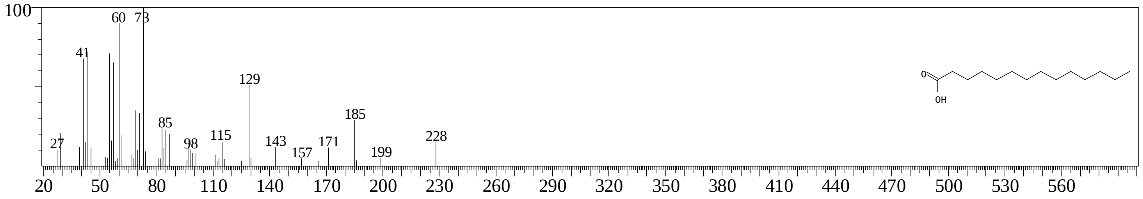

<< Target >>
Line#:44 R.Time:19.658(Scan#:2000) MassPeaks:156
RawMode:Averaged 19.650-19.667(1999-2001) BasePeak:73.10(33320)
BG Mode:Calc. from Peak Group 1 - Event 1

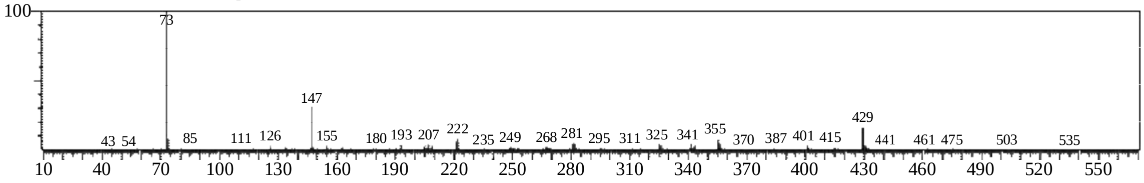

Hit#:1 Entry:160407 Library:NIST05.LIB
SI:77 Formula:C16H50O7Si8 CAS:19095-24-0 MolWeight:578 RetIndex:1710
CompName:Octasiloxane, 1,1,3,3,5,5,7,7,9,9,11,11,13,13,15,15-hexadecamethyl- $$ 1,1,3,3,5,5,7,7,9,9,11,11,13,13,15,15-Hexadecamethyloctasiloxane # $$

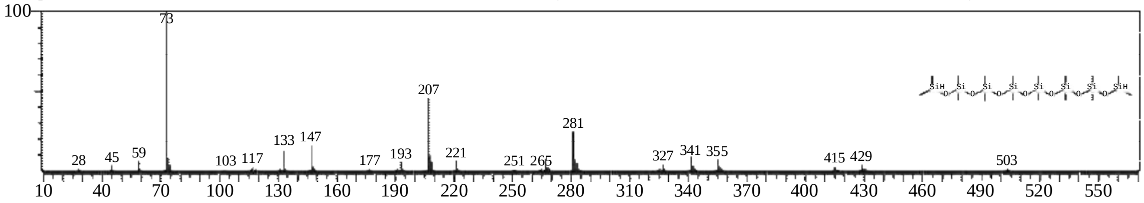

Hit#:2 Entry:157431 Library:NIST05.LIB
SI:76 Formula:C14H44O6Si7 CAS:19095-23-9 MolWeight:504 RetIndex:1526
CompName:Heptasiloxane, 1,1,3,3,5,5,7,7,9,9,11,11,13,13-tetradecamethyl- $$ 1,1,3,3,5,5,7,7,9,9,11,11,13,13-Tetradecamethylheptasiloxane # $$

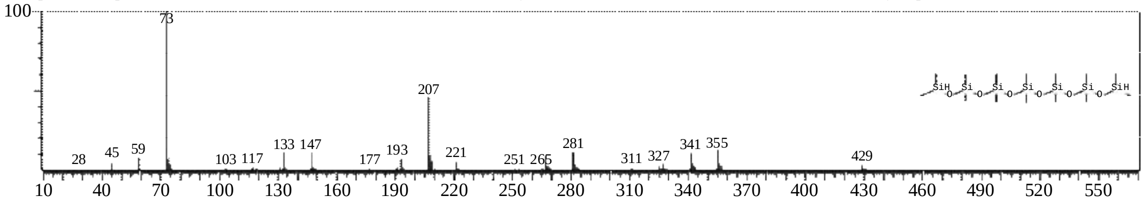

Hit#:3 Entry:152156 Library:NIST05.LIB
SI:75 Formula:C12H36O6Si6 CAS:540-97-6 MolWeight:444 RetIndex:1240
CompName:Cyclohexasiloxane, dodecamethyl- $$ Dodecamethylcyclohexasiloxane $$ 2,2,4,4,6,6,8,8,10,10,12,12-Dodecamethylcyclohexasiloxane # $$

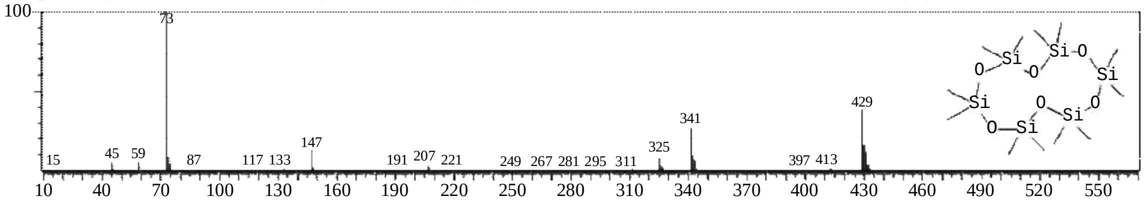

Hit#:4 Entry:160345 Library:NIST05.LIB
SI:74 Formula:C18H52O7Si7 CAS:71579-69-6 MolWeight:576 RetIndex:1648
CompName:3-Isopropoxy-1,1,1,7,7,7-hexamethyl-3,5,5-tris(trimethylsiloxy)tetrasiloxane $$ 1-Isopropoxy-3,3,3-trimethyl-1-[(trimethylsilyl)oxy]disiloxanyl tris

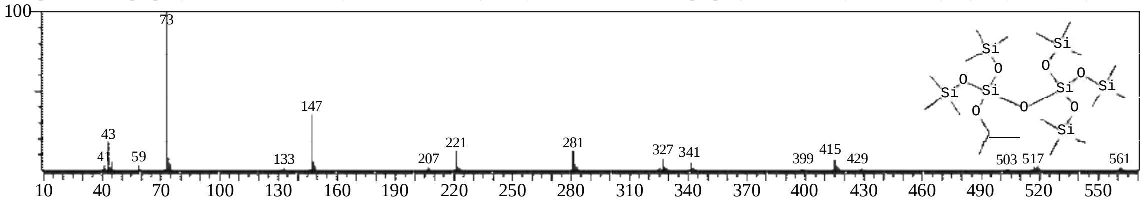

Hit#:5 Entry:27002 Library:NIST05s.LIB
SI:74 Formula:C12H36O6Si6 CAS:540-97-6 MolWeight:444 RetIndex:1240
CompName:Cyclohexasiloxane, dodecamethyl- $$ Dodecamethylcyclohexasiloxane $$ 2,2,4,4,6,6,8,8,10,10,12,12-Dodecamethylcyclohexasiloxane # $$

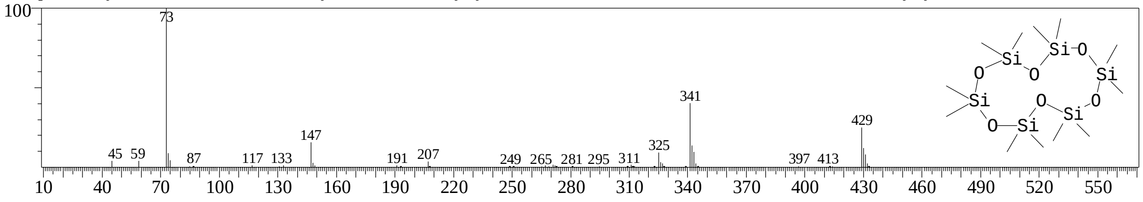

<< Target >>
Line#:45 R.Time:20.025(Scan#:2044) MassPeaks:171
RawMode:Averaged 20.017-20.033(2043-2045) BasePeak:57.10(12701)
BG Mode:Calc. from Peak Group 1 - Event 1

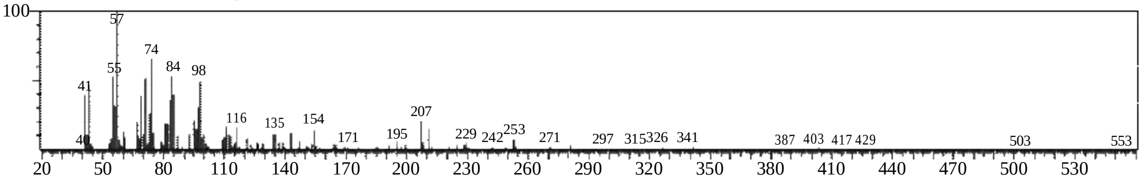

Hit#:1 Entry:117519 Library:NIST05.LIB
SI:82 Formula:C19H38O4 CAS:23470-00-0 MolWeight:330 RetIndex:2498
CompName:Hexadecanoic acid, 2-hydroxy-1-(hydroxymethyl)ethyl ester $$ Palmitin, 2-mono- $$ Palmitic acid .beta.-monoglyceride $$ 2-Hexadecanoyl glycero

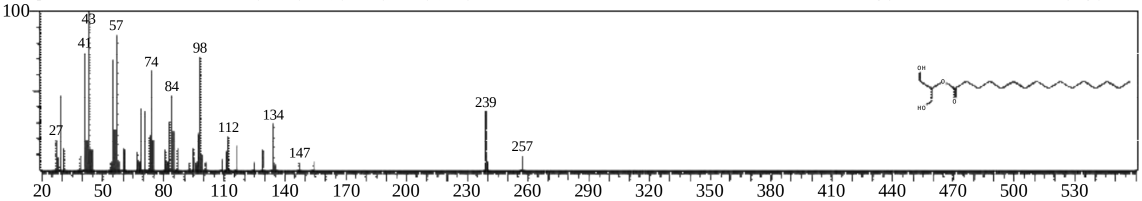

Hit#:2 Entry:110136 Library:NIST05.LIB
SI:81 Formula:C18H36O4 CAS:98863-01-5 MolWeight:316 RetIndex:2399
CompName:Pentadecanoic acid, 2-hydroxy-1-(hydroxymethyl)ethyl ester $$ 2-Hydroxy-1-(hydroxymethyl)ethyl pentadecanoate # $$

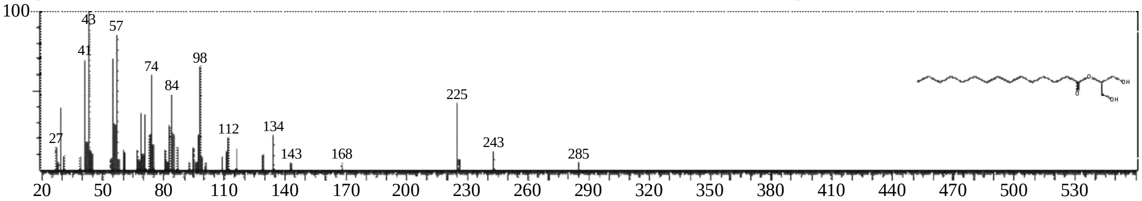

Hit#:3 Entry:130614 Library:NIST05.LIB
SI:79 Formula:C21H42O4 CAS:123-94-4 MolWeight:358 RetIndex:2681
CompName:Octadecanoic acid, 2,3-dihydroxypropyl ester $$ Stearin, 1-mono- $$ .alpha.-Monostearin $$ Aldo MSD $$ Aldo MSLG $$ Aldo 33 $$ Aldo 75 $$

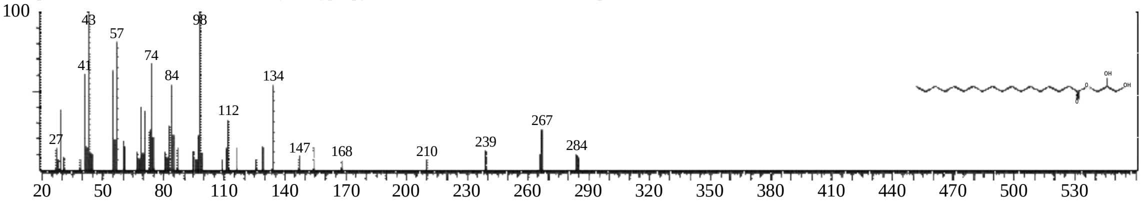

Hit#:4 Entry:140926 Library:NIST05.LIB
SI:78 Formula:C23H46O4 CAS:55334-78-6 MolWeight:386 RetIndex:2896
CompName:Eicosanoic acid, 2-hydroxy-1-(hydroxymethyl)ethyl ester $$ 2-Hydroxy-1-(hydroxymethyl)ethyl icosanoate # $$

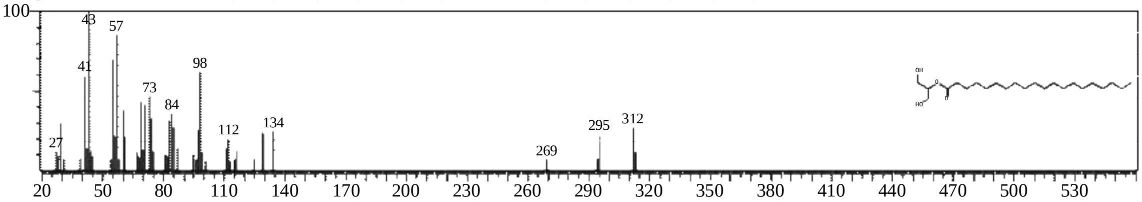

Hit#:5 Entry:102189 Library:NIST05.LIB
SI:78 Formula:C17H34O4 CAS:589-68-4 MolWeight:302 RetIndex:2283
CompName:Tetradecanoic acid, 2,3-dihydroxypropyl ester $$ Myristin, 1-mono- $$ .alpha.-Monomyristin $$ Glycerol 1-myristate $$ Myristic acid 1-monoglyce

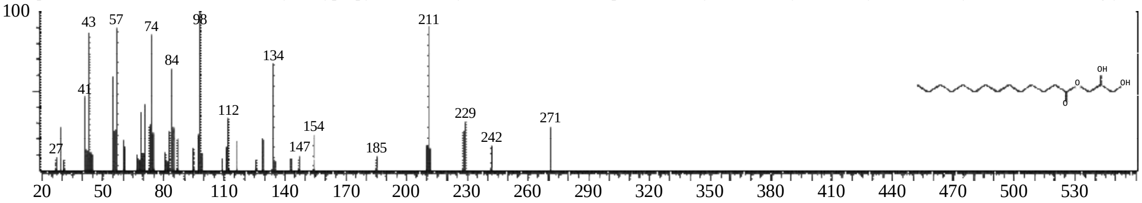

<< Target >>
Line#:46 R.Time:20.467(Scan#:2097) MassPeaks:130
RawMode:Averaged 20.458-20.475(2096-2098) BasePeak:57.10(10343)
BG Mode:Calc. from Peak Group 1 - Event 1

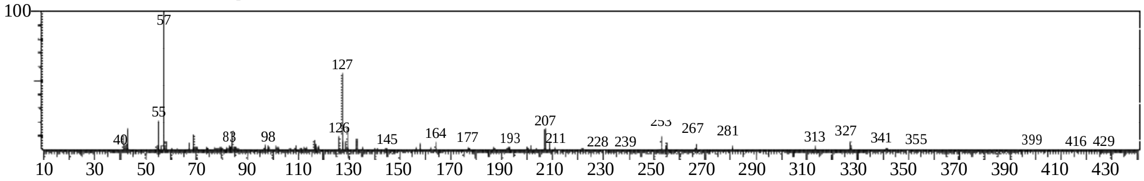

Hit#:1 Entry:24742 Library:NIST05.LIB
SI:72 Formula:C10H18O2 CAS:818-44-0 MolWeight:170 RetIndex:1173
CompName:Vinyl caprylate $$ n-Caprylic acid vinyl ester $$ Octanoic acid, ethenyl ester $$ Caprylic acid, vinyl ester $$ Octanoic acid, ethenyl ester (mixed iso

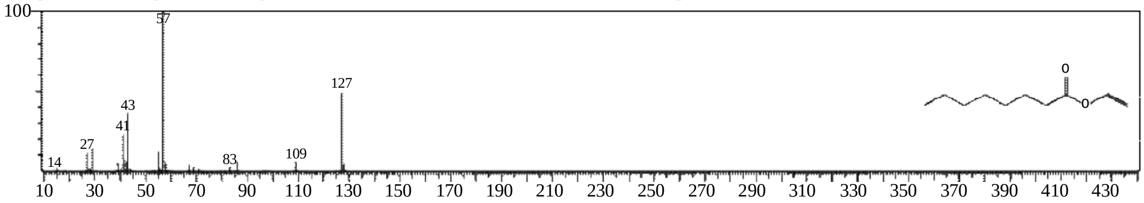

Hit#:2 Entry:154907 Library:NIST05.LIB
SI:72 Formula:C27H50O6 CAS:538-23-8 MolWeight:470 RetIndex:3143
CompName:Glycerol tricaprylate $$ Octanoin, tri- $$ Caprylic acid triglyceride $$ Caprylin $$ Glycerol trioctanoate $$ Glyceryl trioctanoate $$ Octanoic acid t

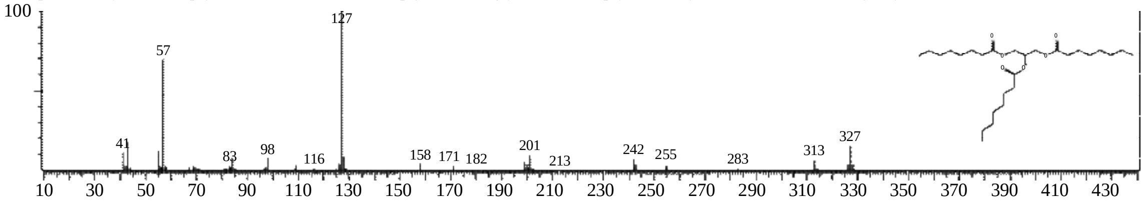

Hit#:3 Entry:74732 Library:NIST05.LIB
SI:72 Formula:C14H18F2O2 CAS:0-00-0 MolWeight:256 RetIndex:1607
CompName:Octanoic acid, 3,5-difluorophenyl ester

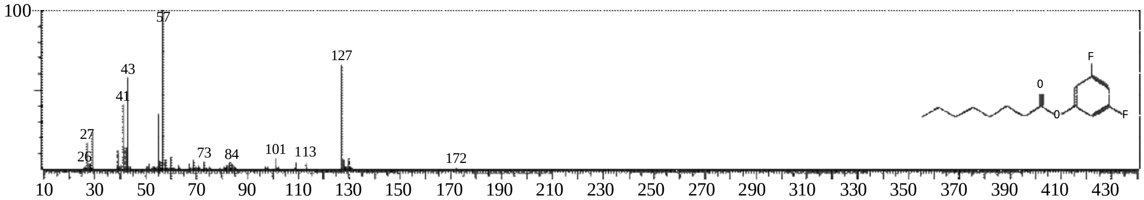

Hit#:4 Entry:68135 Library:NIST05.LIB
SI:72 Formula:C15H19NO2 CAS:0-00-0 MolWeight:245 RetIndex:1935
CompName:Octanoic acid, 4-cyanophenyl ester

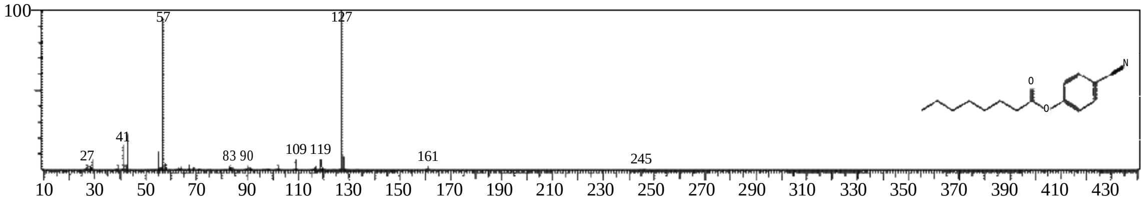

Hit#:5 Entry:67577 Library:NIST05.LIB
SI:71 Formula:C14H28OS CAS:55590-85-7 MolWeight:244 RetIndex:1800
CompName:Octanethioic acid, S-hexyl ester $$ Octanethioic acid, hexyl ester $$ S-Hexyl octanethioate # $$

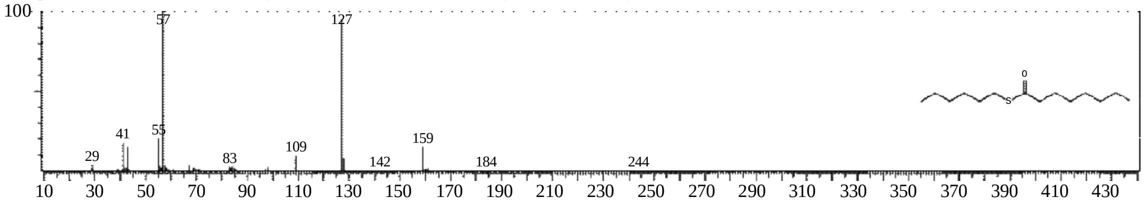

<< Target >>
Line#:47 R.Time:20.592(Scan#:2112) MassPeaks:170
RawMode:Averaged 20.583-20.600(2111-2113) BasePeak:57.05(20226)
BG Mode:Calc. from Peak Group 1 - Event 1

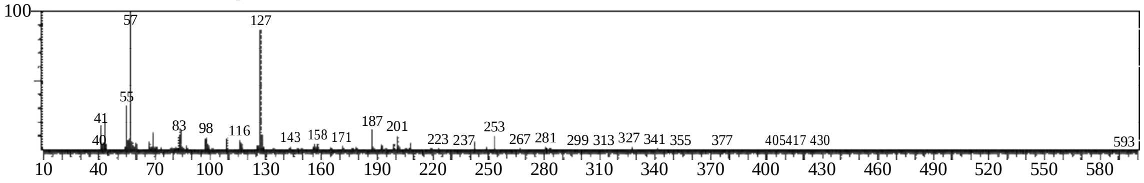

Hit#:1 Entry:154907 Library:NIST05.LIB
SI:79 Formula:C27H50O6 CAS:538-23-8 MolWeight:470 RetIndex:3143
CompName:Glycerol tricaprylate $$ Octanoin, tri- $$ Caprylic acid triglyceride $$ Caprylin $$ Glycerol trioctanoate $$ Glyceryl trioctanoate $$ Octanoic acid t

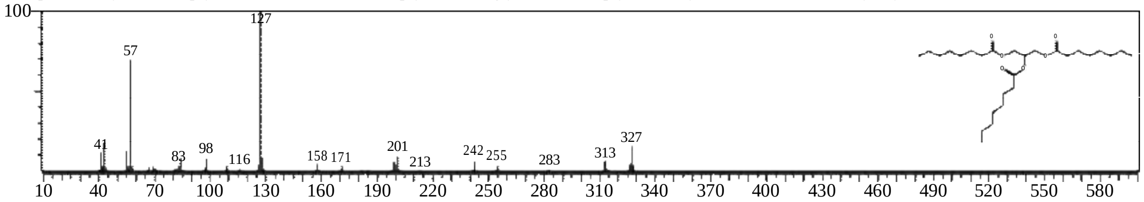

Hit#:2 Entry:67577 Library:NIST05.LIB
SI:77 Formula:C14H28OS CAS:55590-85-7 MolWeight:244 RetIndex:1800
CompName:Octanethioic acid, S-hexyl ester $$ Octanethioic acid, hexyl ester $$ S-Hexyl octanethioate # $$

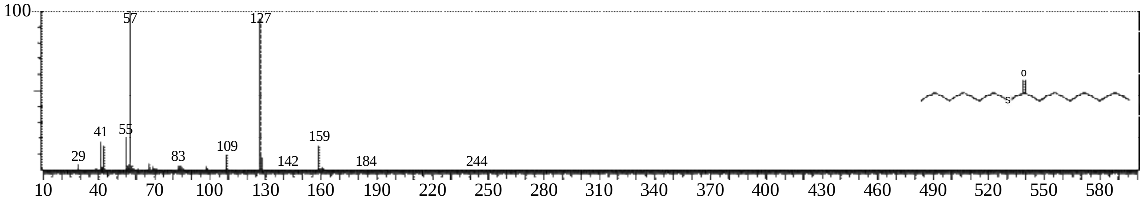

Hit#:3 Entry:19057 Library:NIST05.LIB
SI:77 Formula:C8H17NO2 CAS:7377-03-9 MolWeight:159 RetIndex:1356
CompName:Octanamide, N-hydroxy- $$ Octanohydroxamic acid $$ Caprylohydroxamic acid $$ N-Hydroxyoctanamide $$ Octanoylhydroxamic acid $$ Taselin

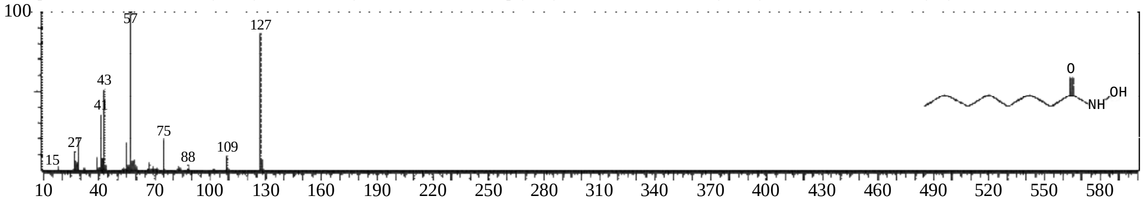

Hit#:4 Entry:68135 Library:NIST05.LIB
SI:75 Formula:C15H19NO2 CAS:0-00-0 MolWeight:245 RetIndex:1935
CompName:Octanoic acid, 4-cyanophenyl ester

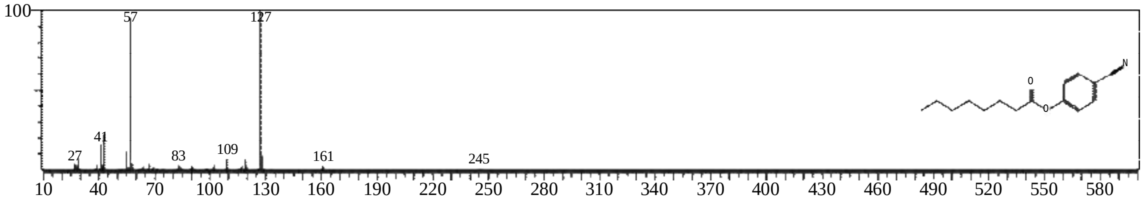

Hit#:5 Entry:24742 Library:NIST05.LIB
SI:74 Formula:C10H18O2 CAS:818-44-0 MolWeight:170 RetIndex:1173
CompName:Vinyl caprylate $$ n-Caprylic acid vinyl ester $$ Octanoic acid, ethenyl ester $$ Caprylic acid, vinyl ester $$ Octanoic acid, ethenyl ester (mixed iso

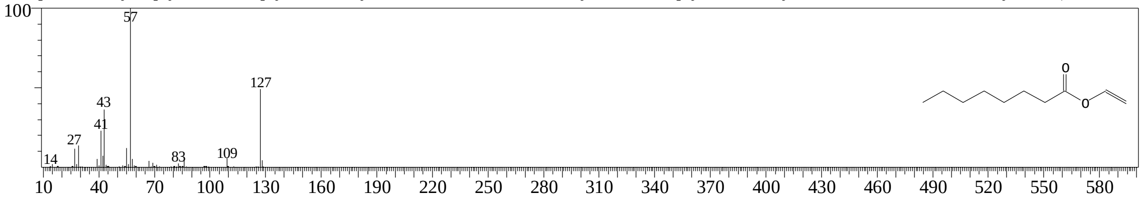

<< Target >>
Line#:48 R.Time:20.833(Scan#:2141) MassPeaks:186
RawMode:Averaged 20.825-20.842(2140-2142) BasePeak:73.10(23275)
BG Mode:Calc. from Peak Group 1 - Event 1

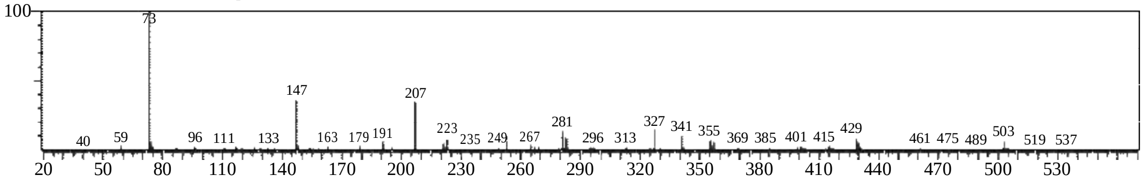

Hit#:1 Entry:160407 Library:NIST05.LIB
SI:79 Formula:C16H50O7Si8 CAS:19095-24-0 MolWeight:578 RetIndex:1710
CompName:Octasiloxane, 1,1,3,3,5,5,7,7,9,9,11,11,13,13,15,15-hexadecamethyl- $$ 1,1,3,3,5,5,7,7,9,9,11,11,13,13,15,15-Hexadecamethyloctasiloxane # $$

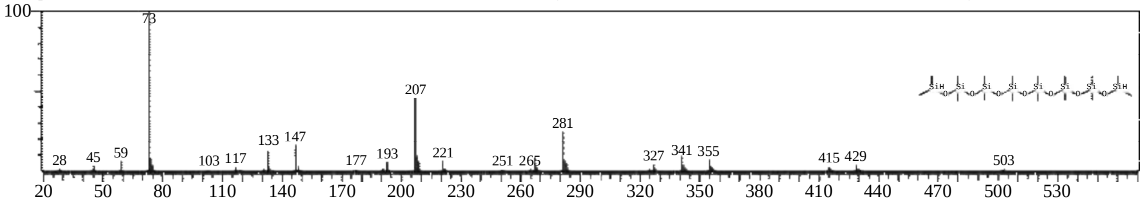

Hit#:2 Entry:157431 Library:NIST05.LIB
SI:76 Formula:C14H44O6Si7 CAS:19095-23-9 MolWeight:504 RetIndex:1526
CompName:Heptasiloxane, 1,1,3,3,5,5,7,7,9,9,11,11,13,13-tetradecamethyl- $$ 1,1,3,3,5,5,7,7,9,9,11,11,13,13-Tetradecamethylheptasiloxane # $$

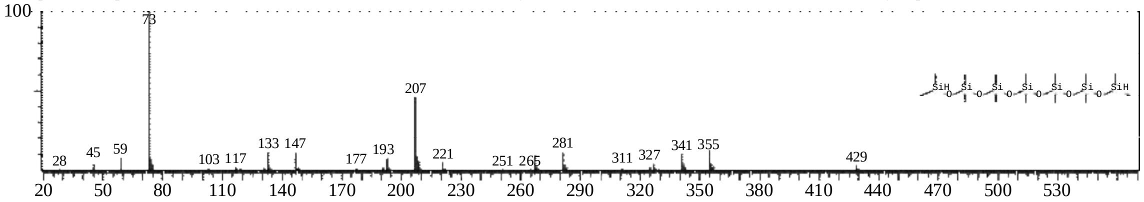

Hit#:3 Entry:150228 Library:NIST05.LIB
SI:75 Formula:C12H38O5Si6 CAS:995-82-4 MolWeight:430 RetIndex:1341
CompName:Hexasiloxane, 1,1,3,3,5,5,7,7,9,9,11,11-dodecamethyl- $$ 1,1,3,3,5,5,7,7,9,9,11,11-Dodecamethylhexasiloxane # $$

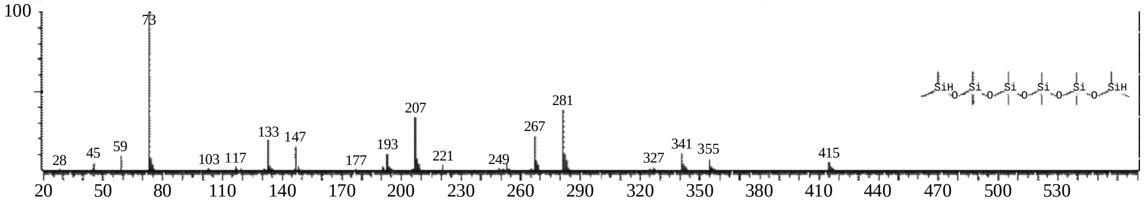

Hit#:4 Entry:160345 Library:NIST05.LIB
SI:75 Formula:C18H52O7Si7 CAS:71579-69-6 MolWeight:576 RetIndex:1648
CompName:3-Isopropoxy-1,1,1,7,7,7-hexamethyl-3,5,5-tris(trimethylsiloxy)tetrasiloxane $$ 1-Isopropoxy-3,3,3-trimethyl-1-[(trimethylsilyl)oxy]disiloxanyl tris

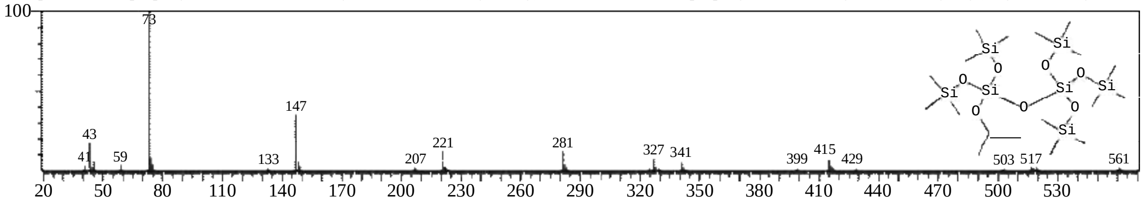

Hit#:5 Entry:158804 Library:NIST05.LIB
SI:74 Formula:C16H48O6Si7 CAS:541-01-5 MolWeight:532 RetIndex:1437
CompName:Heptasiloxane, hexadecamethyl- $$ Hexadecamethylheptasiloxane $$ 1,1,1,3,3,5,5,7,7,9,9,11,11,13,13,13-Hexadecamethylheptasiloxane # $$

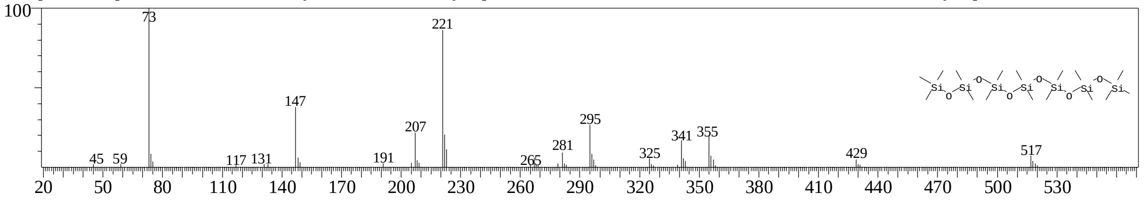

<< Target >>
Line#:49 R.Time:21.450(Scan#:2215) MassPeaks:154
RawMode:Averaged 21.442-21.458(2214-2216) BasePeak:281.15(2449)
BG Mode:Calc. from Peak Group 1 - Event 1

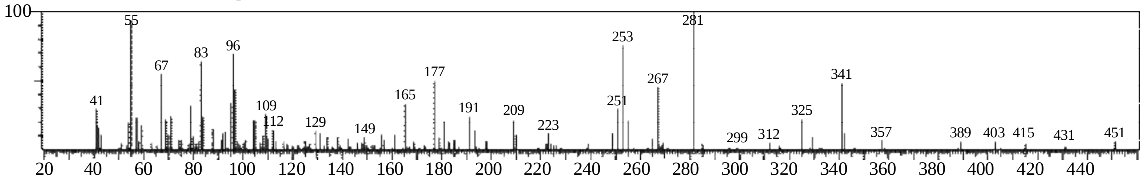

Hit#:1 Entry:17228 Library:NIST05s.LIB
SI:53 Formula:C15H28 CAS:30824-81-8 MolWeight:208 RetIndex:1432
CompName:Naphthalene, decahydro-1,4a-dimethyl-7-(1-methylethyl)-, [1S-(1.alpha.,4a.alpha.,7.alpha.,8a.beta.)]- $$ 4.alpha.H-Eudesmane $$ Selinan $$ Selin

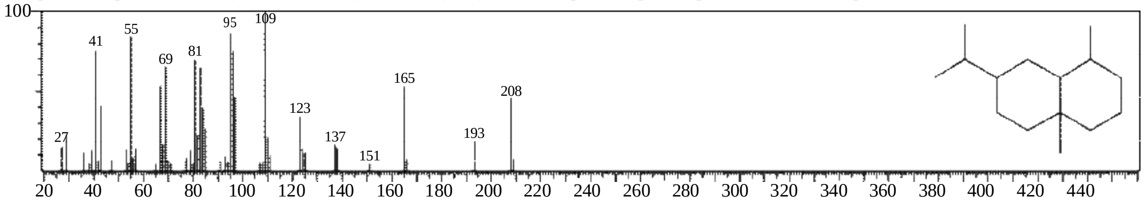

Hit#:2 Entry:160039 Library:NIST05.LIB
SI:52 Formula:C38H76O2 CAS:17367-12-3 MolWeight:564 RetIndex:3958
CompName:9-Octadecene, 1-[2-(octadecyloxy)ethoxy]-, (Z)- $$ Ethane, 1-(9-octadecenyloxy)-2-(octadecyloxy)-, (Z)- $$ (9Z)-1-[2-(Octadecyloxy)ethoxy]-9-oc

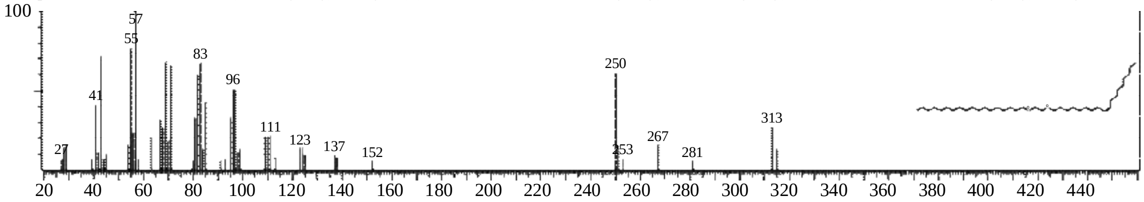

Hit#:3 Entry:89378 Library:NIST05.LIB
SI:52 Formula:C20H40 CAS:56009-20-2 MolWeight:280 RetIndex:1842
CompName:Cyclohexane, 1-(1,5-dimethylhexyl)-4-(4-methylpentyl)- $$ 1-(1,5-Dimethylhexyl)-4-(4-methylpentyl)cyclohexane # $$

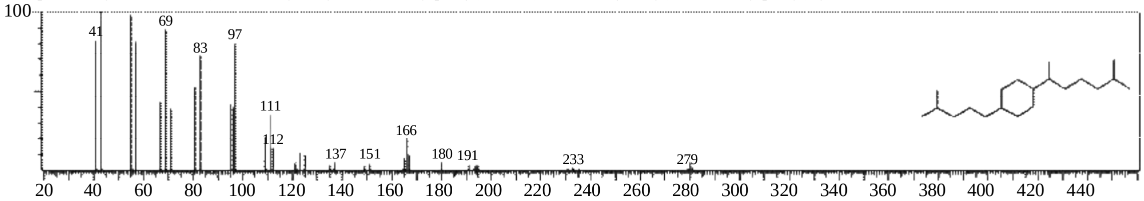

Hit#:4 Entry:46123 Library:NIST05.LIB
SI:52 Formula:C15H28 CAS:30824-81-8 MolWeight:208 RetIndex:1432
CompName:Naphthalene, decahydro-1,4a-dimethyl-7-(1-methylethyl)-, [1S-(1.alpha.,4a.alpha.,7.alpha.,8a.beta.)]- $$ 4.alpha.H-Eudesmane $$ Selinan $$ Selin

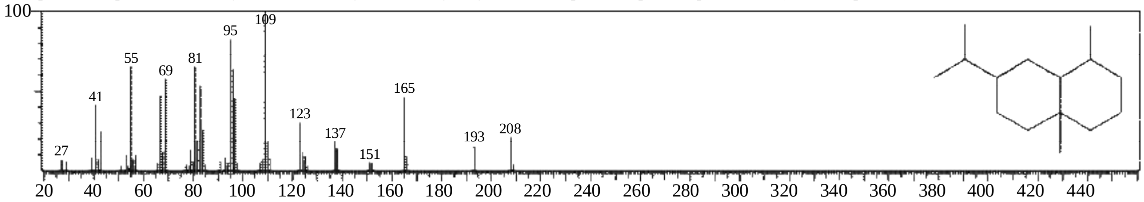

Hit#:5 Entry:46102 Library:NIST05.LIB
SI:52 Formula:C15H28 CAS:114614-84-5 MolWeight:208 RetIndex:1584
CompName:1-Cyclohexylnonene $$ (1E)-1-Nonenylcyclohexane # $$

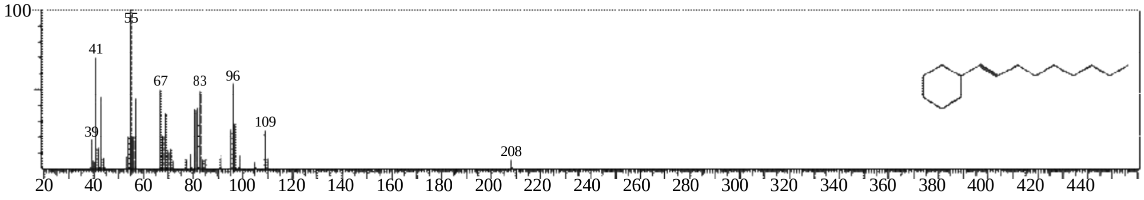

<< Target >>
Line#:50 R.Time:21.717(Scan#:2247) MassPeaks:158
RawMode:Averaged 21.708-21.725(2246-2248) BasePeak:207.00(5239)
BG Mode:Calc. from Peak Group 1 - Event 1

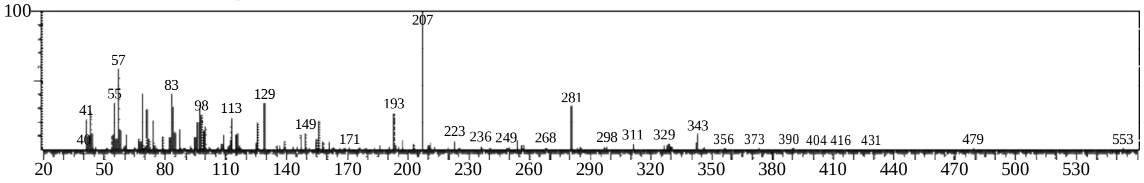

Hit#:1 Entry:80943 Library:NIST05.LIB
SI:64 Formula:C18H34O CAS:56554-88-2 MolWeight:266 RetIndex:2007
CompName:5-Octadecenal $$ (5E)-5-Octadecenal # $$

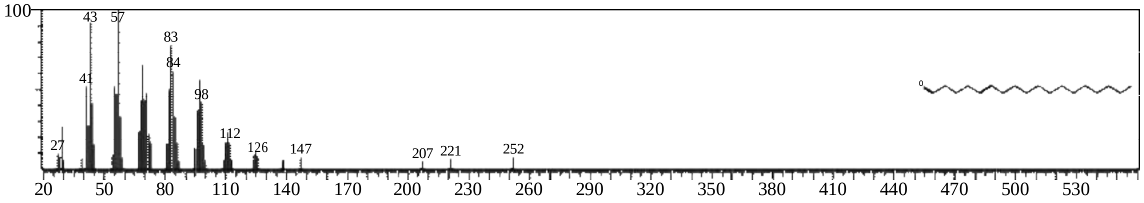

Hit#:2 Entry:125389 Library:NIST05.LIB
SI:63 Formula:C20H39ClO2 CAS:5348-82-3 MolWeight:346 RetIndex:2402
CompName:Acetic acid, chloro-, octadecyl ester $$ Chloroacetic acid, octadecyl ester $$ Octadecyl chloroacetate # $$

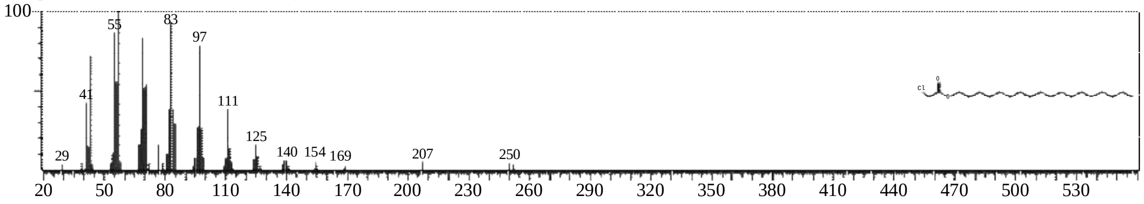

Hit#:3 Entry:61283 Library:NIST05.LIB
SI:63 Formula:C11H23Br CAS:55162-38-4 MolWeight:234 RetIndex:1283
CompName:Nonane, 2-bromo-5-ethyl- $$ 2-Bromo-5-ethylnonane # $$

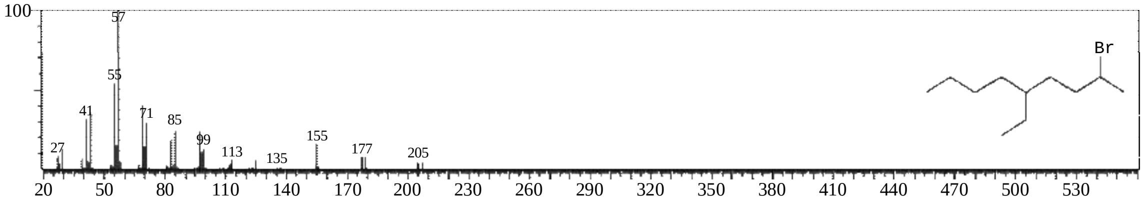

Hit#:4 Entry:100059 Library:NIST05.LIB
SI:63 Formula:C19H38O2 CAS:15965-99-8 MolWeight:298 RetIndex:2076
CompName:Oxirane, [(hexadecyloxy)methyl]- $$ Propane, 1,2-epoxy-3-(hexadecyloxy)- $$ (Cetyloxymethyl)oxirane $$ Cetyl glycidyl ether $$ Glycidol .alpha.

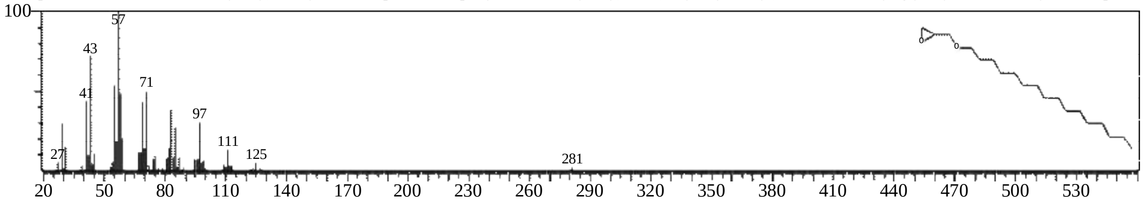

Hit#:5 Entry:27524 Library:NIST05s.LIB
SI:63 Formula:C39H76O5 CAS:504-40-5 MolWeight:624 RetIndex:4395
CompName:Octadecanoic acid, 2-hydroxy-1,3-propanediyl ester $$ Stearin, 1,3-di- $$ Glycerin 1,3-distearate $$ Glyceryl 1,3-distearate $$ Stearic acid diglycer

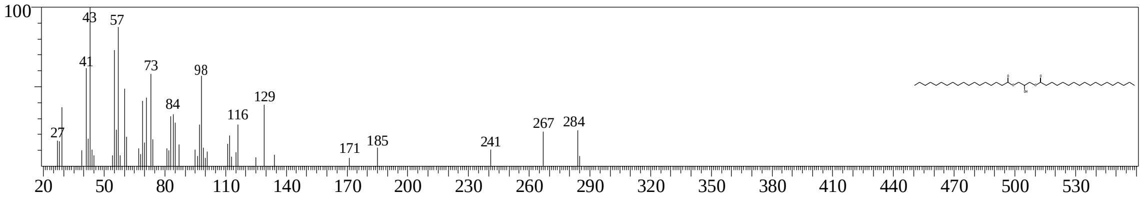

<< Target >>
Line#:51 R.Time:21.933(Scan#:2273) MassPeaks:182
RawMode:Averaged 21.925-21.942(2272-2274) BasePeak:73.10(19360)
BG Mode:Calc. from Peak Group 1 - Event 1

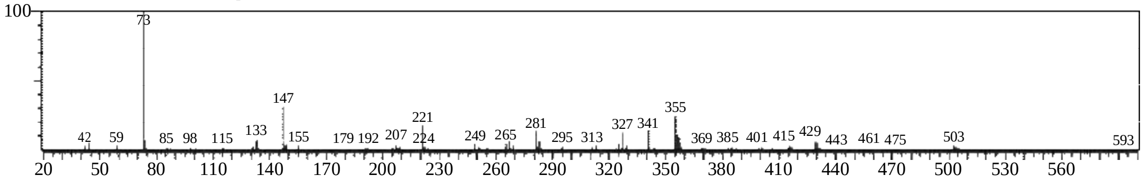

Hit#:1 Entry:160407 Library:NIST05.LIB
SI:76 Formula:C16H50O7Si8 CAS:19095-24-0 MolWeight:578 RetIndex:1710
CompName:Octasiloxane, 1,1,3,3,5,5,7,7,9,9,11,11,13,13,15,15-hexadecamethyl- $$ 1,1,3,3,5,5,7,7,9,9,11,11,13,13,15,15-Hexadecamethyloctasiloxane # $$

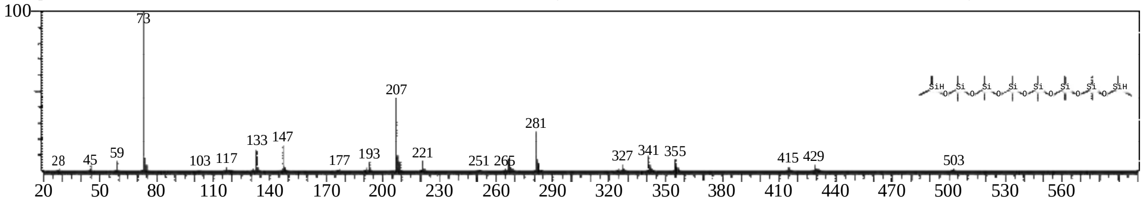

Hit#:2 Entry:157431 Library:NIST05.LIB
SI:75 Formula:C14H44O6Si7 CAS:19095-23-9 MolWeight:504 RetIndex:1526
CompName:Heptasiloxane, 1,1,3,3,5,5,7,7,9,9,11,11,13,13-tetradecamethyl- $$ 1,1,3,3,5,5,7,7,9,9,11,11,13,13-Tetradecamethylheptasiloxane # $$

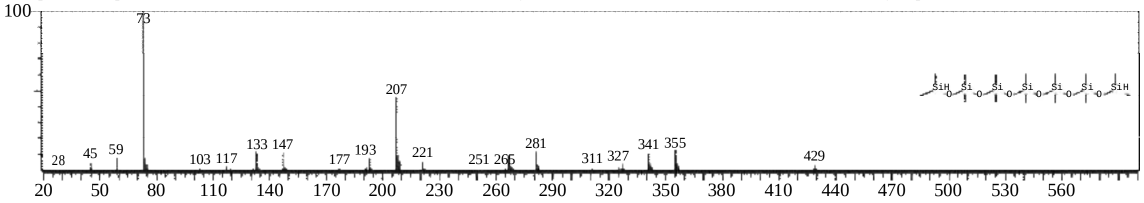

Hit#:3 Entry:158804 Library:NIST05.LIB
SI:74 Formula:C16H48O6Si7 CAS:541-01-5 MolWeight:532 RetIndex:1437
CompName:Heptasiloxane, hexadecamethyl- $$ Hexadecamethylheptasiloxane $$ 1,1,1,3,3,5,5,7,7,9,9,11,11,13,13,13-Hexadecamethylheptasiloxane # $$

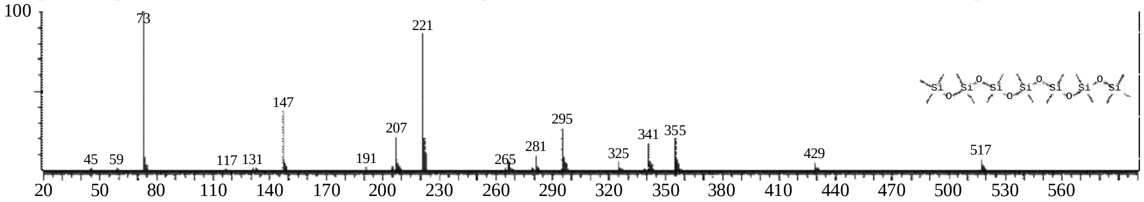

Hit#:4 Entry:150228 Library:NIST05.LIB
SI:71 Formula:C12H38O5Si6 CAS:995-82-4 MolWeight:430 RetIndex:1341
CompName:Hexasiloxane, 1,1,3,3,5,5,7,7,9,9,11,11-dodecamethyl- $$ 1,1,3,3,5,5,7,7,9,9,11,11-Dodecamethylhexasiloxane # $$

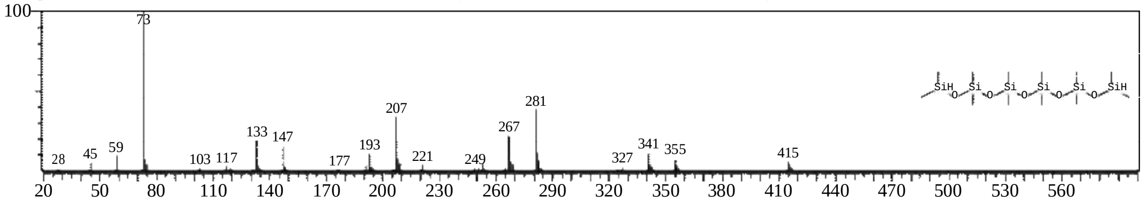

Hit#:5 Entry:160345 Library:NIST05.LIB
SI:71 Formula:C18H52O7Si7 CAS:71579-69-6 MolWeight:576 RetIndex:1648
CompName:3-Isopropoxy-1,1,1,7,7,7-hexamethyl-3,5,5-tris(trimethylsiloxy)tetrasiloxane $$ 1-Isopropoxy-3,3,3-trimethyl-1-[(trimethylsilyl)oxy]disiloxanyl tris

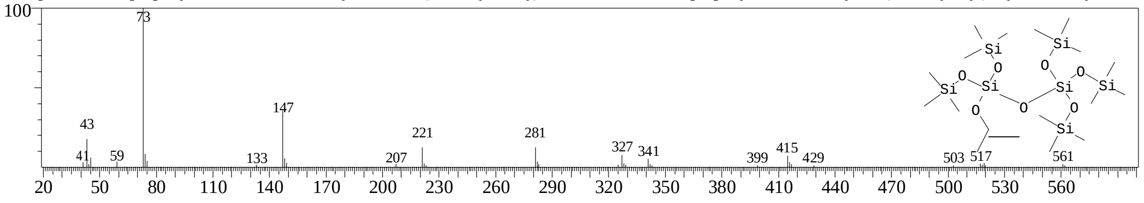

<< Target >>
Line#:52 R.Time:22.092(Scan#:2292) MassPeaks:189
RawMode:Averaged 22.083-22.100(2291-2293) BasePeak:99.05(15690)
BG Mode:Calc. from Peak Group 1 - Event 1

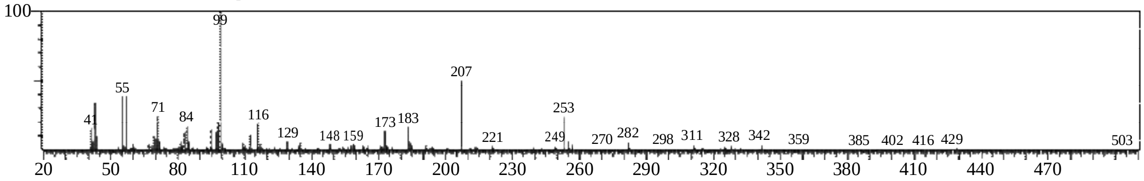

Hit#:1 Entry:100084 Library:NIST05.LIB
SI:70 Formula:C19H38O2 CAS:0-00-0 MolWeight:298 RetIndex:2013
CompName:Hexanoic acid, 4-tridecyl ester

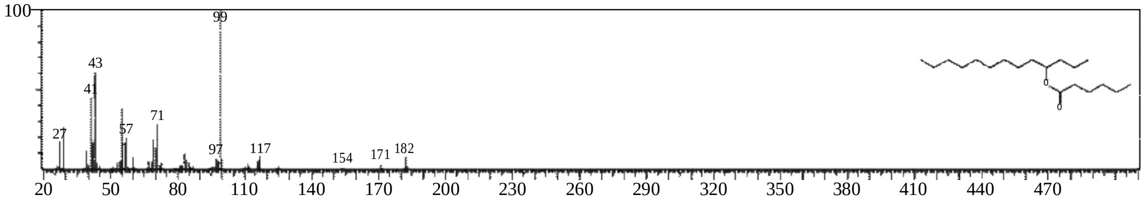

Hit#:2 Entry:122714 Library:NIST05.LIB
SI:69 Formula:C22H44O2 CAS:0-00-0 MolWeight:340 RetIndex:2311
CompName:Hexanoic acid, 4-hexadecyl ester

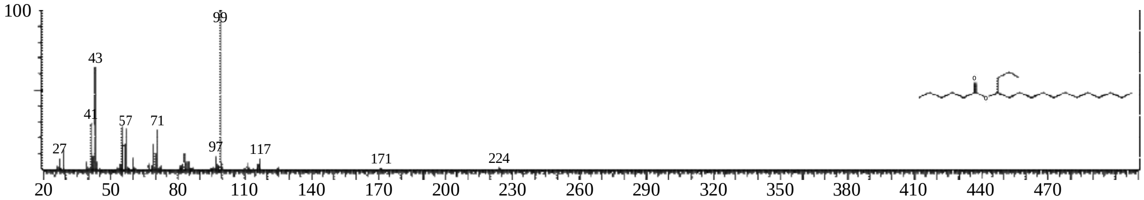

Hit#:3 Entry:100083 Library:NIST05.LIB
SI:69 Formula:C19H38O2 CAS:0-00-0 MolWeight:298 RetIndex:2013
CompName:Hexanoic acid, 3-tridecyl ester

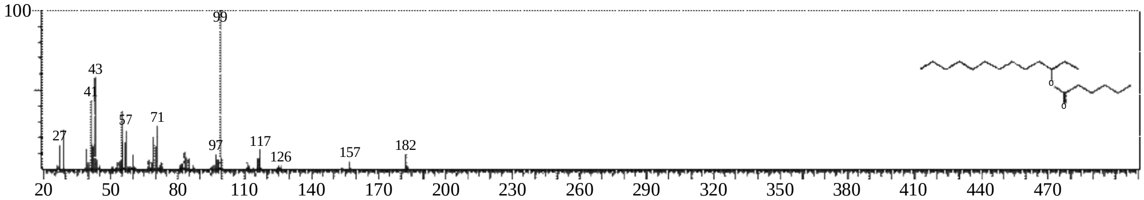

Hit#:4 Entry:100085 Library:NIST05.LIB
SI:68 Formula:C19H38O2 CAS:0-00-0 MolWeight:298 RetIndex:2013
CompName:Hexanoic acid, 5-tridecyl ester

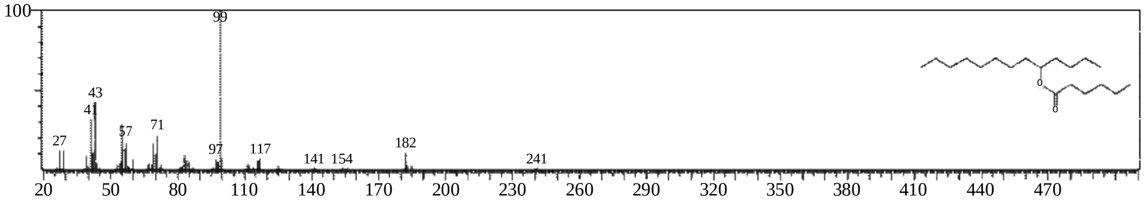

Hit#:5 Entry:100086 Library:NIST05.LIB
SI:68 Formula:C19H38O2 CAS:0-00-0 MolWeight:298 RetIndex:2013
CompName:Hexanoic acid, 6-tridecyl ester

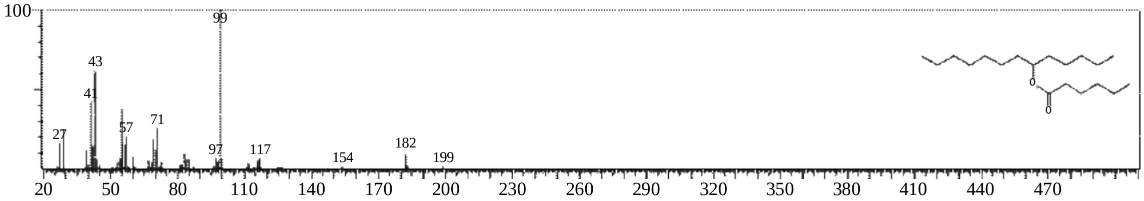

<< Target >>
Line#:53 R.Time:22.192(Scan#:2304) MassPeaks:248
RawMode:Averaged 22.183-22.200(2303-2305) BasePeak:99.15(31844)
BG Mode:Calc. from Peak Group 1 - Event 1

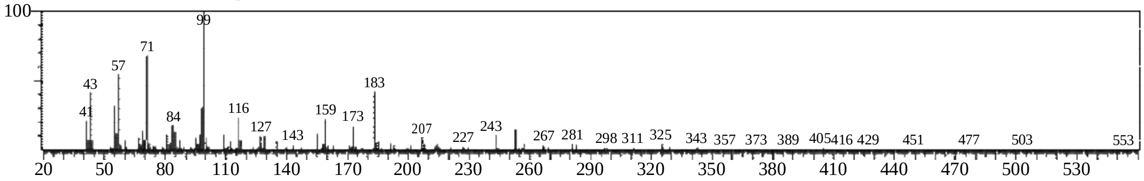

Hit#:1 Entry:100083 Library:NIST05.LIB
SI:72 Formula:C19H38O2 CAS:0-00-0 MolWeight:298 RetIndex:2013
CompName:Hexanoic acid, 3-tridecyl ester

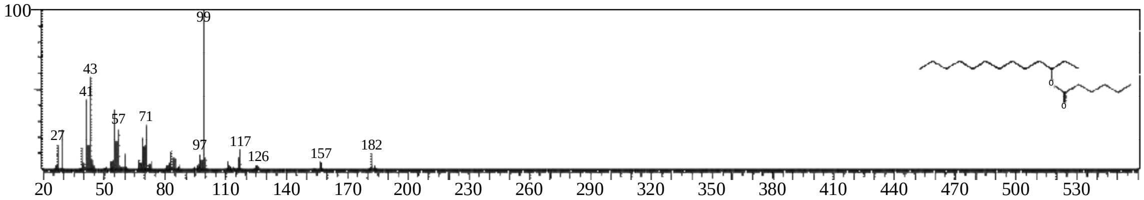

Hit#:2 Entry:100086 Library:NIST05.LIB
SI:71 Formula:C19H38O2 CAS:0-00-0 MolWeight:298 RetIndex:2013
CompName:Hexanoic acid, 6-tridecyl ester

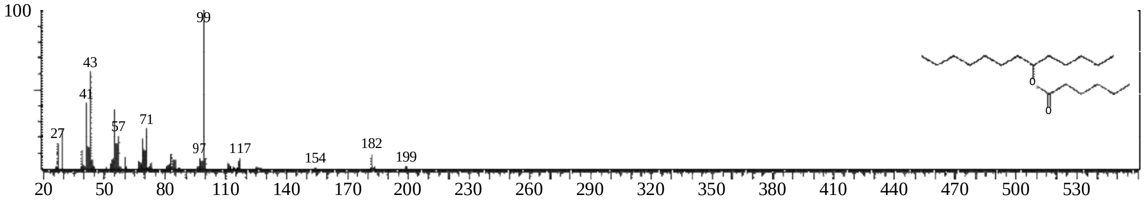

Hit#:3 Entry:153564 Library:NIST05.LIB
SI:71 Formula:C27H52O5 CAS:17598-94-6 MolWeight:456 RetIndex:3218
CompName:Dodecanoic acid, 1-(hydroxymethyl)-1,2-ethanediyl ester $$ 2-(Dodecanoyloxy)-1-(hydroxymethyl)ethyl laurate # $$

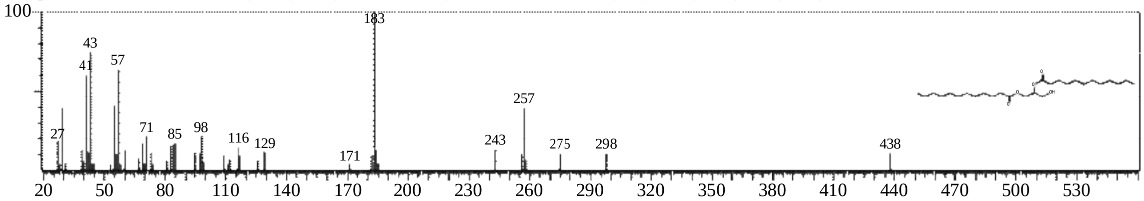

Hit#:4 Entry:100084 Library:NIST05.LIB
SI:71 Formula:C19H38O2 CAS:0-00-0 MolWeight:298 RetIndex:2013
CompName:Hexanoic acid, 4-tridecyl ester

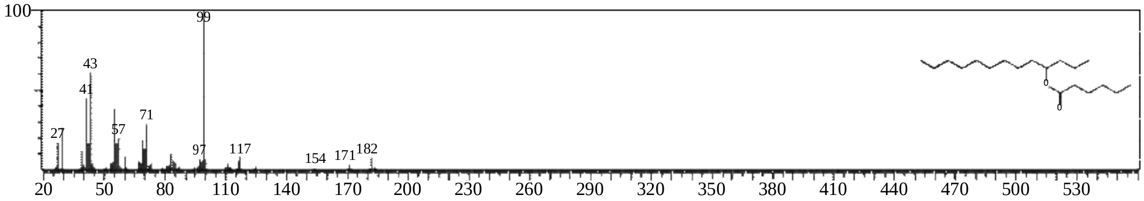

Hit#:5 Entry:100085 Library:NIST05.LIB
SI:71 Formula:C19H38O2 CAS:0-00-0 MolWeight:298 RetIndex:2013
CompName:Hexanoic acid, 5-tridecyl ester

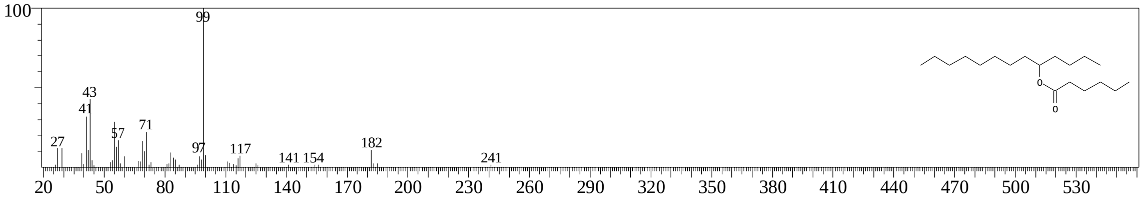

<< Target >>
Line#:54 R.Time:22.967(Scan#:2397) MassPeaks:209
RawMode:Averaged 22.958-22.975(2396-2398) BasePeak:73.10(21672)
BG Mode:Calc. from Peak Group 1 - Event 1

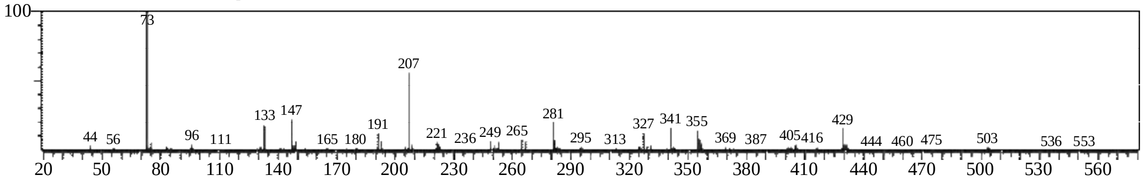

Hit#:1 Entry:160407 Library:NIST05.LIB
SI:81 Formula:C16H50O7Si8 CAS:19095-24-0 MolWeight:578 RetIndex:1710
CompName:Octasiloxane, 1,1,3,3,5,5,7,7,9,9,11,11,13,13,15,15-hexadecamethyl- $$ 1,1,3,3,5,5,7,7,9,9,11,11,13,13,15,15-Hexadecamethyloctasiloxane # $$

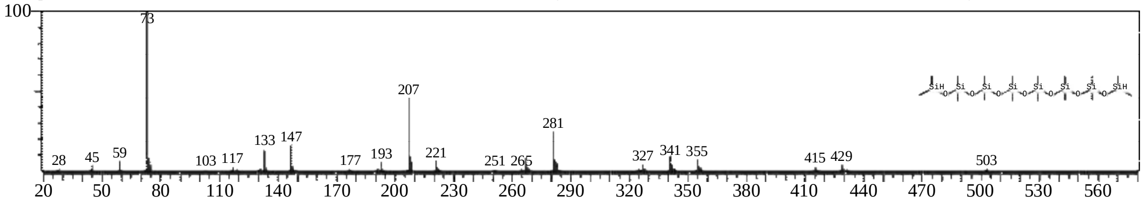

Hit#:2 Entry:157431 Library:NIST05.LIB
SI:80 Formula:C14H44O6Si7 CAS:19095-23-9 MolWeight:504 RetIndex:1526
CompName:Heptasiloxane, 1,1,3,3,5,5,7,7,9,9,11,11,13,13-tetradecamethyl- $$ 1,1,3,3,5,5,7,7,9,9,11,11,13,13-Tetradecamethylheptasiloxane # $$

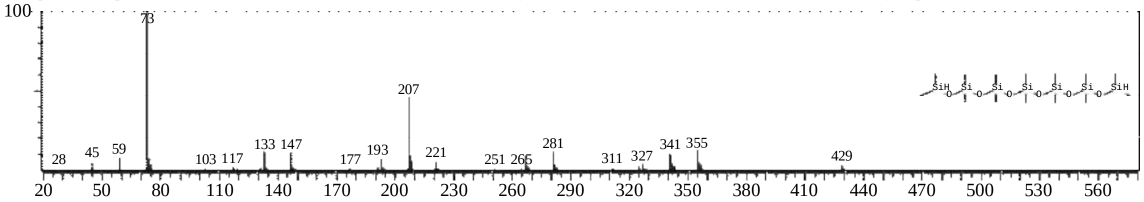

Hit#:3 Entry:150228 Library:NIST05.LIB
SI:76 Formula:C12H38O5Si6 CAS:995-82-4 MolWeight:430 RetIndex:1341
CompName:Hexasiloxane, 1,1,3,3,5,5,7,7,9,9,11,11-dodecamethyl- $$ 1,1,3,3,5,5,7,7,9,9,11,11-Dodecamethylhexasiloxane # $$

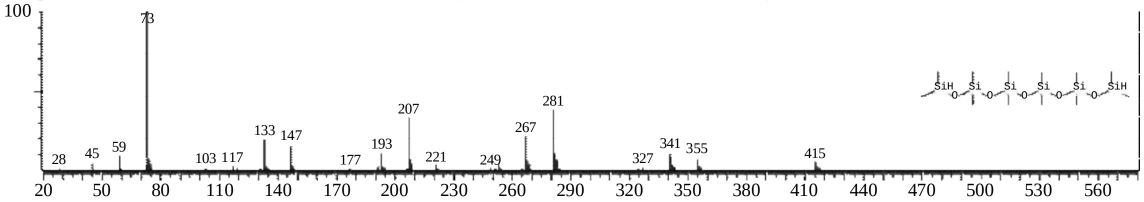

Hit#:4 Entry:158804 Library:NIST05.LIB
SI:73 Formula:C16H48O6Si7 CAS:541-01-5 MolWeight:532 RetIndex:1437
CompName:Heptasiloxane, hexadecamethyl- $$ Hexadecamethylheptasiloxane $$ 1,1,1,3,3,5,5,7,7,9,9,11,11,13,13,13-Hexadecamethylheptasiloxane # $$

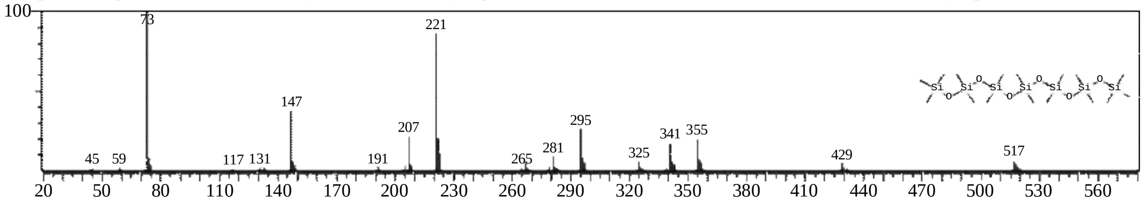

Hit#:5 Entry:27366 Library:NIST05s.LIB
SI:72 Formula:C16H48O6Si7 CAS:541-01-5 MolWeight:532 RetIndex:1437
CompName:Heptasiloxane, hexadecamethyl- $$ Hexadecamethylheptasiloxane $$ 1,1,1,3,3,5,5,7,7,9,9,11,11,13,13,13-Hexadecamethylheptasiloxane # $$

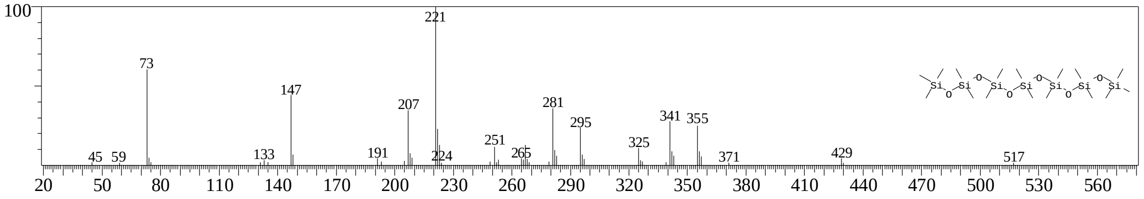

<< Target >>
Line#:55 R.Time:23.117(Scan#:2415) MassPeaks:169
RawMode:Averaged 23.108-23.125(2414-2416) BasePeak:207.10(12317)
BG Mode:Calc. from Peak Group 1 - Event 1

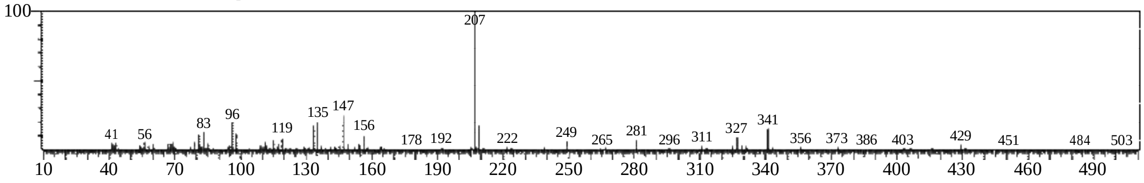

Hit#:1 Entry:54551 Library:NIST05.LIB
SI:56 Formula:C15H26O CAS:117591-80-7 MolWeight:222 RetIndex:1432
CompName:3,3,7,11-Tetramethyltricyclo[5.4.0.0(4,11)]undecan-1-ol

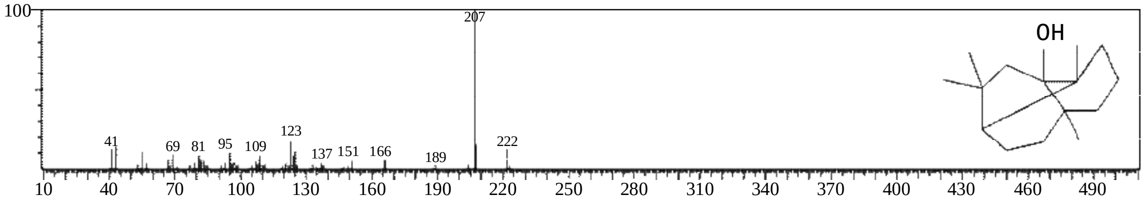

Hit#:2 Entry:53689 Library:NIST05.LIB
SI:56 Formula:C6H18O3Si3 CAS:541-05-9 MolWeight:222 RetIndex:620
CompName:Cyclotrisiloxane, hexamethyl- $$ Dimethylsiloxane cyclic trimer $$ Hexamethylcyclotrisiloxane $$ CH7260 $$ 2,2,4,4,6,6-Hexamethyl-1,3,5,2,4,6-

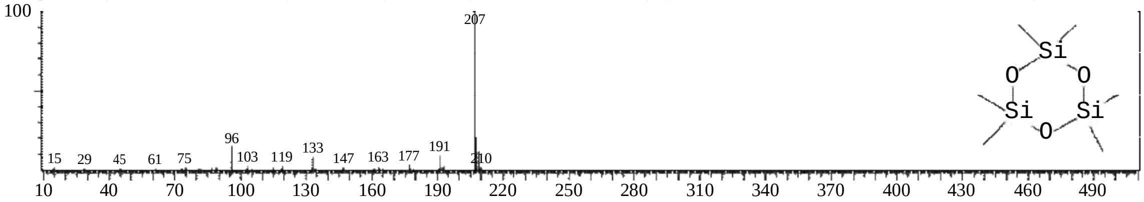

Hit#:3 Entry:18524 Library:NIST05s.LIB
SI:55 Formula:C6H18O3Si3 CAS:541-05-9 MolWeight:222 RetIndex:620
CompName:Cyclotrisiloxane, hexamethyl- $$ Dimethylsiloxane cyclic trimer $$ Hexamethylcyclotrisiloxane $$ CH7260 $$ 2,2,4,4,6,6-Hexamethyl-1,3,5,2,4,6-

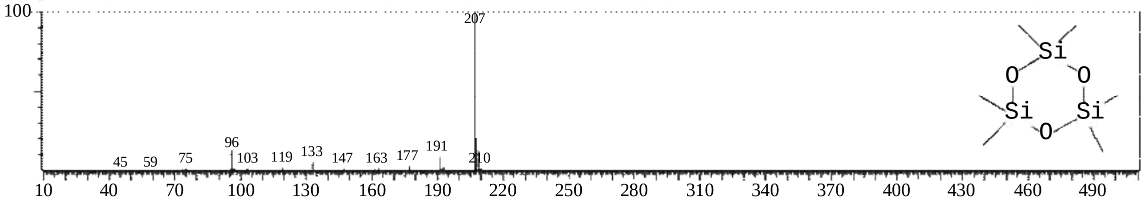

Hit#:4 Entry:54151 Library:NIST05.LIB
SI:55 Formula:C12H22Si2 CAS:13183-70-5 MolWeight:222 RetIndex:1124
CompName:Silane, 1,4-phenylenebis[trimethyl- $$ Silane, p-phenylenebis[trimethyl- $$ p-Bis(trimethylsilyl)benzene $$ Benzene, p-bis(trimethylsilyl)- $$ 1,4-

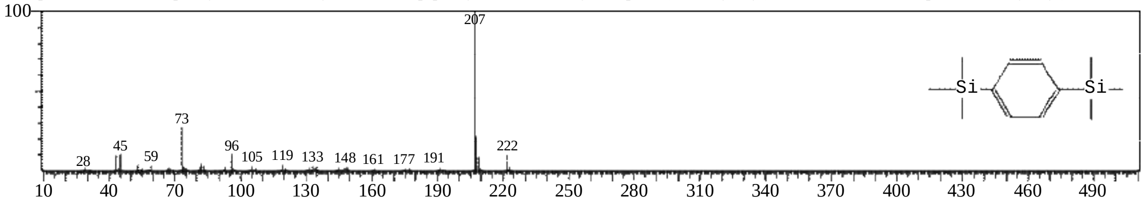

Hit#:5 Entry:54150 Library:NIST05.LIB
SI:55 Formula:C12H22Si2 CAS:17151-09-6 MolWeight:222 RetIndex:1124
CompName:1,2-Bis(trimethylsilyl)benzene $$ Trimethyl[2-(trimethylsilyl)phenyl]silane # $$

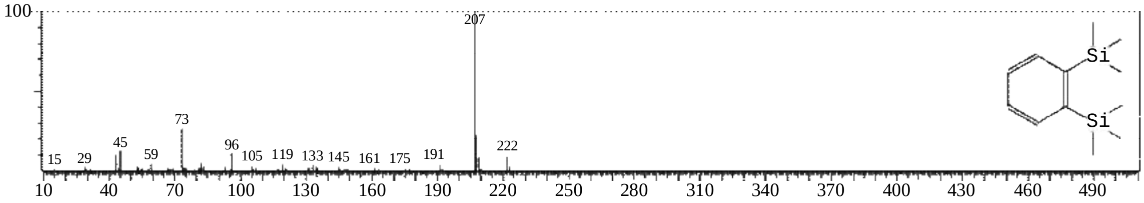

<< Target >>
Line#:56 R.Time:23.508(Scan#:2462) MassPeaks:304
RawMode:Averaged 23.500-23.517(2461-2463) BasePeak:57.10(128794)
BG Mode:Calc. from Peak Group 1 - Event 1

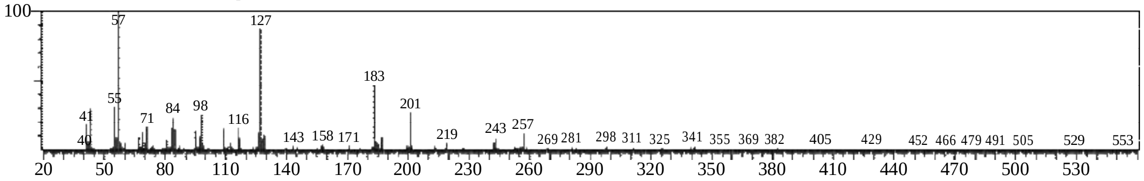

Hit#:1 Entry:153564 Library:NIST05.LIB
SI:73 Formula:C27H52O5 CAS:17598-94-6 MolWeight:456 RetIndex:3218
CompName:Dodecanoic acid, 1-(hydroxymethyl)-1,2-ethanediyl ester $$ 2-(Dodecanoyloxy)-1-(hydroxymethyl)ethyl laurate # $$

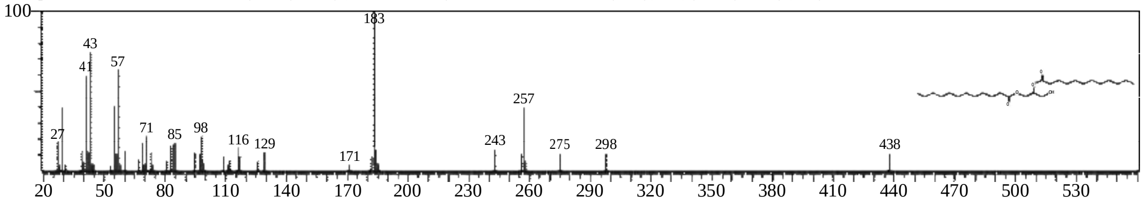

Hit#:2 Entry:115568 Library:NIST05.LIB
SI:72 Formula:C21H42O2 CAS:0-00-0 MolWeight:326 RetIndex:2212
CompName:Octanoic acid, 4-tridecyl ester

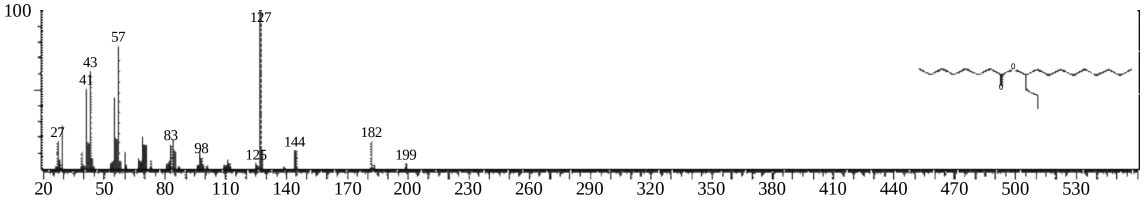

Hit#:3 Entry:115567 Library:NIST05.LIB
SI:71 Formula:C21H42O2 CAS:0-00-0 MolWeight:326 RetIndex:2212
CompName:Octanoic acid, 3-tridecyl ester

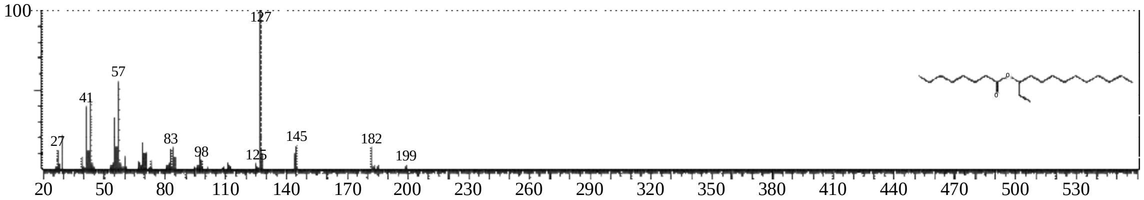

Hit#:4 Entry:57935 Library:NIST05.LIB
SI:71 Formula:C11H20N2O3 CAS:184637-49-8 MolWeight:228 RetIndex:1761
CompName:Azacyclohexane, 1-BOC-3-formamido- $$ tert-Butyl 3-(formylamino)-1-piperidinecarboxylate # $$

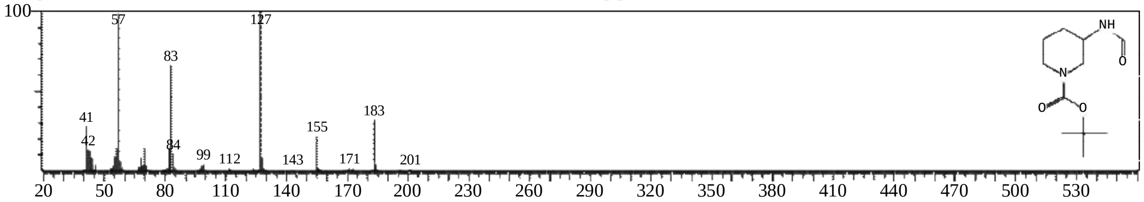

Hit#:5 Entry:75009 Library:NIST05.LIB
SI:71 Formula:C16H32O2 CAS:37811-72-6 MolWeight:256 RetIndex:1715
CompName:Isobutyl laurate $$ Lauric acid isobutyl ester $$ Dodecanoic acid, 2-methylpropyl ester $$

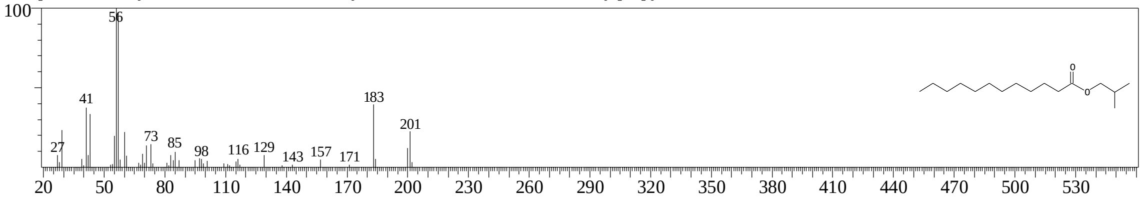

<< Target >>
Line#:57 R.Time:23.642(Scan#:2478) MassPeaks:333
RawMode:Averaged 23.633-23.650(2477-2479) BasePeak:127.15(239758)
BG Mode:Calc. from Peak Group 1 - Event 1

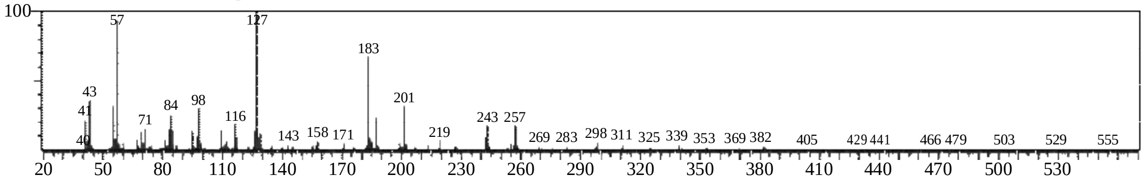

Hit#:1 Entry:153564 Library:NIST05.LIB
SI:73 Formula:C27H52O5 CAS:17598-94-6 MolWeight:456 RetIndex:3218
CompName:Dodecanoic acid, 1-(hydroxymethyl)-1,2-ethanediyl ester $$ 2-(Dodecanoyloxy)-1-(hydroxymethyl)ethyl laurate # $$

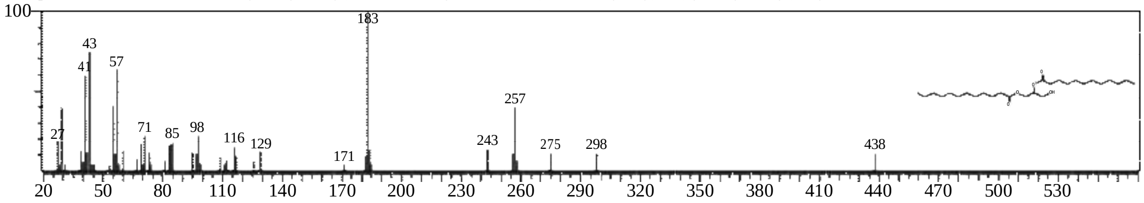

Hit#:2 Entry:27542 Library:NIST05s.LIB
SI:69 Formula:C39H74O6 CAS:538-24-9 MolWeight:638 RetIndex:4336
CompName:Dodecanoic acid, 1,2,3-propanetriyl ester $$ Laurin, tri- $$ Glycerol trilaurate $$ Glyceryl tridodecanoate $$ Glyceryl trilaurate $$ Lauric acid trigly

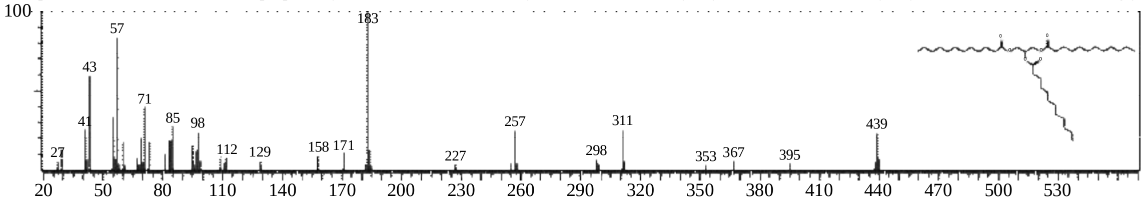

Hit#:3 Entry:115568 Library:NIST05.LIB
SI:69 Formula:C21H42O2 CAS:0-00-0 MolWeight:326 RetIndex:2212
CompName:Octanoic acid, 4-tridecyl ester

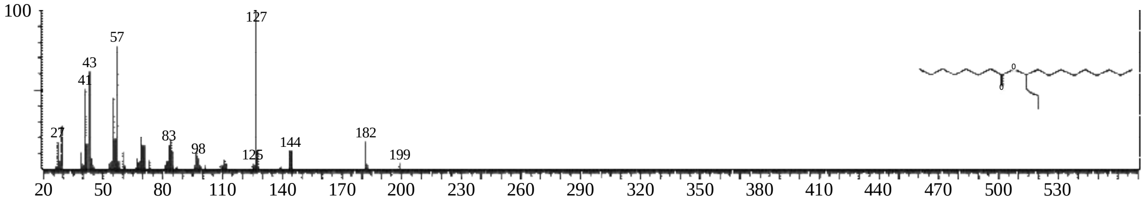

Hit#:4 Entry:115567 Library:NIST05.LIB
SI:68 Formula:C21H42O2 CAS:0-00-0 MolWeight:326 RetIndex:2212
CompName:Octanoic acid, 3-tridecyl ester

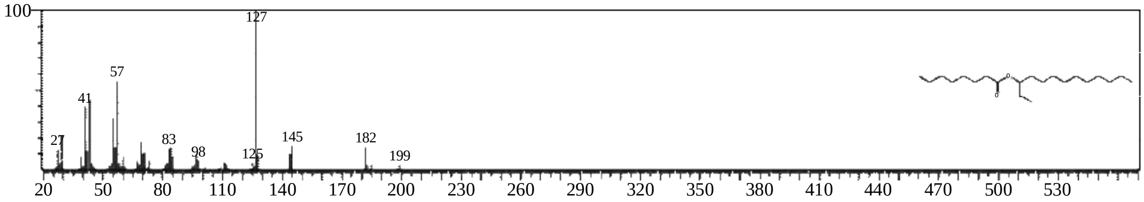

Hit#:5 Entry:57935 Library:NIST05.LIB
SI:68 Formula:C11H20N2O3 CAS:184637-49-8 MolWeight:228 RetIndex:1761
CompName:Azacyclohexane, 1-BOC-3-formamido- $$ tert-Butyl 3-(formylamino)-1-piperidinecarboxylate # $$

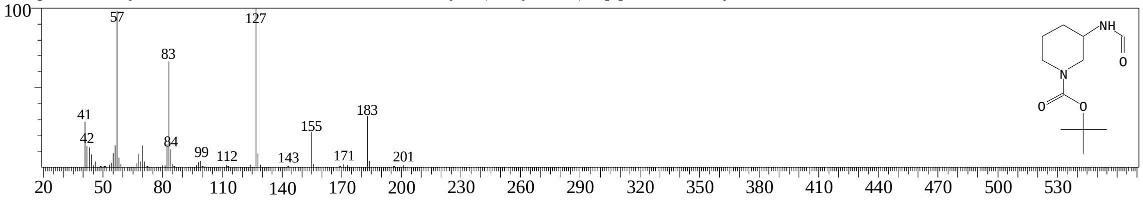

<< Target >>
Line#:58 R.Time:23.925(Scan#:2512) MassPeaks:215
RawMode:Averaged 23.917-23.933(2511-2513) BasePeak:73.00(9382)
BG Mode:Calc. from Peak Group 1 - Event 1

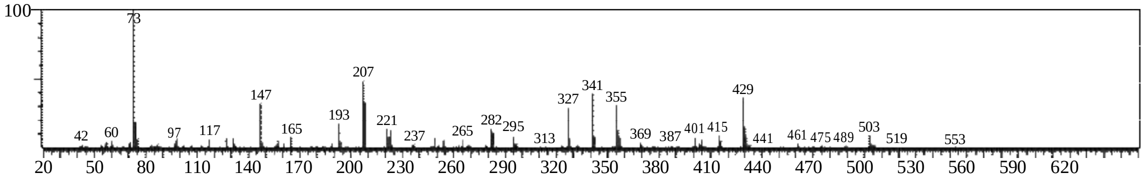

Hit#:1 Entry:162013 Library:NIST05.LIB
SI:69 Formula:C18H54O9Si9 CAS:556-71-8 MolWeight:666 RetIndex:1860
CompName:Cyclononasiloxane, octadecamethyl- $$ Octadeamethyl-cyclononasiloxane $$ 2,2,4,4,6,6,8,8,10,10,12,12,14,14,16,16,18,18-Octadecamethylcyclon

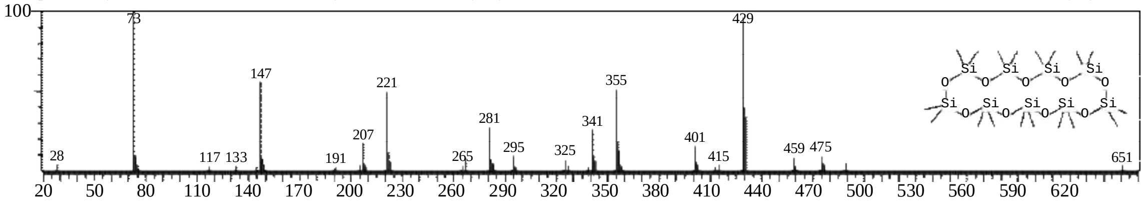

Hit#:2 Entry:162623 Library:NIST05.LIB
SI:68 Formula:C20H60O10Si10 CAS:18772-36-6 MolWeight:740 RetIndex:2067
CompName:Cyclodecasiloxane, eicosamethyl- $$ 2,2,4,4,6,6,8,8,10,10,12,12,14,14,16,16,18,18,20,20-Icosamethylcyclodecasiloxane # $$

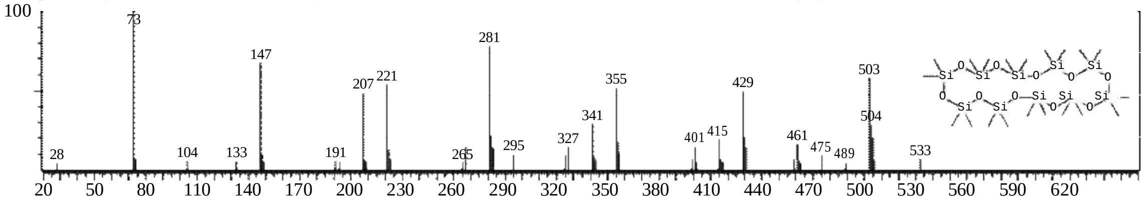

Hit#:3 Entry:27563 Library:NIST05s.LIB
SI:67 Formula:C18H54O9Si9 CAS:556-71-8 MolWeight:666 RetIndex:1860
CompName:Cyclononasiloxane, octadecamethyl- $$ Octadeamethyl-cyclononasiloxane $$ 2,2,4,4,6,6,8,8,10,10,12,12,14,14,16,16,18,18-Octadecamethylcyclon

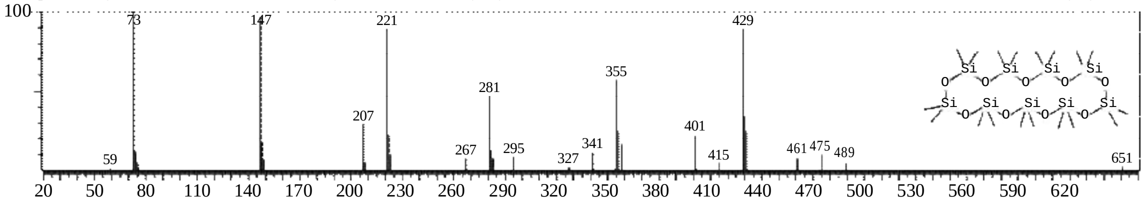

Hit#:4 Entry:160407 Library:NIST05.LIB
SI:66 Formula:C16H50O7Si8 CAS:19095-24-0 MolWeight:578 RetIndex:1710
CompName:Octasiloxane, 1,1,3,3,5,5,7,7,9,9,11,11,13,13,15,15-hexadecamethyl- $$ 1,1,3,3,5,5,7,7,9,9,11,11,13,13,15,15-Hexadecamethyloctasiloxane # $$

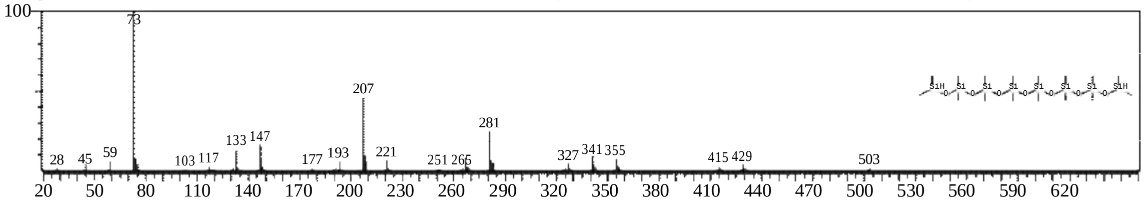

Hit#:5 Entry:158804 Library:NIST05.LIB
SI:66 Formula:C16H48O6Si7 CAS:541-01-5 MolWeight:532 RetIndex:1437
CompName:Heptasiloxane, hexadecamethyl- $$ Hexadecamethylheptasiloxane $$ 1,1,1,3,3,5,5,7,7,9,9,11,11,13,13,13-Hexadecamethylheptasiloxane # $$

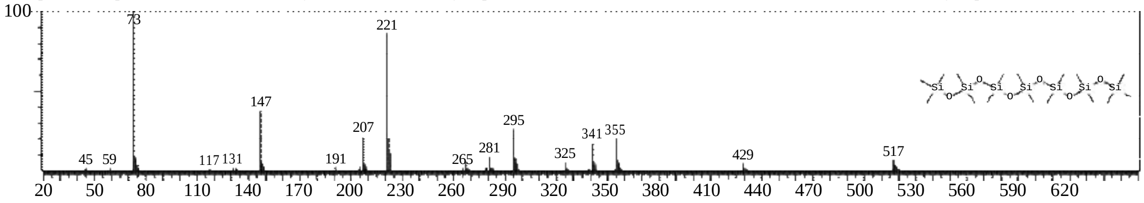

<< Target >>
Line#:59 R.Time:24.067(Scan#:2529) MassPeaks:155
RawMode:Averaged 24.058-24.075(2528-2530) BasePeak:73.10(16974)
BG Mode:Calc. from Peak Group 1 - Event 1

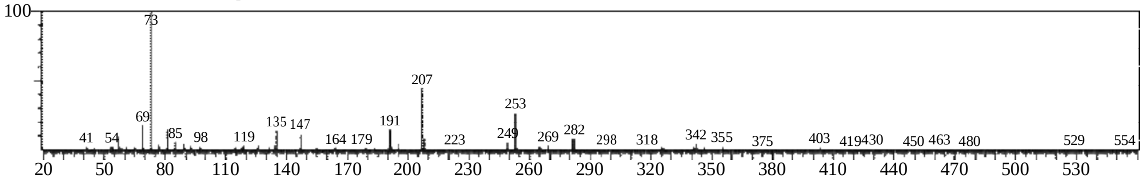

Hit#:1 Entry:157431 Library:NIST05.LIB
SI:65 Formula:C14H44O6Si7 CAS:19095-23-9 MolWeight:504 RetIndex:1526
CompName:Heptasiloxane, 1,1,3,3,5,5,7,7,9,9,11,11,13,13-tetradecamethyl- $$ 1,1,3,3,5,5,7,7,9,9,11,11,13,13-Tetradecamethylheptasiloxane # $$

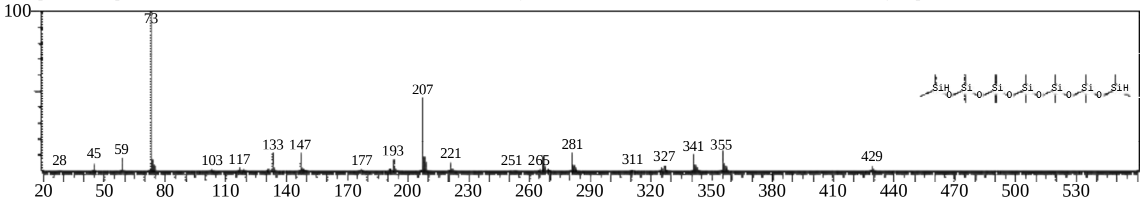

Hit#:2 Entry:150228 Library:NIST05.LIB
SI:62 Formula:C12H38O5Si6 CAS:995-82-4 MolWeight:430 RetIndex:1341
CompName:Hexasiloxane, 1,1,3,3,5,5,7,7,9,9,11,11-dodecamethyl- $$ 1,1,3,3,5,5,7,7,9,9,11,11-Dodecamethylhexasiloxane # $$

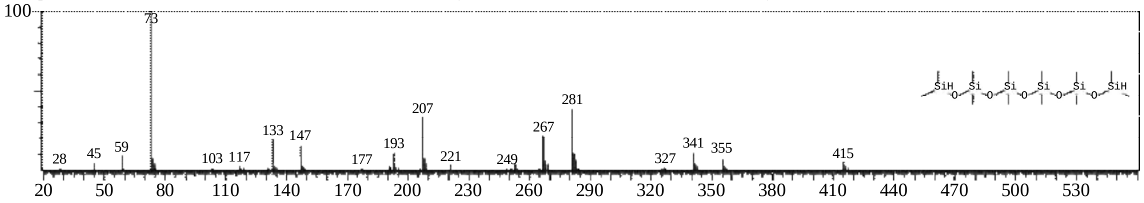

Hit#:3 Entry:160407 Library:NIST05.LIB
SI:62 Formula:C16H50O7Si8 CAS:19095-24-0 MolWeight:578 RetIndex:1710
CompName:Octasiloxane, 1,1,3,3,5,5,7,7,9,9,11,11,13,13,15,15-hexadecamethyl- $$ 1,1,3,3,5,5,7,7,9,9,11,11,13,13,15,15-Hexadecamethyloctasiloxane # $$

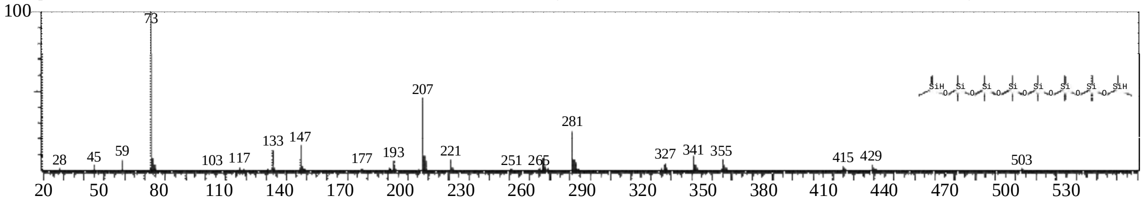

Hit#:4 Entry:88693 Library:NIST05.LIB
SI:59 Formula:C7H24O2Si5 CAS:0-00-0 MolWeight:280 RetIndex:0
CompName:2,2,3,5,6,6,7-Heptamethyl[1,4,2,3,5,6,7] dioxapentasilepane

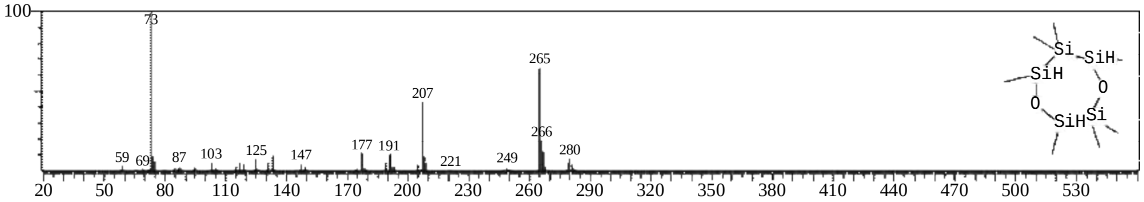

Hit#:5 Entry:98467 Library:NIST05.LIB
SI:58 Formula:C15H32Si3 CAS:0-00-0 MolWeight:296 RetIndex:1222
CompName:1,4-Cyclohexadiene, 1,3,6-tris(trimethylsilyl)-

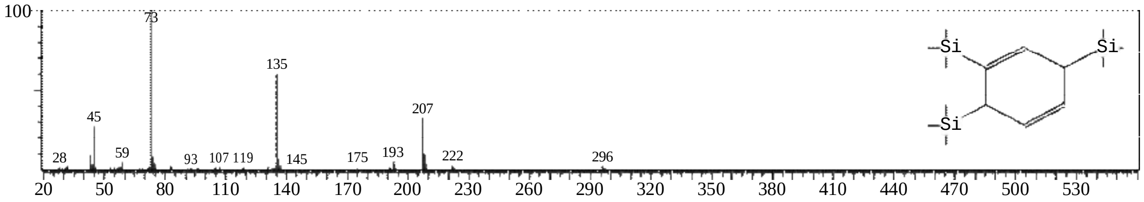

<< Target >>
Line#:60 R.Time:24.417(Scan#:2571) MassPeaks:167
RawMode:Averaged 24.408-24.425(2570-2572) BasePeak:207.05(16760)
BG Mode:Calc. from Peak Group 1 - Event 1

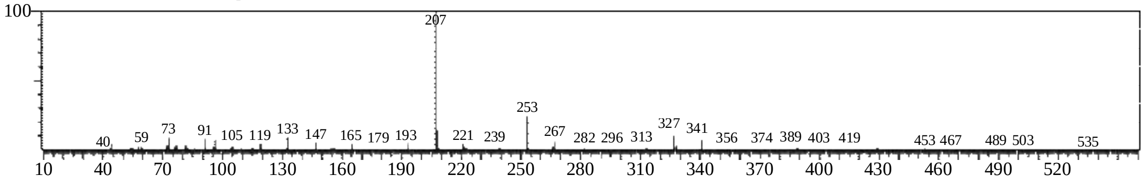

Hit#:1 Entry:53689 Library:NIST05.LIB
SI:64 Formula:C6H18O3Si3 CAS:541-05-9 MolWeight:222 RetIndex:620
CompName:Cyclotrisiloxane, hexamethyl- $$ Dimethylsiloxane cyclic trimer $$ Hexamethylcyclotrisiloxane $$ CH7260 $$ 2,2,4,4,6,6-Hexamethyl-1,3,5,2,4,6-

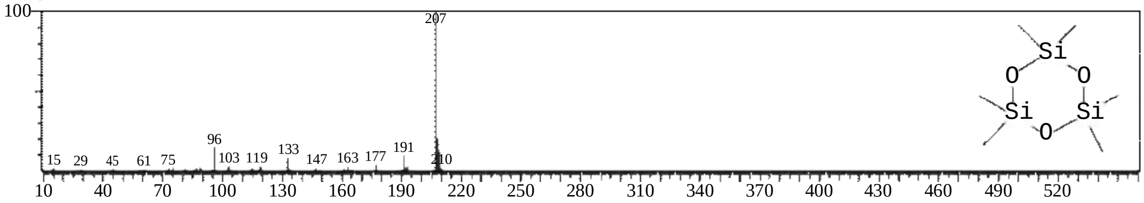

Hit#:2 Entry:18524 Library:NIST05s.LIB
SI:64 Formula:C6H18O3Si3 CAS:541-05-9 MolWeight:222 RetIndex:620
CompName:Cyclotrisiloxane, hexamethyl- $$ Dimethylsiloxane cyclic trimer $$ Hexamethylcyclotrisiloxane $$ CH7260 $$ 2,2,4,4,6,6-Hexamethyl-1,3,5,2,4,6-

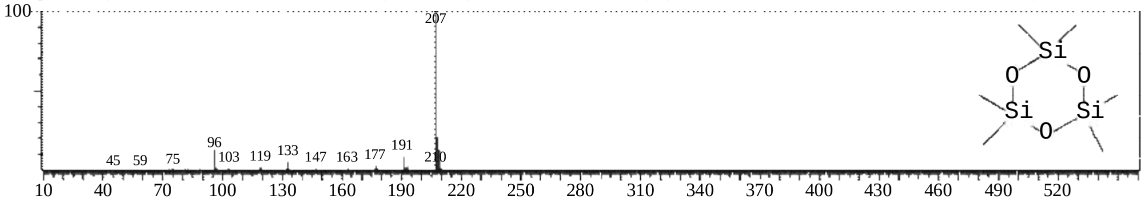

Hit#:3 Entry:54242 Library:NIST05.LIB
SI:63 Formula:C13H18O3 CAS:54889-98-4 MolWeight:222 RetIndex:1563
CompName:Methanol, [4-(1,1-dimethylethyl)phenoxy]-, acetate $$ (4-tert-Butylphenoxy)methyl acetate # $$

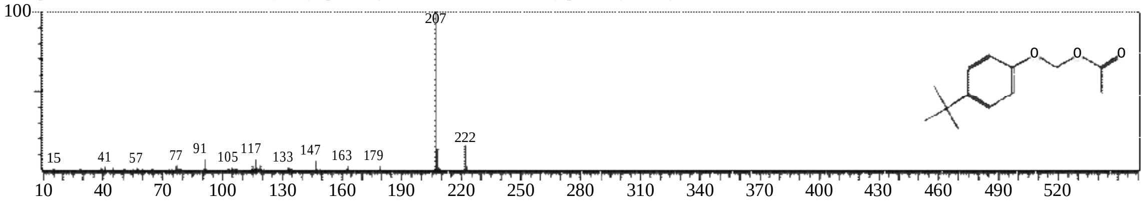

Hit#:4 Entry:54151 Library:NIST05.LIB
SI:63 Formula:C12H22Si2 CAS:13183-70-5 MolWeight:222 RetIndex:1124
CompName:Silane, 1,4-phenylenebis[trimethyl- $$ Silane, p-phenylenebis[trimethyl- $$ p-Bis(trimethylsilyl)benzene $$ Benzene, p-bis(trimethylsilyl)- $$ 1,4-

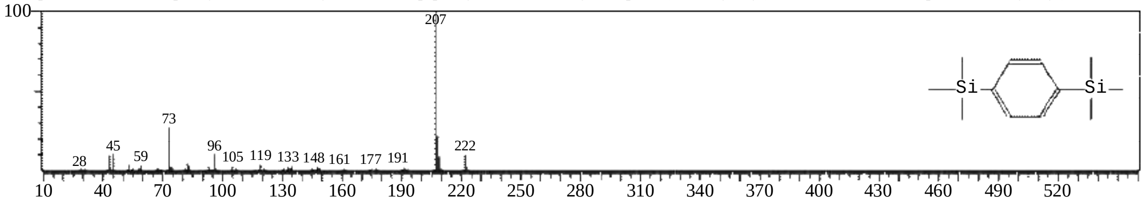

Hit#:5 Entry:54150 Library:NIST05.LIB
SI:62 Formula:C12H22Si2 CAS:17151-09-6 MolWeight:222 RetIndex:1124
CompName:1,2-Bis(trimethylsilyl)benzene $$ Trimethyl[2-(trimethylsilyl)phenyl]silane # $$

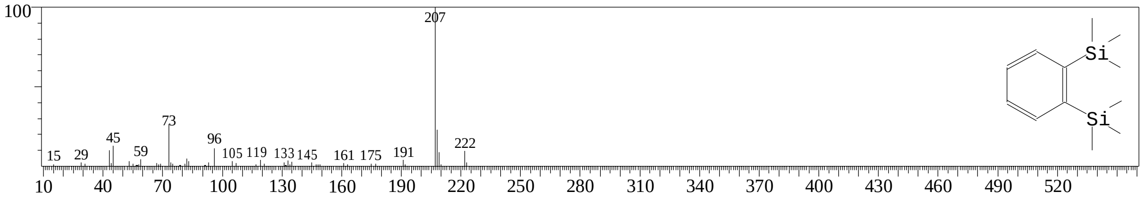

<< Target >>
Line#:61 R.Time:24.933(Scan#:2633) MassPeaks:313
RawMode:Averaged 24.925-24.942(2632-2634) BasePeak:57.05(35915)
BG Mode:Calc. from Peak Group 1 - Event 1

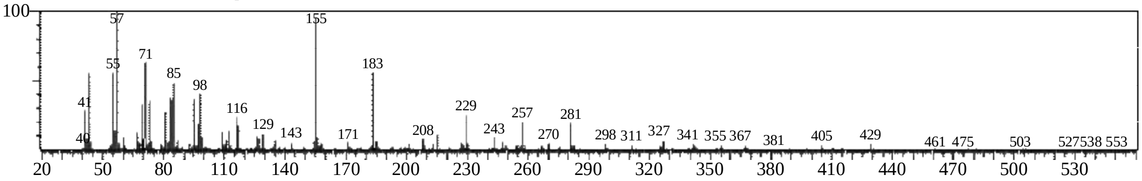

Hit#:1 Entry:162815 Library:NIST05.LIB
SI:74 Formula:C49H94O6 CAS:56846-96-9 MolWeight:778 RetIndex:5330
CompName:Octadecanoic acid, 2,3-bis[(1-oxotetradecyl)oxy]propyl ester $$ 2,3-Bis(tetradecanoyloxy)propyl stearate # $$

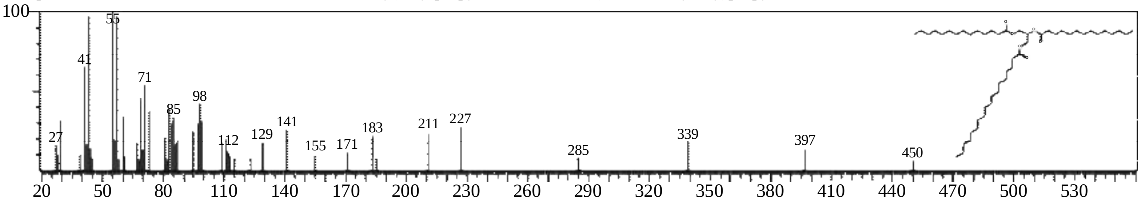

Hit#:2 Entry:162295 Library:NIST05.LIB
SI:73 Formula:C37H74NO8P CAS:3026-45-7 MolWeight:691 RetIndex:0
CompName:Hexadecanoic acid, 1-[[[(2-aminoethoxy)hydroxyphosphinyl]oxy]methyl]-1,2-ethanediyl ester $$ Palmitin, 1,2-di-, 2-aminoethyl hydrogen phospha

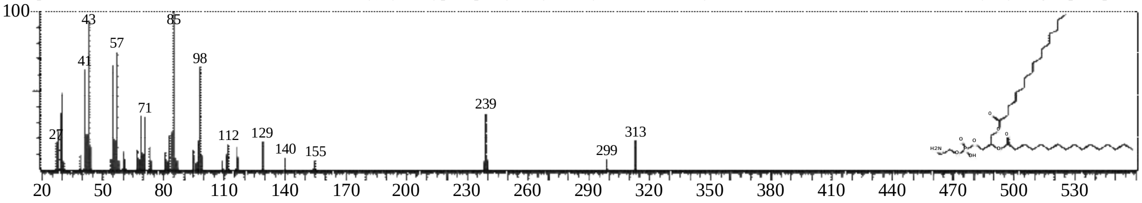

Hit#:3 Entry:68617 Library:NIST05.LIB
SI:72 Formula:C13H26O4 CAS:3376-48-5 MolWeight:246 RetIndex:1902
CompName:Decanoic acid, 2-hydroxy-1-(hydroxymethyl)ethyl ester $$ Decanoin, 2-mono- $$ Decanoic acid 2-monoglyceride $$ 2-Hydroxy-1-(hydroxymethyl)

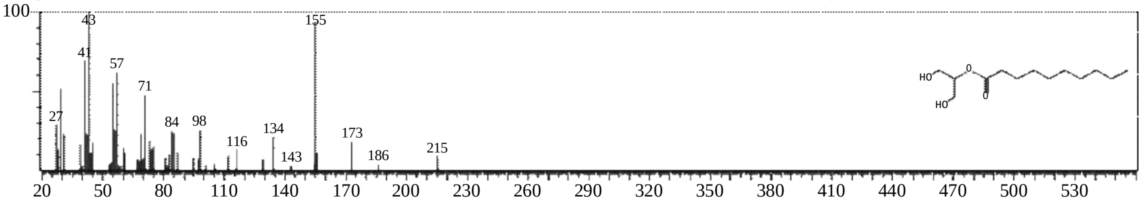

Hit#:4 Entry:153564 Library:NIST05.LIB
SI:72 Formula:C27H52O5 CAS:17598-94-6 MolWeight:456 RetIndex:3218
CompName:Dodecanoic acid, 1-(hydroxymethyl)-1,2-ethanediyl ester $$ 2-(Dodecanoyloxy)-1-(hydroxymethyl)ethyl laurate # $$

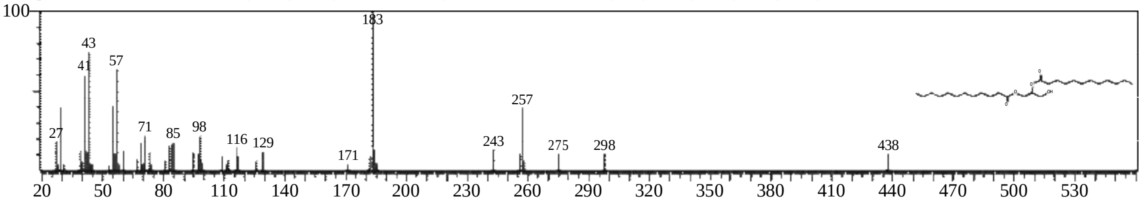

Hit#:5 Entry:137704 Library:NIST05.LIB
SI:71 Formula:C21H44O3S CAS:0-00-0 MolWeight:376 RetIndex:2668
CompName:Sulfurous acid, octadecyl 2-propyl ester

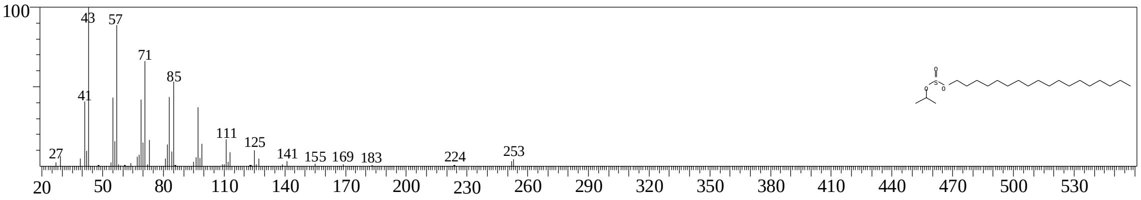

<< Target >>
Line#:62 R.Time:25.108(Scan#:2654) MassPeaks:340
RawMode:Averaged 25.100-25.117(2653-2655) BasePeak:57.10(85082)
BG Mode:Calc. from Peak Group 1 - Event 1

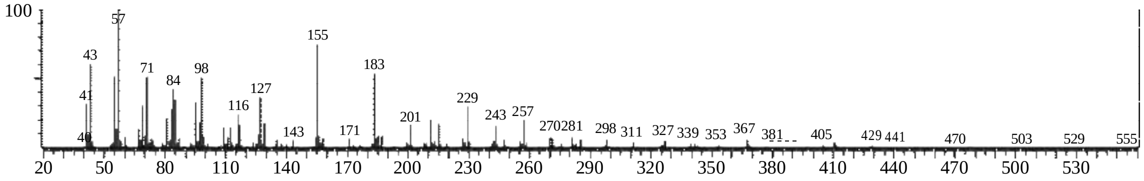

Hit#:1 Entry:162815 Library:NIST05.LIB
SI:72 Formula:C49H94O6 CAS:56846-96-9 MolWeight:778 RetIndex:5330
CompName:Octadecanoic acid, 2,3-bis[(1-oxotetradecyl)oxy]propyl ester $$ 2,3-Bis(tetradecanoyloxy)propyl stearate # $$

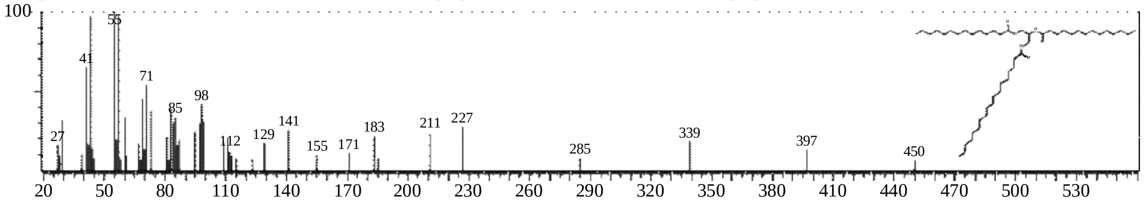

Hit#:2 Entry:153564 Library:NIST05.LIB
SI:70 Formula:C27H52O5 CAS:17598-94-6 MolWeight:456 RetIndex:3218
CompName:Dodecanoic acid, 1-(hydroxymethyl)-1,2-ethanediyl ester $$ 2-(Dodecanoyloxy)-1-(hydroxymethyl)ethyl laurate # $$

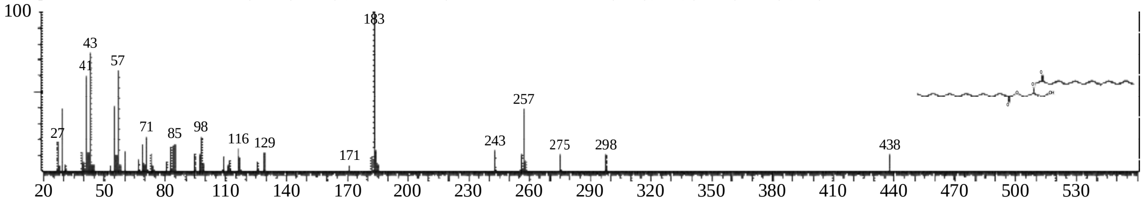

Hit#:3 Entry:27542 Library:NIST05s.LIB
SI:69 Formula:C39H74O6 CAS:538-24-9 MolWeight:638 RetIndex:4336
CompName:Dodecanoic acid, 1,2,3-propanetriyl ester $$ Laurin, tri- $$ Glycerol trilaurate $$ Glyceryl tridodecanoate $$ Glyceryl trilaurate $$ Lauric acid trigly

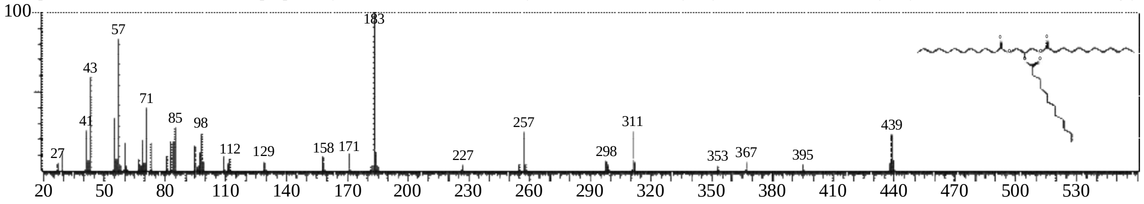

Hit#:4 Entry:162295 Library:NIST05.LIB
SI:69 Formula:C37H74NO8P CAS:3026-45-7 MolWeight:691 RetIndex:0
CompName:Hexadecanoic acid, 1-[[[(2-aminoethoxy)hydroxyphosphinyl]oxy]methyl]-1,2-ethanediyl ester $$ Palmitin, 1,2-di-, 2-aminoethyl hydrogen phospha

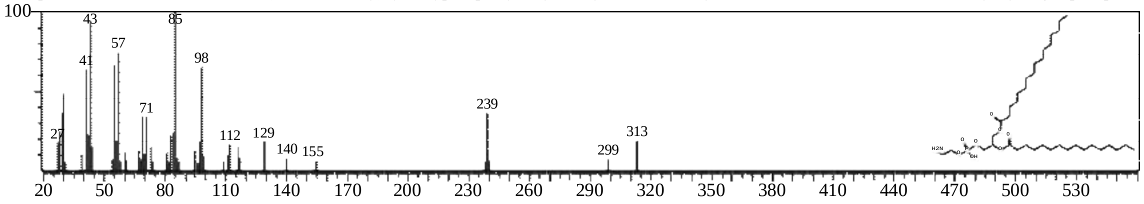

Hit#:5 Entry:27543 Library:NIST05s.LIB
SI:69 Formula:C39H74O6 CAS:538-24-9 MolWeight:638 RetIndex:4336
CompName:Dodecanoic acid, 1,2,3-propanetriyl ester $$ Laurin, tri- $$ Glycerol trilaurate $$ Glyceryl tridodecanoate $$ Glyceryl trilaurate $$ Lauric acid trigly

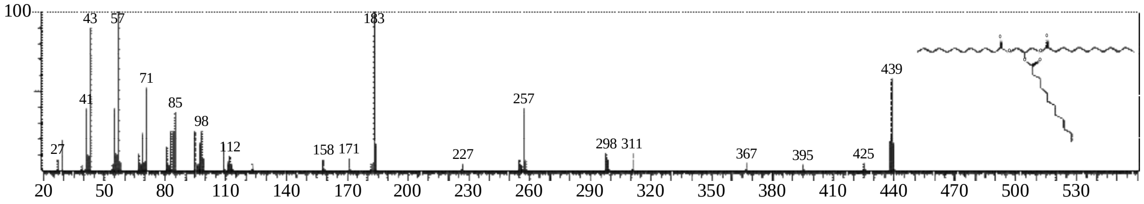

<< Target >>
Line#:63 R.Time:25.500(Scan#:2701) MassPeaks:155
RawMode:Averaged 25.492-25.508(2700-2702) BasePeak:207.05(6523)
BG Mode:Calc. from Peak Group 1 - Event 1

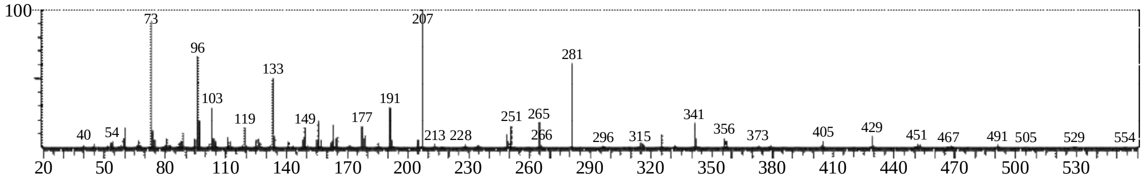

Hit#:1 Entry:122175 Library:NIST05.LIB
SI:56 Formula:C11H32O4Si4 CAS:18030-67-6 MolWeight:340 RetIndex:1058
CompName:3-Ethoxy-1,1,1,5,5,5-hexamethyl-3-(trimethylsiloxy)trisiloxane $$ 3-Ethoxy-1,1,1,5,5,5-hexamethyl-3-(trimethylsilyloxy)trisiloxane $$ Ethyl tris(tr

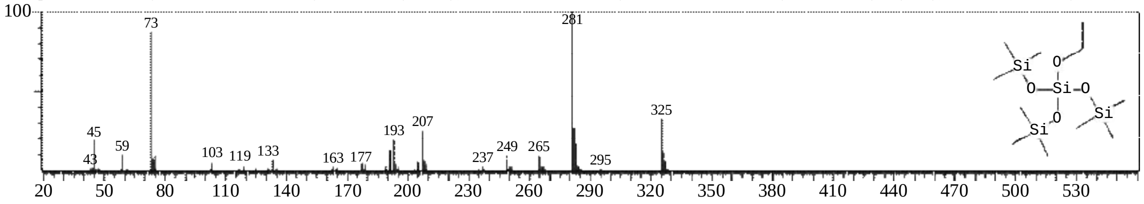

Hit#:2 Entry:23533 Library:NIST05s.LIB
SI:54 Formula:C8H24O4Si4 CAS:556-67-2 MolWeight:296 RetIndex:827
CompName:Cyclotetrasiloxane, octamethyl- $$ Oktamethylcyklotetrasiloxan $$ NUC Silicone VS 7207 $$ CO9810 $$ O9810 $$ Octamethyltetrasiloxane $$ 2,

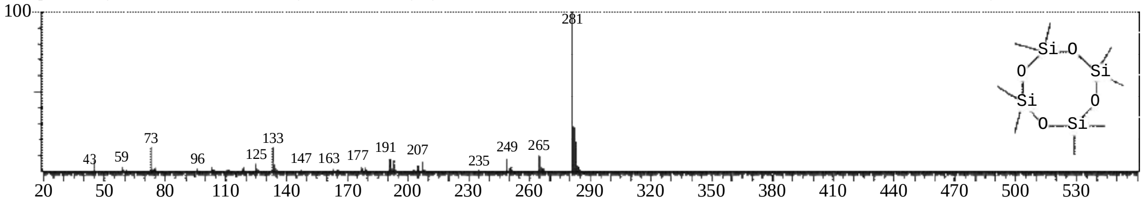

Hit#:3 Entry:88721 Library:NIST05.LIB
SI:54 Formula:C9H28O2Si4 CAS:139347-50-5 MolWeight:280 RetIndex:851
CompName:Trimethylsilyl-di(timethylsiloxy)-silane $$ 1,1,1,5,5,5-Hexamethyl-3-(trimethylsilyl)trisiloxane # $$

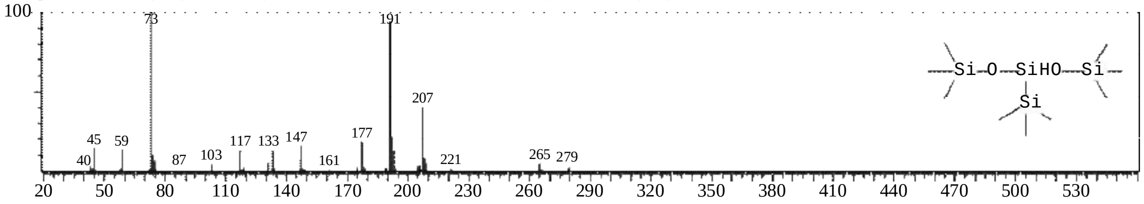

Hit#:4 Entry:160407 Library:NIST05.LIB
SI:54 Formula:C16H50O7Si8 CAS:19095-24-0 MolWeight:578 RetIndex:1710
CompName:Octasiloxane, 1,1,3,3,5,5,7,7,9,9,11,11,13,13,15,15-hexadecamethyl- $$ 1,1,3,3,5,5,7,7,9,9,11,11,13,13,15,15-Hexadecamethyloctasiloxane # $$

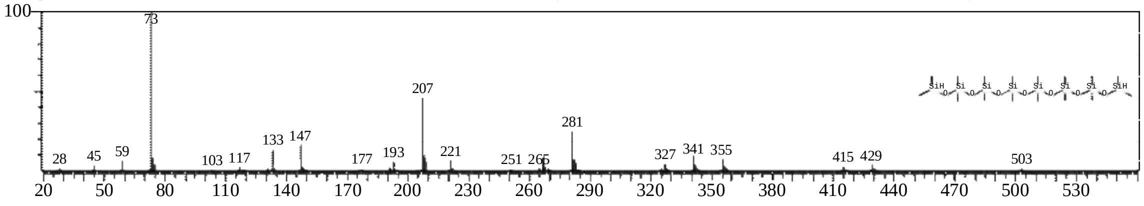

Hit#:5 Entry:88693 Library:NIST05.LIB
SI:54 Formula:C7H24O2Si5 CAS:0-00-0 MolWeight:280 RetIndex:0
CompName:2,2,3,5,6,6,7-Heptamethyl[1,4,2,3,5,6,7] dioxapentasilepane

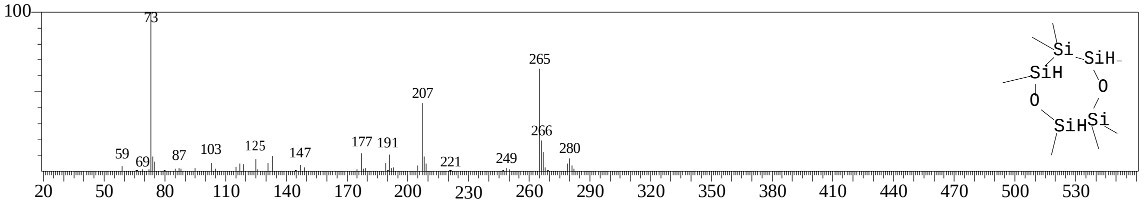

<< Target >>
Line#:64 R.Time:26.008(Scan#:2762) MassPeaks:195
RawMode:Averaged 26.000-26.017(2761-2763) BasePeak:207.00(12700)
BG Mode:Calc. from Peak Group 1 - Event 1

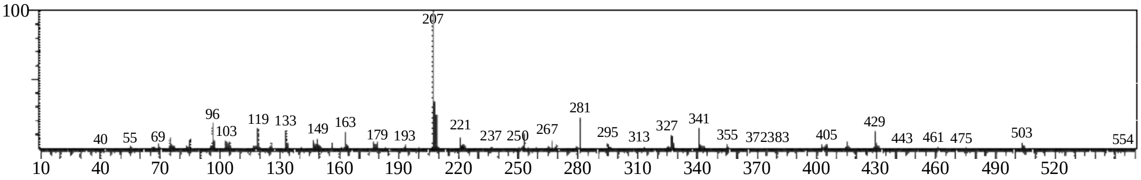

Hit#:1 Entry:53689 Library:NIST05.LIB
SI:62 Formula:C6H18O3Si3 CAS:541-05-9 MolWeight:222 RetIndex:620
CompName:Cyclotrisiloxane, hexamethyl- $$ Dimethylsiloxane cyclic trimer $$ Hexamethylcyclotrisiloxane $$ CH7260 $$ 2,2,4,4,6,6-Hexamethyl-1,3,5,2,4,6-

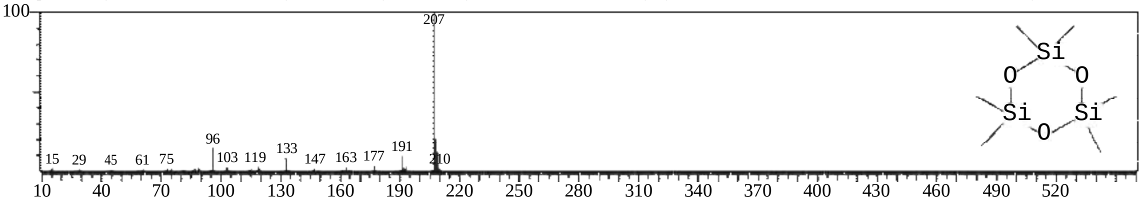

Hit#:2 Entry:18524 Library:NIST05s.LIB
SI:61 Formula:C6H18O3Si3 CAS:541-05-9 MolWeight:222 RetIndex:620
CompName:Cyclotrisiloxane, hexamethyl- $$ Dimethylsiloxane cyclic trimer $$ Hexamethylcyclotrisiloxane $$ CH7260 $$ 2,2,4,4,6,6-Hexamethyl-1,3,5,2,4,6-

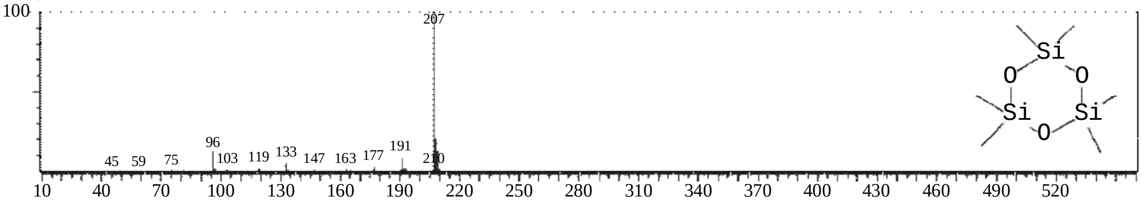

Hit#:3 Entry:54030 Library:NIST05.LIB
SI:60 Formula:C11H18N2OSi CAS:17993-84-9 MolWeight:222 RetIndex:0
CompName:N,N-Dimethyl-4-nitroso-3-(trimethylsilyl)aniline

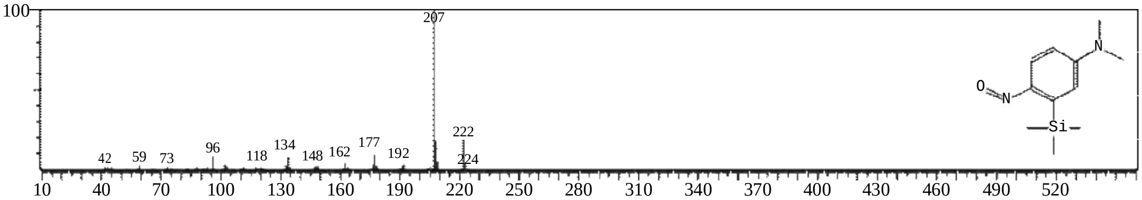

Hit#:4 Entry:98224 Library:NIST05.LIB
SI:60 Formula:C10H28O4Si3 CAS:3555-45-1 MolWeight:296 RetIndex:1049
CompName:Silicic acid, diethyl bis(trimethylsilyl) ester $$ 3,3-Diethoxy-1,1,1,5,5,5-hexamethyltrisiloxane $$ Diethyl bis(trimethylsilyl) orthosilicate # $$

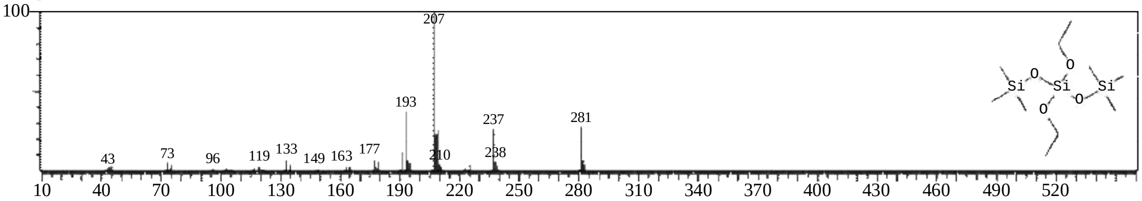

Hit#:5 Entry:18523 Library:NIST05s.LIB
SI:58 Formula:C6H18O3Si3 CAS:541-05-9 MolWeight:222 RetIndex:620
CompName:Cyclotrisiloxane, hexamethyl- $$ Dimethylsiloxane cyclic trimer $$ Hexamethylcyclotrisiloxane $$ CH7260 $$ 2,2,4,4,6,6-Hexamethyl-1,3,5,2,4,6-

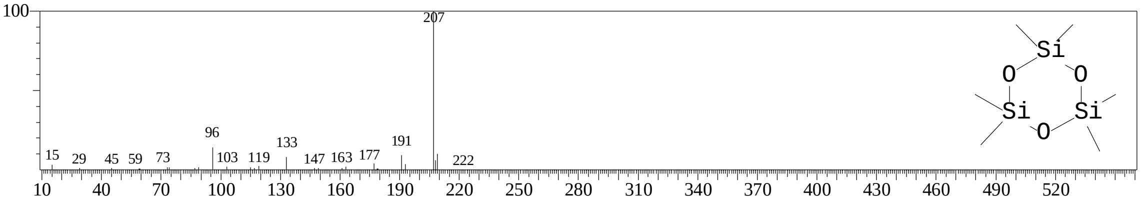

<< Target >>
Line#:65 R.Time:26.708(Scan#:2846) MassPeaks:349
RawMode:Averaged 26.700-26.717(2845-2847) BasePeak:183.20(116048)
BG Mode:Calc. from Peak Group 1 - Event 1

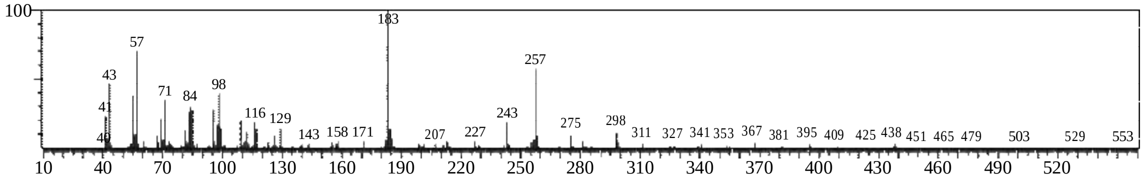

Hit#:1 Entry:153564 Library:NIST05.LIB
SI:87 Formula:C27H52O5 CAS:17598-94-6 MolWeight:456 RetIndex:3218
CompName:Dodecanoic acid, 1-(hydroxymethyl)-1,2-ethanediyl ester $$ 2-(Dodecanoyloxy)-1-(hydroxymethyl)ethyl laurate # $$

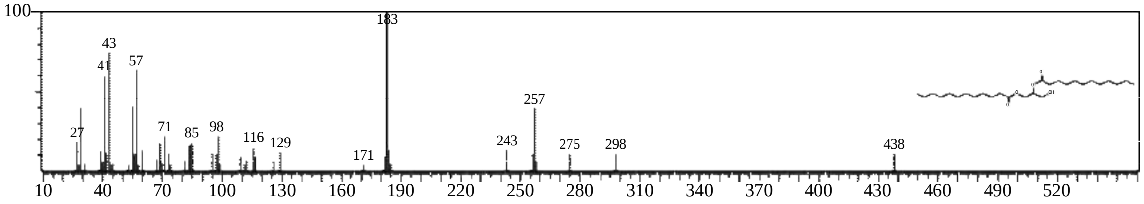

Hit#:2 Entry:27542 Library:NIST05s.LIB
SI:82 Formula:C39H74O6 CAS:538-24-9 MolWeight:638 RetIndex:4336
CompName:Dodecanoic acid, 1,2,3-propanetriyl ester $$ Laurin, tri- $$ Glycerol trilaurate $$ Glyceryl tridodecanoate $$ Glyceryl trilaurate $$ Lauric acid trigly

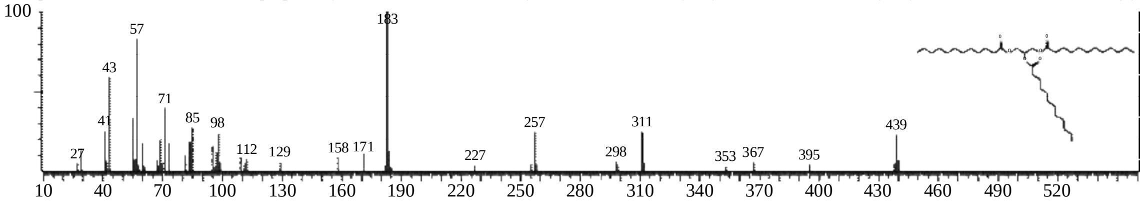

Hit#:3 Entry:27543 Library:NIST05s.LIB
SI:81 Formula:C39H74O6 CAS:538-24-9 MolWeight:638 RetIndex:4336
CompName:Dodecanoic acid, 1,2,3-propanetriyl ester $$ Laurin, tri- $$ Glycerol trilaurate $$ Glyceryl tridodecanoate $$ Glyceryl trilaurate $$ Lauric acid trigly

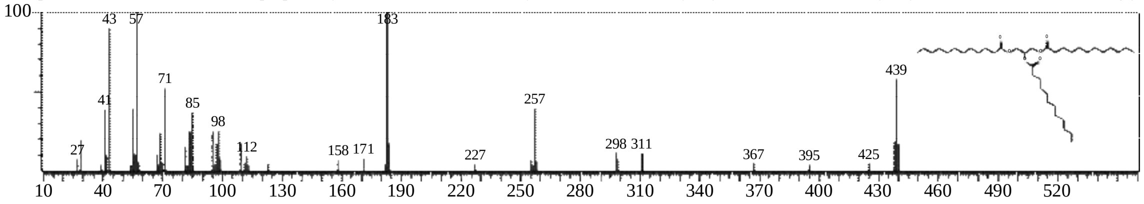

Hit#:4 Entry:161659 Library:NIST05.LIB
SI:78 Formula:C39H74O6 CAS:538-24-9 MolWeight:638 RetIndex:4336
CompName:Dodecanoic acid, 1,2,3-propanetriyl ester $$ Laurin, tri- $$ Glycerol trilaurate $$ Glyceryl tridodecanoate $$ Glyceryl trilaurate $$ Lauric acid trigly

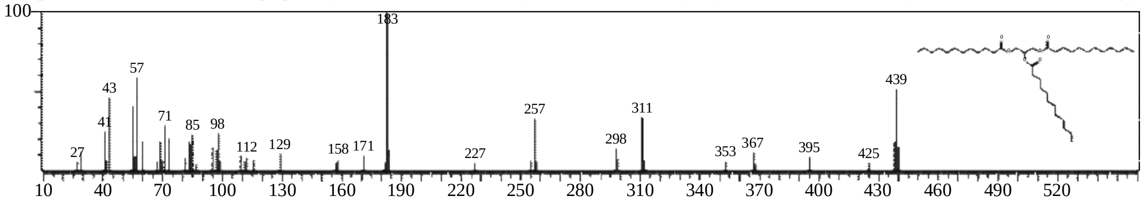

Hit#:5 Entry:57042 Library:NIST05.LIB
SI:74 Formula:C14H26O2 CAS:2146-71-6 MolWeight:226 RetIndex:1570
CompName:Dodecanoic acid, ethenyl ester $$ Lauric acid, vinyl ester $$ Vinyl laurate $$ Vinyl dodecanoate $$

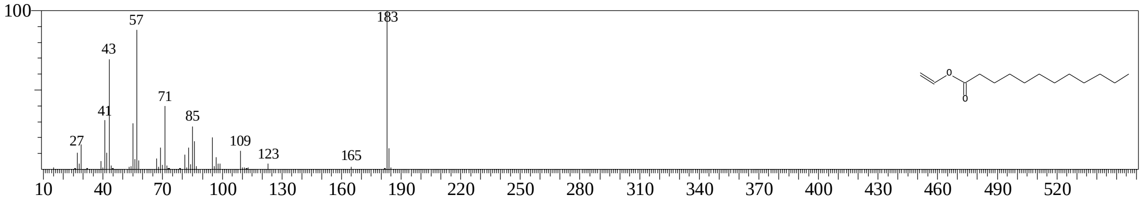

<< Target >>
Line#:66 R.Time:26.950(Scan#:2875) MassPeaks:366
RawMode:Averaged 26.942-26.958(2874-2876) BasePeak:183.20(258185)
BG Mode:Calc. from Peak Group 1 - Event 1

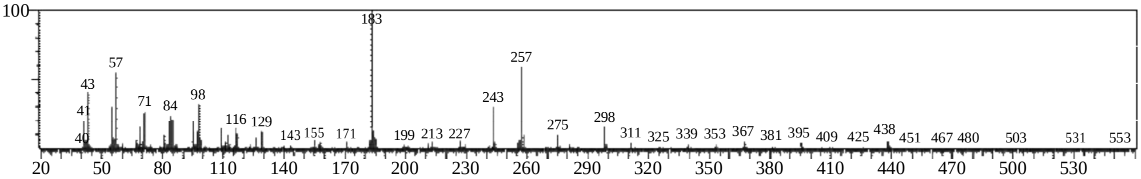

Hit#:1 Entry:153564 Library:NIST05.LIB
SI:87 Formula:C27H52O5 CAS:17598-94-6 MolWeight:456 RetIndex:3218
CompName:Dodecanoic acid, 1-(hydroxymethyl)-1,2-ethanediyl ester $$ 2-(Dodecanoyloxy)-1-(hydroxymethyl)ethyl laurate # $$

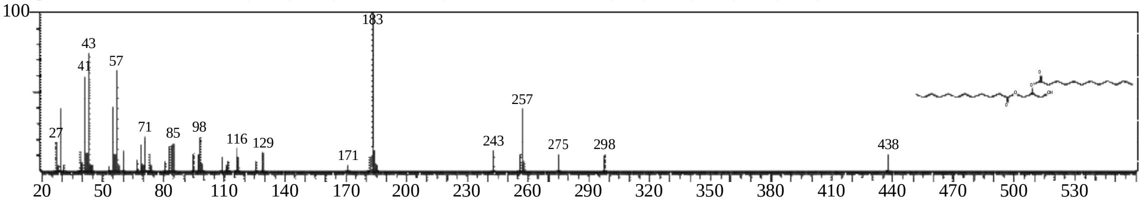

Hit#:2 Entry:27542 Library:NIST05s.LIB
SI:81 Formula:C39H74O6 CAS:538-24-9 MolWeight:638 RetIndex:4336
CompName:Dodecanoic acid, 1,2,3-propanetriyl ester $$ Laurin, tri- $$ Glycerol trilaurate $$ Glyceryl tridodecanoate $$ Glyceryl trilaurate $$ Lauric acid trigly

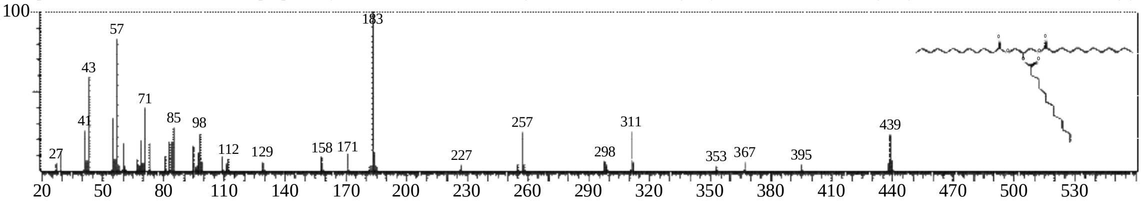

Hit#:3 Entry:161659 Library:NIST05.LIB
SI:80 Formula:C39H74O6 CAS:538-24-9 MolWeight:638 RetIndex:4336
CompName:Dodecanoic acid, 1,2,3-propanetriyl ester $$ Laurin, tri- $$ Glycerol trilaurate $$ Glyceryl tridodecanoate $$ Glyceryl trilaurate $$ Lauric acid trigly

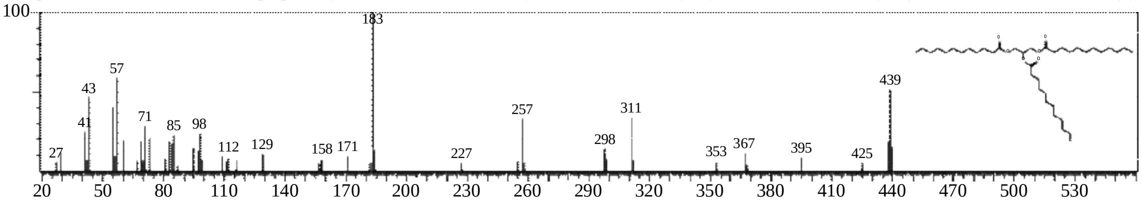

Hit#:4 Entry:27543 Library:NIST05s.LIB
SI:78 Formula:C39H74O6 CAS:538-24-9 MolWeight:638 RetIndex:4336
CompName:Dodecanoic acid, 1,2,3-propanetriyl ester $$ Laurin, tri- $$ Glycerol trilaurate $$ Glyceryl tridodecanoate $$ Glyceryl trilaurate $$ Lauric acid trigly

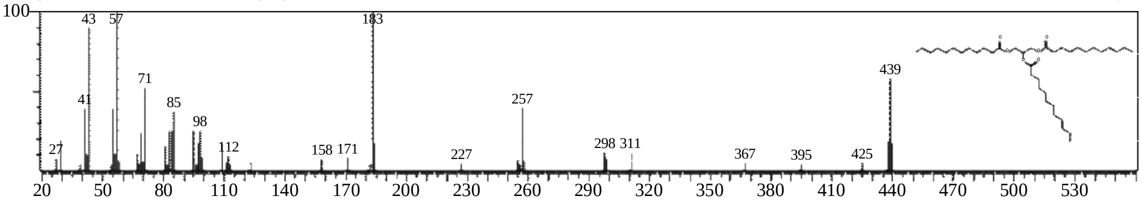

Hit#:5 Entry:161368 Library:NIST05.LIB
SI:73 Formula:C32H64NO8P CAS:18194-25-7 MolWeight:621 RetIndex:0
CompName:3,5,9-Trioxa-4-phosphaheneicosan-1-aminium, 4-hydroxy-N,N,N-trimethyl-10-oxo-7-[(1-oxododecyl)oxy]-, hydroxide, inner salt, 4-oxide, (R)- $$

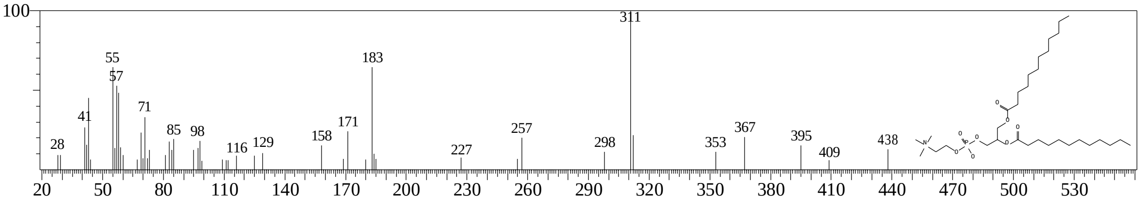

<< Target >>
Line#:67 R.Time:27.333(Scan#:2921) MassPeaks:198
RawMode:Averaged 27.325-27.342(2920-2922) BasePeak:73.05(11462)
BG Mode:Calc. from Peak Group 1 - Event 1

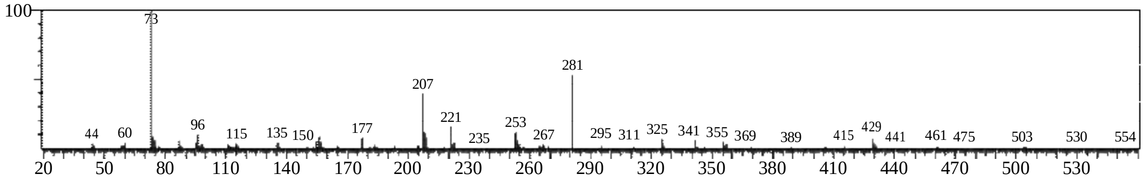

Hit#:1 Entry:160407 Library:NIST05.LIB
SI:69 Formula:C16H50O7Si8 CAS:19095-24-0 MolWeight:578 RetIndex:1710
CompName:Octasiloxane, 1,1,3,3,5,5,7,7,9,9,11,11,13,13,15,15-hexadecamethyl- $$ 1,1,3,3,5,5,7,7,9,9,11,11,13,13,15,15-Hexadecamethyloctasiloxane # $$

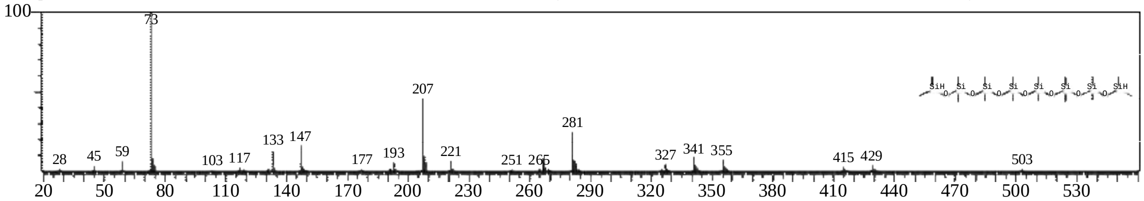

Hit#:2 Entry:157431 Library:NIST05.LIB
SI:69 Formula:C14H44O6Si7 CAS:19095-23-9 MolWeight:504 RetIndex:1526
CompName:Heptasiloxane, 1,1,3,3,5,5,7,7,9,9,11,11,13,13-tetradecamethyl- $$ 1,1,3,3,5,5,7,7,9,9,11,11,13,13-Tetradecamethylheptasiloxane # $$

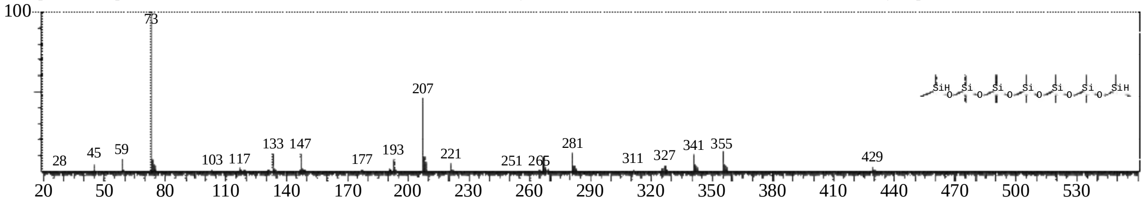

Hit#:3 Entry:150228 Library:NIST05.LIB
SI:66 Formula:C12H38O5Si6 CAS:995-82-4 MolWeight:430 RetIndex:1341
CompName:Hexasiloxane, 1,1,3,3,5,5,7,7,9,9,11,11-dodecamethyl- $$ 1,1,3,3,5,5,7,7,9,9,11,11-Dodecamethylhexasiloxane # $$

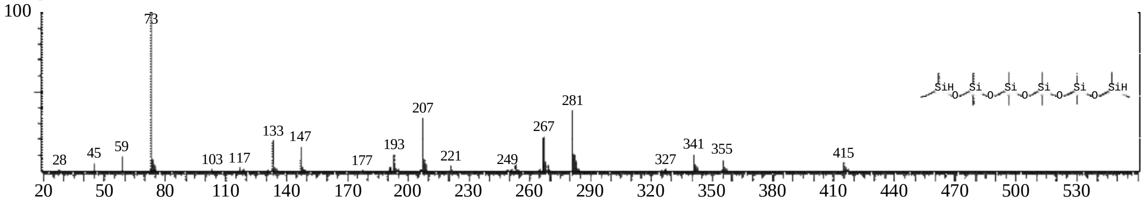

Hit#:4 Entry:158804 Library:NIST05.LIB
SI:62 Formula:C16H48O6Si7 CAS:541-01-5 MolWeight:532 RetIndex:1437
CompName:Heptasiloxane, hexadecamethyl- $$ Hexadecamethylheptasiloxane $$ 1,1,1,3,3,5,5,7,7,9,9,11,11,13,13,13-Hexadecamethylheptasiloxane # $$

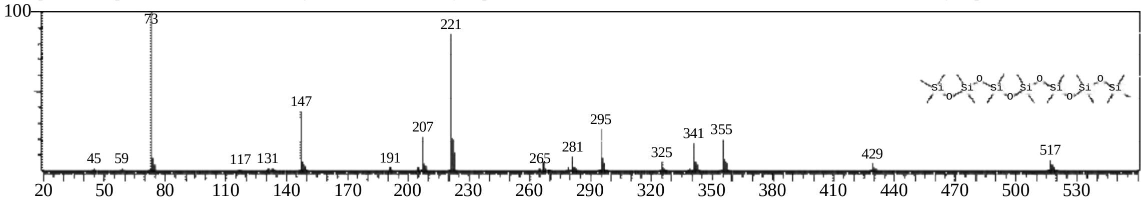

Hit#:5 Entry:128617 Library:NIST05.LIB
SI:62 Formula:C12H34O4Si4 CAS:72182-11-7 MolWeight:354 RetIndex:1094
CompName:3-Isopropoxy-1,1,1,5,5,5-hexamethyl-3-(trimethylsiloxy)trisiloxane $$ Isopropyl tris(trimethylsilyl) orthosilicate # $$

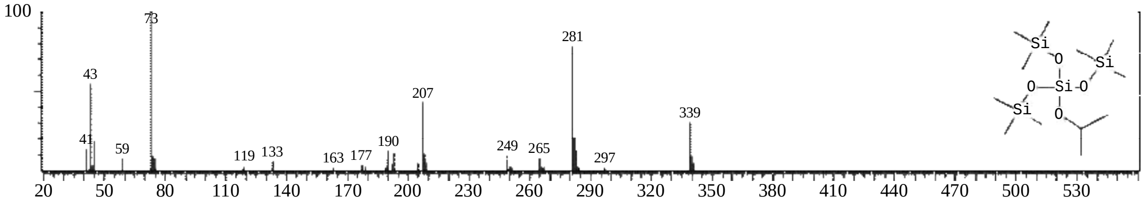

<< Target >>
Line#:68 R.Time:27.692(Scan#:2964) MassPeaks:175
RawMode:Averaged 27.683-27.700(2963-2965) BasePeak:207.05(11704)
BG Mode:Calc. from Peak Group 1 - Event 1

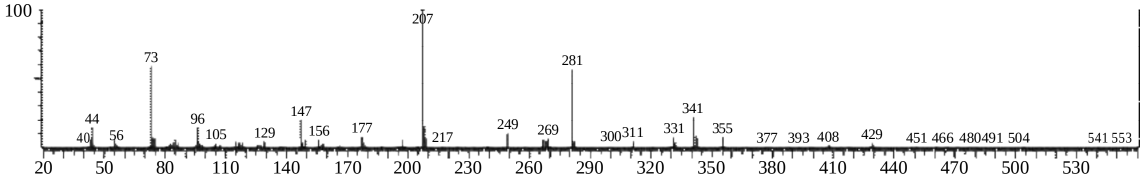

Hit#:1 Entry:160407 Library:NIST05.LIB
SI:63 Formula:C16H50O7Si8 CAS:19095-24-0 MolWeight:578 RetIndex:1710
CompName:Octasiloxane, 1,1,3,3,5,5,7,7,9,9,11,11,13,13,15,15-hexadecamethyl- $$ 1,1,3,3,5,5,7,7,9,9,11,11,13,13,15,15-Hexadecamethyloctasiloxane # $$

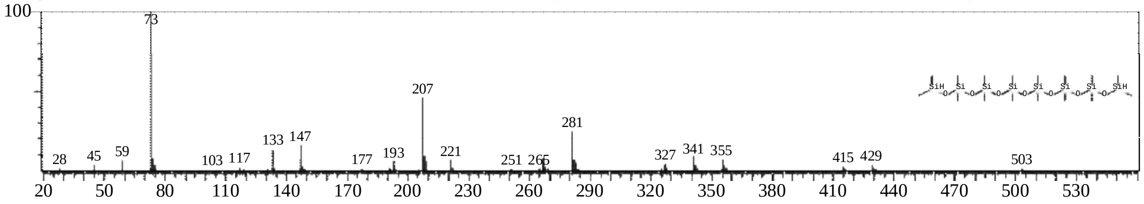

Hit#:2 Entry:157431 Library:NIST05.LIB
SI:62 Formula:C14H44O6Si7 CAS:19095-23-9 MolWeight:504 RetIndex:1526
CompName:Heptasiloxane, 1,1,3,3,5,5,7,7,9,9,11,11,13,13-tetradecamethyl- $$ 1,1,3,3,5,5,7,7,9,9,11,11,13,13-Tetradecamethylheptasiloxane # $$

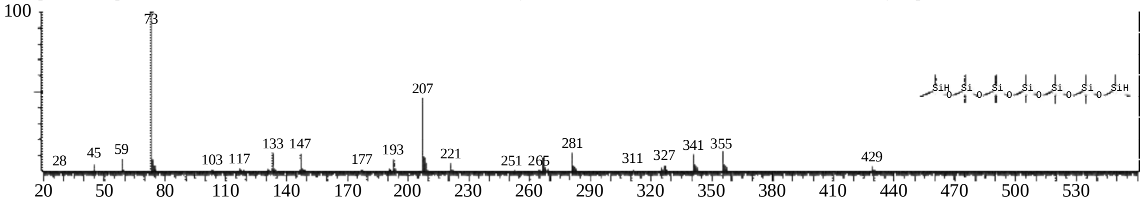

Hit#:3 Entry:150228 Library:NIST05.LIB
SI:62 Formula:C12H38O5Si6 CAS:995-82-4 MolWeight:430 RetIndex:1341
CompName:Hexasiloxane, 1,1,3,3,5,5,7,7,9,9,11,11-dodecamethyl- $$ 1,1,3,3,5,5,7,7,9,9,11,11-Dodecamethylhexasiloxane # $$

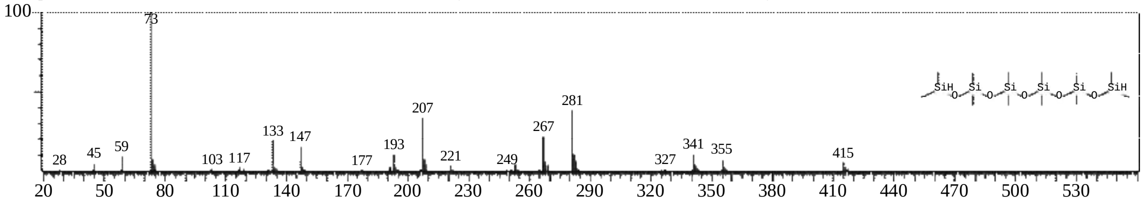

Hit#:4 Entry:129452 Library:NIST05.LIB
SI:59 Formula:C10H32O4Si5 CAS:995-83-5 MolWeight:356 RetIndex:1156
CompName:Pentasiloxane, 1,1,3,3,5,5,7,7,9,9-decamethyl- $$ 1,1,3,3,5,5,7,7,9,9-Decamethylpentasiloxane # $$

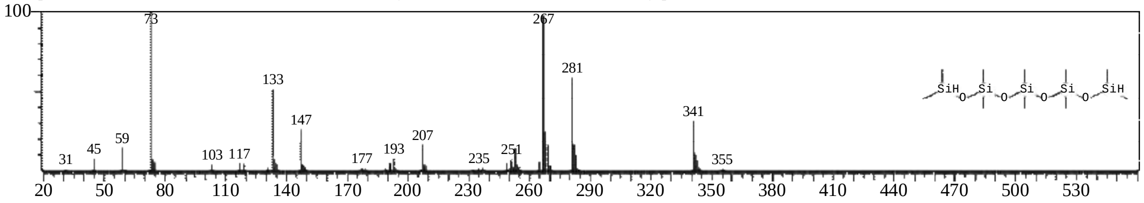

Hit#:5 Entry:106361 Library:NIST05.LIB
SI:58 Formula:C10H30O3Si4 CAS:141-62-8 MolWeight:310 RetIndex:883
CompName:Tetrasiloxane, decamethyl- $$ Decamethyltetrasiloxane $$ [(CH3)3SiOSi(CH3)2]2O $$ CD3780 $$ D3780 $$ 1,1,1,3,3,5,5,7,7,7-Decamethyltetras

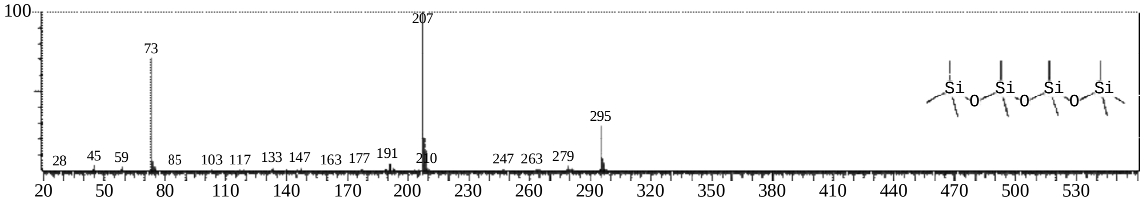

<< Target >>
Line#:69 R.Time:28.625(Scan#:3076) MassPeaks:211
RawMode:Averaged 28.617-28.633(3075-3077) BasePeak:207.15(12637)
BG Mode:Calc. from Peak Group 1 - Event 1

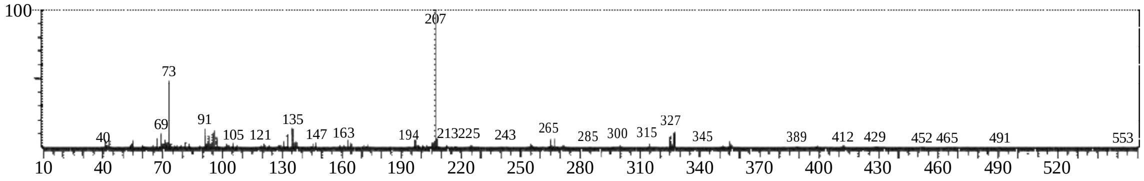

Hit#:1 Entry:54151 Library:NIST05.LIB
SI:63 Formula:C12H22Si2 CAS:13183-70-5 MolWeight:222 RetIndex:1124
CompName:Silane, 1,4-phenylenebis[trimethyl- $$ Silane, p-phenylenebis[trimethyl- $$ p-Bis(trimethylsilyl)benzene $$ Benzene, p-bis(trimethylsilyl)- $$ 1,4-

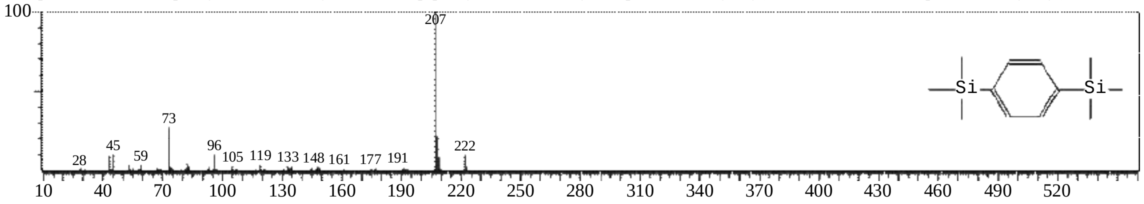

Hit#:2 Entry:54150 Library:NIST05.LIB
SI:63 Formula:C12H22Si2 CAS:17151-09-6 MolWeight:222 RetIndex:1124
CompName:1,2-Bis(trimethylsilyl)benzene $$ Trimethyl[2-(trimethylsilyl)phenyl]silane # $$

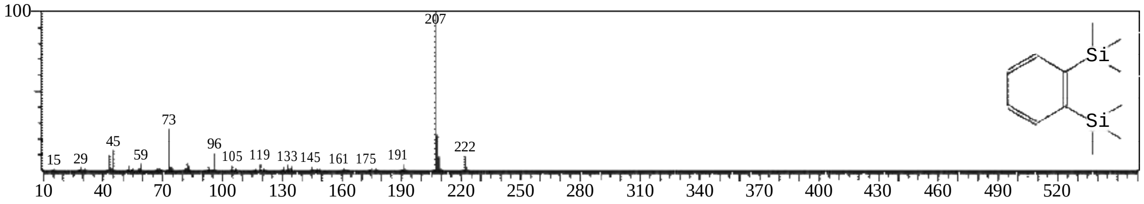

Hit#:3 Entry:71033 Library:NIST05.LIB
SI:63 Formula:C13H22OSi2 CAS:0-00-0 MolWeight:250 RetIndex:1354
CompName:2,4,6-Cycloheptatrien-1-one, 3,5-bis-trimethylsilyl-

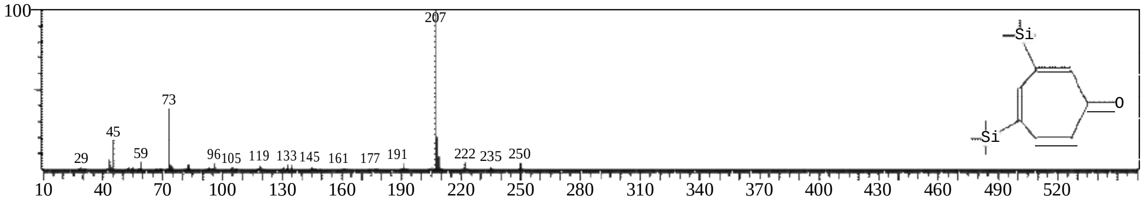

Hit#:4 Entry:54286 Library:NIST05.LIB
SI:62 Formula:C13H22OSi CAS:55012-80-1 MolWeight:222 RetIndex:1339
CompName:Silane, trimethyl[5-methyl-2-(1-methylethyl)phenoxy]- $$ Thymol-TMS $$ (2-Isopropyl-5-methylphenoxy)(trimethyl)silane # $$

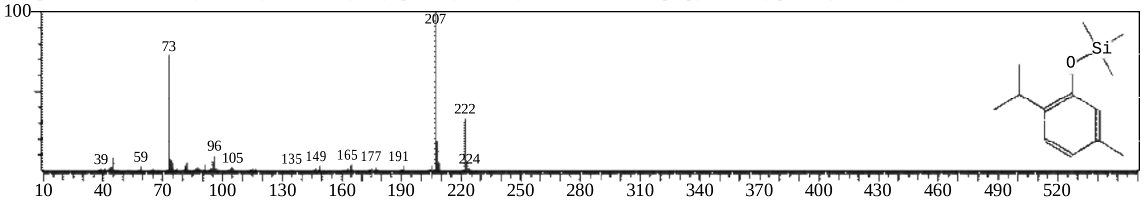

Hit#:5 Entry:100121 Library:NIST05.LIB
SI:62 Formula:C20H26O2 CAS:0-00-0 MolWeight:298 RetIndex:2092
CompName:Phenylacetic acid, 2-(1-adamantyl)ethyl ester

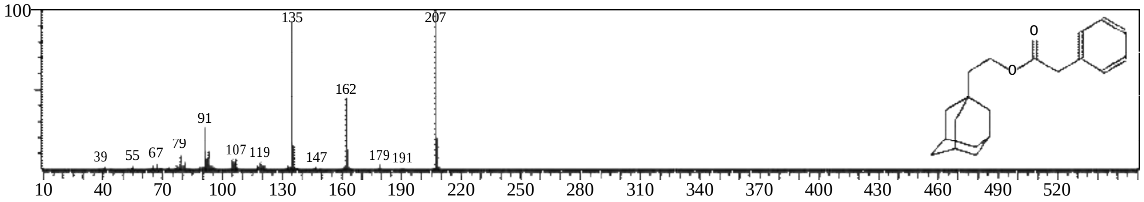

<< Target >>
Line#:70 R.Time:28.825(Scan#:3100) MassPeaks:344
RawMode:Averaged 28.817-28.833(3099-3101) BasePeak:57.10(198866)
BG Mode:Calc. from Peak Group 1 - Event 1

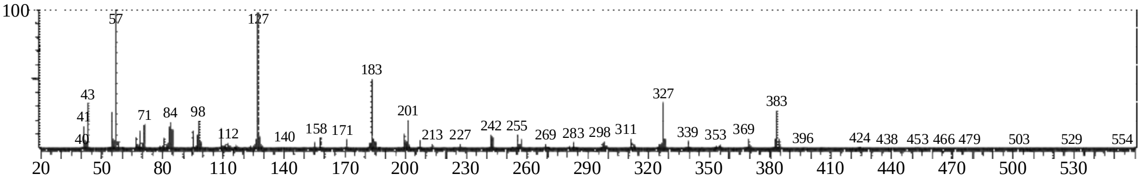

Hit#:1 Entry:154907 Library:NIST05.LIB
SI:72 Formula:C27H50O6 CAS:538-23-8 MolWeight:470 RetIndex:3143
CompName:Glycerol tricaprylate $$ Octanoin, tri- $$ Caprylic acid triglyceride $$ Caprylin $$ Glycerol trioctanoate $$ Glyceryl trioctanoate $$ Octanoic acid t

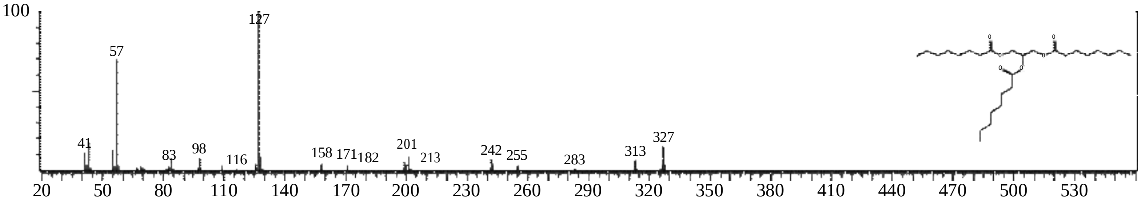

Hit#:2 Entry:57935 Library:NIST05.LIB
SI:67 Formula:C11H20N2O3 CAS:184637-49-8 MolWeight:228 RetIndex:1761
CompName:Azacyclohexane, 1-BOC-3-formamido- $$ tert-Butyl 3-(formylamino)-1-piperidinecarboxylate # $$

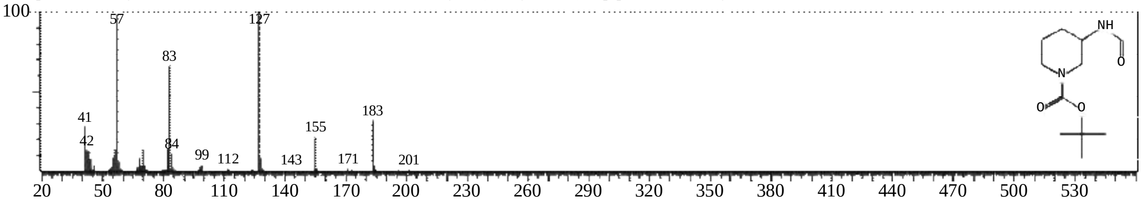

Hit#:3 Entry:115568 Library:NIST05.LIB
SI:67 Formula:C21H42O2 CAS:0-00-0 MolWeight:326 RetIndex:2212
CompName:Octanoic acid, 4-tridecyl ester

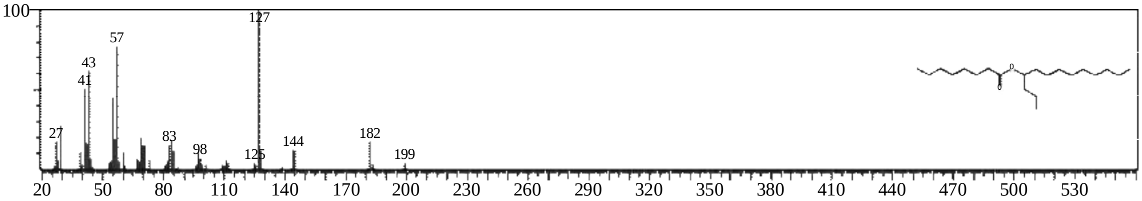

Hit#:4 Entry:115567 Library:NIST05.LIB
SI:66 Formula:C21H42O2 CAS:0-00-0 MolWeight:326 RetIndex:2212
CompName:Octanoic acid, 3-tridecyl ester

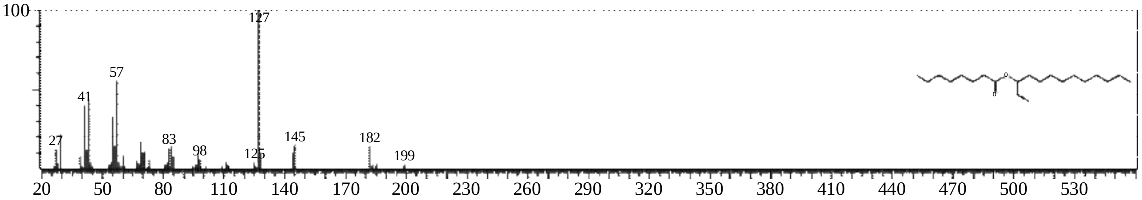

Hit#:5 Entry:129085 Library:NIST05.LIB
SI:65 Formula:C23H46O2 CAS:0-00-0 MolWeight:354 RetIndex:2411
CompName:Octanoic acid, 4-pentadecyl ester

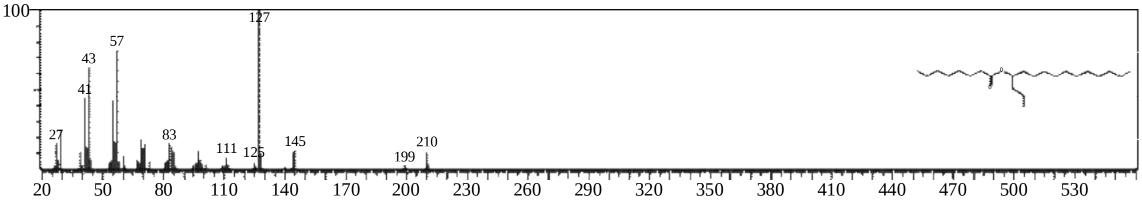

<< Target >>
Line#:71 R.Time:29.125(Scan#:3136) MassPeaks:333
RawMode:Averaged 29.117-29.133(3135-3137) BasePeak:57.10(42419)
BG Mode:Calc. from Peak Group 1 - Event 1

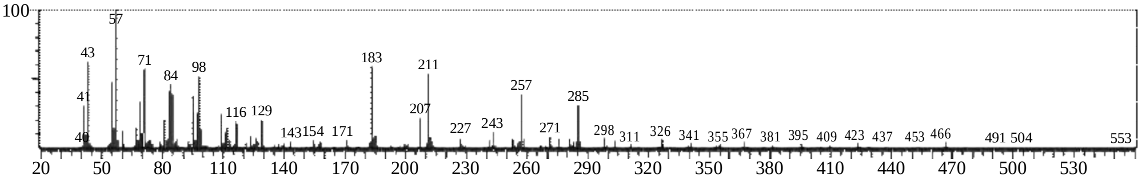

Hit#:1 Entry:157933 Library:NIST05.LIB
SI:78 Formula:C31H60O5 CAS:7770-09-4 MolWeight:512 RetIndex:3600
CompName:Tetradecanoic acid, 2-hydroxy-1,3-propanediyl ester $$ 2-Hydroxy-3-(tetradecanoyloxy)propyl myristate # $$

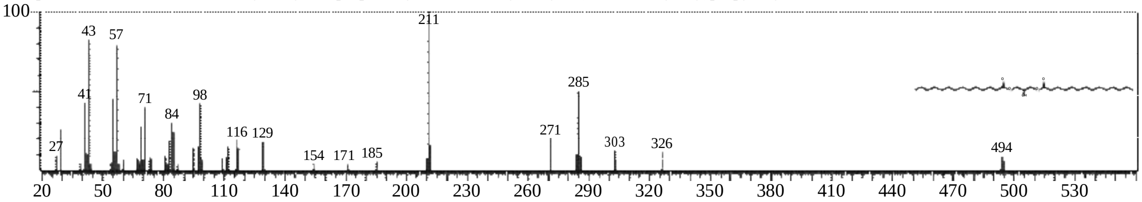

Hit#:2 Entry:162815 Library:NIST05.LIB
SI:76 Formula:C49H94O6 CAS:56846-96-9 MolWeight:778 RetIndex:5330
CompName:Octadecanoic acid, 2,3-bis[(1-oxotetradecyl)oxy]propyl ester $$ 2,3-Bis(tetradecanoyloxy)propyl stearate # $$

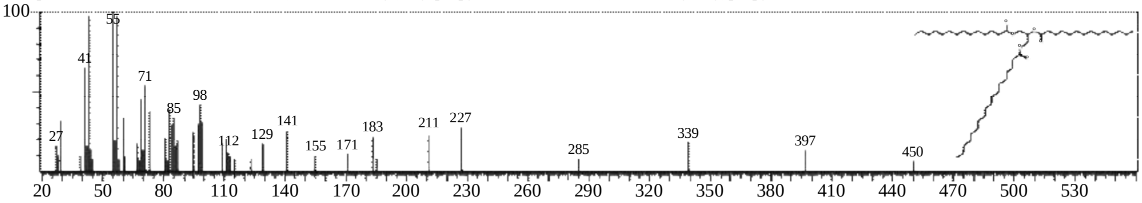

Hit#:3 Entry:153564 Library:NIST05.LIB
SI:76 Formula:C27H52O5 CAS:17598-94-6 MolWeight:456 RetIndex:3218
CompName:Dodecanoic acid, 1-(hydroxymethyl)-1,2-ethanediyl ester $$ 2-(Dodecanoyloxy)-1-(hydroxymethyl)ethyl laurate # $$

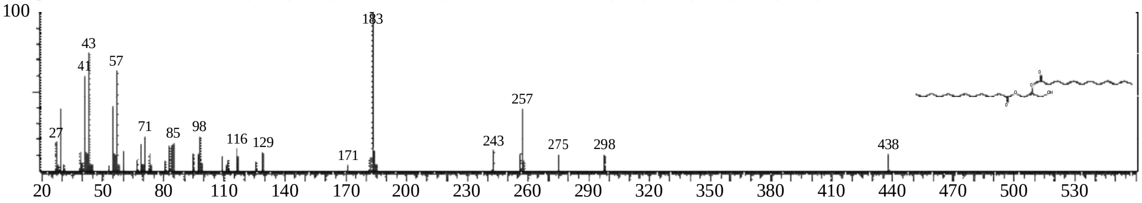

Hit#:4 Entry:27543 Library:NIST05s.LIB
SI:73 Formula:C39H74O6 CAS:538-24-9 MolWeight:638 RetIndex:4336
CompName:Dodecanoic acid, 1,2,3-propanetriyl ester $$ Laurin, tri- $$ Glycerol trilaurate $$ Glyceryl tridodecanoate $$ Glyceryl trilaurate $$ Lauric acid trigly

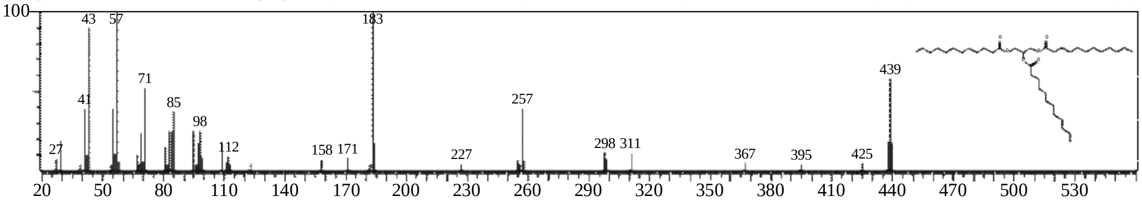

Hit#:5 Entry:162527 Library:NIST05.LIB
SI:72 Formula:C45H86O6 CAS:555-45-3 MolWeight:722 RetIndex:4932
CompName:Trimyristin $$ Tetradecanoic acid, 1,2,3-propanetriyl ester $$ Myristin, tri- $$ Glycerol trimyristate $$ Glyceryl trimyristate $$ Myristic acid triglyc

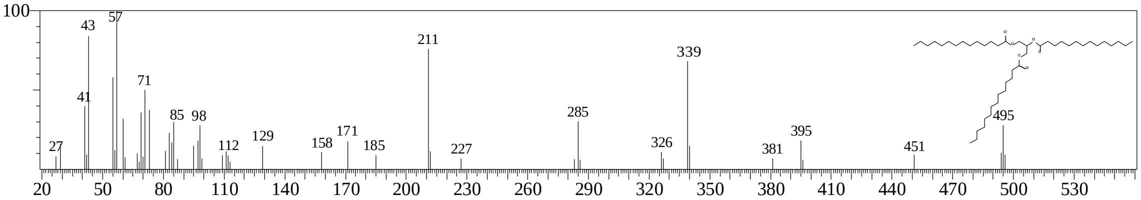

<< Target >>
Line#:72 R.Time:29.308(Scan#:3158) MassPeaks:239
RawMode:Averaged 29.300-29.317(3157-3159) BasePeak:57.10(5064)
BG Mode:Calc. from Peak Group 1 - Event 1

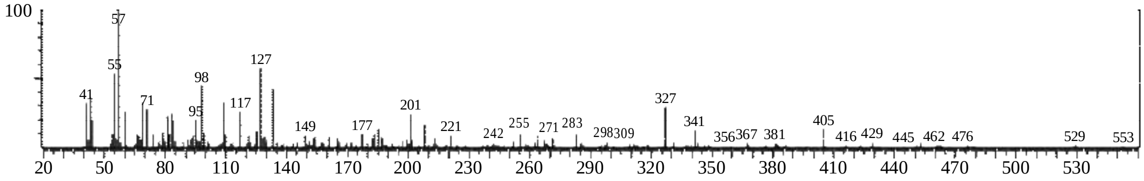

Hit#:1 Entry:32315 Library:NIST05.LIB
SI:59 Formula:C12H24O CAS:0-00-0 MolWeight:184 RetIndex:1316
CompName:3-t-Butyl-oct-6-en-1-ol

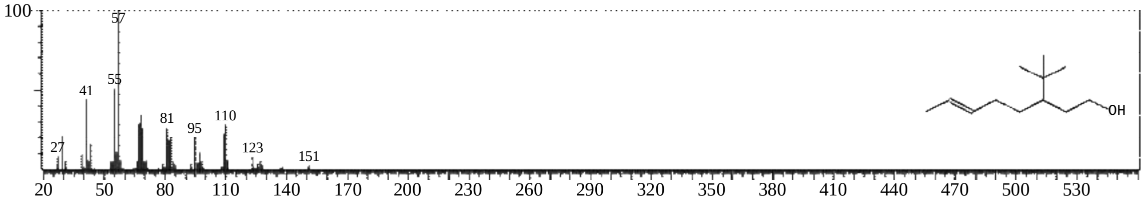

Hit#:2 Entry:14031 Library:NIST05s.LIB
SI:59 Formula:C11H20O2 CAS:4230-97-1 MolWeight:184 RetIndex:1272
CompName:Octanoic acid, 2-propenyl ester $$ Allyl caprylate $$ Allyl octanoate $$ Octanoic acid, allyl ester $$ Allyl n-octanoate $$

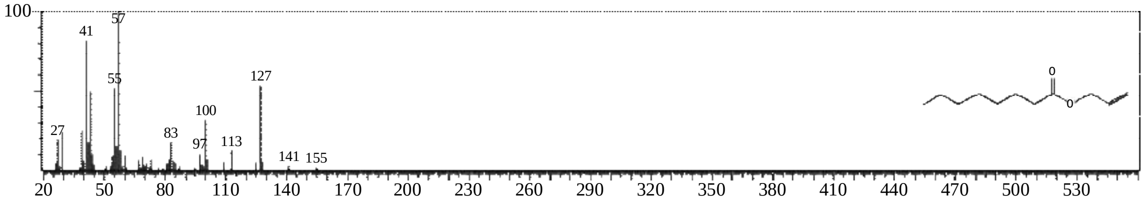

Hit#:3 Entry:122717 Library:NIST05.LIB
SI:58 Formula:C22H44O2 CAS:0-00-0 MolWeight:340 RetIndex:2311
CompName:Octanoic acid, 3-tetradecyl ester

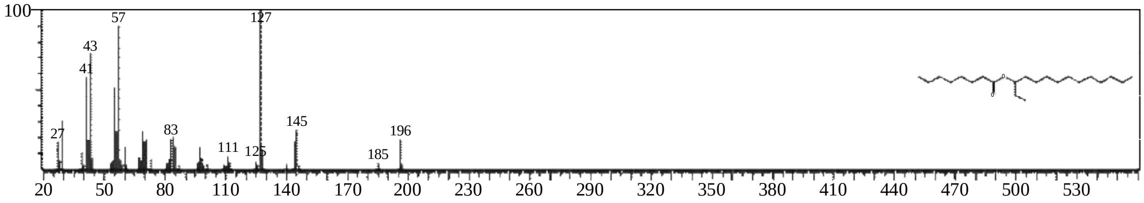

Hit#:4 Entry:109869 Library:NIST05.LIB
SI:58 Formula:C14H18BrFO2 CAS:2923-78-6 MolWeight:316 RetIndex:1951
CompName:Octanoic acid, 2-bromo-4-fluorophenyl ester $$ 2-Bromo-4-fluorophenyl octanoate # $$

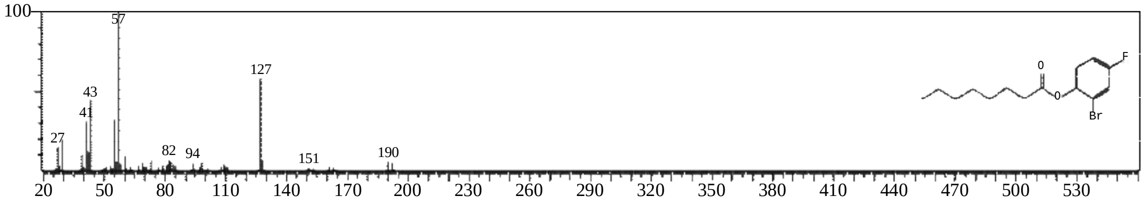

Hit#:5 Entry:49537 Library:NIST05.LIB
SI:58 Formula:C13H26O2 CAS:0-00-0 MolWeight:214 RetIndex:1521
CompName:Heptanoic acid, 3,5,5-triethyl-

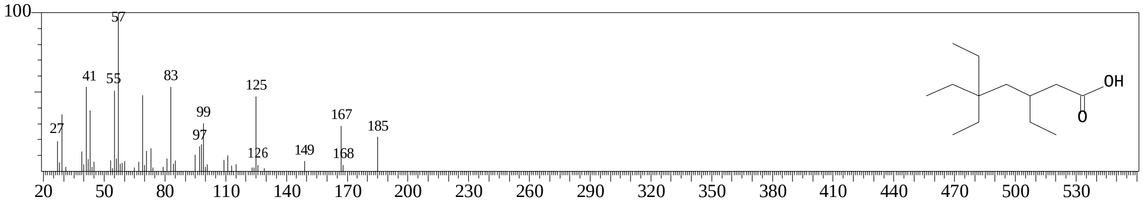

<< Target >>
Line#:73 R.Time:29.467(Scan#:3177) MassPeaks:365
RawMode:Averaged 29.458-29.475(3176-3178) BasePeak:57.10(84247)
BG Mode:Calc. from Peak Group 1 - Event 1

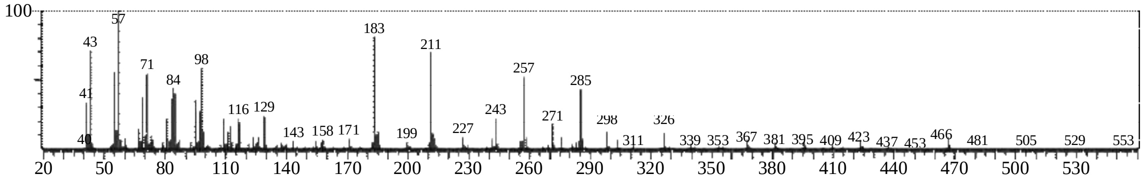

Hit#:1 Entry:157933 Library:NIST05.LIB
SI:79 Formula:C31H60O5 CAS:7770-09-4 MolWeight:512 RetIndex:3600
CompName:Tetradecanoic acid, 2-hydroxy-1,3-propanediyl ester $$ 2-Hydroxy-3-(tetradecanoyloxy)propyl myristate # $$

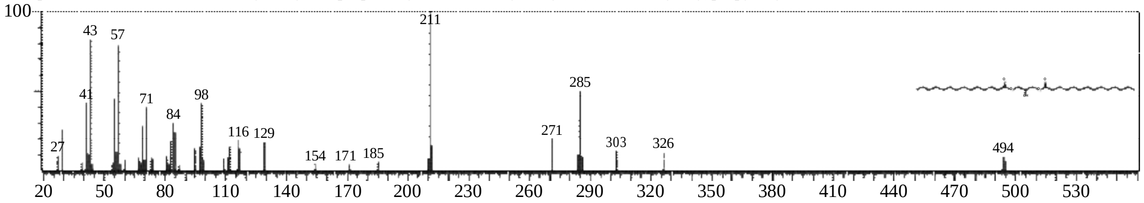

Hit#:2 Entry:153564 Library:NIST05.LIB
SI:76 Formula:C27H52O5 CAS:17598-94-6 MolWeight:456 RetIndex:3218
CompName:Dodecanoic acid, 1-(hydroxymethyl)-1,2-ethanediyl ester $$ 2-(Dodecanoyloxy)-1-(hydroxymethyl)ethyl laurate # $$

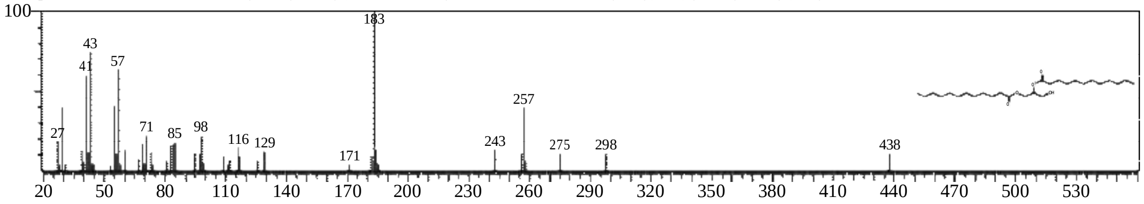

Hit#:3 Entry:162815 Library:NIST05.LIB
SI:73 Formula:C49H94O6 CAS:56846-96-9 MolWeight:778 RetIndex:5330
CompName:Octadecanoic acid, 2,3-bis[(1-oxotetradecyl)oxy]propyl ester $$ 2,3-Bis(tetradecanoyloxy)propyl stearate # $$

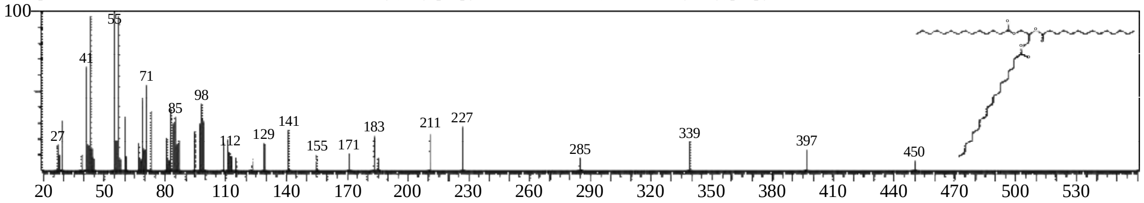

Hit#:4 Entry:27543 Library:NIST05s.LIB
SI:72 Formula:C39H74O6 CAS:538-24-9 MolWeight:638 RetIndex:4336
CompName:Dodecanoic acid, 1,2,3-propanetriyl ester $$ Laurin, tri- $$ Glycerol trilaurate $$ Glyceryl tridodecanoate $$ Glyceryl trilaurate $$ Lauric acid trigly

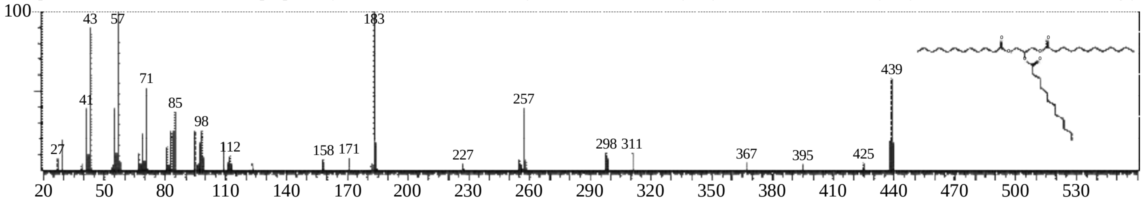

Hit#:5 Entry:162527 Library:NIST05.LIB
SI:71 Formula:C45H86O6 CAS:555-45-3 MolWeight:722 RetIndex:4932
CompName:Trimyristin $$ Tetradecanoic acid, 1,2,3-propanetriyl ester $$ Myristin, tri- $$ Glycerol trimyristate $$ Glyceryl trimyristate $$ Myristic acid triglyc

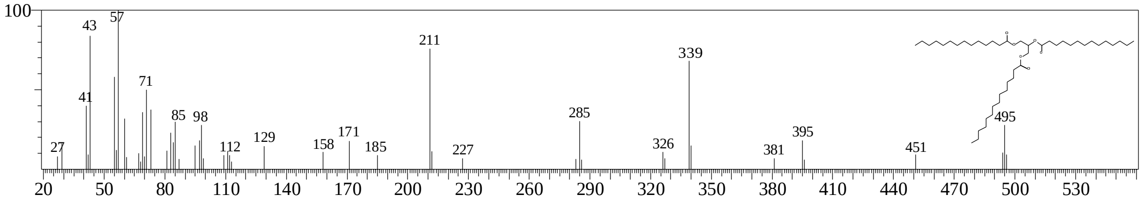

<< Target >>
Line#:74 R.Time:29.667(Scan#:3201) MassPeaks:215
RawMode:Averaged 29.658-29.675(3200-3202) BasePeak:207.05(8652)
BG Mode:Calc. from Peak Group 1 - Event 1

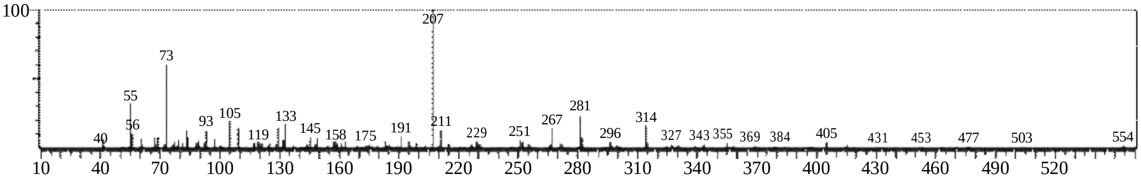

Hit#:1 Entry:157068 Library:NIST05.LIB
SI:51 Formula:C27H54O4Si2 CAS:54284-45-6 MolWeight:498 RetIndex:2796
CompName:1-Monolinoleoylglycerol trimethylsilyl ether $$ 9,12-Octadecadienoic acid (Z,Z)-, 2,3-bis[(trimethylsilyl)oxy]propyl ester $$ 2,3-Bis[(trimethylsily

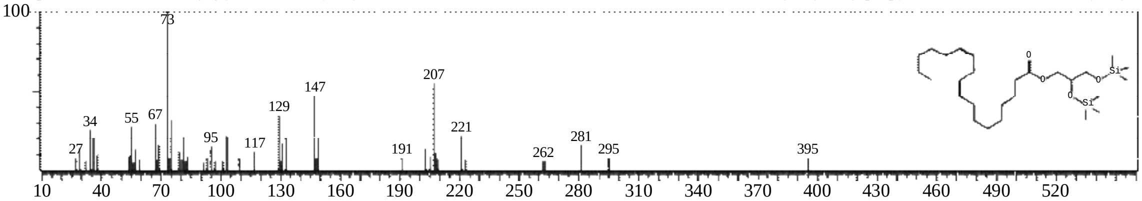

Hit#:2 Entry:18590 Library:NIST05s.LIB
SI:51 Formula:C12H22Si2 CAS:17151-09-6 MolWeight:222 RetIndex:1124
CompName:1,2-Bis(trimethylsilyl)benzene $$ Trimethyl[2-(trimethylsilyl)phenyl]silane # $$

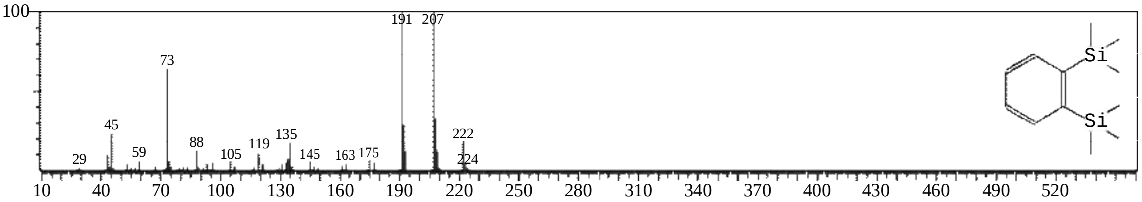

Hit#:3 Entry:71033 Library:NIST05.LIB
SI:51 Formula:C13H22OSi2 CAS:0-00-0 MolWeight:250 RetIndex:1354
CompName:2,4,6-Cycloheptatrien-1-one, 3,5-bis-trimethylsilyl-

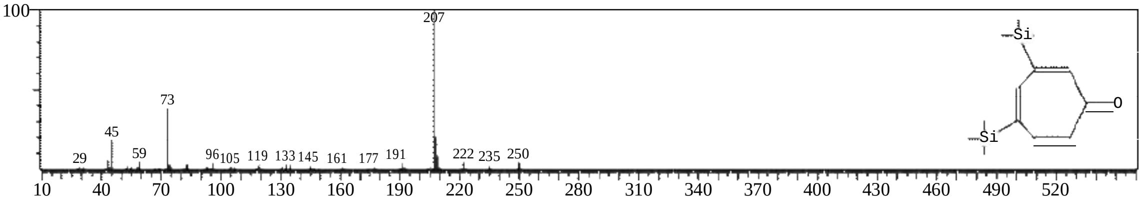

Hit#:4 Entry:54150 Library:NIST05.LIB
SI:50 Formula:C12H22Si2 CAS:17151-09-6 MolWeight:222 RetIndex:1124
CompName:1,2-Bis(trimethylsilyl)benzene $$ Trimethyl[2-(trimethylsilyl)phenyl]silane # $$

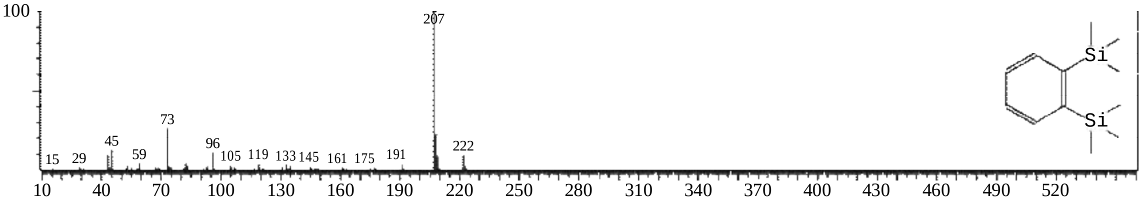

Hit#:5 Entry:54151 Library:NIST05.LIB
SI:50 Formula:C12H22Si2 CAS:13183-70-5 MolWeight:222 RetIndex:1124
CompName:Silane, 1,4-phenylenebis[trimethyl- $$ Silane, p-phenylenebis[trimethyl- $$ p-Bis(trimethylsilyl)benzene $$ Benzene, p-bis(trimethylsilyl)- $$ 1,4-

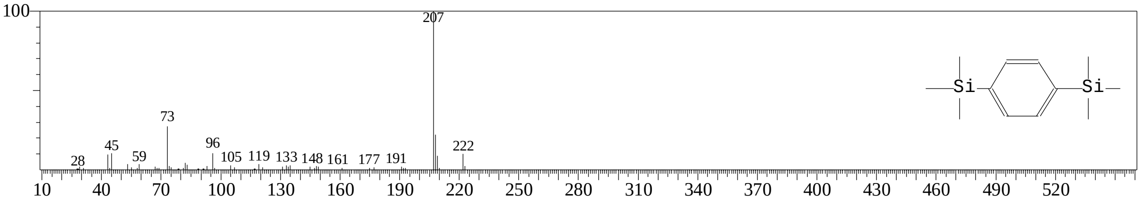

<< Target >>
Line#:75 R.Time:29.842(Scan#:3222) MassPeaks:198
RawMode:Averaged 29.833-29.850(3221-3223) BasePeak:207.00(16268)
BG Mode:Calc. from Peak Group 1 - Event 1

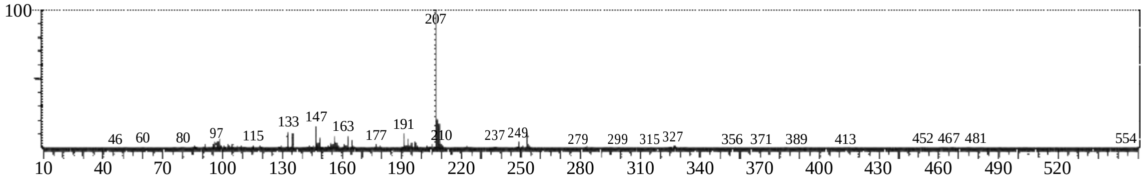

Hit#:1 Entry:53689 Library:NIST05.LIB
SI:74 Formula:C6H18O3Si3 CAS:541-05-9 MolWeight:222 RetIndex:620
CompName:Cyclotrisiloxane, hexamethyl- $$ Dimethylsiloxane cyclic trimer $$ Hexamethylcyclotrisiloxane $$ CH7260 $$ 2,2,4,4,6,6-Hexamethyl-1,3,5,2,4,6-

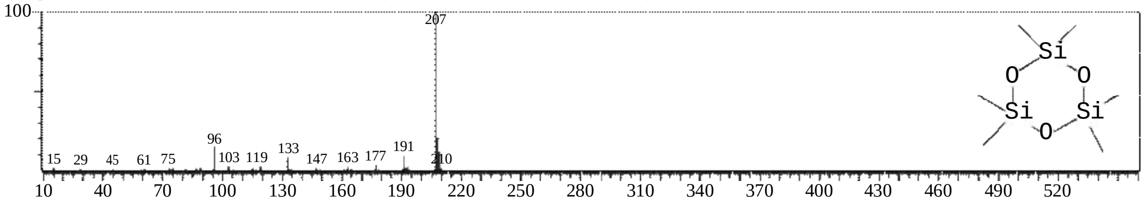

Hit#:2 Entry:18524 Library:NIST05s.LIB
SI:74 Formula:C6H18O3Si3 CAS:541-05-9 MolWeight:222 RetIndex:620
CompName:Cyclotrisiloxane, hexamethyl- $$ Dimethylsiloxane cyclic trimer $$ Hexamethylcyclotrisiloxane $$ CH7260 $$ 2,2,4,4,6,6-Hexamethyl-1,3,5,2,4,6-

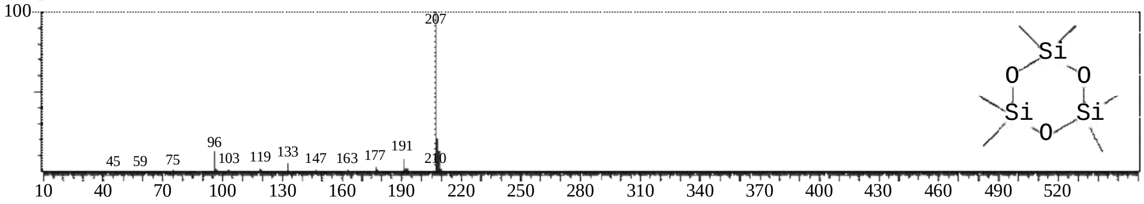

Hit#:3 Entry:71033 Library:NIST05.LIB
SI:71 Formula:C13H22OSi2 CAS:0-00-0 MolWeight:250 RetIndex:1354
CompName:2,4,6-Cycloheptatrien-1-one, 3,5-bis-trimethylsilyl-

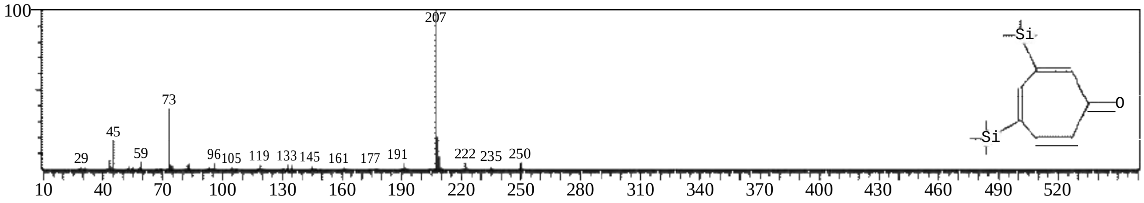

Hit#:4 Entry:79632 Library:NIST05.LIB
SI:70 Formula:C16H28OSi CAS:330455-64-6 MolWeight:264 RetIndex:1553
CompName:Benzene, 2-[(tert-butyldimethylsilyl)oxy]-1-isopropyl-4-methyl- $$ tert-Butyl(2-isopropyl-5-methylphenoxy)dimethylsilane # $$

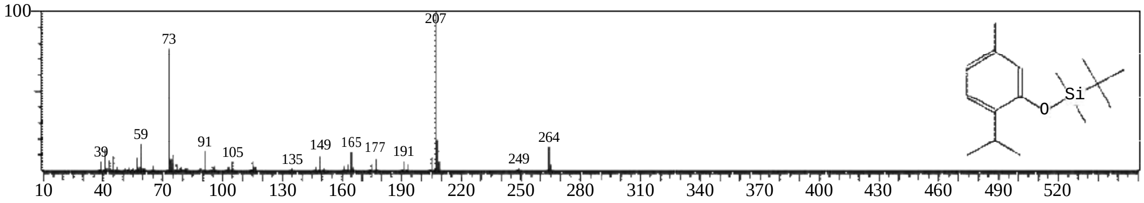

Hit#:5 Entry:54151 Library:NIST05.LIB
SI:69 Formula:C12H22Si2 CAS:13183-70-5 MolWeight:222 RetIndex:1124
CompName:Silane, 1,4-phenylenebis[trimethyl- $$ Silane, p-phenylenebis[trimethyl- $$ p-Bis(trimethylsilyl)benzene $$ Benzene, p-bis(trimethylsilyl)- $$ 1,4-

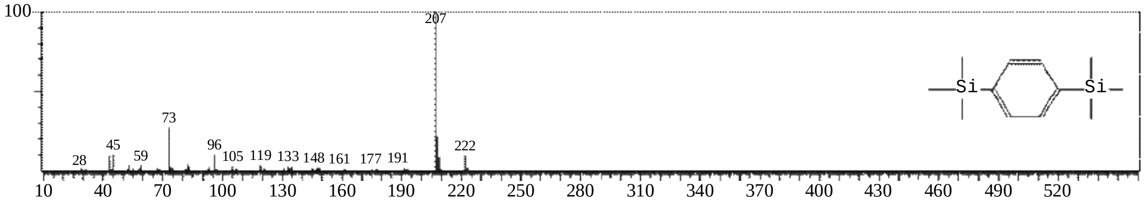

<< Target >>
Line#:76 R.Time:30.642(Scan#:3318) MassPeaks:235
RawMode:Averaged 30.633-30.650(3317-3319) BasePeak:207.00(8568)
BG Mode:Calc. from Peak Group 1 - Event 1

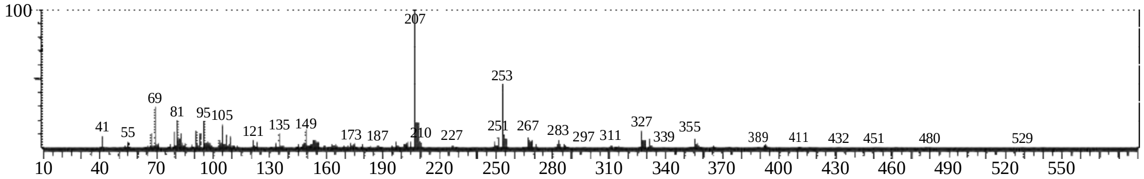

Hit#:1 Entry:130956 Library:NIST05.LIB
SI:60 Formula:C20H22ClNO3 CAS:25304-04-5 MolWeight:359 RetIndex:3105
CompName:2-[4-Cyclohexylbutanoylamino]-3-chloro-1,4-naphthoquinone $$ N-(3-Chloro-1,4-dioxo-1,4-dihydro-2-naphthalenyl)-4-cyclohexylbutanamide # $

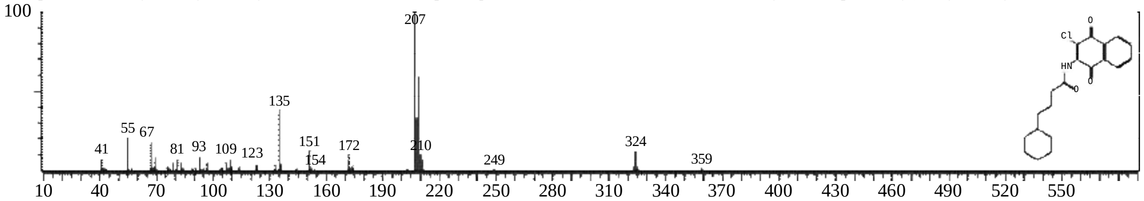

Hit#:2 Entry:54551 Library:NIST05.LIB
SI:57 Formula:C15H26O CAS:117591-80-7 MolWeight:222 RetIndex:1432
CompName:3,3,7,11-Tetramethyltricyclo[5.4.0.0(4,11)]undecan-1-ol

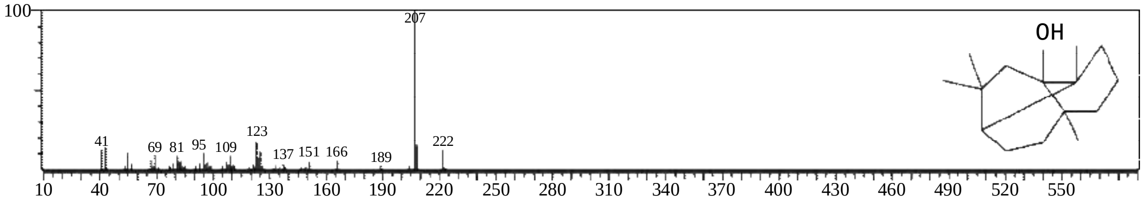

Hit#:3 Entry:100121 Library:NIST05.LIB
SI:53 Formula:C20H26O2 CAS:0-00-0 MolWeight:298 RetIndex:2092
CompName:Phenylacetic acid, 2-(1-adamantyl)ethyl ester

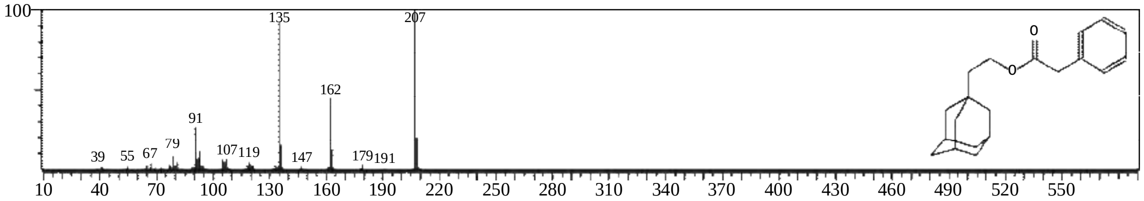

Hit#:4 Entry:143780 Library:NIST05.LIB
SI:53 Formula:C26H36O3 CAS:0-00-0 MolWeight:396 RetIndex:2956
CompName:3-Isopropyl-6a,10b-dimethyl-8-(2-oxo-2-phenyl-ethyl)-dodecahydro-benzo[f]chromen-7-one

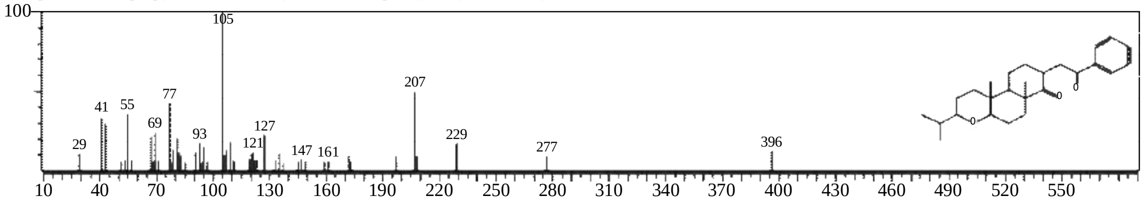

Hit#:5 Entry:92638 Library:NIST05.LIB
SI:52 Formula:C13H19BrO2 CAS:14575-01-0 MolWeight:286 RetIndex:1590
CompName:Methyl 3-bromo-1-adamantaneacetate $$ Tricyclo[3.3.1.1(3,7)-]decane-1-carboxylic acid, 3-bromo-5-methyl-, methyl ester $$ Methyl (3-bromo-1-a

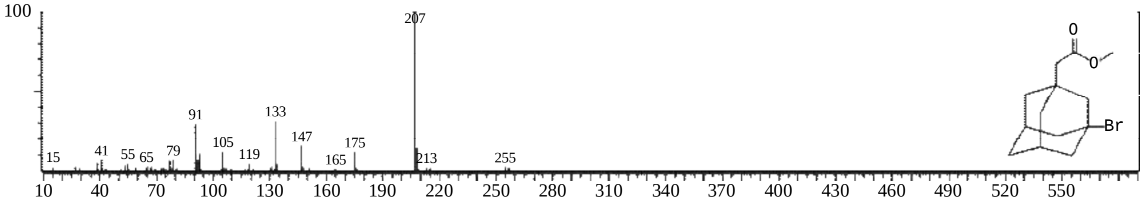

<< Target >>
Line#:77 R.Time:30.783(Scan#:3335) MassPeaks:185
RawMode:Averaged 30.775-30.792(3334-3336) BasePeak:206.95(15894)
BG Mode:Calc. from Peak Group 1 - Event 1

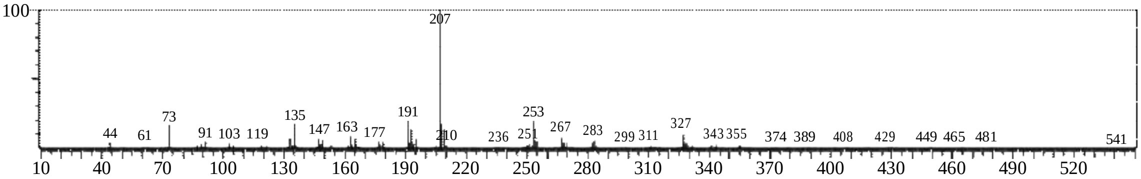

Hit#:1 Entry:53689 Library:NIST05.LIB
SI:69 Formula:C6H18O3Si3 CAS:541-05-9 MolWeight:222 RetIndex:620
CompName:Cyclotrisiloxane, hexamethyl- $$ Dimethylsiloxane cyclic trimer $$ Hexamethylcyclotrisiloxane $$ CH7260 $$ 2,2,4,4,6,6-Hexamethyl-1,3,5,2,4,6-

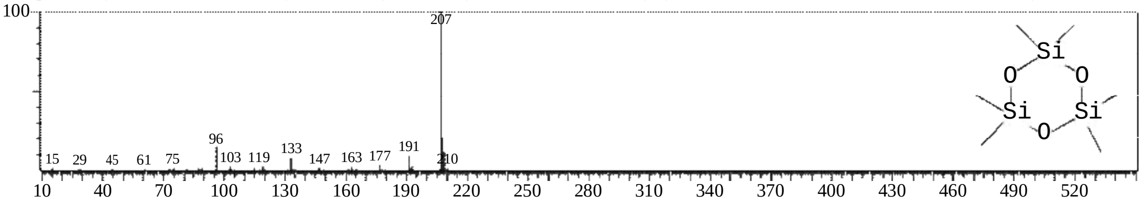

Hit#:2 Entry:18524 Library:NIST05s.LIB
SI:68 Formula:C6H18O3Si3 CAS:541-05-9 MolWeight:222 RetIndex:620
CompName:Cyclotrisiloxane, hexamethyl- $$ Dimethylsiloxane cyclic trimer $$ Hexamethylcyclotrisiloxane $$ CH7260 $$ 2,2,4,4,6,6-Hexamethyl-1,3,5,2,4,6-

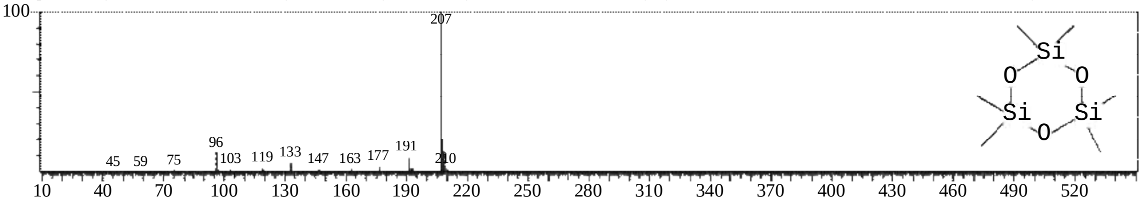

Hit#:3 Entry:98224 Library:NIST05.LIB
SI:66 Formula:C10H28O4Si3 CAS:3555-45-1 MolWeight:296 RetIndex:1049
CompName:Silicic acid, diethyl bis(trimethylsilyl) ester $$ 3,3-Diethoxy-1,1,1,5,5,5-hexamethyltrisiloxane $$ Diethyl bis(trimethylsilyl) orthosilicate # $$

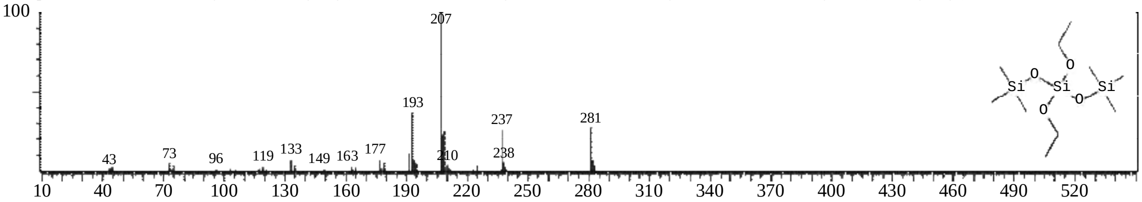

Hit#:4 Entry:113988 Library:NIST05.LIB
SI:64 Formula:C12H32O4Si3 CAS:18082-56-9 MolWeight:324 RetIndex:1119
CompName:3,3-Diisopropoxy-1,1,1,5,5,5-hexamethyltrisiloxane $$ Diisopropyl bis(trimethylsilyl) orthosilicate # $$

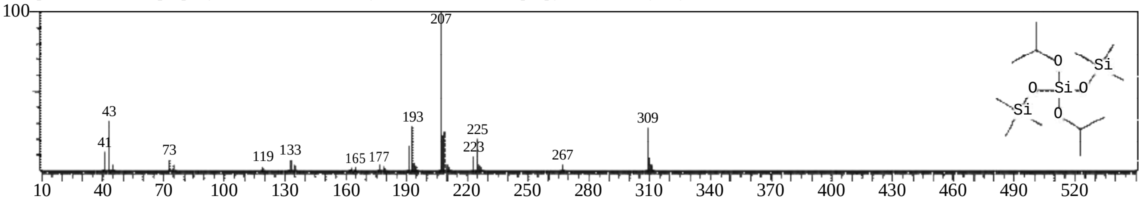

Hit#:5 Entry:147580 Library:NIST05.LIB
SI:63 Formula:C24H38O2Si2 CAS:70244-15-4 MolWeight:414 RetIndex:2431
CompName:Hexestrol di-TMS $$ [4-(1-Ethyl-2-(4-[(trimethylsilyl)oxy]phenyl)butyl)phenoxy](trimethyl)silane # $$

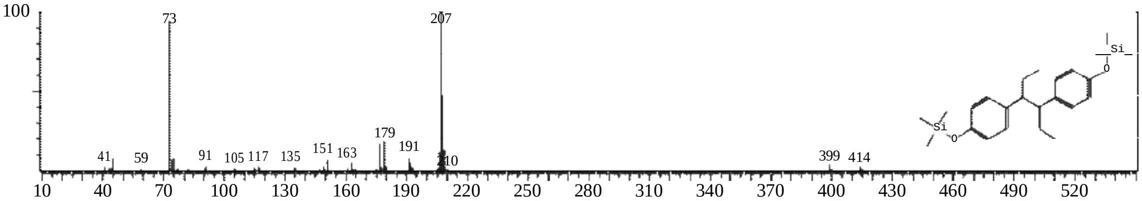

<< Target >>
Line#:78 R.Time:31.017(Scan#:3363) MassPeaks:203
RawMode:Averaged 31.008-31.025(3362-3364) BasePeak:207.05(16774)
BG Mode:Calc. from Peak Group 1 - Event 1

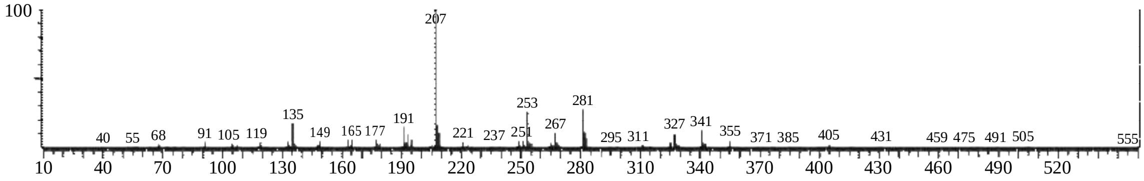

Hit#:1 Entry:98224 Library:NIST05.LIB
SI:67 Formula:C10H28O4Si3 CAS:3555-45-1 MolWeight:296 RetIndex:1049
CompName:Silicic acid, diethyl bis(trimethylsilyl) ester $$ 3,3-Diethoxy-1,1,1,5,5,5-hexamethyltrisiloxane $$ Diethyl bis(trimethylsilyl) orthosilicate # $$

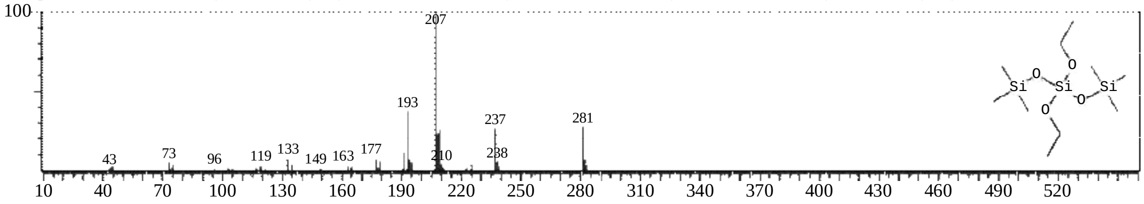

Hit#:2 Entry:18524 Library:NIST05s.LIB
SI:59 Formula:C6H18O3Si3 CAS:541-05-9 MolWeight:222 RetIndex:620
CompName:Cyclotrisiloxane, hexamethyl- $$ Dimethylsiloxane cyclic trimer $$ Hexamethylcyclotrisiloxane $$ CH7260 $$ 2,2,4,4,6,6-Hexamethyl-1,3,5,2,4,6-

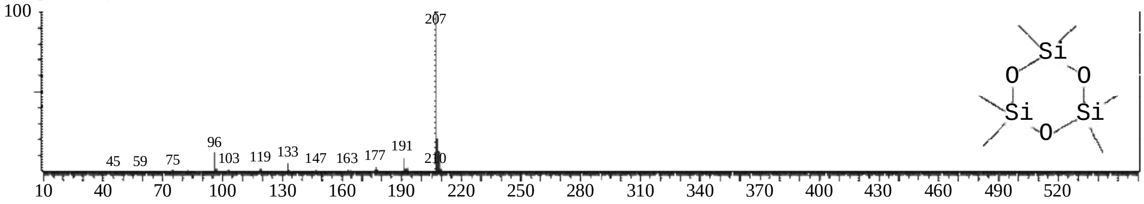

Hit#:3 Entry:53689 Library:NIST05.LIB
SI:59 Formula:C6H18O3Si3 CAS:541-05-9 MolWeight:222 RetIndex:620
CompName:Cyclotrisiloxane, hexamethyl- $$ Dimethylsiloxane cyclic trimer $$ Hexamethylcyclotrisiloxane $$ CH7260 $$ 2,2,4,4,6,6-Hexamethyl-1,3,5,2,4,6-

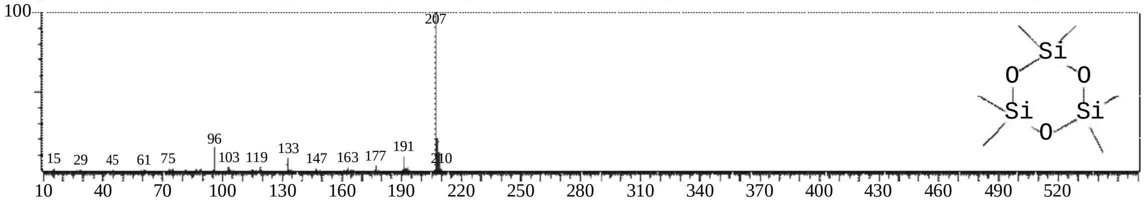

Hit#:4 Entry:113988 Library:NIST05.LIB
SI:58 Formula:C12H32O4Si3 CAS:18082-56-9 MolWeight:324 RetIndex:1119
CompName:3,3-Diisopropoxy-1,1,1,5,5,5-hexamethyltrisiloxane $$ Diisopropyl bis(trimethylsilyl) orthosilicate # $$

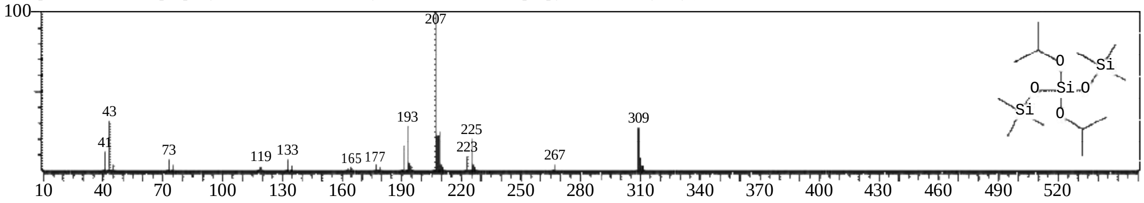

Hit#:5 Entry:135378 Library:NIST05.LIB
SI:57 Formula:C21H26N2O2S CAS:0-00-0 MolWeight:370 RetIndex:2979
CompName:2-[3-(4-tert-Butyl-phenoxy)-2-hydroxy-propylsulfanyl]-4,6-dimethyl-nicotinonitrile

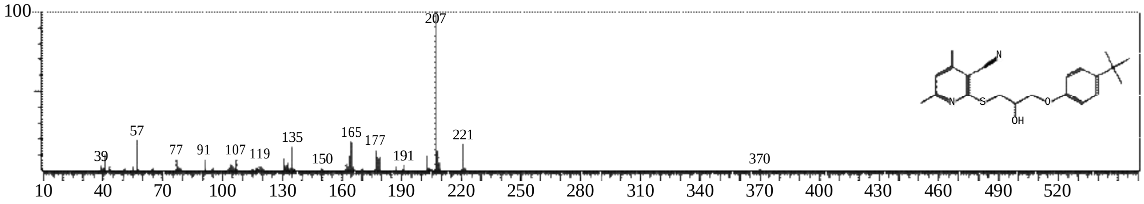

<< Target >>
Line#:79 R.Time:31.133(Scan#:3377) MassPeaks:171
RawMode:Averaged 31.125-31.142(3376-3378) BasePeak:207.10(12049)
BG Mode:Calc. from Peak Group 1 - Event 1

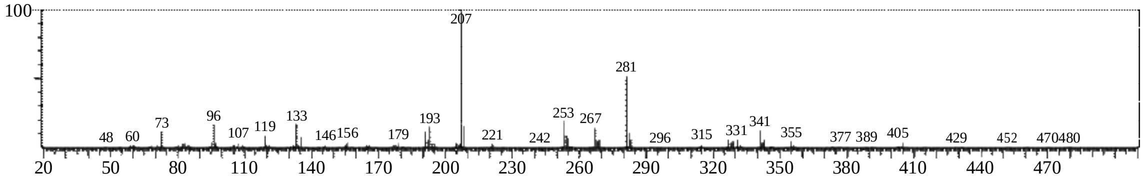

Hit#:1 Entry:150228 Library:NIST05.LIB
SI:65 Formula:C12H38O5Si6 CAS:995-82-4 MolWeight:430 RetIndex:1341
CompName:Hexasiloxane, 1,1,3,3,5,5,7,7,9,9,11,11-dodecamethyl- $$ 1,1,3,3,5,5,7,7,9,9,11,11-Dodecamethylhexasiloxane # $$

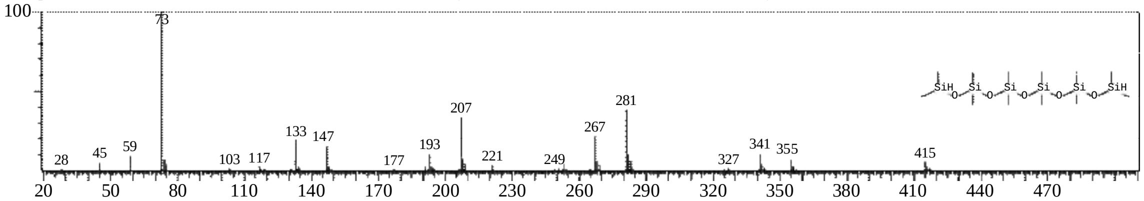

Hit#:2 Entry:129452 Library:NIST05.LIB
SI:61 Formula:C10H32O4Si5 CAS:995-83-5 MolWeight:356 RetIndex:1156
CompName:Pentasiloxane, 1,1,3,3,5,5,7,7,9,9-decamethyl- $$ 1,1,3,3,5,5,7,7,9,9-Decamethylpentasiloxane # $$

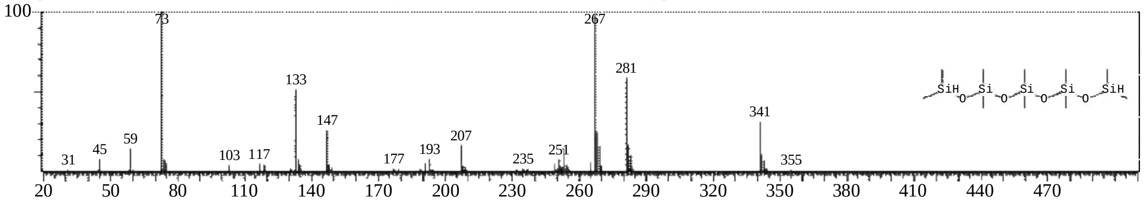

Hit#:3 Entry:98224 Library:NIST05.LIB
SI:61 Formula:C10H28O4Si3 CAS:3555-45-1 MolWeight:296 RetIndex:1049
CompName:Silicic acid, diethyl bis(trimethylsilyl) ester $$ 3,3-Diethoxy-1,1,1,5,5,5-hexamethyltrisiloxane $$ Diethyl bis(trimethylsilyl) orthosilicate # $$

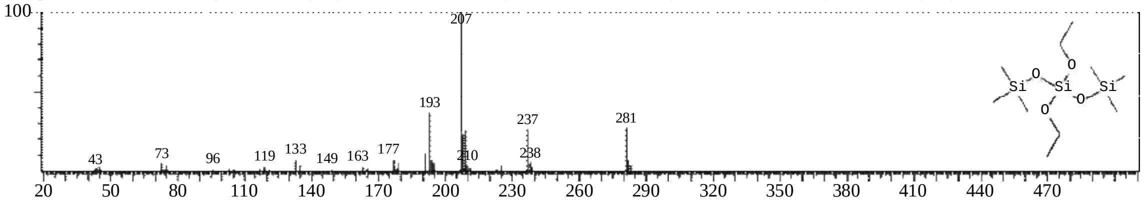

Hit#:4 Entry:157431 Library:NIST05.LIB
SI:61 Formula:C14H44O6Si7 CAS:19095-23-9 MolWeight:504 RetIndex:1526
CompName:Heptasiloxane, 1,1,3,3,5,5,7,7,9,9,11,11,13,13-tetradecamethyl- $$ 1,1,3,3,5,5,7,7,9,9,11,11,13,13-Tetradecamethylheptasiloxane # $$

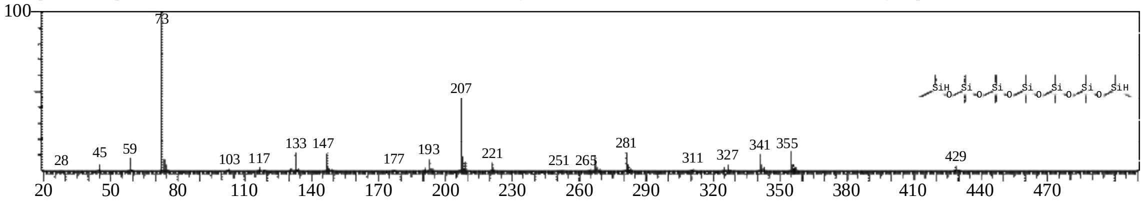

Hit#:5 Entry:160407 Library:NIST05.LIB
SI:60 Formula:C16H50O7Si8 CAS:19095-24-0 MolWeight:578 RetIndex:1710
CompName:Octasiloxane, 1,1,3,3,5,5,7,7,9,9,11,11,13,13,15,15-hexadecamethyl- $$ 1,1,3,3,5,5,7,7,9,9,11,11,13,13,15,15-Hexadecamethyloctasiloxane # $$

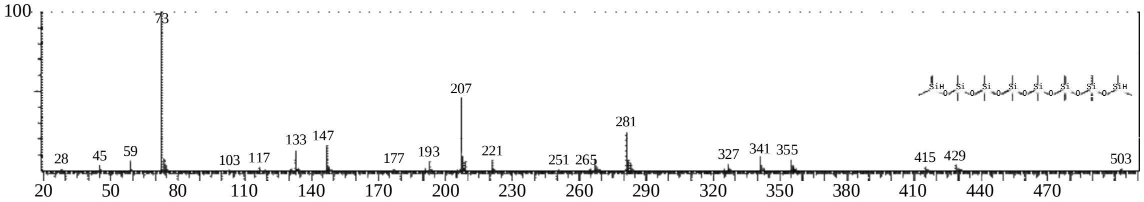

<< Target >>
Line#:80 R.Time:31.933(Scan#:3473) MassPeaks:357
RawMode:Averaged 31.925-31.942(3472-3474) BasePeak:57.10(187638)
BG Mode:Calc. from Peak Group 1 - Event 1

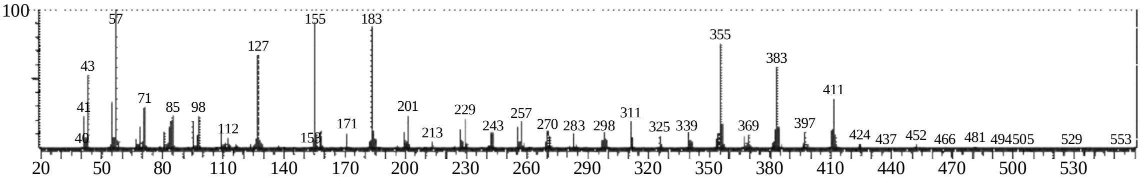

Hit#:1 Entry:159711 Library:NIST05.LIB
SI:61 Formula:C33H62O6 CAS:621-71-6 MolWeight:554 RetIndex:3739
CompName:Decanoic acid, 1,2,3-propanetriyl ester $$ Decanoin, tri- $$ Capric acid triglyceride $$ Caprin $$ Glycerol tricaprate $$ Glycerol tricaprin $$ Glycer

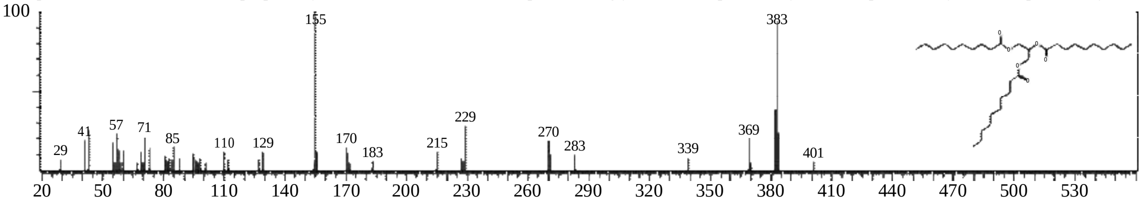

Hit#:2 Entry:27433 Library:NIST05s.LIB
SI:61 Formula:C33H62O6 CAS:621-71-6 MolWeight:554 RetIndex:3739
CompName:Decanoic acid, 1,2,3-propanetriyl ester $$ Decanoin, tri- $$ Capric acid triglyceride $$ Caprin $$ Glycerol tricaprate $$ Glycerol tricaprin $$ Glycer

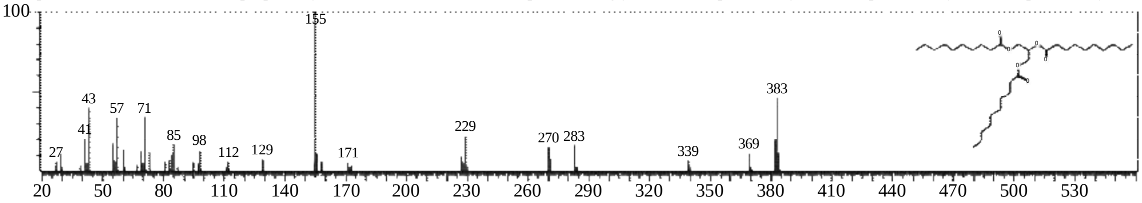

Hit#:3 Entry:27542 Library:NIST05s.LIB
SI:60 Formula:C39H74O6 CAS:538-24-9 MolWeight:638 RetIndex:4336
CompName:Dodecanoic acid, 1,2,3-propanetriyl ester $$ Laurin, tri- $$ Glycerol trilaurate $$ Glyceryl tridodecanoate $$ Glyceryl trilaurate $$ Lauric acid trigly

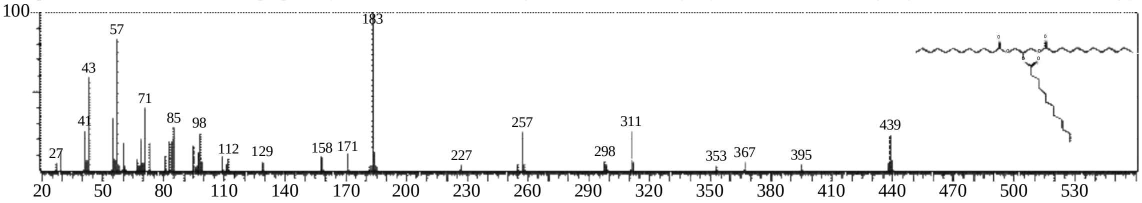

Hit#:4 Entry:160049 Library:NIST05.LIB
SI:59 Formula:C28H56NO8P CAS:3436-44-0 MolWeight:565 RetIndex:0
CompName:3,5,9-Trioxa-4-phosphanonadecan-1-aminium, 4-hydroxy-N,N,N-trimethyl-10-oxo-7-[(1-oxodecyl)oxy]-, hydroxide, inner salt, 4-oxide, (R)- $$ Ch

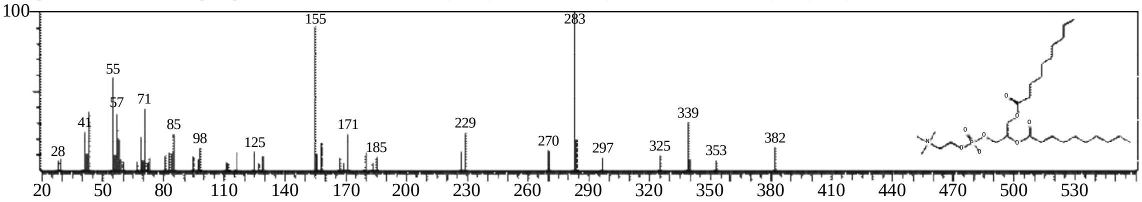

Hit#:5 Entry:27543 Library:NIST05s.LIB
SI:58 Formula:C39H74O6 CAS:538-24-9 MolWeight:638 RetIndex:4336
CompName:Dodecanoic acid, 1,2,3-propanetriyl ester $$ Laurin, tri- $$ Glycerol trilaurate $$ Glyceryl tridodecanoate $$ Glyceryl trilaurate $$ Lauric acid trigly

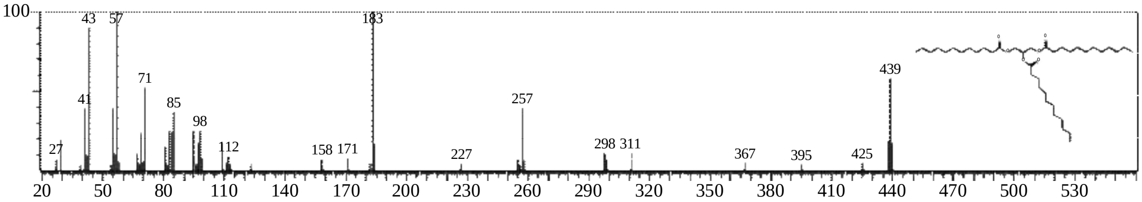

<< Target >>
Line#:81 R.Time:32.100(Scan#:3493) MassPeaks:374
RawMode:Averaged 32.092-32.108(3492-3494) BasePeak:183.20(239329)
BG Mode:Calc. from Peak Group 1 - Event 1

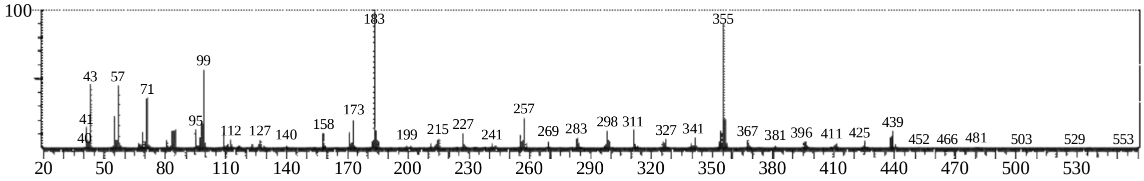

Hit#:1 Entry:27542 Library:NIST05s.LIB
SI:73 Formula:C39H74O6 CAS:538-24-9 MolWeight:638 RetIndex:4336
CompName:Dodecanoic acid, 1,2,3-propanetriyl ester $$ Laurin, tri- $$ Glycerol trilaurate $$ Glyceryl tridodecanoate $$ Glyceryl trilaurate $$ Lauric acid trigly

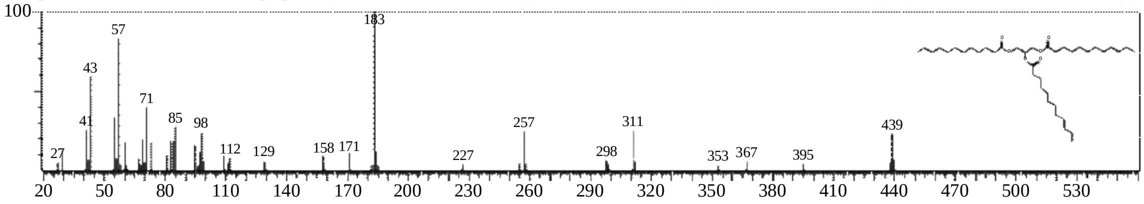

Hit#:2 Entry:161659 Library:NIST05.LIB
SI:72 Formula:C39H74O6 CAS:538-24-9 MolWeight:638 RetIndex:4336
CompName:Dodecanoic acid, 1,2,3-propanetriyl ester $$ Laurin, tri- $$ Glycerol trilaurate $$ Glyceryl tridodecanoate $$ Glyceryl trilaurate $$ Lauric acid trigly

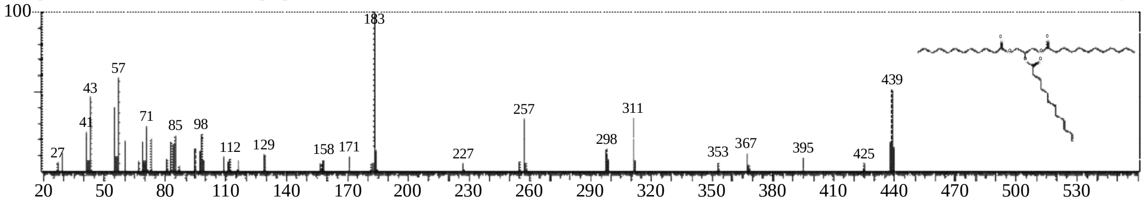

Hit#:3 Entry:27543 Library:NIST05s.LIB
SI:72 Formula:C39H74O6 CAS:538-24-9 MolWeight:638 RetIndex:4336
CompName:Dodecanoic acid, 1,2,3-propanetriyl ester $$ Laurin, tri- $$ Glycerol trilaurate $$ Glyceryl tridodecanoate $$ Glyceryl trilaurate $$ Lauric acid trigly

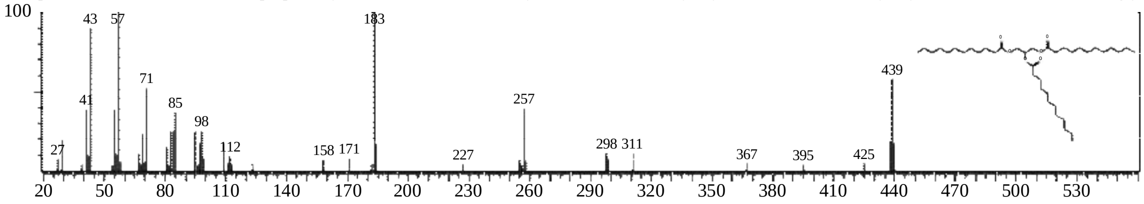

Hit#:4 Entry:153564 Library:NIST05.LIB
SI:66 Formula:C27H52O5 CAS:17598-94-6 MolWeight:456 RetIndex:3218
CompName:Dodecanoic acid, 1-(hydroxymethyl)-1,2-ethanediyl ester $$ 2-(Dodecanoyloxy)-1-(hydroxymethyl)ethyl laurate # $$

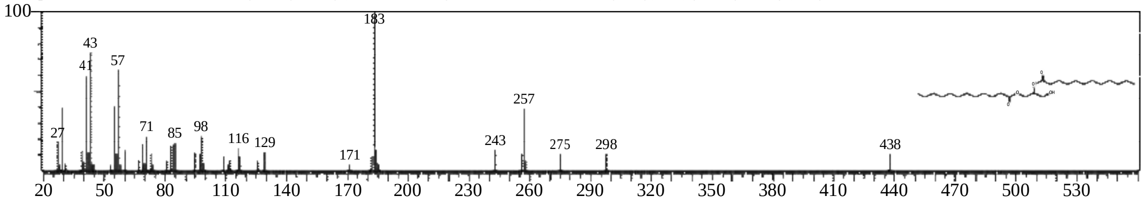

Hit#:5 Entry:161368 Library:NIST05.LIB
SI:65 Formula:C32H64NO8P CAS:18194-25-7 MolWeight:621 RetIndex:0
CompName:3,5,9-Trioxa-4-phosphaheneicosan-1-aminium, 4-hydroxy-N,N,N-trimethyl-10-oxo-7-[(1-oxododecyl)oxy]-, hydroxide, inner salt, 4-oxide, (R)- $$

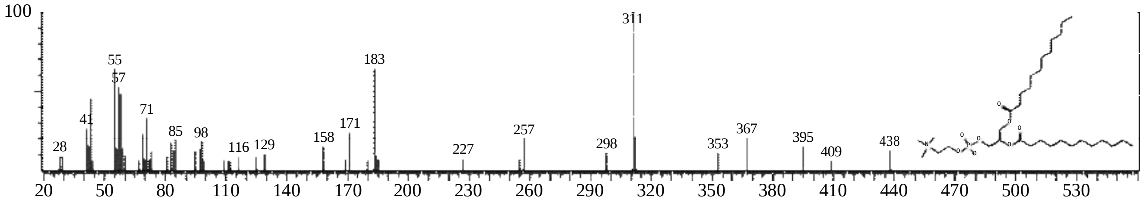

<< Target >>
Line#:82 R.Time:32.583(Scan#:3551) MassPeaks:293
RawMode:Averaged 32.575-32.592(3550-3552) BasePeak:43.10(10574)
BG Mode:Calc. from Peak Group 1 - Event 1

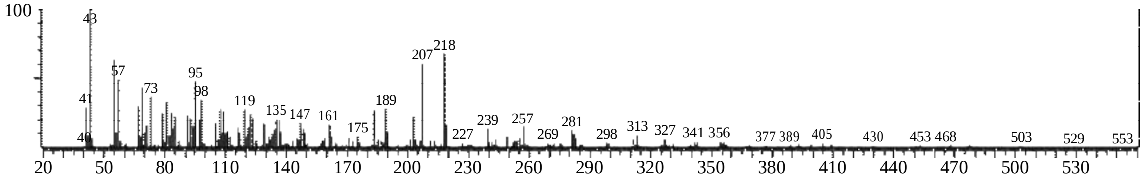

Hit#:1 Entry:149710 Library:NIST05.LIB
SI:70 Formula:C30H50O CAS:545-47-1 MolWeight:426 RetIndex:2848
CompName:Lupeol $$ Lup-20(29)-en-3-ol, (3.beta.)- $$ Lup-20(29)-en-3.beta.-ol $$ .beta.-Viscol $$ Clerodol $$ Fagarasterol $$ Fagarsterol $$ Lupenol $$ Mo

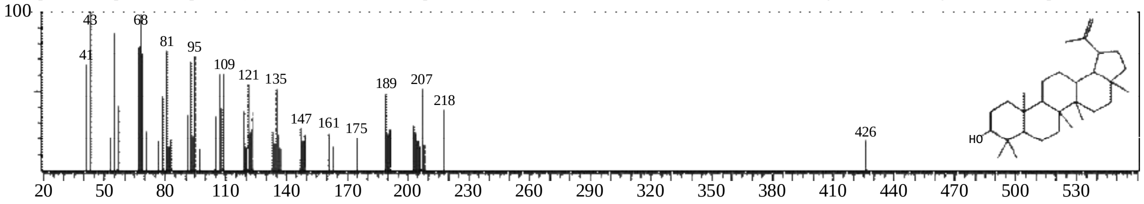

Hit#:2 Entry:146900 Library:NIST05.LIB
SI:69 Formula:C30H50 CAS:464-97-1 MolWeight:410 RetIndex:2685
CompName:Urs-12-ene

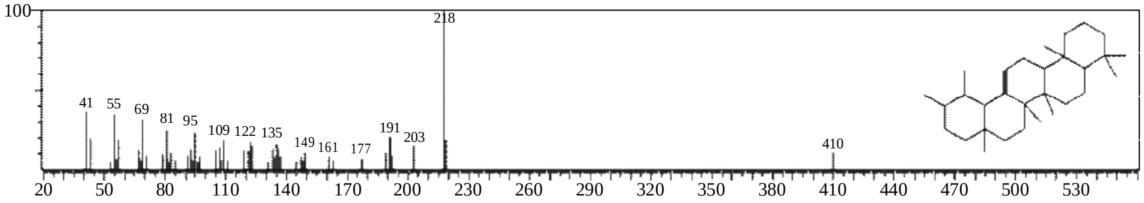

Hit#:3 Entry:159022 Library:NIST05.LIB
SI:69 Formula:C37H76O CAS:105794-58-9 MolWeight:536 RetIndex:3942
CompName:1-Heptatriacotanol $$ 1-Heptatriacontanol # $$

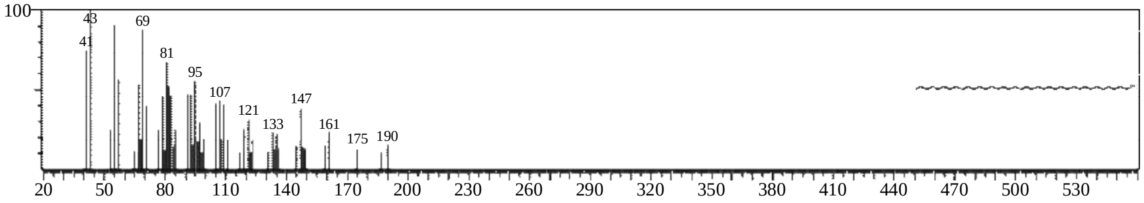

Hit#:4 Entry:54508 Library:NIST05.LIB
SI:69 Formula:C15H26O CAS:465-24-7 MolWeight:222 RetIndex:1593
CompName:1,4-Methanoazulen-9-ol, decahydro-1,5,5,8a-tetramethyl-, [1R-(1.alpha.,3a.beta.,4.alpha.,8a.beta.,9S*)]- $$ Longiborneol $$

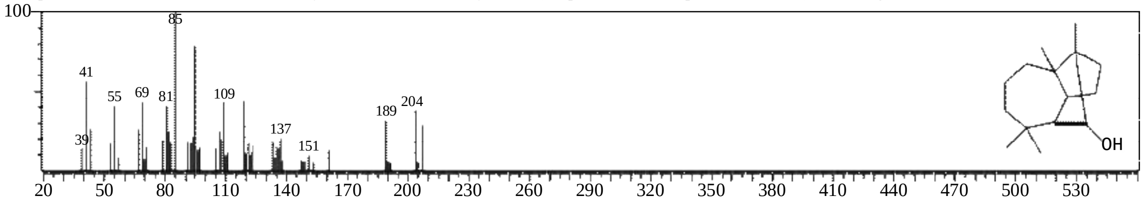

Hit#:5 Entry:54449 Library:NIST05.LIB
SI:69 Formula:C15H26O CAS:16981-75-2 MolWeight:222 RetIndex:1543
CompName:Illudol $$ 3,6,6,7b-Tetramethyldecahydro-1H-cyclobuta[e]inden-3-ol # $$

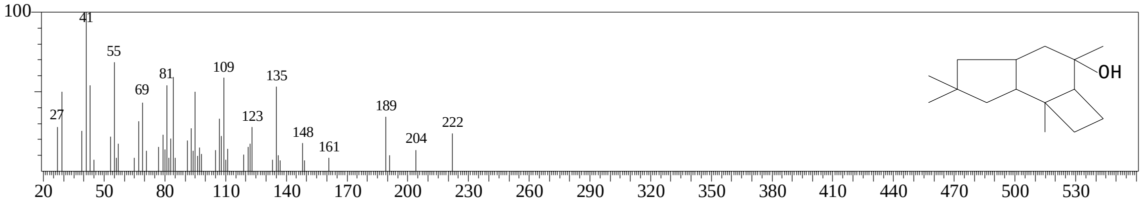

<< Target >>
Line#:83 R.Time:32.733(Scan#:3569) MassPeaks:161
RawMode:Averaged 32.725-32.742(3568-3570) BasePeak:207.05(6333)
BG Mode:Calc. from Peak Group 1 - Event 1

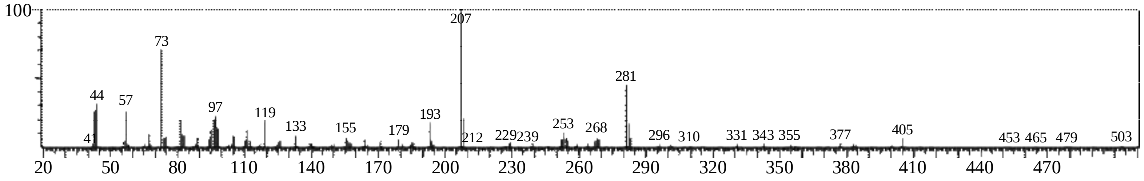

Hit#:1 Entry:128617 Library:NIST05.LIB
SI:54 Formula:C12H34O4Si4 CAS:72182-11-7 MolWeight:354 RetIndex:1094
CompName:3-Isopropoxy-1,1,1,5,5,5-hexamethyl-3-(trimethylsiloxy)trisiloxane $$ Isopropyl tris(trimethylsilyl) orthosilicate # $$

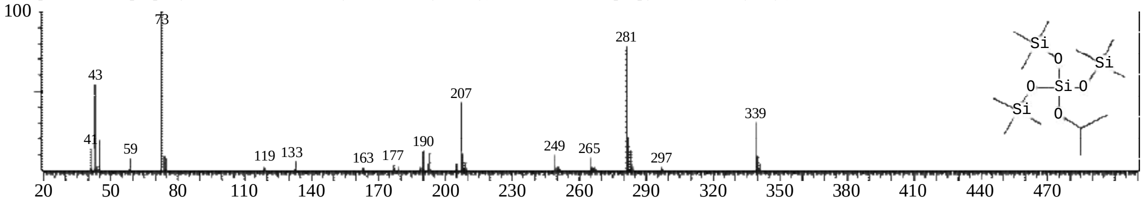

Hit#:2 Entry:54286 Library:NIST05.LIB
SI:54 Formula:C13H22OSi CAS:55012-80-1 MolWeight:222 RetIndex:1339
CompName:Silane, trimethyl[5-methyl-2-(1-methylethyl)phenoxy]- $$ Thymol-TMS $$ (2-Isopropyl-5-methylphenoxy)(trimethyl)silane # $$

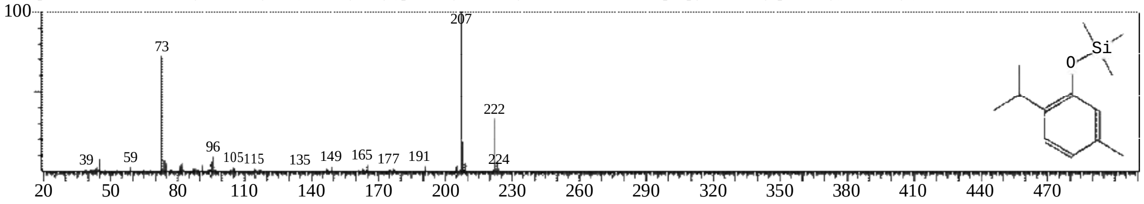

Hit#:3 Entry:54151 Library:NIST05.LIB
SI:53 Formula:C12H22Si2 CAS:13183-70-5 MolWeight:222 RetIndex:1124
CompName:Silane, 1,4-phenylenebis[trimethyl- $$ Silane, p-phenylenebis[trimethyl- $$ p-Bis(trimethylsilyl)benzene $$ Benzene, p-bis(trimethylsilyl)- $$ 1,4-

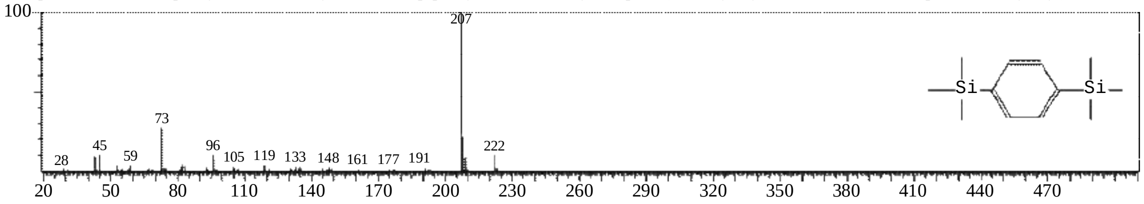

Hit#:4 Entry:98224 Library:NIST05.LIB
SI:53 Formula:C10H28O4Si3 CAS:3555-45-1 MolWeight:296 RetIndex:1049
CompName:Silicic acid, diethyl bis(trimethylsilyl) ester $$ 3,3-Diethoxy-1,1,1,5,5,5-hexamethyltrisiloxane $$ Diethyl bis(trimethylsilyl) orthosilicate # $$

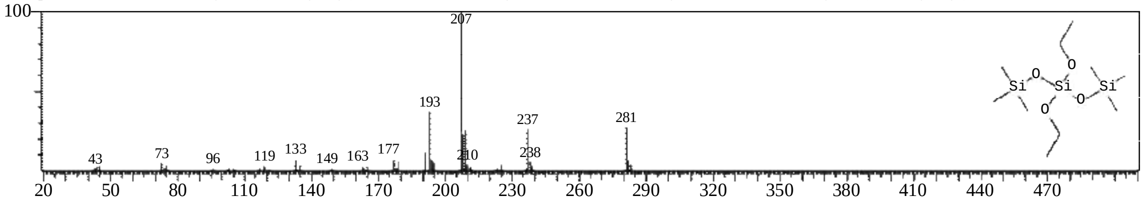

Hit#:5 Entry:134303 Library:NIST05.LIB
SI:52 Formula:C13H36O4Si4 CAS:87867-97-8 MolWeight:368 RetIndex:1257
CompName:3-Butoxy-1,1,1,5,5,5-hexamethyl-3-(trimethylsiloxy)trisiloxane $$ Butyl tris(trimethylsilyl) orthosilicate # $$

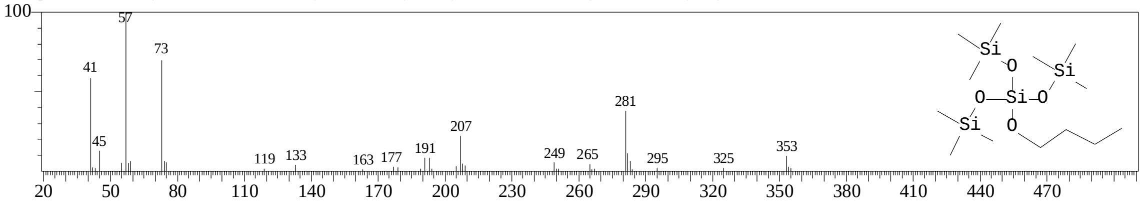

<< Target >>
Line#:84 R.Time:33.033(Scan#:3605) MassPeaks:300
RawMode:Averaged 33.025-33.042(3604-3606) BasePeak:57.10(18967)
BG Mode:Calc. from Peak Group 1 - Event 1

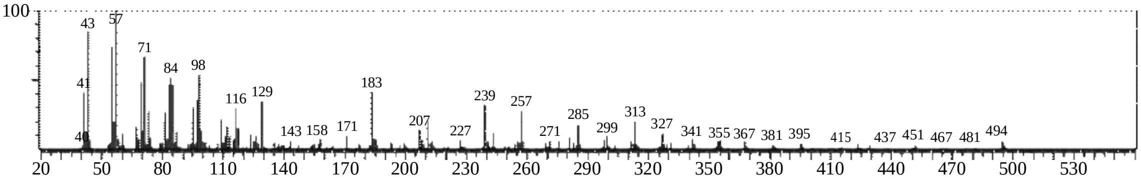

Hit#:1 Entry:27447 Library:NIST05s.LIB
SI:81 Formula:C35H68O5 CAS:761-35-3 MolWeight:568 RetIndex:4013
CompName:Hexadecanoic acid, 1-(hydroxymethyl)-1,2-ethanediyl ester $$ Palmitin, 1,2-di- $$ Dipalmitin $$ Glycerol 1,2-dipalmitate $$ 1,2-Dipalmitin $$ 1,2

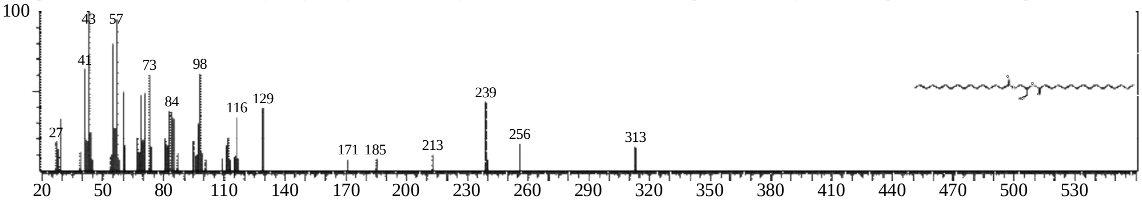

Hit#:2 Entry:160166 Library:NIST05.LIB
SI:79 Formula:C35H68O5 CAS:502-52-3 MolWeight:568 RetIndex:3997
CompName:Hexadecanoic acid, 2-hydroxy-1,3-propanediyl ester $$ Palmitin, 1,3-di- $$ Glycerol 1,3-dipalmitate $$ Palmitic acid diglycerin ester $$ 1,3-Dipalm

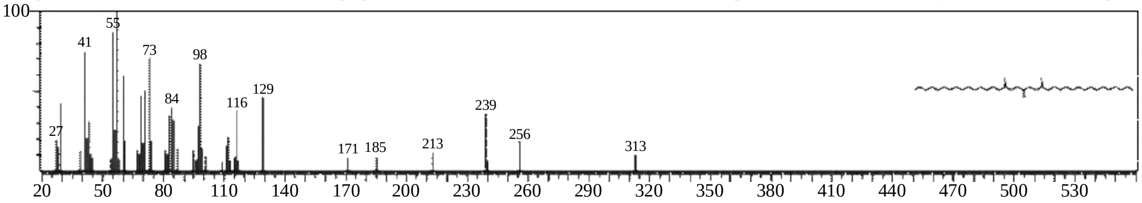

Hit#:3 Entry:162815 Library:NIST05.LIB
SI:79 Formula:C49H94O6 CAS:56846-96-9 MolWeight:778 RetIndex:5330
CompName:Octadecanoic acid, 2,3-bis[(1-oxotetradecyl)oxy]propyl ester $$ 2,3-Bis(tetradecanoyloxy)propyl stearate # $$

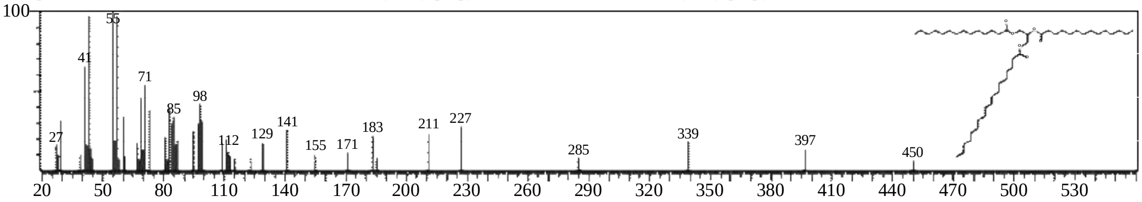

Hit#:4 Entry:27448 Library:NIST05s.LIB
SI:78 Formula:C35H68O5 CAS:502-52-3 MolWeight:568 RetIndex:3997
CompName:Hexadecanoic acid, 2-hydroxy-1,3-propanediyl ester $$ Palmitin, 1,3-di- $$ Glycerol 1,3-dipalmitate $$ Palmitic acid diglycerin ester $$ 1,3-Dipalm

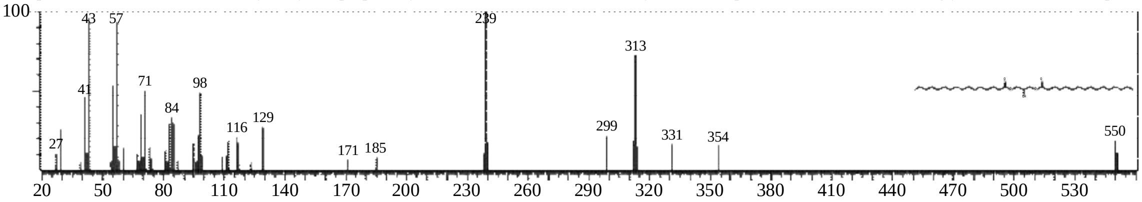

Hit#:5 Entry:162295 Library:NIST05.LIB
SI:78 Formula:C37H74NO8P CAS:3026-45-7 MolWeight:691 RetIndex:0
CompName:Hexadecanoic acid, 1-[[[(2-aminoethoxy)hydroxyphosphinyl]oxy]methyl]-1,2-ethanediyl ester $$ Palmitin, 1,2-di-, 2-aminoethyl hydrogen phospha

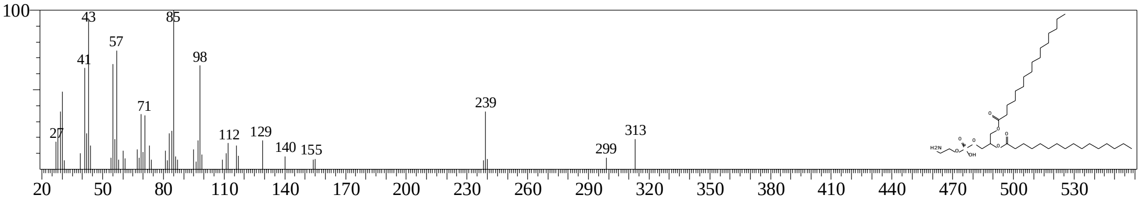

<< Target >>
Line#:85 R.Time:33.375(Scan#:3646) MassPeaks:183
RawMode:Averaged 33.367-33.383(3645-3647) BasePeak:207.15(11345)
BG Mode:Calc. from Peak Group 1 - Event 1

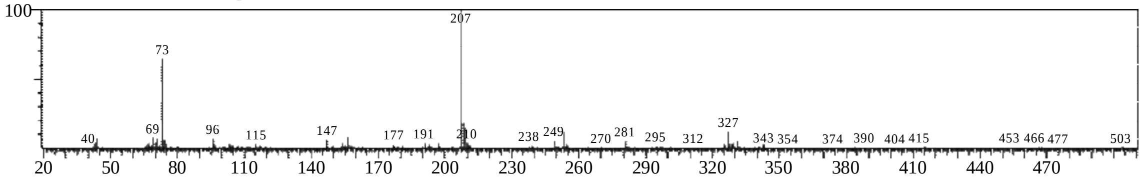

Hit#:1 Entry:106361 Library:NIST05.LIB
SI:67 Formula:C10H30O3Si4 CAS:141-62-8 MolWeight:310 RetIndex:883
CompName:Tetrasiloxane, decamethyl- $$ Decamethyltetrasiloxane $$ [(CH3)3SiOSi(CH3)2]2O $$ CD3780 $$ D3780 $$ 1,1,1,3,3,5,5,7,7,7-Decamethyltetras

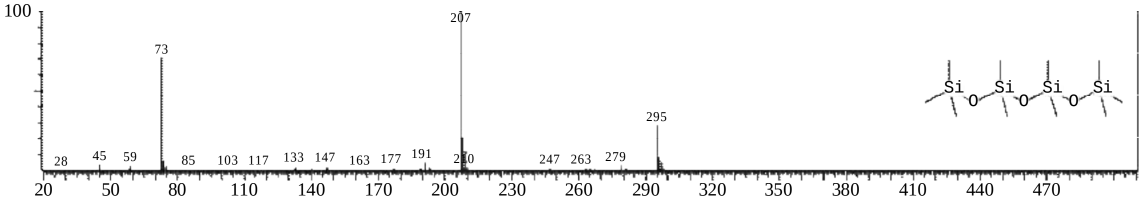

Hit#:2 Entry:24157 Library:NIST05s.LIB
SI:66 Formula:C10H30O3Si4 CAS:141-62-8 MolWeight:310 RetIndex:883
CompName:Tetrasiloxane, decamethyl- $$ Decamethyltetrasiloxane $$ [(CH3)3SiOSi(CH3)2]2O $$ CD3780 $$ D3780 $$ 1,1,1,3,3,5,5,7,7,7-Decamethyltetras

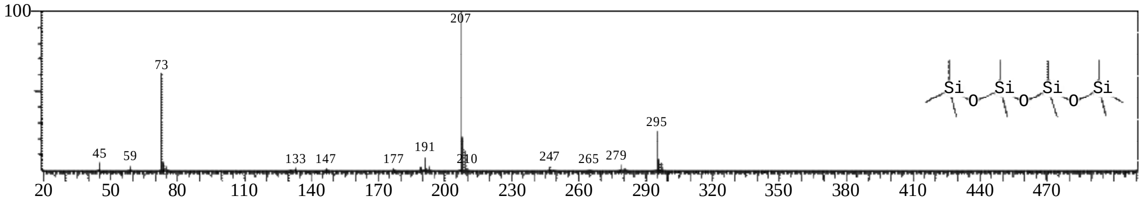

Hit#:3 Entry:71033 Library:NIST05.LIB
SI:66 Formula:C13H22OSi2 CAS:0-00-0 MolWeight:250 RetIndex:1354
CompName:2,4,6-Cycloheptatrien-1-one, 3,5-bis-trimethylsilyl-

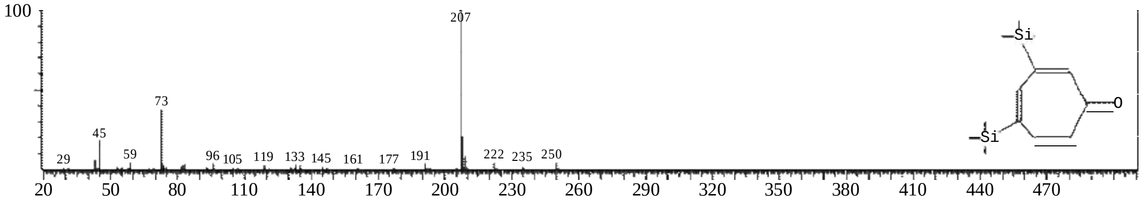

Hit#:4 Entry:79524 Library:NIST05.LIB
SI:66 Formula:C15H24O2Si CAS:0-00-0 MolWeight:264 RetIndex:1640
CompName:Trimethyl[4-(2-methyl-4-oxo-2-pentyl)phenoxy]silane

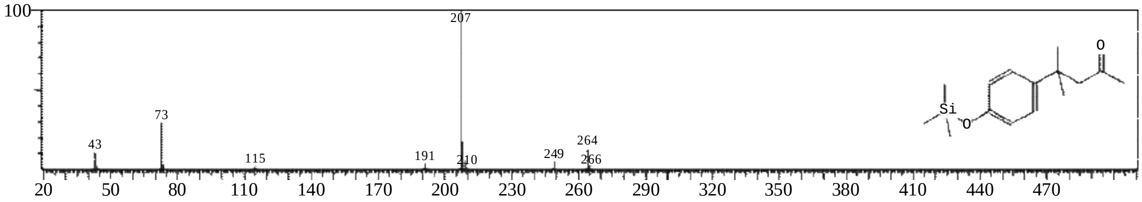

Hit#:5 Entry:54286 Library:NIST05.LIB
SI:66 Formula:C13H22OSi CAS:55012-80-1 MolWeight:222 RetIndex:1339
CompName:Silane, trimethyl[5-methyl-2-(1-methylethyl)phenoxy]- $$ Thymol-TMS $$ (2-Isopropyl-5-methylphenoxy)(trimethyl)silane # $$

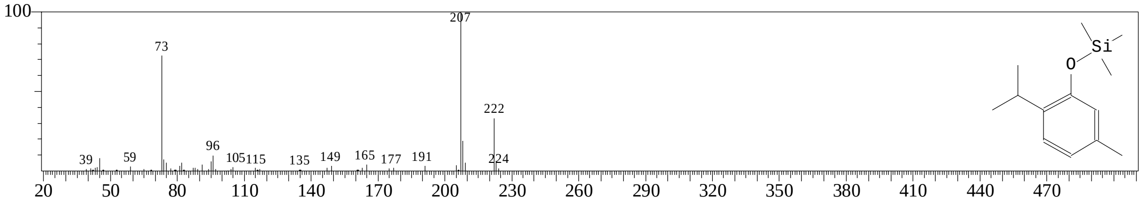

Supplement: S1 File — (PDF) [file pone.0330939.s001.pdf]
