# Supplementary material for: Phytochemical and pharmacoinformatics analysis of a traditional antipsoriatic oil formulation for its potential against proinflammatory cytokines TNF-α and IL-17A
Source: PLoS One. 2025 Sep 2;20(9):e0330939. doi: 10.1371/journal.pone.0330939 (PMC12404448; doi:10.1371/journal.pone.0330939)
Supplement: S2 File — (PDF) [file pone.0330939.s002.pdf]

Title: Phytochemical and pharmacoinformatics analysis of a traditional antipsoriatic oil formulation for its potential against proinflammatory cytokines TNF-α and IL-17A.

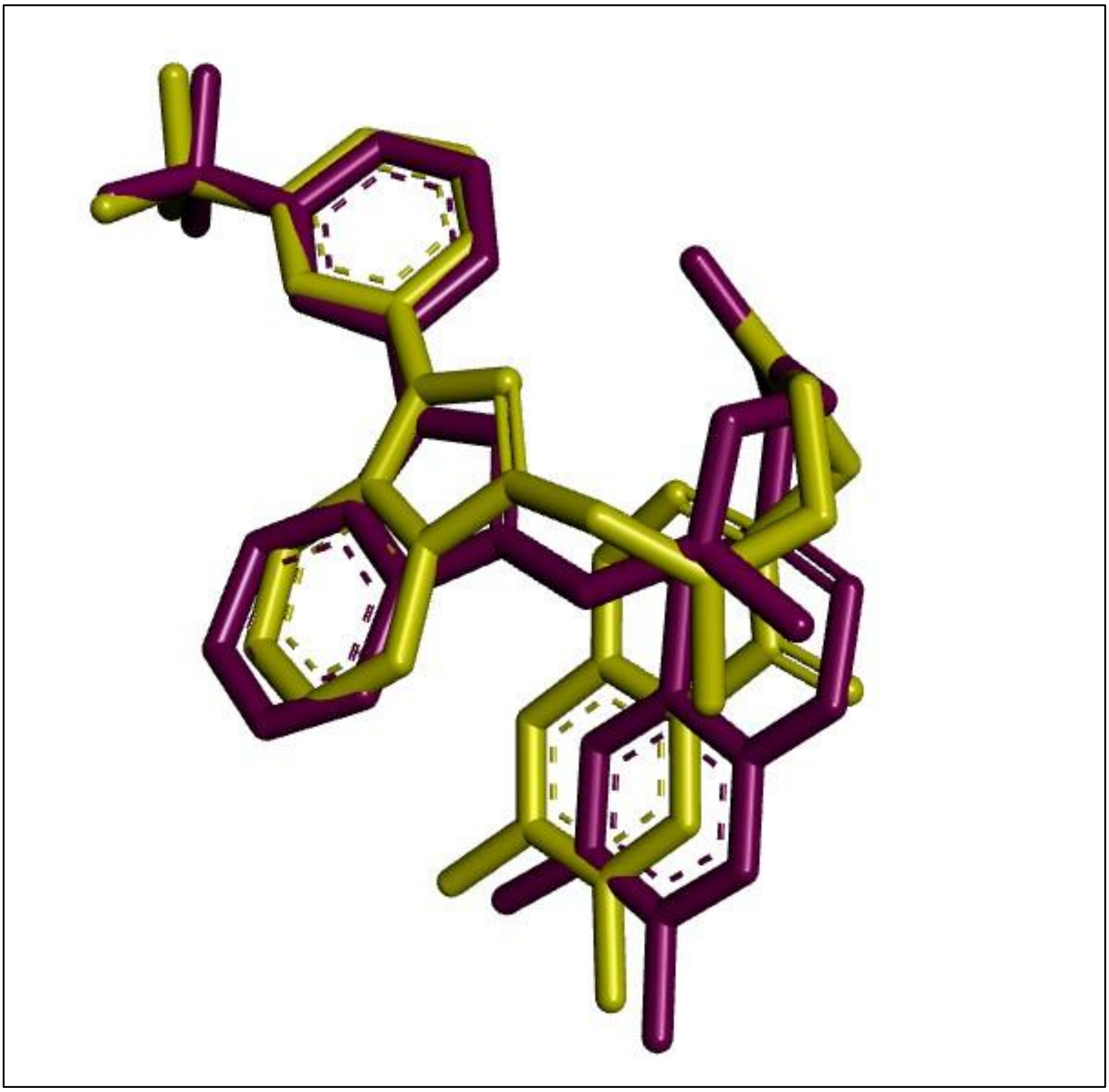

S1 Fig: Validation of docking method: Superimposition of the native (represented in yellow) and re-docked (represented in magenta) ligand of TNF-α (RMSD 1.610 Å).

DockRMSD Results

```
##########################################################################
# DockRMSD (v1.1): docking pose distance calculation                     #
#                                                                        #
# If you use DockRMSD in your work, please cite:                         #
#                                                                        #
# Bell, E.W., Zhang, Y. DockRMSD: an open-source tool for atom           #
# mapping and RMSD calculation of symmetric molecules through graph      #
# isomorphism. Journal of Cheminformatics, 11:40 (2019).                 #
##########################################################################

Calculated Docking RMSD: 1.610

Total # of Possible Mappings: 2985984
Optimal mapping (First file -> Second file, * indicates correspondence is not one-to-one):
C 1 -> C 1
C 2 -> C 2
C 3 -> C 3
N 4 -> N 4
C 5 -> C 5
C 6 -> C 6
C 7 -> C 7
C 8 -> C 8
C 9 -> C 9
C 10 -> C 10
C 11 -> C 11
C 12 -> C 12
C 13 -> C 13
C 14 -> C 14
C 15 -> C 15
C 16 -> C 16
C 17 -> C 17
F 18 -> F 20 *
F 19 -> F 18 *
F 20 -> F 19 *
N 21 -> N 21
C 22 -> C 22
C 23 -> C 23
C 24 -> C 24
N 25 -> N 25
C 26 -> C 26
C 27 -> C 27
C 28 -> C 28
C 29 -> C 29
O 30 -> O 30
C 31 -> C 31
C 32 -> C 32
C 33 -> C 33
C 34 -> C 34
C 35 -> C 35
C 36 -> C 36
C 37 -> C 37
C 38 -> C 38
C 39 -> C 39
O 40 -> O 40
```

S2 Fig: Validation of docking method: Distance measured between two binding poses (Native and redocked ligand) of crystal structure of TNF-α using DockRMSD server, calculated docking RMSD is 1.610 Å.

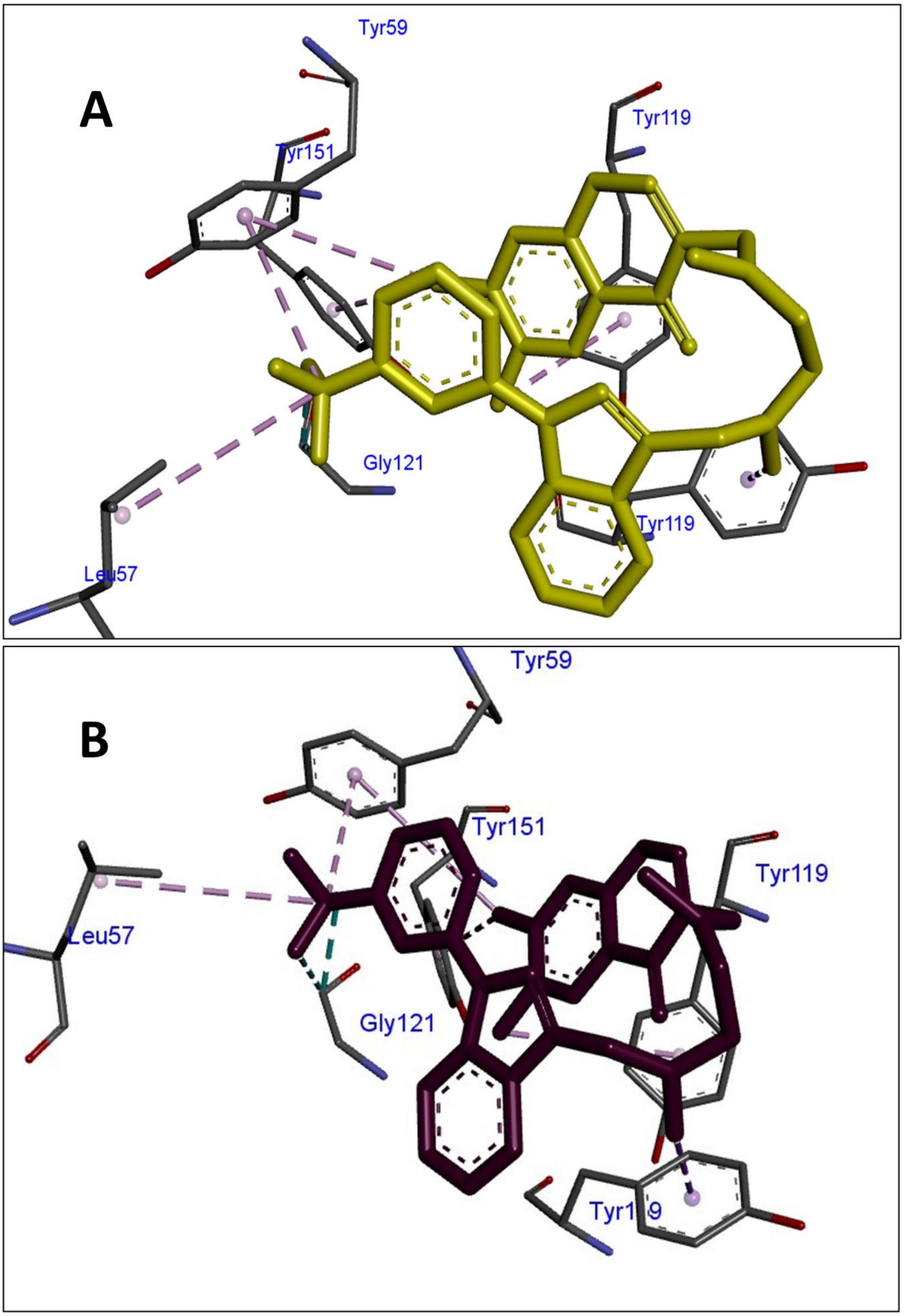

S3 Fig: Validation of docking method: Binding orientation and interactions of A) Native ligand with TNF-α B) Re-docked ligand with TNF-α.

	Name	Visible	Color	Parent	Distance	Category	Types	From	From Chemistry
1	C:TYR151:OH - C:3072:O34	Yes		Ligand No...	2.93491	Hydrogen Bo...	Conventi...	C:TYR15...	H-Donor
2	C:GLY121:C - C:3072:F9	Yes		Ligand No...	3.56226	Halogen	Halogen ...	C:GLY12...	Halogen Acceptor
3	C:3072:C28 - C:TYR119	Yes		Ligand No...	3.76334	Hydrophobic	Pi-Sigma	C:3072:...	C-H
4	C:3072:C37 - C:TYR59	Yes		Ligand No...	3.88539	Hydrophobic	Pi-Sigma	C:3072:...	C-H
5	C:TYR59 - C:3072	Yes		Ligand No...	5.46043	Hydrophobic	Pi-Pi Stac...	C:TYR59	Pi-Orbitals
6	C:TYR59 - C:3072	Yes		Ligand No...	4.13932	Hydrophobic	Pi-Pi Stac...	C:TYR59	Pi-Orbitals
7	D:TYR59 - C:3072	Yes		Ligand No...	5.12181	Hydrophobic	Pi-Pi Stac...	D:TYR59	Pi-Orbitals
8	C:3072:C7 - D:LEU57	Yes		Ligand No...	5.07278	Hydrophobic	Alkyl	C:3072:C7	Alkyl
9	D:TYR59 - C:3072:C7	Yes		Ligand No...	3.91519	Hydrophobic	Pi-Alkyl	D:TYR59	Pi-Orbitals

A

	Name	Visible	Color	Parent	Distance	Category	Types	From	From Chemistry
1	C:GLY121:C - C:3072:F9	Yes		Ligand No...	3.62057	Halogen	Halogen ...	C:GLY12...	Halogen Acceptor
2	C:GLY121:C - C:3072:F8	Yes		Ligand No...	2.82162	Halogen	Halogen ...	C:GLY12...	Halogen Acceptor
3	C:3072:C22 - C:TYR119	Yes		Ligand No...	3.35587	Hydrophobic	Pi-Sigma	C:3072:...	C-H
4	C:3072:C7 - C:LEU57	Yes		Ligand No...	5.25185	Hydrophobic	Alkyl	C:3072:C7	Alkyl
5	D:TYR59 - C:3072:C7	Yes		Ligand No...	4.50701	Hydrophobic	Pi-Alkyl	D:TYR59	Pi-Orbitals
6	D:TYR59 - C:3072:C39	Yes		Ligand No...	4.94006	Hydrophobic	Pi-Alkyl	D:TYR59	Pi-Orbitals
7	D:TYR119 - C:3072:C37	Yes		Ligand No...	4.88271	Hydrophobic	Pi-Alkyl	D:TYR119	Pi-Orbitals
8	D:TYR151 - C:3072:C39	Yes		Ligand No...	5.02518	Hydrophobic	Pi-Alkyl	D:TYR151	Pi-Orbitals

B

S4 Fig: A) Interacting residue information of redocked ligand against TNF-α. B) Interacting residue information of native ligand against TNF-α.

A

	Name	Visible	Color	Parent	Distance	Category	Types	From	From Chemistry	To	To Chemistry	Angle XDA	Angle DAY	Angle DHA	Angle HAY	Theta
1	A:LEU97:N - B:63Q4000:O2	Yes		Ligand No...	2.78609	Hydrogen Bo...	Conventi...	A:LEU97:N	H-Donor	B:63...	H-Acceptor	25.173	138.864			
2	B:TRP67:N - B:63Q4000:O1	Yes		Ligand No...	2.94483	Hydrogen Bo...	Conventi...	B:TRP67:N	H-Donor	B:63...	H-Acceptor	15.284	144.658			
3	B:LEU97:N - B:63Q4000:O4	Yes		Ligand No...	2.70891	Hydrogen Bo...	Conventi...	B:LEU97:N	H-Donor	B:63...	H-Acceptor	13.15	148.394			
4	B:63Q4000:H12 - B:LEU97:O	Yes		Ligand No...	2.4709	Hydrogen Bo...	Conventi...	B:63Q40...	H-Donor	B:LEU...	H-Acceptor			143.399	168.65	
5	B:63Q4000:H28 - A:LEU97:O	Yes		Ligand No...	1.97024	Hydrogen Bo...	Conventi...	B:63Q40...	H-Donor	A:LE...	H-Acceptor			164.915	159.229	
6	B:TRP67:CD1 - B:63Q4000:O1	Yes		Ligand No...	3.52104	Hydrogen Bo...	Carbon H...	B:TRP67...	H-Donor	B:63...	H-Acceptor	29.552	121.287			
7	B:63Q4000:H32 - B:GLU95:O	Yes		Ligand No...	2.72064	Hydrogen Bo...	Carbon H...	B:63Q40...	H-Donor	B:GL...	H-Acceptor			100.306	131.338	
8	B:63Q4000:H13 - B:LEU97:O	Yes		Ligand No...	2.28771	Hydrogen Bo...	Carbon H...	B:63Q40...	H-Donor	B:LEU...	H-Acceptor			155.625	129.373	
9	A:ILE96:CD1 - B:63Q4000	Yes		Ligand No...	3.65272	Hydrophobic	Pi-Sigma	A:ILE96:...	C-H	B:63...	Pi-Orbitals					19.147
10	A:LEU97:CD1 - B:63Q4000	Yes		Ligand No...	3.56105	Hydrophobic	Pi-Sigma	A:LEU97...	C-H	B:63...	Pi-Orbitals					18.63
11	B:ILE96:CD1 - B:63Q4000	Yes		Ligand No...	3.88029	Hydrophobic	Pi-Sigma	B:ILE96:...	C-H	B:63...	Pi-Orbitals					23.744
12	A:LEU97 - B:63Q4000	Yes		Ligand No...	5.11871	Hydrophobic	Alkyl	A:LEU97	Alkyl	B:63...	Alkyl					
13	B:PRO63 - B:63Q4000	Yes		Ligand No...	4.30039	Hydrophobic	Alkyl	B:PRO63	Alkyl	B:63...	Alkyl					
14	B:63Q4000:C1 - B:PRO37	Yes		Ligand No...	4.2289	Hydrophobic	Alkyl	B:63Q40...	Alkyl	B:PR...	Alkyl					
15	B:TRP67 - B:63Q4000:C1	Yes		Ligand No...	4.46767	Hydrophobic	Pi-Alkyl	B:TRP67	Pi-Orbitals	B:63...	Alkyl					
16	B:63Q4000 - A:PRO63	Yes		Ligand No...	4.54904	Hydrophobic	Pi-Alkyl	B:63Q40...	Pi-Orbitals	A:PR...	Alkyl					
17	B:63Q4000 - B:PRO63	Yes		Ligand No...	5.0975	Hydrophobic	Pi-Alkyl	B:63Q40...	Pi-Orbitals	B:PR...	Alkyl					
18	B:63Q4000 - B:PRO63	Yes		Ligand No...	4.51095	Hydrophobic	Pi-Alkyl	B:63Q40...	Pi-Orbitals	B:PR...	Alkyl					
19	B:63Q4000 - A:LEU97	Yes		Ligand No...	5.14782	Hydrophobic	Pi-Alkyl	B:63Q40...	Pi-Orbitals	A:LE...	Alkyl					
20	B:63Q4000 - B:PRO63	Yes		Ligand No...	5.31617	Hydrophobic	Pi-Alkyl	B:63Q40...	Pi-Orbitals	B:PR...	Alkyl					

B

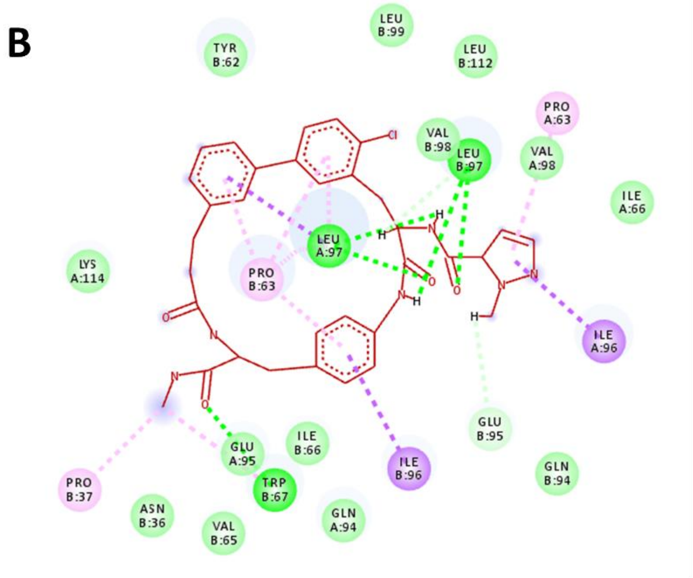

S5 Fig: A) Interacting residue information of native macrocyclic ligand (compound 1) complexed with IL17A (5HI5). B) 2D view of interactions of native macrocyclic ligand (compound 1) complexed with IL17A (5HI5).

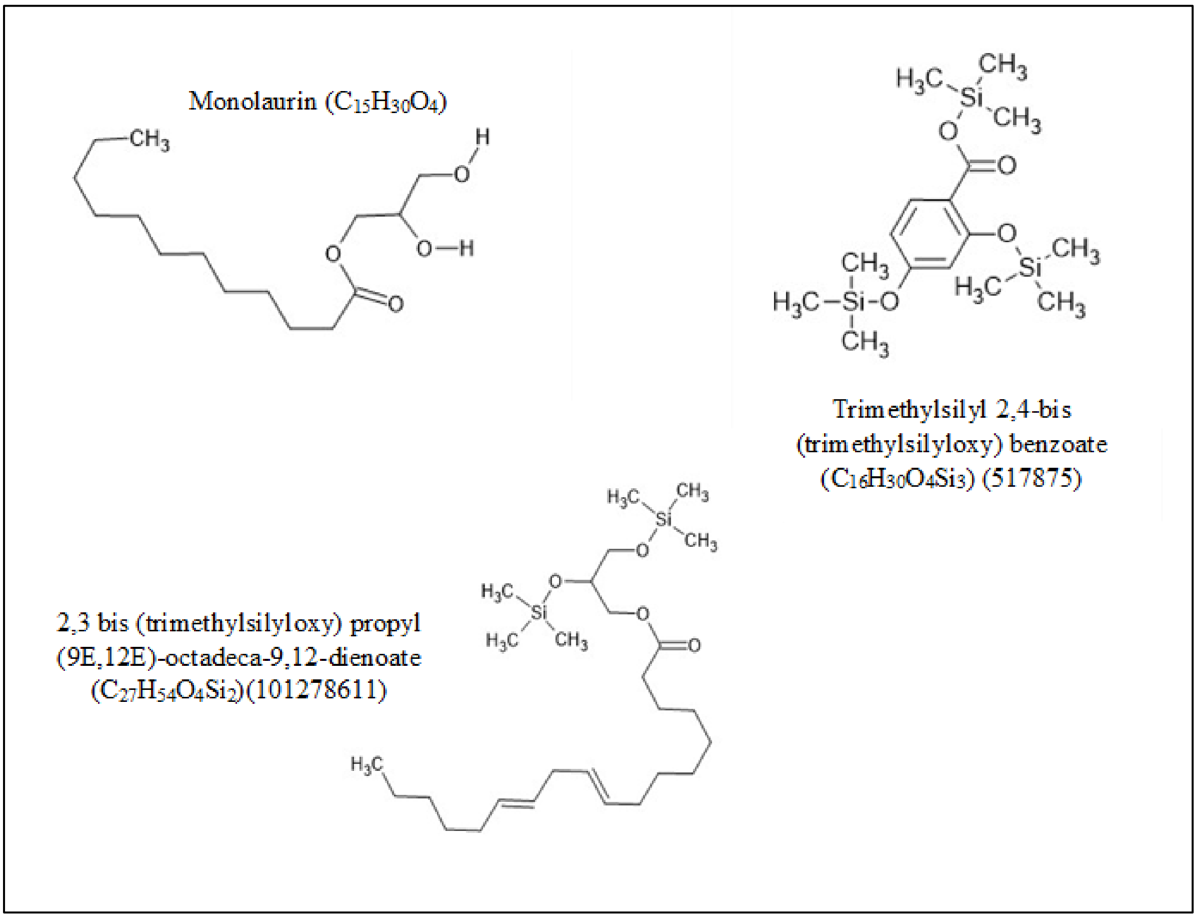

S6 Fig: The 2D structures of the shortlisted ligands demonstrated the lowest binding energies and interactions with key residues of IL-17A.

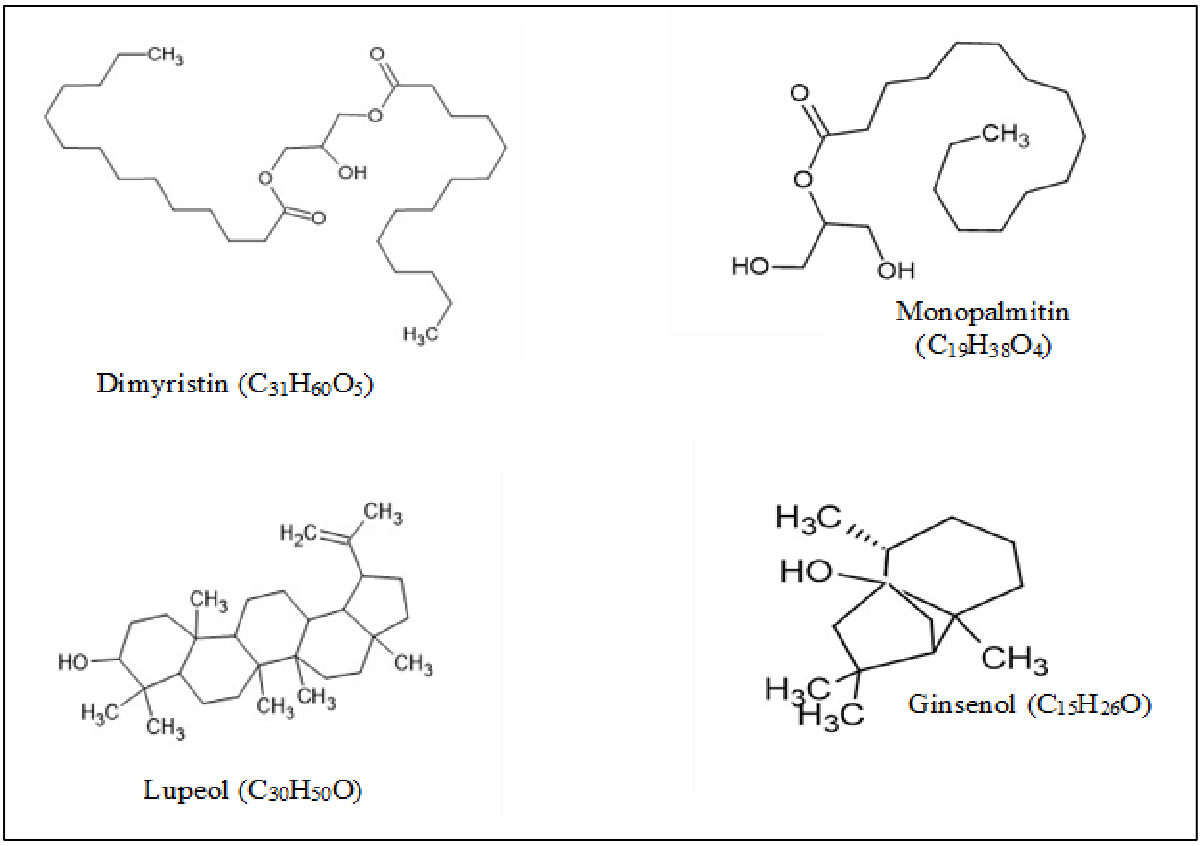

S7 Fig: The 2D structures of the shortlisted ligands demonstrated the lowest binding energies and interactions with key residues of TNF-α.

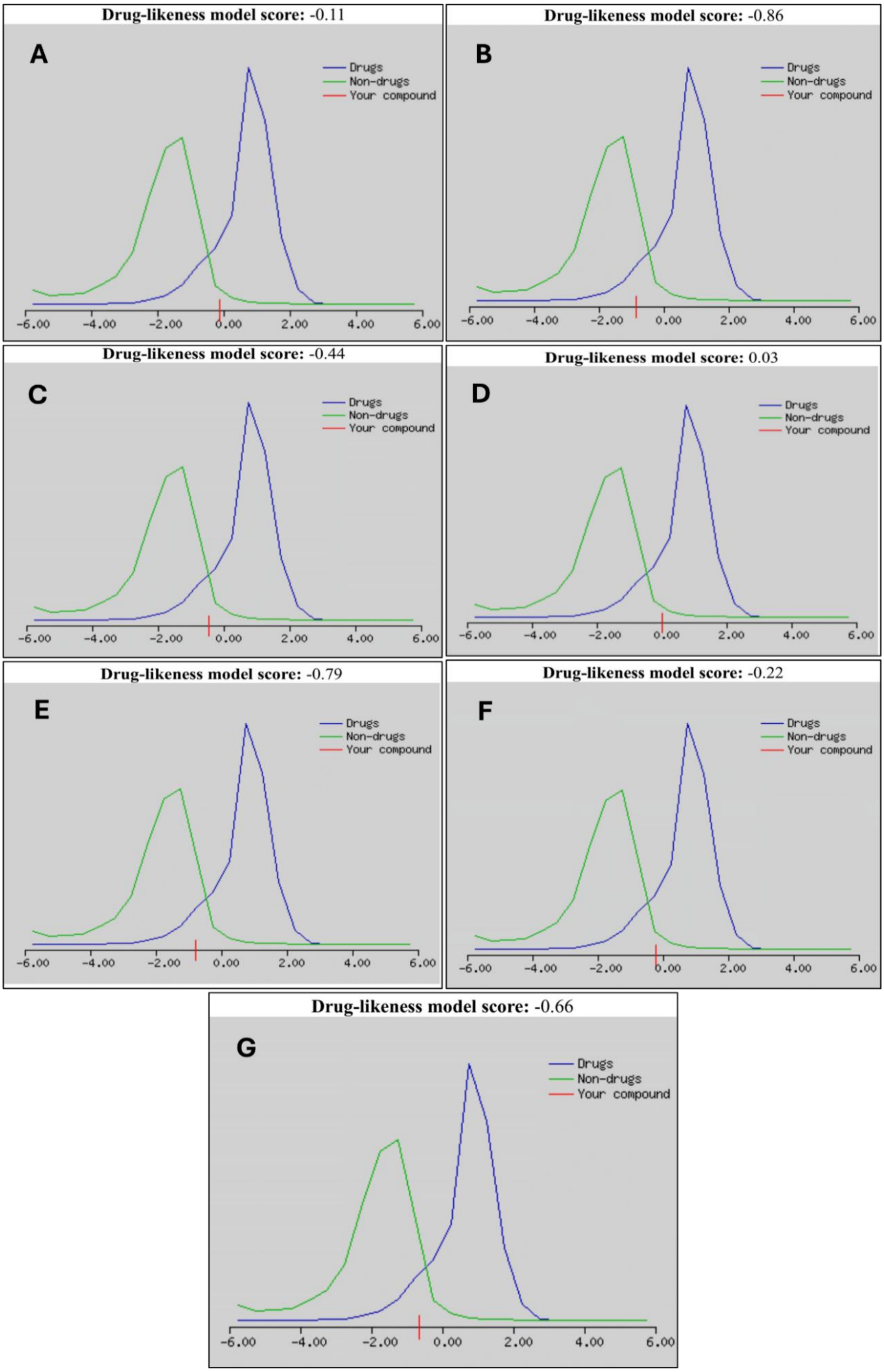

S8 Fig: Drug-likeness scores of shortlisted ligands from VT. A) Monolaurin, B) Trimethylsilyl 2,4-bis(trimethylsilyloxy)benzoate, C) 2,3-bis(trimethylsilyloxy)propyl (9E,12E)-octadeca- 9,12-dienoate, D) Dimyristin, E) 2-Monopalmitin, F) Lupeol, G) Ginsenol.
