# Supplementary material for: Phytochemical and pharmacoinformatics analysis of a traditional antipsoriatic oil formulation for its potential against proinflammatory cytokines TNF-α and IL-17A
Source: PLoS One. 2025 Sep 2;20(9):e0330939. doi: 10.1371/journal.pone.0330939 (PMC12404448; doi:10.1371/journal.pone.0330939)
Supplement: S3 File — (PDF) [file pone.0330939.s003.pdf]

Title: Phytochemical and pharmacoinformatics analysis of a traditional antipsoriatic oil formulation for its potential against proinflammatory cytokines TNF-α and IL-17A

S1 Table: List of phytoconstituents identified from GC-MS analysis of sample VT oil.

Sl. No	R. time (min)	Compound Name	Area %	Formula	Molecular weight (Da)	CAS number
1	31.934	Capric acid triglyceride	14.52	C33H62O6	554.8418	621-71-6
2	32.101	Lauric acid triglyceride	13.9	C39H74O	639.0013	538-24-9
3	26.95	Dilaurin	13.59	C27H52O5	456.7	17598-94-6
4	12.594	Lauric acid	12.22	C12H24O2	200.3178	143-07-7
5	28.829	Glycerol tricaprylate	7.3	C27H50O6	470.6823	538-23-8
6	7.411	Caprylic acid	6.26	C8H16O2	144.2114	124-07-2
7	14.814	Myristic acid	5.77	C14H28O2	228.3709	544-63-8
8	29.465	Dimyristin	4.61	C31H60O5	512.8	04-09-7770
9	4.611	3-Hexen-2-one	3.77	C6H10O	98.143	763-93-9
10	25.107	2,3-di(tetradecanoyloxy) propyl octadecanoate	3.56	C49H94O6	779.3	2177-98-2
11	10.054	Capric acid	1.98	C10H20O2	172.2646	334-48-5
12	33.036	1,2-Dipalmitin	1.43	C35H68O5	568.9	40290-32-2
13	15.406	1,1,3,3,5,5,7,7,9,9,11,11,13,13, 15,15-Hexadecamethyloctasiloxane	1.07	C16H48O7 Si8	577.2	19095-24-0
14	16.858	Palmitic acid	0.89	C15H30O2	242.3975	1002-84-2
15	18.192	Monolaurin	0.68	C15H30O4	274.3963	142-18-7
16	4.035	Deoxymevalonic acid	0.66	C6H12O3	132.1577	150-96-9
17	32.579	Lupeol	0.63	C30H50O	426.7174	545-47-1
18	4.39	2-hydroperoxyhexane	0.59	C6H14O2	118.1742	24254-55-5
19	4.946	1,1,2,3-tetramethylcyclopropane	0.56	C7H14	98.19	74752-93-5
20	18.56	Oleic Acid	0.55	C18H34O2	282.4614	112-80-1
21	3.813	5-methoxypentan-2-one	0.51	C6H12O2	116.16	17429-04-8
22	4.273	3-hydroperoxyhexane	0.49	C6H14O2	118.1742	24254-56-6
23	22.193	tridecan-3-yl hexanoate	0.47	C19H38O2	298.5	-
24	13.653	2,2,4,4,6,6,8,8,10,10,12,12,14, 14,16,16-hexadecamethyl-1,3,5,7,9,11,13,15-octaoxa-2,4,6,8,10,12,14,16-octasilacyclohexadecane	0.35	C16H48O8 Si8	593.2315	556-68-3
25	8.73	(E)-tridec-2-enal	0.27	C13H24O	196.3291	7069-41-2
26	11.561	Propan-2-yl bis(trimethylsilyl) tris(trimethylsilyloxy)silyl silicate	0.26	C18H52O7 Si7	577.2	-
27	13.985	Butyl caprylate	0.25	C12H24O2	200.3178	589-75-3
28	9.162	delta. Nonalactone	0.23	C9H16O2	156.22	3301-94-8
29	20.025	Lginseno	0.19	C19H38O4	330.5026	23470-00-0

30	30.782	2,2,4,4,6,6-hexamethyl-1,3,5,2,4,6-trioxatrisilinane	0.18	C6H18O3Si3	222.4618	541-05-9
31	3.151	Hexanal	0.15	C6H12O	100.1589	66-25-1
32	11.382	2,2,4,4,6,6,8,8,10,10,12,12,14,14-tetradecamethyl-1,3,5,7,9,11,13-heptaoxa-2,4,6,8,10,12,14-heptasilacyclotetradecane	0.11	C14H42O7Si7	519.0776	107-50-6
33	5.657	Cyclohexyl nitrite	0.11	C6H11NO2	129.157	5156-40-1
34	18.743	Octadecanoic acid	0.1	C18H36O2	284.4772	57-11-4
35	10.688	Z-2-Dodecenol	0.1	C12H24O	184.3184	69064-36-4
36	22.094	Tridecan-4-yl hexanoate	0.09	C19H38O2	298.5	-
37	17.486	Glyceryl laurate diacetate	0.09	C19H34O6	358.4697	55191-44-1
38	14.193	1,5-Anhydro-d-talitol	0.09	-	-	-
39	11.91	Delta-Decalactone	0.09	C10H18O2	170.2487	705-86-2
40	3.649	Heptan-2-one	0.09	C7H14O	114.1855	110-43-0
41	15.948	Butoxymethyl(trimethyl)silane	0.08	C8H20OSi	160.33	
42	13.543	Trimethylsilyl 2,4-bis(trimethylsilyloxy)benzoate	0.07	C16H30O4Si3	370.6635	10586-16-0
43	7.808	Azulene	0.07	C10H8	128.1705	275-51-4
44	5.083	9-Oxabicyclo [6.1.0] nonane	0.07	C8H14O	126.1962	286-62-4
45	31.016	Diethyl bis(trimethylsilyl) silic ate	0.06	C10H28O4Si3	296.58	3555-45-1
46	29.307	(E)-3-tert-butyloct-6-en-1-ol	0.06	C12H24O	184.32	-
47	33.376	[dimethyl(trimethylsilyloxy)silyl] oxy-dimethyl-trimethylsilyloxysilane	0.05	C10H30O3Si4	310.6854	141-62-8
48	31.13	1,1,3,3,5,5,7,7,9,9,11,11-Dodecamethylhexasiloxane	0.05	C12H36O5Si6	428.92	995-82-4
49	28.629	Trimethyl-(4-trimethylsilylphenyl) silane	0.05	C12H22Si2	222.4741	13183-70-5
50	21.715	5-Octadecenal octadec-5-enal	0.05	C18H34O	266.462	56554-88-2
51	16.504	Methyl tuberuculostearate	0.05	-	-	-
52	29.671	2,3-bis(trimethylsilyloxy)propyl (9E,12E)-octadeca-9,12-dienoate	0.04	C27H54O4Si2	498.8863	54284-45-6
53	23.927	2,2,4,4,6,6,8,8,10,10,12,12,14,14,16,16,18,18-octadecamethyl-1,3,5,7,9,11,13,15,17-nonaoxa-2,4,6,8,10,12,14,16,18-nonasilacyclooctadecane	0.04	C18H54O9Si9	667.3855	556-71-8
54	20.468	Vinyl caprylate	0.04	C10H18O2	-	818-44-0
55	16.636	δ-lactone	0.04	C18H34O2	282.4614	1227-51-6
56	12.922	Ethyl icosanoate	0.04	C22H44O2	340.5836	18281-05-5
57	32.736	propan-2-yl tris(trimethylsilyl) silicate	0.03	C12H34O4Si4	354.74	72182-11-7

58	25.499	Ethyl tris(trimethylsilyl) silicate	0.03	C11H32O4 Si4	340.71	18030-67-6
59	24.069	1,1,3,3,5,5,7,7,9,9,11,11,13,13-Tetradecamethylheptasiloxane	0.03	C14H42O6 Si7	503.07	19095-23-9
60	23.116	Ginsenol	0.03	C15H26O	222.3663	117591-80-7
61	21.452	2,3,4,4a,5,6,7,8-octahydro-1H-naphthalene	0.03	C15H28	208.3828	30824-81-8
62	11.772	2,6,11-trimethyldodecane	0.03	C15H32	212.4146	31295-56-4
63	9.501	(2E,4E)-deca-2,4-dienal	0.03	C10H16O	152.2334	25152-84-5
64	8.535	(E)-3-ethenyloct-2-en-1-ol	0.03	C10H18O	154.25	-
65	6.487	Nonanal	0.03	C9H18O	142.2386	124-19-6
**Note:** R. Time – Retention time, CAS number – Chemical Abstract Number, min-minutes, Da- Daltons						

S2 Table: Physical characterization and fulfilment of Lipinski's rule of five of shortlisted ligands from VT.

Compounds	Molecular weight	TPSA	Rotatable Bonds	H Bonds donors	H Bonds acceptors	Lipinski's rule
Monolaurin	274.21g/mol	66.76	14	2	4	Yes
Trimethylsilyl 2,4-bis(trimethylsilyloxy)benzoate	370.15g/mol	44.76	7	1	4	Yes
2,3-bis(trimethylsilyloxy)propyl (9E,12E)-octadeca-9,12-dienoate	498.36g/mol	44.76	22	0	4	Yes
Dimyristin	512.44g/mol	72.83	30	1	5	No
2-Monopalmitin	330.28g/mol	66.76	18	2	4	Yes
Lupeol	426.39g/mol	20.23	1	1	1	Yes
Ginsenol	222.37g/mol	20.23	0	1	1	Yes
**Note:** TPSA=Topological polar surface area, cut-off values: Lipinski's rule – MW ≤ 500, No of H bond acceptor - ≤ 10, No of H bond donor - ≤ 5 (If two properties are out of range a poor absorption or permeability is possible, one is acceptable)						

S3 Table: Predicted bioactivity scores of shortlisted ligands from VT oil.

Compounds	Nuclear receptor ligand	Protease inhibitor	Enzyme inhibitor
Monolaurin	-	-	-
Trimethylsilyl 2,4-bis(trimethylsilyloxy)benzoate	-	+	+
2,3-bis(trimethylsilyloxy)propyl (9E,12E)-octadeca-9,12-dienoate	-		+
Dimyristin	-	-	-
2-Monopalmitin	-	-	-
Lupeol	+	-	+
Ginsenol	-	-	-

Note: +=Present, - = Absent

S4 Table: Permeability, absorption, and distribution predictions of shortlisted ligands from VT oil.

Compounds	Permeability			Absorption			Distribution	
	Mol LogP	Mol LogS	Bio-availability score	CaCO2 permeability	P-glycoprotein substrate	GI Absorption	PPB	BBB Penetration
Monolaurin	4.13	-3.99	0.55	-4.683	No	High	92.79%	+
Trimethylsilyl 2,4-bis(trimethylsilyloxy)benzoate	5.93 (> 5)	-5.58	0.55	-5.326	Yes	High	97.70%	-
2,3-bis(trimethylsilyloxy)propyl (9E,12E)-octadeca-9,12-dienoate	10.27 (> 5)	-5.82	0.55	-4.811	No	Low	100%	-
Dimyristin	11.65 (> 5)	-5.95	0.17	-5.113	No	Low	96.30%	-
2-Monopalmitin	6.00 (> 5)	-5.23	0.55	-4.752	No	High	95.95%	-
Lupeol	8.35 (> 5)	-6.31	0.55	-5.02	No	Low	98.80%	+
Ginsenol	3.64	-3.82	0.55	-4.627	No	High	96.11%	-

Note: MolLogP - Logarithm of partitioning coefficient between n-octanol and water phases (0-3), Mol LogS - Logarithm of aqueous solubility (-4 – 0.5log moL/L), BBB - (blood–brain barrier) permeation, PPB - Plasma Protein Binding (optimal <90%), CaCO2 – human colon adenocarcinoma cell line (optimal > -5.15 log unit)

S5 Table: predictions of scores related to excretion and toxicity of shortlisted ligands from VT oil.

**Compounds**	**Excretion**		**Toxicity**			
	**Cl**	**T1/2**	**DILI**	**AMES**	**Carcinogenicity**	**LD50**
Monolaurin	9.45	0.825	-	-	Absent	5000mg/kg
Trimethylsilyl 2,4-bis(trimethylsilyloxy)benzoate	2.958	0.346	-	-	Absent	1250mg/kg
2,3-bis(trimethylsilyloxy)propyl (9E,12E)-octadeca-9,12-dienoate	3.424	0.155	-	-	Absent	39800mg/kg
Dimyristin	5.596	0.211	-	-	Present	5000mg/kg
2-Monopalmitin	6.212	0.768	-	-	Absent	5000mg/kg
Lupeol	17.929	0.01	-	-	Absent	2000mg/kg
Ginsenol	11.353	0.101	-	-	Absent	2000mg/kg

Note: Cl – Clearance (>15 mL/min/kg – High, 5-15 mL/min/kg – moderate, <5 mL/min/kg - low), T1/2 – Half-life (>3 long half-life, <3 short half-life), DILI - Drug Induced Liver Injury, (+) present (-) absent (output value is probability of being toxic)

S6 Table: List of identified pharmacological properties of some of the phytoconstituents present in VT oil from the literature.

Sl. No	Name of the phytochemical	Pharmacological properties
**1**	(2E,4E)-deca-2,4-dienal	Nematocidal Activity [1]
**2**	Azulene	Anti-inflammatory activity- inhibited the production of TNF-α, IL-1β and IL-8 in lipopolysaccharide-activated THP-1 macrophages [2]
**3**	Trimethylsilyl 2,4-bis(trimethylsilyloxy) benzoate	Antifungal, anti-bacterial [3]
**4**	Lupeol	• Anti-inflammatory - decreased the generation of pro-inflammatory cytokines such as TNFα and ILβ in lipopolysaccharide treated macrophages, antidiabetic activities [4]<br>• Inhibited phosphatidyl inositol 3-kinase (PI3K), Akt, Nuclear factor kappa B (NFkB) and activated Inhibitory-kB kinase-((IKK-(α) in human lung cancer cell line [5]
**5**	Nonanal	Antidiarrhoeal activity [6]<br>Antifungal activity [7]
**6**	2,3-bis(trimethylsilyloxy) propyl (9Z,12Z)-octadeca-9,12-dienoate	Antioxidant, anticancer and antimicrobial activities [8]

**7**	Capric acid	Anti-inflammatory:Alleviated the production of TNF-α and IL-6 inflammatory and gene expression of NF-κB, TNF-α and IFN-γ,in cyclophosphamide-treated IPEC-J2 cells [9]<br>Significantly reduced IL-6 and IL-8 production in P. acnes-stimulated SZ95 sebocytes. Suppressed mRNA levels and secretion of IL-8 and TNF-a in THP-1 cells, further inhibited NF-kB activation and phosphorylation of MAP kinases [10]
**8**	Caprylic acid	Suppressed inflammation and ameliorated atherosclerosis in Apo E deficient mice (apoE −/− mice) through TLR4/NF-κB signalling pathway [11]
**9**	Lauric acid	Stimulated the phosphorylation of EGFR, ERK and c-Jun and induced the expression of c-fos in breast and endometrial cancer cells [12]
**10**	Oleic acid	Inhibited NF-κB pathway in ECs (endothelial cells), VSMCs (vascular smooth<br>muscle cells) and Cardiomyocytes [13]
**11**	Palmitic acid	Showed epidermal morphogenesis in invitro developed human skin equivalents (HSE's) [14]
**12**	Stearic acid	Anti-inflammatory:<br>• Attenuated Bile duct ligation (BDL)-induced leukocyte accumulation and NF-kB activation in cholestasis induced liver injury in rats [15]
**13**	Myristic acid	Protected the testes against oxidative stress via preventing the upregulation of RAGE, Keap1, and the downregulation of Nrf2, NQO1, HO1, SOD, CAT and GPx. MA also prevented increase in testicular inflammation and apoptosis, as indicated by low inflammatory (NF-κB p65, IKKβ, TNF-α, IL-1β and iNOS) and apoptosis (Bax and caspase-9) [16]

**14**	Monolaurin	Antibacterial activity [17]
**15**	Ginsenol	Antiviral activity [18]

References for S6 Table:

1. Caboni P, Ntalli NG, Aissani N, Cavoski I, Angioni A. Nematicidal activity of (E,E)-2,4-decadienal and (E)-2-decenal from Ailanthus altissima against Meloidogyne javanica. J Agric Food Chem. 2012;60(4):1146–51. https://doi.org/10.1021/jf2044586

2. Bakun P, Czarczynska-Goslinska B, Goslinski T, Lijewski S. In vitro and in vivo biological activities of azulene derivatives with potential applications in medicine. Med Chem Res. 2021;30:834–46. https://doi.org/10.1007/s00044-021-02701-0

3. Carvalho PL, et al. Paraconiothyrium sp. P83F4/1: antioxidant and antiproliferative activities of endophytic fungus associated with Rheedia brasiliensis plant. Int J Biotechnol Wellness Ind. 2012;1:172–6. https://doi.org/10.6000/1927-3037/2012.01.03.03

4. Sharma N, Palia P, Chaudhary A, Shalini, Verma K, Kumar I. A review on pharmacological activities of lupeol and its triterpene derivatives. J Drug Deliv Ther. 2020;10(5):325–32. https://doi.org/10.22270/jddt.v10i5.4280

5. Dwivedi N, Dwivedi B, Mishra S, Shukla Y. Lupeol induced apoptosis in human lung cancer cell line: a flow cytometry study. Res J Pharmacol Pharmacodyn. 2014;6:197–203.

6. Zavala-Sánchez MA, Gutiérrez SP, González CP, Saldivar DS, García LA. Antidiarrhoeal activity of nonanal, an aldehyde isolated from Artemisia ludoviciana. Pharm Biol. 2002;40:263–8. https://doi.org/10.1076/phbi.40.4.263.8465

7. Zhang JH, Sun HL, Chen SY, Zeng L, Wang TT. Anti-fungal activity, mechanism studies on α-phellandrene and nonanal against Penicillium cyclopium. Bot Stud. 2017;58:13. https://doi.org/10.1186/s40529-017-0168-8

8. Tyagi T, Agarwal M. Phytochemical screening and GC-MS analysis of bioactive constituents in the ethanolic extract of Pistia stratiotes L. and Eichhornia crassipes (Mart.) Solms. J Pharmacogn Phytochem. 2017;6:195–206.

9. Lee SI, Kang KS. Function of capric acid in cyclophosphamide-induced intestinal inflammation, oxidative stress, and barrier function in pigs. Sci Rep. 2017;7:16530. https://doi.org/10.1038/s41598-017-16561-5

10. Huang WC, Tsai TH, Chuang LT, Li YY, Zouboulis CC, Tsai PJ. Anti-bacterial and anti-inflammatory properties of capric acid against Propionibacterium acnes: a comparative study with lauric acid. J Dermatol Sci. 2014;73:232–40. https://doi.org/10.1016/j.jdermsci.2013.10.010

11. Zhang X, et al. Caprylic acid suppresses inflammation via TLR4/NF-κB signaling and improves atherosclerosis in ApoE-deficient mice. Nutr Metab. 2019;16:40. https://doi.org/10.1186/s12986-019-0359-2

12. Lappano R, et al. The lauric acid-activated signaling prompts apoptosis in cancer cells. Cell

Death Discov. 2017;3:17063. https://doi.org/10.1038/cddiscovery.2017.63

13. Perdomo L, et al. Protective role of oleic acid against cardiovascular insulin resistance and in the early and late cellular atherosclerotic process. Cardiovasc Diabetol. 2015;14:75. https://doi.org/10.1186/s12933-015-0237-9

14. Mieremet A, et al. Contribution of palmitic acid to epidermal morphogenesis and lipid barrier formation in human skin equivalents. Int J Mol Sci. 2019;20:6069. https://doi.org/10.3390/ijms20236069

15. Pan PH, et al. Stearic acid attenuates cholestasis-induced liver injury. Biochem Biophys Res Commun. 2010;391:1537–42. https://doi.org/10.1016/j.bbrc.2009.12.119

16. Khalil ASM, et al. Myristic acid defends against testicular oxidative stress, inflammation, apoptosis: restoration of spermatogenesis, steroidogenesis in diabetic rats. Life Sci. 2021;278:119605. https://doi.org/10.1016/j.lfs.2021.119605

17. Mh K, Topchi A. Evaluation of antibacterial effect of monolaurin on *Staphylococcus aureus* isolated from bovine mastitis. Afr J Pharm Pharmacol. 2013;7:1163–6.

18. Volobueva AS, et al. Discovery of new Ginsenol-like compounds with high antiviral activity. Molecules. 2021;26:6794. https://doi.org/10.3390/molecules26226794
