# Supplementary material for: Phytochemical and pharmacoinformatics analysis of a traditional antipsoriatic oil formulation for its potential against proinflammatory cytokines TNF-α and IL-17A
Source: PLoS One. 2025 Sep 2;20(9):e0330939. doi: 10.1371/journal.pone.0330939 (PMC12404448; doi:10.1371/journal.pone.0330939)
Supplement: Attachment — Submitted filename: Response to Reviwers.docx [file pone.0330939.s004.docx]

Dear Editor,

Greetings.

We sincerely thank you for considering our manuscript and for the valuable feedback aimed at its improvement. In response, we have carefully revised the manuscript to comply with the PLOS ONE formatting and submission guidelines. All comments and suggestions provided by the reviewers have been thoroughly addressed.

For your convenience, we have included a point-by-point response table outlining each reviewer’s comment along with our corresponding revisions or clarifications.

Additionally, a revised version of the manuscript is enclosed, with all modified or rephrased sections highlighted in **CYAN** to facilitate easy identification of changes.

We greatly appreciate your time and consideration, and we look forward to any further feedback or recommendations you may have.

Thank you and best regards,

Diya Rajasekhar Chinta

| **S.No** | **Journal Requirements:** |
| --- | --- |
| **1** | When submitting your revision, we need you to address these additional requirements.    Please ensure that your manuscript meets PLOS ONE's style requirements, including those for file naming. The PLOS ONE style templates can be found at  <https://journals.plos.org/plosone/s/file?id=wjVg/PLOSOne_formatting_sample_main_body.pdf> and  <https://journals.plos.org/plosone/s/file?id=ba62/PLOSOne_formatting_sample_title_authors_affiliations.pdf>  **Action taken:** Complied with the journal’s style requirements. |
| **2** | Please note that PLOS ONE has specific guidelines on code sharing for submissions in which author-generated code underpins the findings in the manuscript. In these cases, we expect all author-generated code to be made available without restrictions upon publication of the work. Please review our guidelines at <https://journals.plos.org/plosone/s/materials-and-software-sharing#loc-sharing-code> and ensure that your code is shared in a way that follows best practice and facilitates reproducibility and reuse.  **Not applicable as we did not generate any code.** |
| **3** | In the online submission form, you indicated that “Any additional data can be provided on request, as the other components of research related to this drug is still on going.”  All PLOS journals now require all data underlying the findings described in their manuscript to be freely available to other researchers, either a. In a public repository, b. Within the manuscript itself, or c. Uploaded as supplementary information.  This policy applies to all data except where public deposition would breach compliance with the protocol approved by your research ethics board. If your data cannot be made publicly available for ethical or legal reasons (e.g., public availability would compromise patient privacy), please explain your reasons on resubmission and your exemption request will be escalated for approval.  **Action taken:** All data pertaining to the manuscript will be made available. |
| **4** | We notice that your supplementary figures are uploaded with the file type 'Figure'. Please amend the file type to 'Supporting Information'. Please ensure that each Supporting Information file has a legend listed in the manuscript after the references list.-  **Action Taken:** Modified and uploaded accordingly. |
|  | **Additional Editor Comments:** |
| **1** | The submission deserves another round of substantial revision to address some critical concerns raised by a reviewer. |
|  |  |

| Sl. No. | Reviewer’s comment | Author’s comment |
| --- | --- | --- |
| Reviewer #1 | | |
| The manuscript is well structured with a robust methodology. The combination of both experimental chemical profiling via GC-MS and a well-established in silico analysis, including reliable MD simulations, standard docking procedures, and MMPBSA energy calculations, provides a solid basis for identifying the active ingredients in VT.  The pharmacological relevance of the study is further highlighted by its focus on TNF-α and IL-17A, which are two clinically validated targets in psoriasis. Furthermore, the authors provided insightful discussion on the pharmacological relevance of the key phytochemicals.  A few points to consider to strengthen the robustness of the discussion:  Overall, the manuscript presents a solid pharmacoinformatics analysis of a conventional antipsoriatic formulation.  **Comment Corrections** | | |
| **1** | Provide more details about the GC-MS compound identification procedure. In the NIST database, what match score threshold was applied? Did any annotations match authentic standards or undergo manual verification? | 1.Information updated under Methods section as well as under Results section.  2. Attached GC-MS report as S1 file. |
| **2** | The ADMET and Lipinski evaluations are informative, but there should be more discussion about the relevance of the formulation route. For instance, dimyristin exhibits poor oral bioavailability and fails Lipinski's criteria despite its strong binding affinity. It may be better suited for topical formulations, especially given the traditional mode of VT application. | More description pertaining to pharmacological relevance is added in Discussion. |
| Reviewer #2 | | |
| In this manuscript, Dayanand et al. reports the analysis of isolated chemicals from an oil with anti psoriasis potentials. Authors detailed interaction of select compounds with inflammatory proteins IL-17A and TNF-α. The findings reported are of scientific value that would be of interest to readers, however the manuscript would benefit greatly from the following recommendations. | | |
| **1** | Grammar editing- Sentences in the introduction section were difficult to read. | Revised Introduction as per the suggestion. |
| **2** | Unit of retention time and mol. wt was not given in Table S1. This should be corrected. | Units are added as per the suggestion. (Please verify S1 Table in S3_File) |
| **3** | Image resolutions are low making it difficult to see important details. Authors should replace images with higher resolution files. | All images are retaken and/or upscaled, used Apex.PACE and made them compliant to the journal’s requirement. Revised numbering of tables and figures accordingly. |
| **4** | Figure legend should follow figures in the result section for better readability. | We did not observe such type of insertions in any of the previously published articles with PLOS One. Hence, we retained Figure numbers, table numbers within the results sections at appropriate places complying with the prescribed format. |
| **5** | For the analysis of the observed interaction, there was no explanation made on the significance of the observed interactions with the amino acids. Do these interactions have any potential of translating into desirable phenotypic response? If yes, are there basis for such conclusions? | Updated with interacting residue information under Discussion. Detailed comparison was made between phytochemical ligand complexes and native ligand interactions with respective proteins. Further, the interactions were compared with some of the known or available monoclonal antibodies and small molecules of respective proteins. |
| **6** | Authors should clarify how they arrived at the conclusion on the pharmacological properties and biological activities of selected compounds. Are the stated activities in relation to the studied respective targets? | The conclusion revised based on the findings from this study and comparisons with structural biological functional aspects of respective proteins. I addition, bed on the pharmacological properties claimed by shortlisted phytochemicals from the literature. |
| **7** | Authors should give detailed citations of literature precedence to support claims on the biological potentials of select compounds, considering that in silico studies are very preliminary. | Appropriate citations were included at respective places in the Discussion as well as in the table showcasing the observations made from the literature. (Please verify citations 19, 20 and S6 Table, See: S3_File). |
| Reviewer #3 | | |
| The current study presents a comprehensive phytochemical and pharmacoinformatics analysis of a traditional antipsoriatic oil formulation, with a focus on its potential activity against the proinflammatory cytokines TNF-α and IL-17A. The introduction is well-structured, and the results and discussion are clearly presented. | | |
| **1** | However, the authors should provide a rationale for selecting TNF-α and IL-17A over other cytokines commonly associated with psoriasis. | The significance of these proteins in the pathogenesis of psoriasis was included in the Introduction also discussed under Discussion. (Please verify citations from 1 to 4 and 30.) |
| **2** | For the molecular docking and molecular dynamics simulations, it is recommended to include appropriate reference or standard controls and compare the performance of the top hits against these benchmarks. | The residue interactions documented in this study from molecular docking and simulations were compared with their respective native ligand interactions. Also compared with the known monoclonal antibodies and small molecule inhibitors of respective proteins. |
| **3** | Additionally, the quality of the figures is suboptimal, please provide high-resolution versions to ensure clarity and better interpretation. | All images are retaken and/or upscaled, used Apex.PACE and made them compliant to the journal’s requirement. |
